# Whole genome duplications have provided teleosts with many roads to peptide loaded MHC class I molecules

**DOI:** 10.1186/s12862-018-1138-9

**Published:** 2018-02-23

**Authors:** Unni Grimholt

**Affiliations:** Fish Research Group, Norwegian Veterinary Institute, Ullevaalsveien 68, 0454 Oslo, Norway

**Keywords:** MHC, Antigen presentation, Peptide loading complex, Phylogeny, Teleosts, Salmonids

## Abstract

**Background:**

In sharks, chickens, rats, frogs, medaka and zebrafish there is haplotypic variation in MHC class I and closely linked genes involved in antigen processing, peptide translocation and peptide loading. At least in chicken, such MHCIa haplotypes of MHCIa, TAP2 and Tapasin are shown to influence the repertoire of pathogen epitopes being presented to CD8+ T-cells with subsequent effect on cell-mediated immune responses.

**Results:**

Examining MHCI haplotype variation in Atlantic salmon using transcriptome and genome resources we found little evidence for polymorphism in antigen processing genes closely linked to the classical MHCIa genes. Looking at other genes involved in MHCI assembly and antigen processing we found retention of functional gene duplicates originating from the second vertebrate genome duplication event providing cyprinids, salmonids, and neoteleosts with the potential of several different peptide-loading complexes. One of these gene duplications has also been retained in the tetrapod lineage with orthologs in frogs, birds and opossum.

**Conclusion:**

We postulate that the unique salmonid whole genome duplication (SGD) is responsible for eliminating haplotypic content in the paralog MHCIa regions possibly due to frequent recombination and reorganization events at early stages after the SGD. In return, multiple rounds of whole genome duplications has provided Atlantic salmon, other teleosts and even lower vertebrates with alternative peptide loading complexes. How this affects antigen presentation remains to be established.

**Electronic supplementary material:**

The online version of this article (10.1186/s12862-018-1138-9) contains supplementary material, which is available to authorized users.

## Background

Currently our view of antigen processing, peptide loading and peptide presentation in teleosts mainly relies on extrapolation of functional data generated in humans, a few other mammals and chickens. In these species Major histocompatibility complex class I (MHCI) molecules are key players in discriminating self from non-self. Classical MHCI (MHCIa) molecules, consisting of an alpha chain and a non-covalently associated beta2-microglobulin (b2m) chain, are displayed at the surface of most cells where they normally present peptides derived from self-molecules chopped into smaller fragments in the cytoplasm. If foreign elements such as viruses are present in the cytoplasm, they are prone for degradation and presentation by MHCIa molecules. After peptide loading, the MHCIa molecules are transported to the cell surface for recognition by CD8+ T cells, thereby initiating an immune response when the peptide originates from a non-self protein.

Newly synthesized MHCIa molecules are processed in the endoplasmic reticulum (ER) to become mature properly folded molecules [reviewed in [1]]. This transition to maturity is aided by the molecular chaperones calnexin (CANX), calreticulin (CALR) and heat shock protein family A member 5 (HSPA5 alias BiP) that assist in correct folding and assembly of the MHCIa molecule with the beta2-microglobulin chain. Subsequent association with ERp57 (protein disulphide isomerase family A member 3 alias PDIA3), an enzyme that catalyzes disulfide bond formation, finalizes maturation of the MHCIa molecule. To form the peptide-loading complex, the CALR and ERp57-stabilized MHCIa molecule then associates with tapasin (TAPBP) and the transporters associated with antigen processing (TAP1- TAP2) heterodimer [2].

Peptide loading of MHCIa molecules is a multi-step process starting with the degradation of proteins in cytosol. Proteins tagged for destruction by ubiquitin are generally degraded by a proteasome consisting of an inner core with seven alpha and seven beta subunits associated with one or two regulatory particles [3]. Upon stimulation such as an infection, a MHCIa specific proteasome defined as the immune-proteasome is induced by interferon gamma where three of the seven proteasome beta-components PSMB8, PSMB9 and PSMB10 replace the constitutive components PSMB5, PSMB6 and PSMB7. This change in components produces peptides preferably with hydrophobic or positively charged residues at the C terminus, which are optimally suited for binding to the antigen transporter TAP and to MHC Ia molecules [4]. Two additional interferon-induced subunits called PSME1 and PSME2 provide an added regulatory element to the core immunoproteasome but how they influence the MHCI peptide repertoire is debated [5]. It should be noted that the interferon-inducible immunoproteasome components have not been identified in birds [6].

The protein degradation products generated in cytosol are then transported into the ER through an MHCIa specific transporter consisting of the molecules TAP1 and TAP2. This heterodimer preferentially translocates peptides with a length of 8–12 residues into the ER in an ATP-dependent process [7, 8]. Longer peptides may be transported, but then in a kinked conformation as TAP has a length restriction for the peptide N and C-terminal distance [9]. The three N-terminal and the last C-terminal residues of the peptide are critical for binding to TAP [10–12].

In the ER lumen, the MHCIa-b2m-CALR-ERp57 complex associates with tapasin (TAPBP) and transporter associated with antigen processing TAP1/TAP2 into what is defined as the peptide-loading complex (PLC) [13]. TAPBP acts as a bridge between TAP and MHCIa enhancing TAP stability and peptide translocation. TAPBP also stabilizes empty MHCIa molecules and optimizes MHCIa loading with peptides. MHCIa alleles have a variable TAPBP dependency where some alleles do not require TAPBP for loading with high affinity peptides [14]. TAPBP is also linked to the protein disulphide isomerase ERp57, contributing to structural stability of the PLC [15]. To finalize the functional PLC complex, ERp57 is linked to calreticulin, while calreticulin holds on to the MHCIa glycan located at the C-terminal end of the alpha 1 domain [16].

As the length of peptides translocated into the ER varies, some need N-terminal trimming to fit properly into the MHCIa groove as the C-terminus is already MHCIa compatible. ERAP1/ ERAP2 can trim both free peptides as well as MHCIa-bound peptides [17]. Optimal MHCIa peptide binding may also be influenced by the tapasin-related molecule (TAPBPR) although this molecule is not a member of the PLC [18, 19]. Similar to TAPBP, MHCIa alleles vary in their association with TAPBPR [19]. Recently, Neerincx et al. [20] showed that TAPBPR associates with UDP-glucose:glycoprotein glucosyltransferase 1 (UGT1), a folding sensor in the calnexin/calreticulin quality control cycle that is known to regenerate the Glc1Man9GlcNAc2 moiety on glycoproteins. Thus, TAPBPR could serve a dual role also routing empty or low-affinity bound MHCIa molecules back to the PLC for refolding.

In Chicken, there is a classical dominantly expressed and highly polymorphic MHCIa (BF2) gene residing within the major MHC region. This gene is flanked by TAP1 and TAP2 genes while a single TAPBP gene is located less than 40 kb away [21–23]. The chicken tapasin and TAP genes are polymorphic with each unique BF2, TAP1, TAP2 and TAPBP combination segregating as a stable functional haplotype. TAPBP and TAP polymorphism govern the peptides available for binding to the BF2 allele [24, 25]. Thus BF2 alleles in heterozygous animals can in theory bind peptides pumped by both haplotypes as opposed to alleles in homozygous animals, thus broadening the MHCIa peptide repertoire. The importance of peptide binding can be exemplified by Mareks disease in chicken, where MHCIa alleles binding a large variety of peptides induce protection while those binding a restricted number of peptides are linked to susceptibility. Presumably, presenting a larger repertoire of pathogen epitopes activate a wider range of T cell clones, a response needed for protection against this pathogen. To counteract against inducing autoimmune reactions, the MHCIa molecules with a wide peptide binding repertoire are only present in low copy numbers on the cell surface while MHCIa molecules with a narrow peptide repertoire are much higher expressed on the cell surface [24, 25]. A similar picture was described in humans, where also here lower surface expression levels corresponded with a broader peptide binding ability. Although TAPBP and TAP are not polymorphic in humans, allelic variation in how the molecules associate with TAPBP was suggested to influence the peptide binding repertoire.

Polymorphism in genes closely linked to MHCIa is also reported in other tetrapods. In rats, two allelic variants of TAP2 linked to specific subsets of MHCIa were shown to deliver different spectrum of peptides [26, 27]. In frogs, linked and highly divergent biallelic PSMB8, TAP1, TAP2 and MHCIa sequence variants of ancient origin have been described [28, 29]. Unfortunately the functional difference between these frog gene variants has not yet been investigated.

Similar to frogs both the PSMB8, TAP2 and tapasin molecules are encoded within the major MHC class I regions of teleosts [30]. This region also contains a teleosts specific duplication of the PSMB9 gene denoted PSMB12 and a gene duplicate of the human PSMB10 gene denoted PSMB13. The teleost PSMB10 gene is for some species located within the extended MHCIa region while for others translocated elsewhere [30, 31]. Polymorphism in the proteasome component PSMB8 has been reported [32, 33] where two distinct lineages of PSMB8 denoted PSMB8A and PSMB8F were found in sharks, cyprinids and salmonids [34]. The two variants existed as duplicate genes in shark, but were defined as alleles in cyprinids and salmonids by Tsukamoto et al. [34], while McConnell et al. defined them as paralogs [31]. When comparing gene sequences from three medaka MHCIa haplotypes, sequence polymorphism was observed both within the PSMB8A gene as well as in the PSMB10 gene [33]. Recently haplotypic variation was also reported in zebrafish where haplotypes contained a varying number of MHCIa genes were linked to polymorphic TAP2, tapasin and PSMB8 molecules [31]. Thus, functional haplotypes linked to the classical MHCIa genes may be a common trait in teleosts.

Atlantic salmon is a species with a single classical MHCIa gene [35] similar to chicken, but with a duplicated MHCI region due to the unique salmonid whole genome duplication (SGD) event that occurred 94 million years ago [30, 36]. The MHCIa region on chromosome 27 harbors the single classical MHCIa locus denoted UBA, while the paralog MHCIb region on chromosome 14 has one expressed non-classical MHCI gene denoted UDA and one or two pseudogenes denoted UCA [37, 38]. The MHCI genes in both regions are flanked by tapasin, the proteasome components PSMB8, PSMB9, PSMB12, PSMB13 and TAP2 alongside several other genes also present in the MHCI region of other teleosts. Similar to other teleosts, the TAP1 gene is located outside the major MHCI region. Based on the reported MHCIa haplotypes in sharks, rats, chicken, frogs, zebrafish and medaka, we set out to investigate the peptide loading machinery and functional MHCIa haplotypes in salmonids compared to other teleosts.

## Results

**Fig. 1:**
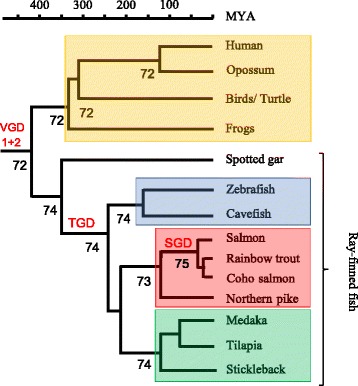
Phylogeny of tetrapod and ray-finned species with approximate dating (MYA, Million Years Ago) shown on top. Timing of the first and second vertebrate whole genome duplication (VGD1 + 2), the teleost specific whole genome duplication (TGD) and the salmonid whole genome duplication (SGD) events are shown using red font. Tetrapods are shown using orange box, Ostariophysian species are shown using blue box, salmonids are shown using red box and neoteleosts are shown using green box. Relevant literature for the respective branch knots [71–74] is indicated

The fact that sequence variation has been found in genes closely linked to MHCI that influence peptide processing, transport and loading in a diverse range of species including the teleosts medaka and zebrafish (See Table 1) could indicate that this is a general trend. To investigate functional MHCIa-linked polymorphism in salmonids we looked at available genomes from Atlantic salmon [39], rainbow trout ([40] and unpublished assembly) and coho salmon (unpublished assembly) as well as Northern pike [41], a species basal to the salmonids that has not experienced the salmonid whole genome duplication event. For Atlantic salmon and trout there are also some BAC sequences covering the paralog MHCI regions [37, 42]. See Fig. 1 for phylogenetic relationships between the various teleost species.

**Table 1:**
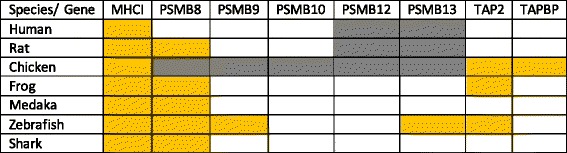
Polymorphism in MHC class I linked genes

Orange cells indicate reported gene polymorphism in MHCI-linked genes from rats [26, 27], chicken [22, 23], frogs [29, 78], medaka and sharks [34] and zebrafish [31]. Grey cells indicate lack of immunoproteasome components [6]. White cells indicate no reported polymorphisms

### PSMB8F linked haplotypes

Based on data from medaka [33, 43, 44] and zebrafish [31] (summarized in Additional file 1; Fig. S1), a good marker for functional teleost MHCIa haplotypes is the PSMB8F gene variant. The term haplotype here defines a group of closely linked gene variants (alleles) residing within one chromosomal region that are inherited together from a single parent. Thus to investigate polymorphism in genes involved in antigen processing in salmonids, we first searched salmonid genome resources for presence of the PSMB8a and PSMB8F variants reported to exist as allelic variants in salmonids [34]. In the following we use -a and –b extensions for paralog genes originating from the salmonid whole genome duplication. In the case of MHCI, Ia and –a extensions refer to genes originating from the salmonid classical UBA regions and Ib to genes in the paralog region containing non-classical MHCI genes respectively. Each chromosomal region containing MHCI and physically linked genes is in the following shown as e.g. Atlantic salmon Ia_#A referring to a specific collection of gene variants in the Ia region while Ia_#B refers to another collection of gene variants in this same region.

**Fig. 2:**
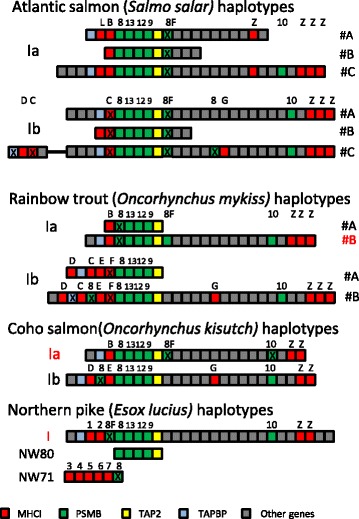
Overview of the included MHC class Ia and Ib haplotypes from Atlantic salmon (*Salmo salar*), Rainbow trout (*Oncorhynchus mykiss*) and Coho salmon (*Oncorhynchys kisutch*) in addition to the assumed Northern pike (*Esox lucius*) allelic haplotypes represented by the Chr. 10 region (Ia) and the unplaced genomic scaffolds NW80 (NW_017859580.1) and NW71 (NW_017859271.1). Color coding for individual genes are shown on the bottom of the figure. Sequences originate from either previously published BAC sequences [Rainbow trout haplotype Ia_#A and Ib_#A: [42]; Atlantic salmon haplotypes Ia_#A&#B, Ib_#A&#B: [37, 38]], from genomes available in GenBank [Atlantic salmon Ia_#C and Ib_#C: [39], Northern pike: [41]] or unpublished genome assemblies for rainbow trout and Coho salmon. See Materials and Methods for additional references. A 7.2 Mb region dividing MHCI-related genes in the Atlantic genome Ib region is indicated by a black line. Gene boxes are colored red for MHCIA, dark green for PSMB subunits, yellow for TAP2, blue for TAPBP and grey for remaining genes. Individual genes are shown above the boxes using abbreviations B, C, D, E, F, L, G for individual U lineage genes; Z for Z lineage genes; 8, 8F, 9, 10, 12, 13 for individual PSMB genes; 1 through 7 for undefined MHCI lineage genes in Northern pike. X denotes pseudogenes. More regional details can be found in Additional file 1: Figure S1

**Fig. 3:**
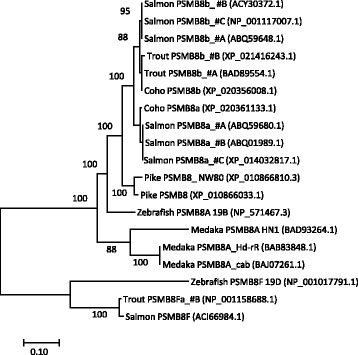
Phylogeny of deduced PSMB8 amino acid sequences from selected species. The evolutionary history was inferred by using the Maximum Likelihood method based on the JTT matrix-based model [75]. The percentage of trees in which the associated taxa clustered together (100 bootstrap trials) are shown next to the branches. The tree is drawn to scale, with branch lengths measured in the number of substitutions per site. All positions with less than 95% site coverage were eliminated. Salmonid and Northern pike PSMB8 sequences originate from the haplotypes described in Materials and Methods with the exception of the Atlantic salmon PSMB8F sequence which is from GenBank with accession number in parenthesis. Zebrafish haplotype sequences are from McConnell et al. [31] and medaka haplotype sequences are from Hd-rR [43], HN1 [44] and cab [33]. Accession numbers are shown in parenthesis. Pseudogene fragments are not included in the phylogenetic analysis, but are included in Additional file 1: Text S1 and Additional file 1: Text S2. The tree is unrooted and some bootstrap values are not shown for clarity

None of the previously published MHCIa or MHCIb regions from Atlantic salmon [37, 38] nor those in rainbow trout [42], determined from sequencing of BAC clones, contained a bona fide PSMB8F gene (Figs. 2 and 3, Additional file 1: Figure S1, Additional file 1: Text S1). Neither was this presumed allelic variant present in the paralog MHCI regions of the Atlantic salmon genome originating from a double haploid [39]. However, in both assemblies of the rainbow trout genome [[40] & new unpublished GenBank assembly GCA_002163495.1)] we found the PSMB8F gene located in between the TAP2 and the BRD2 loci in the Ia region (Fig. 2, trout haplotype Ia_#B). Although automatically annotated as two separate open reading frames in the most recent genome assembly (70,115+ CDQ70116) this could well constitute a functional locus in trout as supported by the GenBank transcriptome shotgun assembly sequences GBTD01219587.1 and EZ768376.1. This Ia_#B haplotype also contained a PSMB8 pseudogene in between the UBA and the PSMB13a genes, similar to the location of the PSMB8 gene in the majority of other analysed teleosts [30]. The pseudo-nature of this locus is supported by a PSMB8 pseudogene in this position also in the previously published trout Ia_#A haplotype [42]. Looking back at the published trout MHCI BAC sequences, both these Ia_#A and Ib_#A haplotypes terminated immediately following the TAP2 locus so the PSMB8F gene could potentially also have been present in these haplotypes. Based on the pseudogene nature of the PSMB8 gene in both the trout Ia_#A and Ia_#B haplotypes, perhaps this is compensated for by a functional PSMB8F gene. For the coho (*Oncorhynchus kisutch*) genome, a PSMB8F gene is also located in between the TAP2 and BRD2 genes of the Ia region in this species (Fig. 2, Additional file 1: Figure S1, Additional file 1. Text S2), but based on the genome assembly this is a pseudogene.

**Fig. 4:**
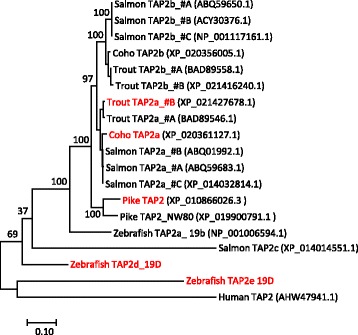
Phylogeny of deduced TAP2 amino acid sequences from selected species. The evolutionary history was inferred by using the Maximum Likelihood method based on the Le and Gascuel 2008 model [76]. The percentage of trees in which the associated taxa clustered together (100 bootstrap trials) are shown next to the branches. The tree is drawn to scale, with branch lengths measured in the number of substitutions per site. Positions with less than 95% site coverage were eliminated. For sequences not originating from the selected haplotypes accession numbers are shown in parenthesis. Gene sequences linked to haplotypes containing the PSMB8F gene variant (Fig. 2) are shown using red font. Sequence references not shown in figure can be found in [31]. The tree is unrooted and some bootstrap values are not shown for clarity

**Fig. 5:**
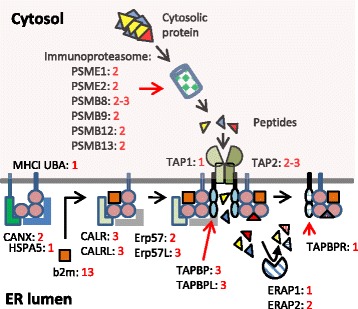
A visual summary of the complexity in number of genes involved in MHCI peptide cleavage, peptide transport, peptide loading and editing in Atlantic salmon. Endoplasmatic reticulum (ER) lumen and cytosolic compartments are shown. Number of Atlantic salmon genes per each human gene ortholog is shown using red font. Like (L) extensions are used when Atlantic salmon sequences exist in duplicates with one groups clustering closer to the human ortholog than the gene-like sequences. Black arrows indicate movement through the compartments while red arrows point to location of molecules

**Fig. 6:**
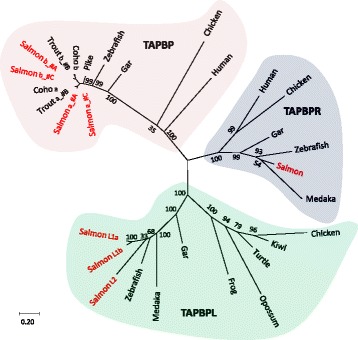
Phylogeny of TAPBP, TAPBPR and TAPBPL amino acid sequences from selected species. The evolutionary history was inferred by using the Maximum Likelihood method based on the Whelan and Goldman model [77]. The tree is drawn to scale, with branch lengths measured in the number of substitutions per site. The percentage of trees in which the associated taxa clustered together (100 bootstrap trials) are shown next to the branches. All positions with less than 95% site coverage were eliminated. Sequence references are as follows: Atlantic salmon (*Salmo salar*) TAPBPa_#C NP_001117077.1, TAPBPR NP_001133983.1*,* TAPBPL1a XP_014069540.1, TAPBPL1b XP_014017660.1, TAPBPL2 XP_014062182.1; Northern pike *(Esox Lucius*) TAPBP XP_010899738.2; Zebrafish (*Danio rerio*) TAPBP GDQH01003123.1, TAPBPR XP_001919985.2, TAPBPL AAI71514.1; Medaka (*Oryzias latipes*) TAPBPR XP_011483883.1, TAPBPL XP_004075780.1; Spotted gar (*Lepisosteus oculatus*) TAPBP GFIM01016833, TAPBPR XP_015193320.1, TAPBPL GFIM01040944.1; Frog (*Xenopus laevis*) TAPBPL XP_018100952.1; Turtle (*Chrysemys picta bellii*) TAPBPL XP_005298961.1; Chicken (*Gallus gallus*) TAPBP1 NP_001029988.2, TAPBPR NP_001026543.1, TAPBPL merged transcripts BU342879.1, BU369515.1 and BX257449.3; Kiwi (*Apteryx australis mantelli*) TAPBPL XP_013817376.1; Opossum (*Monodelphis domestic*) TAPBPL XP_007485846.1; Human (*Homo sapiens*) TAPBPR NP_060479.3, TAPBP NP_003181.3. See Additional file 1: Text S1 and Additional File 1: Text S4 for sequences and amino acid alignment. The tree is unrooted and some bootstrap values are not shown for claritys

As opposed to rainbow trout, there seems to be a bona fide coho PSMB8 gene in the expected position in between the UBA and PSMB13a loci. Thus, the trout and coho data do not support the proposed allelic nature of the PSMB8A and PSMB8F sequence variants suggested by Tsukamoto et al. [34], but rather suggests that they are two different loci residing at different locations within the salmonid MHCIa region. This is further supported by small PSMB8F pseudogene fragments in between the TAP2 and the BRD2 gene in all Atlantic salmon Ia and Ib haplotypes (Fig. 2, Additional file 1: Figure S1, Additional file 1: Text S1, Additional file 1: Text S2), supporting the location of this gene in this position in a predecessor to Atlantic salmon, coho salmon and rainbow trout. There are also PSMB8 fragments elsewhere in salmonid genomes such as in between the trout Ib UCA and UEA genes and the coho Ib region UEA and UDA genes suggesting this gene has been shuffled around during salmonid evolution. Northern pike, a diploid species basal to the salmonids [41], does not add any clarity to the evolution of these genes, as it contains a PSMB8F pseudogene in between a seemingly functional PSMB8 gene and two duplicate MHCI genes. We did however find expressed PSMB8F match for both Northern pike (GATF01015276.1) as well as Atlantic salmon (ACI66984.1) (Additional file 1: Text S2), suggesting this gene may be functional in some haplotypes.

In zebrafish the PSMB8F gene is located in a unique haplotype on chromosome 19 denoted 19D alongside a single classical MHCIa gene denoted UGA and two highly divergent TAP2 genes [31]. The two other haplotypes A and B described by McConnell et al. [31] contain the PSMB8A variant only in a location different from the PSMB8F gene in the 19D haplotype (Additional file 1; Figure S1). It should be noted that this zebrafish UGA gene is not identical to the non-classical gene denoted UGA in salmonids. Even if the PSMB8F gene has been silenced in most trout, coho and Northern pike haplotypes, there should still be remnants of other gene polymorphisms in these species. We thus contrasted closely linked gene sequence variants in PSMB8F positive regions against those in Atlantic salmon regions without the PSMB8F variant (Fig. 2). Based on data from zebrafish and medaka suggesting that TAPBP, TAP2 and PSMB variants have evolved to serve specific MHCIa alleles [31, 34], we first investigated the phylogenetic relationship amongst the MHCIa gene sequences. As teleosts have alpha 1 domain lineages that cluster into lineages shared between distantly related species [30, 33, 45] this analysis was performed based on individual alpha 1–3 domains.

Alpha I domain sequence of the rainbow trout UBA locus, the second pike UBA locus and the zebrafish UGA locus all cluster together alongside other alpha 1 domain sequences from lineage V (Additional file 1: Figure S2) [30, 45]. The two other PSMB8F containing regions did not comply with linkage of the MHCIa alpha 1 domain region and the PSMB8F gene sequence, as the coho a1 domain sequence clusters alongside lineage I sequences and the pike UBA1 sequence clusters alongside lineage III sequences. Comparing pike with the Atlantic salmon MHCIa region could suggest that the pike UBA1 locus is an ortholog of the Atlantic salmon ULA locus while the pike UBA2 locus is the ortholog of the salmon UBA locus, although the pike UBA1 locus still contains a transmembrane region as opposed to the salmon ULA gene. If so, the only deviation from a functional haplotype including MHCIa gene variants and the PSMB8F gene is coho based on the alpha 1 domain analysis. However, the sequenced Northern pike animal was not a double haploid as seen in the two unassembled scaffolds containing five additional U lineage loci and a duplicate PSMB-TAP2 region (Additional file 1: Text S1, scaffolds NW_017859271.1 and NW_017859580.1) which most likely represents an allelic variant. There is currently no data available enabling definition of these pike genes as classical or non-classical referring to polymorphic content, peptide binding ability and tissue expression distribution.

We know that the alpha 1 domain in salmonids fluctuate between different alpha 2 and downstream domains [30, 45–48] so potentially there has been a translocation or recombination of the coho alpha 1 domain, losing the alpha 1 domain V lineage in this haplotype. This is consistent with the clustering of the alpha 2 domain of coho and rainbow trout alpha 2 domains with other lineage I alpha 2 domains (Additional file 1: Figure S3). But for the alpha 2 domain zebrafish UGA clusters with alpha 2 domain lineage II sequences with a convincing bootstrap value and the pike sequences form a separate clade unlinked to the other PSMB8F-linked haplotype sequences. For the alpha 3 domain, the sequences cluster in a species specific manner providing no further clues as to functional haplotypes. Thus, the MHCI gene sequence analyses does not convincingly support functional haplotypes represented by the PSMB8F gene sequence in salmonids.

Zebrafish did not contain any polymorphism in the PSMB12 gene sequences, but some sequence variation in the PSMB9 gene [31]. For salmonids, the salmonid PSMB9 and PSMB12 genes had more species specific sequence variation than haplotypic variation and none of the variable sites in the PSMB9 sequences matched the sequence variation seen in zebrafish (Additional file 1: Figure S3).

Of the phylogenetically related zebrafish PSMB7, PSMB10 and PSMB13 genes, only the PSMB13 gene displayed haplotypic sequence variation. Zebrafish PSMB7 and PSMB10 genes were unlinked to any MHC genes residing on chromosome 21 and 4 respectively [31]. In salmonids both the PSMB13 and the PSMB10 genes reside within both the paralog MHCI region where the PSMB10 gene is located closer to the MHCI Z lineage genes than to the U lineage loci (Fig. 2 and Additional file 1: Figure S4). The Atlantic salmon PSMB7 gene resides on linkage group 1 with a pseudogene copy on linkage group 11 (Additional file 1: Text S1). As opposed to zebrafish, there is no indication of any haplotypic variation in any of these PSMB8F linked genes in any salmonid species. As an example the rainbow trout Ia_#A haplotype contains a UBA*0501 allele while the trout Ia_#B haplotype contains a new yet unnamed allele, but their PSMB13 sequences are identical. Ditto for Atlantic salmon where the PSMB13 sequences are identical in the Ia_#A and Ia_#C haplotypes containing UBA*0201 and UBA*0301 alleles respectively.

In zebrafish, the TAP2 alleles linked to the B and D haplotypes are highly divergent [31]. In salmonids the most prominent sequence variation is seen in the Atlantic salmon TAP2a_#C sequence where there are 11 amino acid differences within the first 31 amino acids in the N-terminal region when compared to TAP2 variants of the other two MHCIa haplotypes (Fig. 4 and Additional file 1: Text S3). This TAP2 sequence resembles both those in the rainbow trout Ia region as well as those in the paralog Ib region, but expressed support (transcriptome shotgun assembly sequence GBRB01034973.1) suggests it is a true sequence and not a genome assembly artefact. Northern pike on the other hand, does display considerable variation in the TAP2 gene sequences located in the main Ia region and the NW80 scaffold (Figs. 2 and 4). Sequence identity between the two pike TAP2 gene sequence variants is 87% which is intermediate in comparison to the 98%, 96%, 71% and 61% amino acid sequence identities observed between the chicken, rat, frog and zebrafish TAP2 sequence variants respectively. Nine residue positions coincide with polymorphic residues in rat or chicken and many variable positions are located within and surrounding the first transmembrane domain known to interact with tapasin [49].

For the salmonid tapasin (TAPBP) gene, there is less sequence information available, as many of the sequenced BACs did not cover this locus (Fig. 2). There are a few amino acid differences between salmon, trout and coho, but no apparent haplotypic variation. Atlantic salmon also has a third TAPBP gene linked to a duplicate UDA locus 7 Mb upstream from the major Ib region, but this TAPBP gene seems to be a pseudogene (Additional file 1: Figure S5).

Based on the above analyses of salmonid MHCI haplotype gene sequences, we did not find evidence for polymorphism in antigen processing genes with the exception of a TAP2 sequence variant in Atlantic salmon. This Atlantic salmon TAP2a_#C sequence differs in many of the first 31 amino acid residues of the N-terminal region a variation that could influence binding affinity for TAPBP [49], but does not match the sequence variation seen in zebrafish, rat or chicken TAP2 sequences (Additional file 1: Text S3). Lack of polymorphism in the selected genes is supported by current and previous sequence analyses using other GenBank resources. With Northern pike displaying a TAP2 polymorphism matching many of the polymorphic residue positions found in other species, the lack of functional haplotypes in salmonids may be unique.

### What about other genes influencing MHCI assembly and peptide loading?

A paralog MHCI region with functional gene copies of PSMB8, PSMB9, PSMB12, PSMB13 and TAP2 (Table 2) provides salmonids with an added complexity in comparison with zebrafish and medaka. This complexity of having many duplicate immune genes which potentially have acquired slightly new functions as suggested by Lien et al. [39] could also relate to other genes involved in antigen degradation, transport and loading. This spurred us to investigate a broader set of genes influencing antigen processing, transport and loading. Calnexin, which binds newly synthesized MHCI prior to assembly with b2m, is encoded by two duplicate loci in Atlantic salmon (Fig. 5, Additional file 1: Figure S6). Both genes display fair expression in immunologically important tissues such as head kidney and spleen as well as other organs such as brain and ovary (Table 2). This gene duplication has previously been identified in trout [50] and is a remnant of the SGD displaying high sequence identity between paralogs. If one or both copies serve the classical MHCIa molecules remains to be established.

**Table 2:**
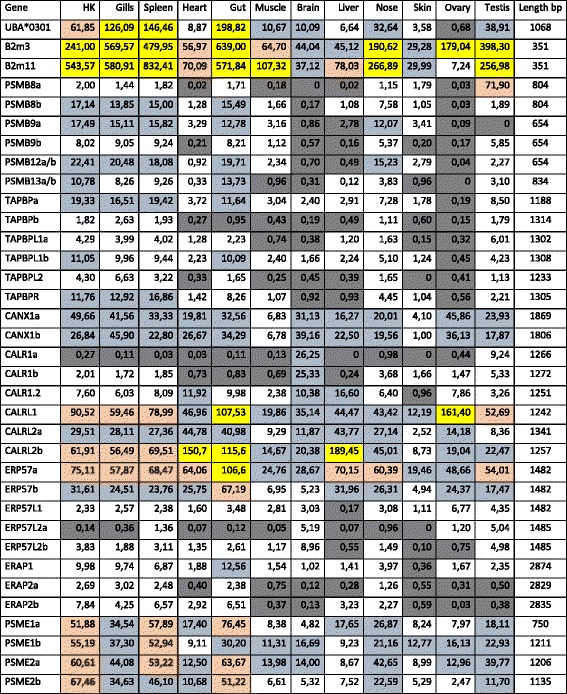
Expression levels of Atlantic salmon genes in various tissues

Read mapping was performed with the stringency of 98% identity and 95% coverage. Tissues with RPKM values below 1 are shaded grey, those with RPKM values between 10 and 50 are shaded blue, those with RPKM values above 50 are shaded pink, and those with levels above 100 are shaded yellow. Nucleotide sequence lengths used in the analysis is indicated in base pairs where only open reading frame sequences were included. Gene names with a and b extensions indicate SGD paralog genes. Sequence identity between PSMB12a/b and PSMB13a/b paralogs was too high to separate between duplicates. The b2m3 and b2m11 sequences represent each of the two sequence groups as sequence identity within group was too high to provide reliable data

The calnexin MHCI heavy chain complex then associates with beta2-microglobulin (b2m) (Fig. 5). This b2m gene is encoded by 3–10 loci in trout [51, 52] while the Atlantic salmon genome contains 13 loci where at least one seems to be a pseudogene (Additional file 1: Text S1). These 12–13 Atlantic salmon gene sequences cluster into two distinct clades with 82% amino acid identity (data not shown). Due to high sequence identity within each clade, it is difficult to assess the expressed status of each Atlantic salmon locus (Table 2). Many other teleosts also have two clades of b2m gene sequences [53], but here the amino acid sequence identity between clades is mostly lower than between the salmonid clades (Additional file 1: Figure S7). The gene duplications have occurred individually in many species with for instance unique gene duplications in Ostariophysi represented by zebrafish and Mexican tetra and another unique duplication in neoteleosts represented by tilapia, medaka and stickleback. Which molecules these divergent teleost b2m molecules support is unknown, but at least in medaka and stickleback the two b2m sequence variants can only serve the U and Z lineages as the remaining MHCI lineages S, L and P are not present in these species [30]. A more speculative idea would be that both b2m sequence groups bind to the classical MHCIa molecule and as such influence the peptide repertoire as previously reported for mouse MHCI molecules [54].

Once the b2m molecule is associated with the MHCI alpha chain, calnexin is replaced by calreticulin (Fig. 5). In Atlantic salmon there are three CALR genes, but also three calreticulin-like (CALRL) genes (Additional file 1: Figure S6) with amino acid sequence identity between the two groups ranging from 67 to 70% (data not shown). Phylogenetically, the gene sequences here denoted Atlantic salmon CALR1a, CALR.1b, CALR1.2 cluster with the human CALR gene sequence and also the previously published catfish sequence denoted CALRL2 [55]. The remaining three Atlantic salmon gene sequences form a separate cluster alongside the two other catfish sequences denoted catfish CALRL and CALR, clustering with a 99% bootstrap value to the other CALR sequences. Sequences belonging to the CALR and CALRL clades also exist in spotted gar, a species that split off from the teleost lineage prior to the teleost whole genome duplication (TGD) [56] (Fig. 1, Additional file 1: Figure S6), suggesting these genes are remnants of the second vertebrate genome duplication (VGD2). The genes represented by the CALRL1 and CALRL2 clades originated before Ostariophysi branched off from the remaining teleosts having orthologs also in catfish. They are thus a product of the TGD event that occurred approximately 350 million years ago after spotted gar split off from the main teleost lineage [56]. Based on sequence identity, phylogenetic clustering and chromosomal location, the CALR1a and CALR1b genes as well as the CALRL2a and CALRL2b genes originate from the SGD duplication event [39]. Human and chicken CALR3 sequences form an outgroup and have no detectable orthologs in Atlantic salmon.

Both the CALR as well as the CALR-like molecules display fair sequence identity as well as conservation of residues known to influence human CALR glycan binding suggesting both groups bind glycan moieties (Additional file 1: Figure S6). Both teleost CALR and CALRL sequence groups also have an ER retention signal (KDEL) and an acidic C-terminal region known to affect ER-retention and recycling [57], further supporting their functional resemblance of human CALR. However, the CALRL genes are generally expressed approximately 10 times higher than the CALR genes (Table 2). And CALRL expression levels are not restricted to immunologically important tissues as seen with high expression levels also in heart, liver and ovary for some of these genes. In catfish, they found both CALR and CALRL genes to be induced upon infection [55]. In rainbow trout, calreticulin was reported as a single copy gene [58], but this sequence is an orthologue of the Atlantic salmon gene here denoted CALRL2b. A later functional study of this trout CALRL2b orthologue reported limited response to endoplasmatic reticulum stress and also little response to stimulation with the viral mimicry stimulant polyI:C [59]. Future studies are needed to clarify the functional distinction of teleost CALR and CALRL genes.

The next molecule to associate with the MHCI/b2m/CALR complex is ERp57 alias PDIA3 (Fig. 5). As seen for the CALR and CALRL genes, a similar picture emerges for ERp57. Here there are two Atlantic salmon ERp57 genes, but also three additional ERp57-like (ERp57L) genes (Additional file 1: Figure S8). The Atlantic salmon ERp57a/b genes cluster with human and zebrafish ERp57 sequences while the ERp57L sequences form a separate clade. Amino acid sequence identity between the Atlantic salmon ERp57 and ERp57L groups is approximately 60% (data not shown). Spotted gar has both an ERp57 sequence as well as an ERp57L sequence, suggesting this duplication originates from the second VGD2 event (Fig. 1) [56]. Both the ERp57L1 sequence and the ERp57L2a/−L2b sequences have orthologs in zebrafish and is most likely a remnant of the TGD event where also here the duplicates have been retained as expressed copies in Atlantic salmon (Table 2). Both Atlantic salmon ERp57a and -b genes and the two ERp57L2a and -L2b genes originate from the unique SGD event with amino acid sequence identities of 95 and 92% respectively. An ERp57 ortholog to the gene sequence here defined as Atlantic salmon ERp57a has been described both in rainbow trout [60] as well as in seabass [61]. This trout gene was induced upon stimulation by a viral mimicry molecule, but also by ER stressors. How the ERp57L genes would respond to similar stimulations remains unknown but the ERp57a/b genes display a much higher expression level in most tissues compared to the ERp57L genes (Table 2).

Following ERp57 association, the complex then associates with Tapasin (Fig. 5). The TAPBP and TAPBPR gene sequences have previously been described in trout [62] where both genes were shown to respond to viral infection. In addition to the tapasin sequences linked to the MHCI Ia and Ib regions in Atlantic salmon and a single TAPBPR gene, there are also three additional gene sequences with blast match to TAPBPR and TAPBP here denoted TAPBP-like or TAPBPL (Fig. 6, Additional file 1: Text S4). Sequence identity between Atlantic salmon TAPBP, TAPBPR and TAPBPL is similar to the 22% amino acid identity found between the human TAPBP and TAPBPR sequences [63]. Both zebrafish and spotted gar only have one TAPBPL gene suggesting the TAPBPL duplications are unique to salmonids. This previously undefined TAPBPL gene is also present in frogs, turtles, alligators, birds and marsupials, but seems to have been lost in the lineage leading to placental mammals (Fig. 6). The TAPBPL gene duplication thus occurred prior to the split between the tetrapod and the bony fish lineages i.e. is also a remnant of the VGD2 event (Fig. 1). The two TAPBPL1a and TAPBPL1b genes originate from the SGD with an amino acid sequence identity of 88% but these two gene sequences only have 53% identity to the third TAPBPL2 sequence which then seems of a more ancient origin.

Many of the TAPBP and TAPBPR residues known to interact with MHC class I [18, 19, 64] are also conserved in the TAPBPL sequences (Additional file 1: Text S4). The human TAPBP C95 residue known to bind ERp57 [64], is not conserved in any of the other TAPBP, TAPBPR or TAPBPL sequences questioning the relevance of this residue in other species. However, a unique cysteine in the TAPBPL sequences located 11 amino acids further downstream could have a similar function as the human TAPBP C95 residue or it could resemble the unique C94 residue in TAPBPR known to interact with UGT1 [20]. The N-linked glycosylation site at N233 known to interact with CALR [65] is preserved in some of the TAPBP, TAPBPR and TAPBPL sequences. The single TAPBP lysine residues in the transmembrane region associating with TAP [2] is not conserved in the TAPBPL sequences, while some TAPBPL sequences display an ER retention signal as found in mammalian TAPBP [66]. Thus, based on conservation of many MHCI-interacting residues, the glycosylation site used to interact with CALR and the ER retention signal, TAPBPL sequences share more structural similarities with TAPBP than with TAPBPR supporting their name as TAPBPL and not TAPBPRL. Although we can only speculate as to the TAPBPL function, all three Atlantic salmon genes are expressed and their expression profiles resemble that of the TAPBP and TAPBPR genes with some tissue specific patterns (Table 2).

In addition to paralog functional copies of the immunoproteasome components PSMB8–13 [Fig. 2, [37]], Atlantic salmon also has duplicate copies of the interferon-inducible PSME1 and PSME2 regulatory subunits. These duplicates originate from the SGD with amino acid sequence identities of 93 and 91% respectively (Additional file 1: Figure S9) and display fair expression in a wide variety of organs (Table 2). As with the majority of paralogs, we do not know how these paralogs influence the peptide repertoire available for MHCI binding. Atlantic salmon also has four genes for the PSME3 subunit (Additional file 1: Figure S9), but at least in humans this subunit is not interferon inducible and thus not a part of the immunoproteasome. If this also holds true for Atlantic salmon remains to be established.

Once transported inside the ER, the N-terminal end of peptides are further trimmed by ERAP1 and ERAP2 molecules [17]. For ERAP1 there is only one gene, while there are two copies of the ERAP2 gene (Fig. 5, Additional file 1: Figure S10). These two ERAP2 genes originate from the SGD with 93% amino acid sequence identity and both gene copies display low expression levels mainly in immunologically important tissues (Table 2).

## Discussion

As opposed to what has been found in sharks, rats, frogs, chicken, medaka and zebrafish and allelic polymorphism in genes closely linked to the MHCIa gene was not evident in salmonids.

Northern pike, a diploid species basal to the salmonids [41], does display TAP2 polymorphism where many of the variable amino acid residues coincide with variable residue positions found in other species suggesting this polymorphism has been lost en route to salmonids.

It seems odd that there are functional MHCIa haplotypes in zebrafish and medaka but not in salmonids. One explanation for the loss of functional Ia haplotypes is the unique salmonid genome duplication that occurred approximately 94 million years ago [39]. Medaka and zebrafish have not experienced an additional WGD event after the third WGD that occurred in a ancestor to all teleost fish approximately 320 million years ago [56]. WGD provides raw material for evolutionary diversification, but must balance against negative dosage-effects, regulatory errors, negative protein-protein interactions and mitotic mistakes. Most WGDs are followed by a reduplication phase with extensive reorganizations to balance against the negative effects of duplications mentioned above. As seen in Atlantic salmon, a burst of transposon-mediated repeat expansions most likely triggered this reduplication phase resulting in increased homeologue sequence divergence and large chromosomal rearrangements such as fusions, fissions, deletions and inversions [39] that disrupted the possibility for homeologous pairing during mitosis. Potentially this extensive rearrangement and sequence divergence eradicated the functional haplotypes found in other teleost species in part including Northern pike. However, it should be noted that this study only includes a few haplotypes so there may be other haplotypes with polymorphic genes influencing MHCIa peptide processing, transport and loading.

Having paralog MHCI regions, the PSMB, TAP and TAPBP genes in the Atlantic salmon Ia and Ib regions could have evolved to serve MHCI molecules encoded by individual regions where genes within the Ia region serve the UBA molecule while genes within the Ib region serve the UDA molecule. Without functional data, these paralog genes could of course also serve MHCI molecules originating from both regions.

The SGD also provided salmonids with many paralog genes that are retained as extant expressed and presumably functional copies. Lien et al. [39] found that SGD duplicates tended to belong to closely related but still different co-expression clusters suggesting far more instances of neo-functionalization than subfunctionalization. Many Atlantic salmon genes involved in peptide processing, transport and loading exist in multiple expressed copies with sequence identity reflecting which of the three whole genome duplication events they originated from. Atlantic salmon has CALR gene duplicates originating from both the SGD as well as the TGD in addition to a gene duplication that originates from the second vertebrate whole genome duplication (VGD2, Fig. 1). This provides Atlantic salmon with three CALR genes and three CALRL genes. As SGD paralogs tend to acquire novel functions based on transcript expression patterns [39], the CALR and CALRL gene duplicates may provide Atlantic salmon with a variety of CALR-like functions. A similar picture is seen for ERp57 and TAPBP, where gene duplications have provided Atlantic salmon with five expressed ERp57/ ERp57L genes and six expressed TAPBP/ TAPBPL genes. As spotted gar also contains these CALR/ CALRL, ERp57/ ERp57L and TAPBP/ TAPBPL gene duplications, they originate from the VGD2 event and not from the teleost specific genome duplication event. This is further supported by the fact that a TAPBPL gene is also present in the tetrapod lineage with what seems to be a bona fide gene even in opossum. How this TAPBPL molecule affects MHCI peptide loading or editing is unclear, but it may hold new and intriguing functions as seen for the TAPBPR gene [18–20].

It is tempting to speculate that there exists alternative peptide-loading complexes in teleosts based on the presence of duplicate CALR/ CALRL, ERp57/ERp57L and TAPBP/ TAPBPL genes in both zebrafish, salmonids and medaka. If this holds true one may only speculate on which MHC molecules these complexes associate with and how that difference in PLC affects the peptides transported and loaded into the peptide binding groove of these MHCI molecules. Expression data support the CALR, ERp57L and TAPBP/ TAPBPL genes to be functionally related as they all display fairly low expression levels in identical tissues. Expression levels of CALRL and ERp57 are much higher and more diversified suggesting they may have different or additional roles.

## Conclusion

We found no evidence pointing to functional polymorphism in TAP, TAPBP, PSMB8, PSMB9, PSMB12 and PSMB13 genes closely linked to classical UBA alleles in salmonids as opposed to what has been found previously in zebrafish, medaka and several other species. The unique salmonid whole genome duplication has most likely disrupted such haplotypes with frequent recombination between chromosomal paralogs prior to their diversification. However, several whole genome duplications have provided salmonids with many duplicated genes involved in peptide generation, loading and editing most likely broadening their biological function compared to what is found in mammals. A surprise is the functional retention of many genes originating from the second vertebrate whole genome duplication event providing both spotted gar as well as teleosts with potential alternative versions of the peptide-loading complex. Future studies are needed to understand the functional relevance of these gene duplications including the alterative PLCs.

## Methods

### Data mining and bioinformatics

MHC class I haplotype gene sequences originate either from genomes available in GenBank or previously sequenced BAC clones as follows: **Atlantic salmon** (*Salmo salar*) BACs [37, 38]: Haplotype **Ia_#A** BACs 868O01 (EF441211), 92I04 (EF427384.1), 539 M19 (EF427383) and 129P21 (GQ505858); Haplotype **Ia_#B** BAC 714P22 (EF210363); Haplotype **Ib_#A** BAC 8I14 (EF427379); Haplotype **Ib_#B** BAC 438 J08 (FJ969490); Genome assembly GCA_000233375.4 [39] Haplotype **Ia_#C** Chr.27 NC_027326: 10.000.000–10.656.000; Haplotype **Ib_#C** genome Chr.14 region NC_027313:50.800.000–59.500.000. **Rainbow trout** (*Oncorhynchus mykiss*) BACs [42] Haplotype **Ia_#A** BAC AB162342; Haplotype **Ib_#A** BAC AB162343; Trout genome (GCA_002163495.1) Haplotype **Ia_#B** Chr.18 (CM007952.1); Haplotype **Ib_#B** Chr.14 (CM007948.1). **Coho salmon** (*Oncorhynchus kisutch*) genome GCA_002021735.1 haplotype **Ia** region Chr.17 NC_034190: 25300000–26,000,000; Haplotype **Ib** region Chr.14 NC_034187: 22.700.000–23.500.000. **Northern pike** (*Esox Lucius*) genome (GCA_000721915.3) Haplotype **Ia** region Chr.10 (NC_025977.3), and presumed allelic haplotype represented by the unplaced genomic scaffolds NW_017859580.1 (**NW80**) continued with NW_017859271.1 (**NW71**). Some genomic un-annotated gene sequences as well as unlinked gene sequences were identified using various blastN and TblastN searches of Ensembl and NCBI databases uand evolutionary diverged as well as species-specific sequences. Open reading frames were predicted using FGENESH [67] and verified using available expressed resources. Potential genomic assembly errors could influence the analyses. Some smaller pseudogene remnants that did not contribute to evolutionary understanding were neglected. Expressed match was either identified through TblastN search against EST, GenBank nucleotide (cDNA) and available TSA/SRA resources. RPKM values shown in Table 2 were defined using CLC Genomic Workbench 6.0.5 [68] and SRR transcriptome runs from a single individual [39] as follows: HK (head kidney) SRR1422860, gills SRR1422858, spleen SRR1422870, heart SRR1422862, gut SRR1422859, muscle SRR1422866, brain SRR1422856, liver SRR22865, nose SRR1422867, skin SRR1422869, ovary SRR1422871, testis SRR1422872. 1.

### Phylogenetic analysis

All amino acid sequence alignments were performed using Clustal X [69].The phylogenetic trees were inferred using best-fit models calculated by MEGA7 [70] and bootstrapped using 100 replicates.

## Additional files

Additional file 1: **Figure S1**. Teleost MHCI haplotypes. **Text S1**. Deduced amino acid gene sequences. **Text S2**. Alignment of deduced PSMB8 amino acid sequences. **Figure S2**. MHCI data. **Figure S3**. PSMB9 and PSMB12 data. **Figure S4**. PSMB7, PSMB10, PSMB13 data. **Text S3**. Alignment of deduced TAP2 amino acid sequences. **Figure S5**. TAPBP data. **Figure S6**. CANX, CALR and CALRL data. **Figure S7**. B2m data. **Figure S8**. ERp57 and ERp57L data. **Text S4**. TAPBP; TAPBPR and TAPBPL data. **Figure S9**. PSME data. **Figure S10**. ERAP data (PDF 4922 kb)

## Abbreviations

B2m: Beta2-microglobulin
CALR: Calreticulin
CANX: Calnexin
MHC: Major histocompatibility complex
MYA: Million years ago
PSMB: Proteasome subunit beta
SGD: Salmonid-specific whole genome duplication
TAP: Transport associated protein
TAPBP: Tapasin
TGD: Teleost-specific whole genome duplication
VGD: Vertebrate genome duplication
