# Supplementary material for: Whole genome duplications have provided teleosts with many roads to peptide loaded MHC class I molecules
Source: BMC Evol Biol. 2018 Feb 23;18:25. doi: 10.1186/s12862-018-1138-9 (PMC5824609; doi:10.1186/s12862-018-1138-9)
Supplement: Supplementary file 1 — Figure S1. Teleost MHCI haplotypes. Text S1. Deduced amino acid gene sequences. Text S2. Alignment of deduced PSMB8 amino acid sequences. Figure S2. MHCI data. Figure S3. PSMB9 and PSMB12 data. Figure S4. PSMB7, PSMB10, PSMB13 data. Text S3. Alignment of deduced TAP2 amino acid sequences. Figure S5. TAPBP data. Figure S6. CANX, CALR and CALRL data. Figure S7. B2m data. Figure S8. ERp57 and ERp57L data. Text S4. TAPBP; TAPBPR and TAPBPL data. Figure S9. PSME data. Figure S10. ERAP data (PDF 4922 kb) [file 12862_2018_1138_MOESM1_ESM.pdf]

Additional file 1: Figure S1. Teleost MHCI haplotypes

Table of Contents		Page
S1a	Summary of previously published zebrafish and medaka MHCI haplotypes	1
S1b	MHCIa and Ib haplotypes from previously published Atlantic salmon and rainbow trout BACs.	2
S1c	MHC class I haplotypes in Atlantic salmon, rainbow trout, coho salmon and Northern pike genomes.	2

Figure S1a. Summary of previously published zebrafish and medaka MHCI haplotypes.

Zebrafish (*Danio rerio*) haplotype amino acid sequences used in this study originate from McConnell et al.(1) and medaka (*Oryzias latipes*) Hd-rR, HN1 and cab haplotype sequences are from Matsuo et al.(2), Tsukamoto et al.(3) and Nonaka & Nonaka (4) respectively. Genes are color coded dark green for proteasome PSMBs, yellow for TAP2, red for MHCI, blue for TAPBP and grey for other genes. Ψ denotes pseudogenes while red font shows location of PSMB8F sequences. Haplotype numbers and chromosomal location are indicated above and on the side of each region respectively. Zebrafish haplotypes are from (1) and medaka haplotypes from (2) (Hd-rR), (3) (HN1) and (4) (cab).

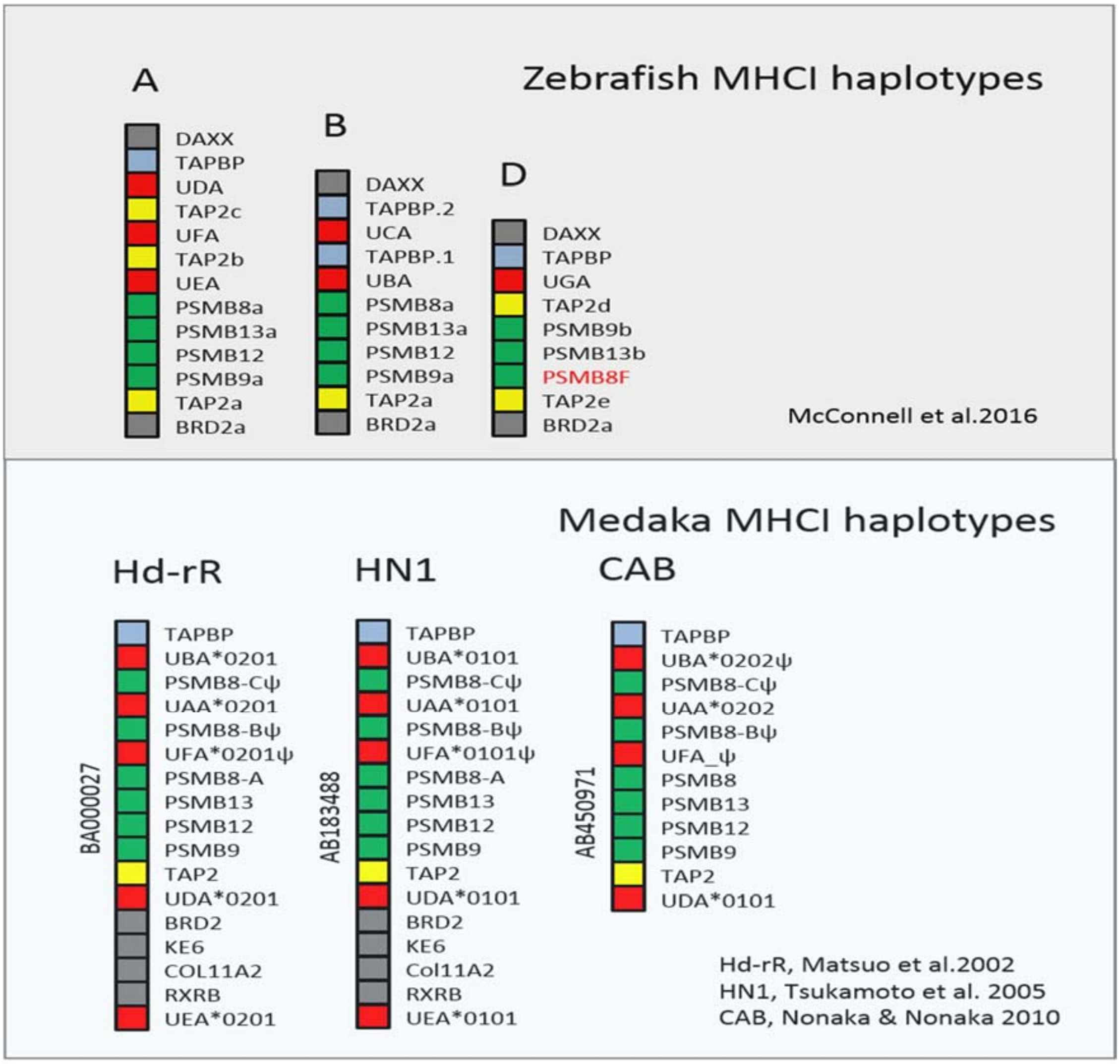

Figure S1b. MHCIa and Ib haplotypes from previously published Atlantic salmon and rainbow trout BACs.

Schematic presentation of previously sequenced BAC sequences from Atlantic salmon and rainbow trout. Salmonid and pike MHC class I haplotype amino acid gene sequences used in this study originate from either previously sequenced BAC clones or genomes available in Genbank as follows: Atlantic salmon (*Salmo salar*) haplotypes from BACs (5, 6): Ia_#A BACs 868O01 (EF441211) and 92I04 (EF427384.1): Ia_#B BAC 714P22 (EF210363); Ib_#A BAC 8I14 (EF427379): Ib_#B BAC 438J08 (FJ969490); Atlantic salmon genome GCA_000233375.4 (7) haplotypes: Ia_#C Chr.27 NC_027326: 10.000.000-10.656.000; Ib_#C Chr.14 region NC_027313: 50.800.000- 59.500.000. Rainbow trout (*Oncorhynchus mykiss*) MHCI haplotypes from BACs (8): Ia_#A BAC (AB162342): Ib_#A BAC sequence (AB162343); Rainbow trout haplotypes from genome GCA_002163495.1 (unpublished GenBank assembly): Ia_#B genome Chr.18 (CM007952.1); Ib_#B genome Chr.14 (CM007948.1). Coho salmon (*Oncorhynchus kisutch*) genome GCA_002021735.1 (unpublished) haplotypes: Ia region LG17 NC_034190: 25300000-26000000, Ib MHCIb region LG14 NC_034187: 22.700.000-23.500.000. Northern pike (*Esox Lucius*) genome GCA_000721915.3 (9) MHCI haplotypes: MHCIa region LG10 (NC_025977.3), presumed allelic haplotype represented by unplaced genomic scaffold NW_017859580.1, and unplaced genomic scaffold NW_017859271.1. More details about each region can be found in Dataset S1. Sequence references are shown vertically alongside each region. Genes are color coded dark green for proteasome PSMBs, yellow for TAP2, red for MHCI, blue for TAPBP and grey for other genes. Ψ denotes pseudogenes. Haplotype numbers are indicated above the regions.

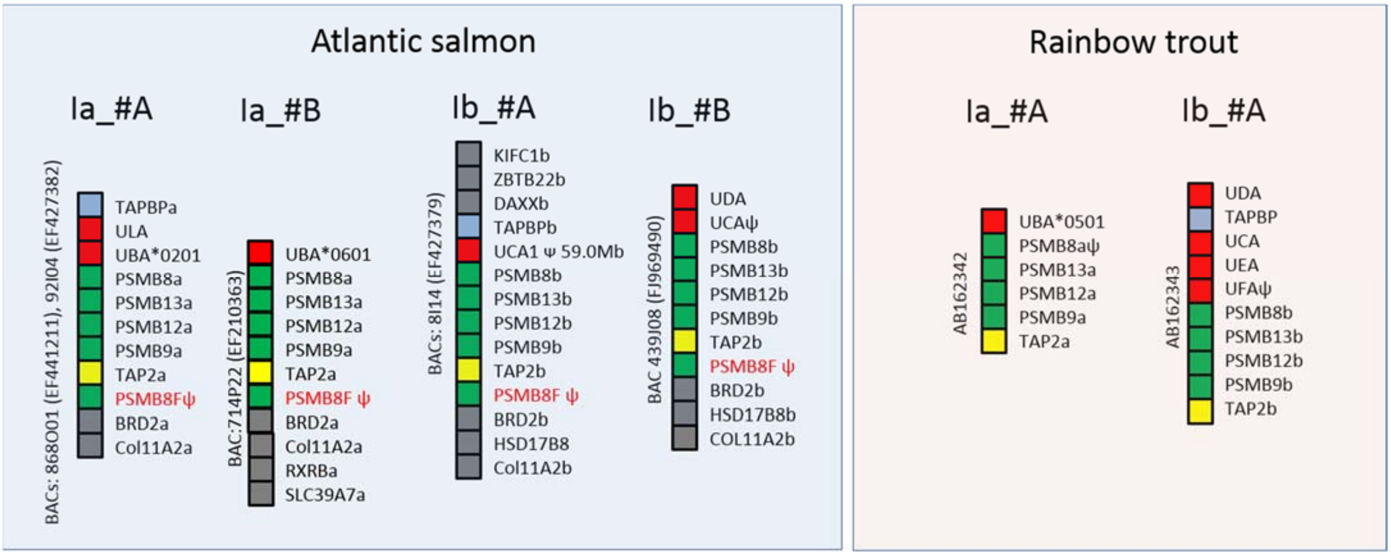

Figure S1c. MHC class I haplotypes in Atlantic salmon, rainbow trout, coho salmon and Northern pike genomes.

Schematic view of duplicate MHCIa and Ib regions in Atlantic salmon (*Salmo salar*), rainbow trout (*Oncorhynchus mykiss*), coho salmon (*Oncorhynchus kisutch*) and MHCIa regions in Northern pike (*Esox lucius*) genomes. Regional references are as follows: Atlantic salmon (*Salmo salar*) haplotypes from genome GCA_000233375.4 (7) haplotypes: Ia_#C Chr.27 NC_027326: 10.000.000-10.656.000; Ib_#C Chr.14 region NC_027313: 50.800.000- 59.500.000. Rainbow trout (*Oncorhynchus mykiss*) MHCI haplotypes from genome GCA_002163495.1 (unpublished GenBank assembly): Ia_#B genome Chr.18 (CM007952.1); Ib_#B genome Chr.14 (CM007948.1). Coho salmon (*Oncorhynchus kisutch*) genome GCA_002021735.1

(unpublished) haplotypes: Ia region LG17 NC_034190: 25300000-26000000, Ib MHCIb region LG14 NC_034187: 22.700.000-23.500.000. Northern pike (*Esox Lucius*) genome GCA_000721915.3 (9) MHCI haplotypes: MHCIa region LG10 (NC_025977.3), presumed allelic haplotype represented by unplaced genomic scaffolds NW_017859580.1, and NW_017859271.1. Sequence references are shown vertically alongside each region. Genes are color coded dark green for proteasome PSMBs, yellow for TAP2, red for MHCI, blue for TAPBP and grey for other genes. Ψ denotes pseudogenes. Haplotype numbers and chromosomal location are indicated above the regions. The trout Ia_#B haplotype contains a new yet undefined UBA allele. The two additional Northern pike scaffolds are most likely haplotypic variation, although organization is undefined in the genome assembly.

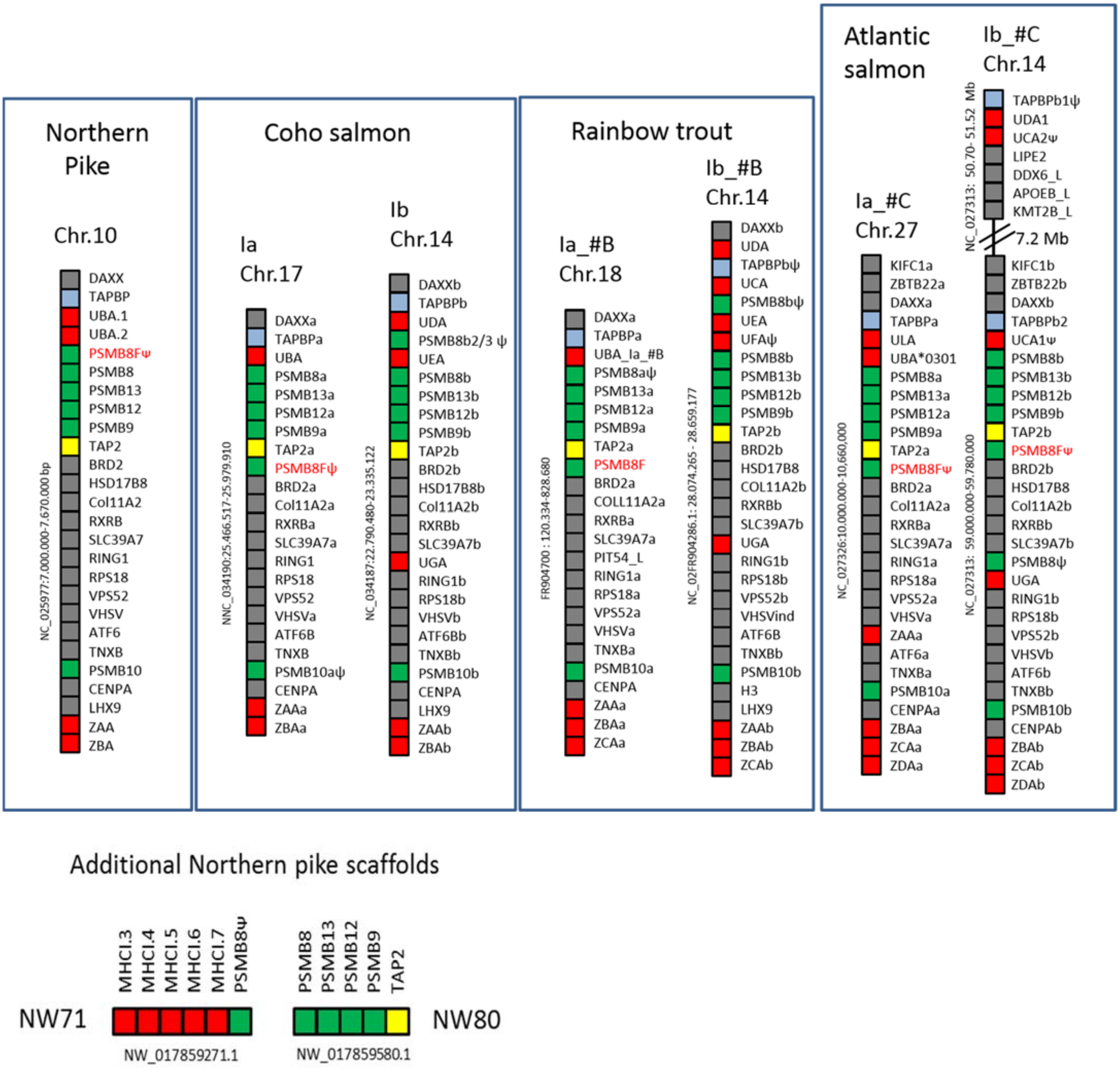

References:

1. McConnell SC, *et al.* (2016) Alternative haplotypes of antigen processing genes in zebrafish diverged early in vertebrate evolution. *Proceedings of the National Academy of Sciences of the United States of America* 113(34):E5014-5023.
2. Matsuo M, Asakawa S, Shimizu N, Kimura H, & Nonaka M (2002) Nucleotide sequence of the MHC class I genomic region of a teleost, the medaka (*Oryzias latipes*). *Immunogenetics* 53:930-940.
3. Tsukamoto K, *et al.* (2005) Unprecedented intraspecific diversity of the MHC class I region of a teleost medaka, *Oryzias latipes*. *Immunogenetics* 57:420-431.
4. Nonaka MI & Nonaka M (2010) Evolutionary analysis of two classical MHC class I loci of the medaka fish, *Oryzias latipes*: haplotype-specific genomic diversity, locus-specific polymorphisms, and interlocus homogenization. *Immunogenetics* 62(5):319-332.
5. Lukacs MF, *et al.* (2010) Comprehensive analysis of MHC class I genes from the U-, S-, and Z-lineages in Atlantic salmon. *BMC genomics* 11:154.
6. Lukacs MF, *et al.* (2007) Genomic organization of duplicated major histocompatibility complex class I regions in Atlantic salmon (*Salmo salar*). *BMC genomics* 8:251.
7. Lien S, *et al.* (2016) The Atlantic salmon genome provides insights into rediploidization. *Nature* 533(7602):200-205.
8. Shiina T, *et al.* (2005) Interchromosomal duplication of major histocompatibility complex class I regions in rainbow trout (*Oncorhynchus mykiss*), a species with a presumably recent tetraploid ancestry. *Immunogenetics* 56(12):878-893.
9. Rondeau EB, *et al.* (2014) The genome and linkage map of the northern pike (*Esox lucius*): conserved synteny revealed between the salmonid sister group and the Neoteleostei. *PloS one* 9(7):e102089.

Additional file 2: Text S1. Deduced amino acid sequences used in this study

Region	Deduced amino acid sequences	Page
Atlantic salmon (*Salmo salar*) haplotypes		
Ia region*	Haplotype Ia_A; BACs 868O01 (EF441211) and 92I04 (EF427384.1) deduced amino acid sequences	2
Ia region*	Haplotype Ia_#B; BAC 714P22 (EF210363) deduced amino acid sequences	3
Ia region	Haplotype Ia_#C; Chr.27 genome NC_027326: 10.000.000-10.656.000; deduced amino acid sequences	4
Ib region*	Haplotype Ib_#A; BAC 8I14 (EF427379) deduced amino acid sequences	5
Ib region*	Haplotype Ib_#B; BAC 438J08 (FJ969490) deduced amino acid sequences	6
Ib region	Haplotype Ib_#C Chr.14 Region NC_027313:50.800.000-51.100.000 deduced amino acid sequences	7
Ib region	Haplotype Ib_#C continued Chr.14 region NC_027313: 59.000.000-59.498.000; deduced amino acid sequences	7
	Other deduced Atlantic salmon amino acid sequences	8
Rainbow trout (*Oncorhynchus mykiss*) haplotypes		
Ia region*	Haplotype Ia_#A; BAC (AB162342) deduced amino acid sequences	13
Ia region	Haplotype Ia_#B; Genome (GCA_002163495.1) Chr.18 (CM007952.1); deduced amino acid sequences	14
Ib region*	Haplotype Ib_#A; BAC sequence (AB162343) deduced amino acid sequences	15
Ib region	Haplotype Ib_#B; Genome Chr.14 (CM007948.1) deduced amino acid sequences	16
Coho salmon (*Oncorhynchus kisutch*) genome GCA_002021735.1		
Ia region	LG17 region NC_034190: 25300000-26000000 deduced amino acid sequences	18
Ib region	LG14 region NC_034187: 22.700.000-23.500.000 deduced amino acid sequences	19
Northern pike (*Esox Lucius*) genome GCA_000721915.3		
	MHC I region LG10 (NC_025977.3) deduced amino acid sequences	21
	Unplaced genomic scaffold Un_scaffold0763 NW_017859271.1 deduced amino acid sequences	22
	Unplaced genomic scaffold Un_scaffold1132 NW_017859580.1 deduced amino acid sequences	23
Deduced amino acid sequences from other species		
	Spotted gar (*Lepisosteus oculatus*) sequences	23
	TAPBP-like sequences from selected species	24
Zebrafish haplotypes 19B, 19D and other published sequences*		25
Medaka Hd-rR, HN1, cab haplotype published sequences*		27
References		29

*Grey coloured sequences are previously published.

A.salmon Ia region Haplotype Ia_A; BACs 868O01 (EF441211) and 92I04 (EF427384.1) deduced amino acid sequences

BAC sequence reference: see Lukacs et al. [1, 2].

```
>SalmonTAPBPa_#A ABO13869.1 EF441211:64.923-69.673
MANISTIYKLSFLAFTYFIHVYGTSCPVLECWFVQEKPGRGGGFPAAMIQEKSLLYINTD
PESEETKSQQGPSADINHDRVYYVTDPAAILCSSSLHPPEGSVHKPQCEINPFMPQPSTV
QWVVPLTDSAHSPIYLQADWYSAALQGLDGQLGLSSVMRAPTATKEPTVVLSVSSRTPLV
RSRLGEPVVLDCGFWMEATSPLSGSGFAVEWRYQFRGDGRLVLAYDGKTDRFAETKEKRA
GLDFTALHETGNASLILQEAQVRHTGTYICTVYLPYLLAQVAVELEIVEPPSLSIFPSPL
PLSVPGQVVMVKVQCEASGFFPLSLDFHWELTGPDGKVRPLGQGSVTGHRQGPDNTYSQT
SRLELDSAKLDLGRGGEVTCVAVHPGGTRRASVTLNVIGINGPSIEDSMAMVAVALGLYG
LIKIVSWTFSSGSDDTNSQEKKVK
>SalmonULA*0102_#A ABO13870.1  EF441211: 79.632-94.364
MKCFILLLLSISLHAASAAMHSLRYVYTATSGMPDFPEFMTVGLVNGEPISYYDSIIRRE
TPRQDWMKEAVDPDYWNRNTQTSIGDEQTFKANIDVAKQRFNQTGGVHVYQNMYGCEWDD
EAGVTEGFDQYGYDGEDFLAFDLKTLKWIAPTPQSLITKLKWDNNMAQIQQDKHYLTQTC
IEWLKKYLDYGKSTLMRTVPPSVSLLQKTPSSPVTCHATGFYPSGVMVSWQKDGQDHHED
VEYGETLQNDDGTFQKSSHLTVTPEEWKNNKYQCVVQVTGVKEDFIKVLTESEIKTNWGN
TNIGFVPANTSDVGSNSSHNTVPKE
>SalmonUBA*0201_#A EF441211: 133.909-140.046 a1 domain and EF427384.1:1-
8.682 a2-TM domains
MKSCILLLFLGIVLLHTVSAATNTLQYFYTATSGIDNFPEFVTMGIVNGHQIDHYDSITK
RAIQKAEWISGAVDPDYWKTNTQIYAGTETVFVNNINVAKSRFNQTGVHVNQKMYGCEWD
DETGVTEGFDQDGYDGEDFLAFDLKTLTWIAPTPQAVITKLKWDSNTAQNEYRKNYLTQT
CIEWLKKYLDYGKSTLMRTVPPSVSLLQKTPSSPVTCHATGFYPSGVMVSWQKDGQDHHE
DVEHGETLQNDDGTFQKSSHLTVTPEEWKNNKYQCVVQVTGLQEDFIKVLTESEIKTNWN
DPNIVLIIGVVVALLLVVVAVVVGVVIWKKKSKKGFVPASTSDTDSDNSGRAAQMT
>SalmonPSMB8a_#A EF427384.1:15.837-18.682 ABQ59680.1
MALFDVSGYKSYSELRGQIIGTGVGHFIDRPNKQFSVPVGVDPSGFLKSCSREGGVSIDL
NHGTTTLAFTFRHGVIVAVDSRASAGSYIASKEANKVIEINPYLLGTMSGSAADCQYWER
LLAKECRLYKLRNKQRISVSAASKLLCNMMLGYRGMGLSMGSMIVGWDNKGPGLYYVDDN
ATRLSGRMFSTGCGSSYAYGVIDSGYREDMTVEEAYELGRRGITHATHRDAYSGGVVNLY
HMQEDGWIKVCKEDVSELIHRYRKGMF
>SalmonPSMB13a_#A EF427384.1:19.476-24.495 ABQ59681.1
MALSNVLEIPTSGFNFKNVARNVALEGLLEGGHTKTLKPMKTGTTIAGLVCKEGVVLGAD
TRATSGEVVADKMCAKIHYISPNIYCCGAGTAADTEKTTDLLSSNLTIFSMNSGRNPRVV
MAVNILQDMLFRYRGQIGASLILGGVDCTGNHLYTVGPYGSIDNVQYLAMGSGDLAALGI
LEDRFKPNMEMEEAKELVRDAIHSGIMSDLGSGNNIDICVITKQGVDYIRPYQESEYKDK
RRKRYKYGTGTTSILTEKIVPLELEVVQKTVQRMDTA
>SalmonPSMB12a_#A EF427384.1:24.652-29.374 ABQ59686.1
MERHLMDSQIKGVSTGTTILAVTFNGGVIIGSDSRASIGGSYVSSKTINKLIQVHDRIFC
CIAGSLADAQAVTKAAKFQISFHSIQMESPPLVKAAASVLKELCYNNKEELQAGFITAGW
DRKKGPQVYTVALGGMLLSQPFTIGGSGSTYIYGYADAKYKPDMSKEECLQFAKNALALA
MGRDNVSGGVAHLVVITEEGVEHVVIPGDKLPKFHDE
>SalmonPSMB9a_#A EF427384.1:32.328-36.287 ABQ59682.1
MLEESSEPGWLSEEVKTGTTIIAIEFDGGVVLGSDSRVSAGETVVNRVMNKLSLLHDKIY
CALSGSAADAQTIAEMVNYQLDVHSIEVGEDPQVRSAATLVKNISYKYKEELSAHLIVAG
WDKRGGGQVYVTLNGLLSRQPFAVGGSGSAYVYGFVDAEYRKAMSKEDCQQFVVNTLSLA
MSRDGSSGGVAYLVTIDEKGAEEKCILGNELPTFYDQ
>SalmonTAP2a_#A EF427384.1:37.057-41.156 ABQ59683.1
MTKMMLRTCALAMAVGLCIDITTLAFGASISKTGPRTFDAFGNVVRLWVVAGIRLVLLLG
LTLLTLGSIKPVFKRWLAVHCFLAPVYETGRRMLYGSSPERVYGSLGLGSPSLWLLCTAA
AAAAALFWETTFPDSNGESNGKQKTQKARVLFMRVLYFYRPDTLLLVGAFIFLSLAVLCE
MFIPFYTGKVIDILGTQYKWNNFLTAIILMGLYSLGSSFSAGCRGGLFMCAINSFTCRMK
VELFGALVKQEIGFFETMKTGDITSRLSTDTTLMGRAVALNVNVLLRTLIKTVGMLSLMM
SLSWKLTLLMLMETPITGLLQSVHDNYYQRLSKEVQDSMARANEAAGETVGGIRTVRSFK
TEQHEAGRYNDRLMDTHNLKTRRDTVRAVYLLLRRLTAVVMQVAMLYYGRLFIQRGQMST
GNLVSFILYQSDLADNIRTLIYIFGDMLNSVGAAGKVFEYLDREPQVSTKGTLQPETLTG
HVHFNNLSFSYPTRQERKVLQGFSLELRPGQLTALVGPSGGGKSTCVSLLERFYQPQQGE
```

ILLDGLPLQSYQHHYLHRKIAMVGQEPVLFSGSIKDNIAYGLADCSLERVQEAARRANAH
SFISQLEKGYDTDVGERGGQLSGGEKQRIAIARALIREPQVLILDEVTSALDTESEHMVQ
EALASCPSQTLLVIAHRLKTIERADQIILIDQGTVQEQGTHQELMDRKGSYYKLKERLFT
EDDAPH
>SalmonPSMB8Fa_#A pseudogene EF427384.1: 50.877-50.960
HKRNTGWIKVSQEDVGDLYHRFYNDKK*

A.salmon Ia region Haplotype Ia_#B; BAC 714P22 (EF210363) deduced amino acid sequences

Bac sequence reference: see Lukacs et al. [1, 2].

>SalmonUBA*0601_#B ABQ01995.1 EF210363:10.646-12.879
VHVNQWMYGCEWDDEAGVTEGFEQWGYDGEDFIAFDLKTKSWIAPTPQAVITKLKWDSDT
AQNEHRKNYYTQICIEWLKKYVDYGKSTLMRTVPPSVSLLQKTPSSPVTCHATGFYPSGV
MVSWQKDGQDHHEDVEYGETLQNDDGTFQKSSHLTVTPEEWKNNKYQCVVQVTGVKEDFI
KVLTESEIKTNWNDPNIVLIIVVVVALLLLVVAVVVGVVIWKKKSKKGFVPASTSDTDSD
NSGRAAQMT
>SalmonPSMB8a_#B ABQ01989.1 EF210363:19.903-22.781
MALFDVSGYKSYSELRGQIIGTGVGHFIDRPNKQFSVPVGVDPSGFLKSCSREGGVSIDL
NHGTTTLAFTFRHGVIVAVDSRASAGSYIASKEANKVIEINPYLLGTMSGSAADCQYWER
LLAKECRLYKLRNKQRISVSAASKLLCNMMLGYRGMGLSMGSMIVGWDNKGPGLYYVDDN
ATRLSGRMFSTGCGSSYAYGVIDSGYREDMTVEEAYELGRRGITHATHRDAYSGGVVNLY
HMQEDGWIKVCKEDVSELIHRYRKGMF
>SalmonPSMB13a_#B ABQ01990.1 EF210363:23.293-28.664
MALSNVLEIPTTGFNFENVARNVALEGLLEGGHTKTLKPMKTGTTIAGLVCKEGVVLGAD
TRATSGEVVADKMCAKIHYISPNIYCCGAGTAADTEKTTDLLSSNLTIFSMNSGRNPRVV
MAVNILQDMLFRYRGQIGASLILGGVDCTGNHLYTVGPYGSIDNVQYLAMGSGDLAALGI
LEDRFKPNMEMEEAKELVRDAIHSGIMSDLGSGNNIDICVITKQGVDYIRPYQESEYKDK
RRKRYKYGTGTTSILTEKIVPLELEVVQKTVQRMDTA
>SalmonPSMB12a_#B ABQ01993.1 EF210363:28.644-33.489
MERHLMDSQIKGVSTGTTILAVTFNGGVIIGSDSRASIGGSYVSSKTINKLIQVHDRIFC
CIAGSLADAQAVTKAAKFQISFHSIQMESPPLVKAAASVLKELCYNNKEELQAGFITAGW
DRKKGPQVYTVALGGMLLSQPFTIGGSGSTYIYGYADAKYKPDMSKEECLQFAKNALALA
MGRDNVSGGVAHLVVITEEGVEHVVIPGDKLPKFHDE
>SalmonPSMB9a_#B ABQ01991.1 EF210363:36.392-40.350
MLEESSEPGWLSEEVKTGTTIIAIEFDGGVVLGSDSRVSAGETVVNRVMNKLSLLHDKIY
CALSGSAADAQTIAEMVNYQLDVHSIEVGEDPQVRSAATLVKNISYKYKEELSAHLIVAG
WDKRGGGQVYVTLNGLLSRQPFAVGGSGSAYVYGFVDAEYRKAMSKEDCQQFVVNTLSLA
MSRDGSSGGVAYLVTIDEKGAEEKCILGNELPTFYDQ
>SalmonTAP2a_#B ABQ01992.1 EF210363:41.120-45.219
MTKMMLRTCALAMAVGLCIDITTLAFGASISKTGPRTFDAFGNVVRLWVVAGIRLVLLLG
LTLLTLGSIKPVFKRWLAVHCFLAPVYETGRRMLYGSSPERVYGSLGLGSPSLWLLCTAA
AAAAALFWETTFPDSNGESNGKQKTQKARVLFMRVLYFYRPDTLLLVGAFIFLSLAVLCE
MFIPFYTGKVIDILGTQYKWNNFLTAIILMGLYSLGSSFSAGCRGGLFMCAINSFTCRMK
VELFGALVKQEIGFFETIKTGDITSRLSTDTTLMGRAVALNVNVLLRTLIKTVGMLSLMM
SLSWKLTLLMLMETPITGLLQSVHDNYYQRLSKEVQDSMARANEAAGETVGGIRTVRSFK
TEQHEAGRYNDRLMDTHNLKTRRDTVRAVYLLLRRLTAVVMQVAMLYYGRLFIQRGQMST
GNLVSFILYQSDLADNIRTLIYIFGDMLNSVGAAGKVFEYLDREPQVSTKGTLQPETLTG
HVHFNNLSFSYPTRQERKVLQGFSLELRPGQLTALVGPSGGGKSTCVSLLERFYQPQQGE
ILLDGLPLQSYQHHYLHRKIAMVGQEPVLFSGSIKDNIAYGLADCSLERVQEAARRANAH
SFISQLEKGYDTDVGERGGQLSGGEKQRIAIARALIREPQVLILDEVTSALDTESEHMVQ
EALASCPSQTLLVIAHRLKTIERADQIILIDQGTVQEQGTHQELMDRKGSYYKLKERLFT
EDDAPH
>SalmonPSMB8Fa_#B EF210363:54.985-55.056 no predicted ORF
HKRNTGWIKVSQEDVGDLYHRFYNDKK*

A.salmon MHCIa Haplotype Ia_#C; Chr.27 genome NC_027326: 10.000.000-10.656.000 deduced amino acid sequences.

Atlantic salmon genome assembly: See Lien et al. [3].

>SalmonTAPBPa_#C NP_001117077.1 NC_027326:10.023.359-10.039.558
MANISTIYKLSFLAFTYFIHVYGTSCPVLECWFVQEKPGRGGGFPAAMIQEKSLLYINTD
PESEETKSQQGPSADINHDRVYYVTDPAAILCSSSLHPPEGSVHKPQCEINPFMPQPSTV
QWVVPLTDSAHSPIYLQADWYSAALQGLDGQLGLSSVMRAPTATKEPTVVLSVSSRTPLV
RSRLGEPVVLDCGFWMEATSPLSGSGFAVEWRYQFRGDGRLVLAYDGKTDRFAETKEKRA
GLDFTALHETGNASLILQEAQVRHTGTNICTVYLPYLLAQVAVELEIVEPPSLSIFPSPL
PLSVPGQVVKVQCEASGFFPLSLDFHWELTGPDGKVRPLGQGSVTGHRQGPDNTYSQTSR
LELDSAKLDLGRGGEVTCVAVHPGGTRRASVTLNVIGINGPSIEDSMAMVAVALGLYGLI
KIVSWTFSSGSDDTNSQEKKVK"
>SalmonULA_#C XP_014032820.1 NC_027326:10.037.566-10.054.430
MKCFILLLLSISLHAASAAMHSLRYVYTATSGMPDFPEFVTVGLVNGEPISYYDSIIRSE
TPRQDWMKEAVDPDYWNRNTQTSIGDEQTFKANIDVAKQRFNQTGGVHVYQNMYGCEWDD
EAGVTEGFDQYGYDGEDFLAFDLKTLKWIAPTPQSLITKLKWDNNMAQIQQDKHYLTQTS
IEWLKKYLDYGKSTLMRTVPPSVSLLQKTPSSPVTCHATGFYPSGVMVSWQKDGQDHHED
VEYGETLQNDDGTFQKSSHLTVTPEEWKNNKYQCVVQVTGVKEDFIKVLTESEIKTNWGN
TNIGFVPANTSDVGSNSSHNTAPKE"
>SalmonUBA*0301_#C XP_014032819.1 NC_027326:10.122.009-10.149.394
MKCFILLLLGIALHSSSAATHSLRYVYTATSGIPDFPEFVTVGLVNGEPISYYDSIIRR
ETPRQDWMAKTEGSDYWESQTQVSIGSEQTFKANIDVAKQRFNQTGGVHVNQKMYGCEWD
DETGVTEGFDQDGYDGEDFLAFDLKTLTWIAPTPQAVITKLKWDSNTAQNEYRKNYLTQT
CIEWLKKYLDYGKSTLMRTVPPSVSLLQKTPSSPVTCHATGFYPSGVMVSWQKDGQDHHE
DVEHGETLQNDDGTFQKSSHLTVTPEEWKNNKYQCVVQVTGLQEDFIKVLTESEIKTNWN
DPNIVLIIGVVVALLLVVVAVVVGVVIWKKKSKKGFVPASTSDTDSDNSGRAAQMT
>SalmonPSMB8a_#C XP_014032817.1 NC_027326:10.153.897-10.157.743
MALFDVSGYKSYSELRGQIIGTGVGHFIDRPNKQFSVPVGVDPSGFLKSCSREGGVSIDL
NHGTTTLAFTFRHGVIVAVDSRASAGSYIASKEANKVIEINPYLLGTMSGSAADCQYWER
LLAKECRLYKLRNKQRISVSAASKLLCNMMLGYRGMGLSMGSMIVGWDNKGPGLYYVDDN
ATRLSGRMFSTGCGSSYAYGVIDSGYREDMTVEEAYELGRRGITHATHRDAYSGGVVNLY
HMQEDGWIKVCKEDVSELIHRYRKGMF
>SalmonPSMB13a_#C NC_027326.1:10.158.350-10.163.366 incomplete sequence
MALSNVLEIPTSGFNFENVARNVALEGLLEGGHTKTLKPMKTGTTIAGLVCKEGVVLGAD
TRATSGEVVADKMCAKIHYISPNISCCGAGTAADTEKTTDLLSSNLTIFSMNSGRNPRVV
MAVRYRGQIGASLILGGVDCTGNHLYTVGPYGSIDNVQYLAMGMEEAKELVRDAIHSGIM
SDLGSGNNIDICVITKQGVDYIRPYQESEGTGTTSILTEKIVPLELEVVQKTVQRMDTA
>SalmonPSMB12a_#C XP_014032816.1 NC_027326:10.163.427-10.168.543
MERHLMDSQIKGVSTGTTILAVTFNGGVIIGSDSRASIGGSYVSSKTINKLIQVHDRIFC
CIAGSLADAQAVTKAAKFQISFHSIQMESPPLVKAAASVLKELCYNNKVMLQAGFITAGW
DRKKGPQVYTVALGGMLLSQPFTIGGSGSTYIYGYADAKYKPDMSKEECLQFAKNALALA
MGRDNVSGGVAHLVVITEEGVEHVVIPGDKLPKFHDE"
>SalmonPSMB9a_#C XP_014032548.1 NC_027326:10.171.104-10.175.148
MLEESSEPGWLSEEVKTGTTIIAIEFDGGVVLGSDSRVSAGETVVNRVMNKLSLLHDKIY
CALSGSAADAQTIAEMVNYQLDVHSIEVGEDPQVRSAATLVKNISYKYKEELSAHLIVAG
WDKRGGGQVYVTLNGLLSRQPFAVGGSGSAYVYGFVDAEYRKAMSKEDCQQFVVNTLSLA
MSRDGSSGGVAYLVTIDEKGAEEKCILGNELPTFYDQ"
>SalmonTAP2a_#C XP_014032814.1 NC_027326:10.176.036-10.180.873 and TSA
GBRB01034973.1
MMLRMCAFAMAVGLCIDITTFCAPCFGESISETGPITFGTFGNVVRLWVVAGIRLVLLLG
LTLLTLGSIKPVFKRWLAVHCFLAPVYETGRRMLYGSSPERVYGSLGLGSPSLWLLCTAA
AAAAALFWETTFPDSNGESNGKQKTQKARVLFMRVLYFYRPDTLLLVGAFIFLSLAVLCE
MFIPFYTGKVIDILGTQYKWNNFLTAIILMGLYSLGSSFSAGCRGGLFMCAINSFTCRMK
VELFGALVKQEIGFFETIKTGDITSRLSTDTTLMGRAVALNVNVLLRTLIKTVGMLSLMM
SLSWKLTLLMLMETPITGLLQSVHDNYYQRLSKEVQDSMARANEAAGETVGGIRTVRSFK
TEQHEAGRYNDRLMDTHNLKTRRDTVRAVYLLLRRLTAVVMQVAMLYYGRLFIQRGQMST
GNLVSFILYQSDLADNIRTLIYIFGDMLNSVGAAGKVFEYLDREPQVSTKGTLQPETLTG
HVHFNNLSFSYPTRQERKVLQGFSLELRPGQLTALVGPSGGGKSTCVSLLERFYQPQQGE
ILLDGLPLQSYQHHYLHRKIAMVGQEPVLFSGSIKDNIAYGLADCSLERVQEAARRANAH
SFISQLEKGYDTDVGERGGQLSGGEKQRIAIARALIREPQVLILDEVTSALDTESEHMVQ
EALASCPSQTLLVIAHRLKTIERADQIILIDQGTVQEQGTHQELMDRKGSYYKLKERLFT
EDDAPH
>SalmonPSMB8Faψ_#C No predicted ORF NC_027326:10189712-10189792

HKRNTGWIKVSQEDVGDLYHRFYNDKK
>SalmonPSMB10a_#C XP_014032774.1 NC_027326:10.580.777-10.585.553
MLHNSRPPQPQSAGFSFENTRRNAVLEGNLSELGYSSPKARKTGTTIAGIVFKDGVILGA
DTRATDDMVVADKNCMKIHYIAPNIYCCGAGVAADAEVTTQMMSSNVELHSLSTGRPPLV
VMVTRQLKQMLFRYQGHIGSSLIVGGVDVTGAHLYSVYPHGSYDKLPFLTMGSGAGAAIS
IFEDRYRPNMELEEAKKLVRDAIAAGIFCDLGSGSNVDLCVITQAGVQYLRSYDQPAQKG
KKEGQYKYKPGTTAVLTKTVTPLPLDVVDESIQLMDAQ"

A.salmon Ib region Haplotype Ib_#A; BAC 8I14 (EF427379) deduced amino acid sequences

BAC sequence reference:see Lukacs et al.[1, 2].

>SalmonTAPBPb_#A ABQ59655.1 EF427379:39.450-49.132
MTNISTILQSFLAFSYFMHVYGASCPVLECWFVQEKPGGGFHAAMSQEKSLLYINTDPDS
EETRSKQGLSTDHDRVYYVTDPAATLCSSSMHPPEGSVQKPQCEINPFMPQPSTVQWTVP
LTDSAHSPIYLQADWYTAALQGFDGQLRLSNVMRAPTATKEPKVLLSVSSRTFMIRSRLG
EPVVLDCGFWVDASSPLSGSGFAVEWRYQFRGDGRLVLAYDGKTDRFAETQEEGAGLDFT
ALHETGNASLILQEAQVRHSGTYICTVYLPYLLAQVAVELEIVEPPSLSIFPSPLPLSMP
GQVVTVQCEASGFYPLSLEFHWVLTGPGGRVSPLGQGSVTGHRQGPDSTYSQTSRVELDS
AKLDLGRGGEVTCVAVLRGGTRRASVTLNITGVSAPSIEDYMAMVAVALGIYGLIKVVSW
TFSSGSDVAYTQEKKVK
>SalmonUCA_#A EF427379:60.574-70.123
MKGCILMFLGIVYHREAFGVTHSLKYFYTASSKVPNFPEFVVVGMVDGVQIDHYDSNSQR
MVPKQDWMNKQTDAEYWERETGIAFDSQQVFKDDVNILKQRFNQSGGVHVLQYIYGCSWD
DETEQRDGFWAAGGFGQLGYNGEDFLVYDMNTLTWKALKQQADVMRDKWNRDISRLVFWK
TYFSQTCIECLKKQVVNGKSSLRTVPPSVSLLQKTPSSPVTCHATGFYPSGVMVFWQKDG
QEQHEDVEHGEILHNDDGTFQKSTHLRVTPEEWKNNKYQCVVQVTGIKEDFIKVLTESEI
QTNWDDPAPIIVPIIGGVVALLLVVVVVVVVVGVVIWKKKSKKGFVPASTSATDSDNSWK
GFQKT*
>SalmonPSMB8b_#A ABQ59648.1 EF427379:72.420-75.566
MALFDVSGYKSHAGLRGQILGTGVGHLVDRPNQEFAVPVGVDPSGFLKSCSREGGVSIDL
NHGTTTLAFTFRHGVIVAVDSRASAGSYIASKEANKVIEINPYLLGTMSGSAADCQYWER
LLAKECRLYKLRNKQRISVSAASKLLCNMMLGYRGMGLSMGSMIIGWDNKGPGLYYVDDN
ATRLSGRMFSTGCGSSYAYGVVDSGYREDMTVEEAYELGRRGITHATHRDAYSGGVVNLY
HMQEDGWIKVCKEDVSELIHRYRKGMF
>SalmonPSMB13b_#A ABQ59647.1 EF427379:76.573-80.426
MALTNVVETPASGFNFENVSRNVALEGLLEGGHTKAPKPMKTGTTIAGVVCKDGVVLGAD
TRATSGEVVADKMCAKIHYISPNMYCCGAGTAADTEKTTDMLSSNLTIFSMTSGRNPRVV
MAVNILQDMLFRYRGQIGASLILGGVDCTGNHLYTVGPYGSIDNVPYLAMGSGDLAALGI
LEDRFKPNMELEEAKELVRDAIHSGIMSDLGSGNNIDICVITKQGVDYIRPYQESEYKDK
RQRRYKYRPGTTSILTEKIVPLELEVVQETVQRMDTA
>SalmonPSMB12b_#A ABQ59652.1 EF427379:80.282-87.669
MERHFMDSQIKGVSTGTTILAVTFNGGVIIGSDSRASIGGSYVSSKTINKLIQVHDRIFC
CIAGSLADAQAVTKAAKFQISFHSIQMESPPLVKAAASVLKELCYNNKEELQAGFITAGW
DRKKGPQVYTVALGGMLLSQPFTIGGSGSTYIYGYADAKYKPDMSKEECLQFATNALALA
MGRDNVSGGVAHLVVITEEGVEHVVIPGDKLPKFHDE
>SalmonPSMB9b_#A ABQ59649.1 EF427379:88-882-93.220
MLEESSEPGWLSEEVKTGTTIIAIEFDGGVVLGSDSRVSAGETVVNRVMNKLSLLHDKIY
CALSGSAADAQTIAEMVNYQLDVHSIEVGEDPQVRSAATLVKNISYKYKEELSAHLIVAG
WDKRGGGQVYVTLNGLLSRQPFAVGGSGSAYVYGFVDAEYRKAMSKEDCQQFVVNTLSLA
MSRDGSSGGVAYLVTIDEKGAEEKCILGNELPTFYDQ
>SalmonTAP2b_#A ABQ59650.1 EF427379:95.267-99.916
MMRRTCVFTMAVGLCIDITTFCATGLGASISKTGPISFDVFGNLVRLWVEAGIRLVLLFG
LSLLTLGSIKPVLKRWLAVHCFLAPVYETGKLMLHGSSPESPYGSLGGPSLWLLCTAAAA
AAALFWEKTFPDSKEESNGKEKTQKARALFMRVLYFYRPDTLLLVGAFIFLALAVLSETF
IPFYTGKVIDILASQYKWNDFLTAIILMGLYSLGSSFSAGCRGGLFMCAINSFTCRMKVE
LFGALVKQEISFFETIKTGDITSRLSTDTTKMARALALNVNVLLRTLIKTVGMLSLMMSL
SWKLTLLMLMETPITGLLQGVYDNYYLRLSKEMQDSMARANEAAGETVAGIRTVRSFNTE
RSEAGRYDHRLMDTHNLKTRRDTVRAVYLLLKRLTALVMQVAMLYYGRLFIQRGQMSTGN
LVSFILYQSNLGANIRTLIYIFGDMLNSVGAAGKVFEYLDREPQVSTKGTLQPETLTGHV

HFHNLSFSYPTRQGRKVLQGFSLELRPGQLTALVGPSGGGKSTCVSLLERFYQPQQGEIL
LDGQPLHSYQHHYLHRKVAMVGQEPVLFSGSIKDNIAYGLADCSLERVQEAARRANAHSF
ISQLEKGYDTDVGERGGQMSGGEKQRIAIARALIREPQVLILDEVTSALDTESEHMVQEA
LASCPSQTLLVIAHRLKTIERADRIILIDRGSVLEQGTHQELMDRKGGYYKLRERLFTED
DTSH
>SalmonPSMB8Fb_#A EF427379:110.523-110.606
HMRETGWINVSQEDVADLYHHLYNEKK

A.salmon Ib region Haplotype Ib_#B; BAC 438J08 (FJ969490) deduced amino acid sequences

BAC sequence reference: see Lukacs et al. [1, 2].

>SalmonUDA_#B FJ969490:15.583-29.784 ACY30371.1
MKGFILMFMGTCHLFEAFGVTHSLKHFYTASSKVTNFPEFMVVGMVDGVQIDHYDSNIQR
MVPKQDWMNKQTEAEYWERETGIAFDSQQVFKDDVNILKQRFNQSGGVHVLQYIYGCSWD
DETEQRDGFGQLGYNGEDFLVYDMNTLTWKALKQQADVMRDKWNRDISRLVFWKTYFSQT
CIECLKKQVVNGKSTLRTAPPSVSLLQKTPSSPVTCHATGFYPSGVMVFWQKDGQEQHED
VEHGEILHNDDGTFQKSTHLRVTPEEWKNNKYQCVVQVTGIKEDFIKVLTESEIQTNWGD
PAPIIVPIIGGVVALLLVVVVVGVVIWKKKSKKGFVPASTNDTDSVYSGKDLLKT
>SalmonPSMB8b_#B FJ969490:79.890-83.013 ACY30372.1
MALFDVSGYKSHAGLRGQILGTGVGHLVDRPNQEFAVPVGVDPSGFLKSCSREGGVSIDL
NHGTTTLAFTFRHGVIVAVDSRASAGSYIASKEANKVIEINPYLLGTMSGSAADCQYWER
LLAKECRLYKLRNKQRISVSAASKLLCNMMLGYRGMGLSMGSMIIGWDNKGPGLYYVDDN
ATRLSGRMFSTGCGSSYAYGVVDSGYREDMTVEEAYELGRRGITHATHRDAYSGGVVNLY
HMQEDGWIKVCKEDVSELIHRYRKGMF
>SalmonPSMB13b_#B FJ969490:84.043-87.912 ACY30373.1
MALTNVVETPASGFNFENVSRNVALEGLLEGGHTKAPKPMKTGTTIAGVVCKDGVVLGAD
TRATSGEVVADKMCAKIHYISPNMYCCGAGTAADTEKTTDMLSSNLTIFSMTSGRNPRVV
MAVNILQDMLFRYRGQIGASLILGGVDCTGNHLYTVGPYGSIDNVPYLAMGSGDLAALGI
LEDRFKPNMELEEAKELVRDAIHSGIMSDLGSGNNIDICVITKQGVDYIRPYQESEYKDK
RQRRYKYRPGTTSILTEKIVPLELEVVQETVQRMDTA
>SalmonPSMB12b_#B FJ969490:87.748-95.135 ACY30374.1
MERHFMDSQIKGVSTGTTILAVTFNGGVIIGSDSRASIGGSYVSSKTINKLIQVHDRIFC
CIAGSLADAQAVTKAAKFQISFHSIQMESPPLVKAAASVLKELCYNNKEELQAGFITAGW
DRKKGPQVYTVALGGMLLSQPFTIGGSGSTYIYGYADAKYKPDMSKEECLQFATNALALA
MGRDNVSGGVAHLVVITEEGVEHVVIPGDKLPKFHDE
>SalmonPSMB9b_#B FJ969490:96.348-100.683 ACY30375.1
MLEESSEPGWLSEEVKTGTTIIAIEFDGGVVLGSDSRVSAGETVVNRVMNKLSLLHDKIY
CALSGSAADAQTIAEMVNYQLDVHSIEVGEDPQVRSAATLVKNISYKYKEELSAHLIVAG
WDKRGGGQVYVTLNGLLSRQPFAVGGSGSAYVYGFVDAEYRKAMSKEDCQQFVVNTLSLA
MSRDGSSGGVAYLVTIDEKGAEEKCILGNELPTFYDQ
>SalmonTAP2b_#B FJ969490:102.730-107.377 ACY30376.1
MMRRTCVFTMAVGLCIDITTFCATGLGASISKTGPISFDVFGNLVRLWVEAGIRLVLLFG
LSLLTLGSIKPVLKRWLAVHCFLAPVYETGKLMLHGSSPESPYGSLGGPSLWLLCTAAAA
AAALFWEKTFPDSKEESNGKEKTQKARALFMRVLYFYRPDTLLLVGAFIFLALAVLSETF
IPFYTGKVIDILASQYKWNDFLTAIILMGLYSLGSSFSAGCRGGLFMCAINSFTCRMKVE
LFGALVKQEISFFETIKTGDITSRLSTDTTKMARALALNVNVLLRTLIKTVGMLSLMMSL
SWKLTLLMLMETPITGLLQGVYDNYYLRLSKEMQDSMARANEAAGETVAGIRTVRSFNTE
RSEAGRYDHRLMDTHNLKTRRDTVRAVYLLLKRLTALVMQVAMLYYGRLFIQRGQMSTGN
LVSFILYQSNLGANIRTLIYIFGDMLNSVGAAGKVFEYLDREPQVSTKGTLQPETLTGHV
HFHNLSFSYPTRQGRKVLQGFSLELRPGQLTALVGPSGGGKSTCVSLLERFYQPQQGEIL
LDGQPLHSYQHHYLHRKVAMVGQEPVLFSGSIKDNIAYGLADCSLERVQEAARRANAHSF
ISQLEKGYDTDVGERGGQMSGGEKQRIAIARALIREPQVLILDEVTSALDTESEHMVQEA
LASCPSQTLLVIAHRLKTIERADRIILIDRGSVLEQGTHQELMDRKGGYYKLRERLFTED
DTSH
>SalmonPSMB8Fb_#B FJ969490:117.938-118.021
HMRETGWINVSQEDVADLYHHLYNEKK*

A.salmon Ib region haplotype Ib_#C; Chr.14 Sally genome NC_027313:50.800.000-59.500.000 deduced amino acid sequences

Atlantic salmon genome assembly: see Lien et al.[3].

A.salmon Haplotype Ib_#C; Chr.14 Region NC_027313:50.800.000-51.100.000

>SalmonTAPBPb_#C_50 XP_013996968.1 NC_027313:50.968.347-50.981.043
PAATLCSSSLHPPEGSVQKPQCEINPFMPQPSTVQWTVPLTDSAHSPIYLQADWYTAALQ
GFDGQLRLSNVMRAPTATKEPKVLLSVSSRTFMIRSRLGEPVVLDCGFWVDASSPLSGSG
FAVEWRYQFRGDGRLVLAYDGKTDRFAETQEEGAGLDFTALHETGNASLILQEAQVRHSG
TYICTVYLPYLLAQVAVELEI
>SalmonUDA1_#C_50 NC_027313.1:50.992.799 -50.986.338
VTHSLKYFYTASSGVPNFPEFMVVGMVDGVQIDHYDSNSQRMVPKQDWMNKQTEAEYWER
ETGIAFDSQQVFKDDVNILKQRFNQSGGVHIVQKTYGCEWDDETGQTGGFTQHGYDGEDF
LGYDMKTFTWIAPKQQAEITRLKWNHDQAGLAFWHNYLTQTCIEWLKKYVDYGKSTLRKV
PPSVSLLQKTPSSPVTCHATGFYPSGVMVFWQKDGQEQHEDVEHGETLPNDDGTFQKSAH
LTVTSEEWKINQYQCVVQVTGIKEDFIKVLTESEIQTNW
>SalmonUCA2_#C _51 NC_027313.1:51.002.977-51.037.583
MKGFILMFMGTCHLFEAFGVTHSLKYFYTASSGVPNFPEFMVVGMVDGVQIDHYDSNSQR
MVPKQDWMNKQTEAEYWERETGIAFDSQQVFKADVDILKQRFNQSGGVHVLQYIYGCSWD
DETEQRDGF*QLGYNGEDFLVYDMNTLTWKALKQQADVMRDKWNRDISRLVFWKTYFSQT
CIECLKKQVVNGKSTLRVPPSVSLLQKTPSSPVTCHATGFYPSGVMVFWQKDGQEQHEDV
ENGETLHNDDGTFQKRTHLKVTSEEWKNNKYQCVVQVTGIKEDFIKVLTESEIQTNW

A.salmon Haplotype Ib_#C continued; Chr.14 region NC_027313: 59.000.000-59.498.000.

>SalmonTAPBPb_#C XP_013997225.1 NC_027313:59.041.175-59.053.861
MTNISTILQSFLAFSYFMHVYGASCPVLECWFVQEKPGGGFHAAMSQEKSLLYINTDPDS
EETRSKQGLSTDHDRVYYVTDPAATLCSSSMHPPEGSVQKPQCEINPFMPQPSTVQWTVP
LTDSAHSPIYLQADWYTAALQGFDGQLRLSNVMRAPMATKEPKVLLSVSSRTFMIRSRLG
EPVVLDCGFWVDASSPLSGSGFAVEWRYQFRGDGRLVLAYDGKTDRFAEIQEEGAGLDFT
ALHETGNASLILQEAEVRHSGTYICTVYLPYLLAQVAVELEIVEPPSLSIFPSPLPLSMP
GQVVTVQCEASGFYPLSLEFHWVLTGPGGRVSPLGQGSVTGHRQGPDSTYSQTSRVELDS
AKLDLGRGGEVTCVAVHRGGTRRASVTLNITGVSAPSIEDYMAMVAVALGIYGLIKVVSW
TFSSGSDVAYTQEKKVK"
>SalmonUCA_#C NC_027313.1:59.088.400-59.091.609 XR_001320594.1
VTHSLKYFYTASSGVPNFPEFMVVGMVDGVQIDHYDSNSQRMVPKQDWMNKQTEAEYWER
ETGIAFDSQQVFKDDVNILKQRFNQSGGVHVLQYIYGCSWDDETEQRDGFWAAGGFGQLG
YNGEDFLVYDMNTLTWKALKQQADVMRDKWNRDISRLVFWKTYFSQTCIECLKKQVVNGK
STLRVPPSVSLLQKTPSSPVTCHATGFYPSGVMVFWQKDGQEQHEDVEHGEILHNDDGTF
QKSTHLRVTPEEWKNNKYQCVVQVTGIKEDFIKVLTESEIQTNW
>SalmonPSMB8b_#C NP_001117007.1 NC_027313:59.095.880-59.098.288
MALFDVSGYKSHAGLRGQILGTGVGHLVDRPNQEFAVPVGVDPSGFLKSCSREGGVSIDL
NHGTTTLAFTFRHGVIVAVDSRASAGSYIASKEANKVIEINPYLLGTMSGSAADCQYWER
LLAKECRLYKLRNKQRISVSAASKLLCNMMLGYRGMGLSMGSMIIGWDNKGPGLYYVDDN
ATRLSGRMFSTGCGSSYAYGVVDSGYREDMTVEEAYELGRRGITHATHRDAYSGGVVNLY
HMQEDGWIKVCKEDVSELIHRYRKGMF"
>SalmonPSMB13b_#C XP_013997221.1 NC_027313:59.099.410-59.103.327
MALTNVVETPASGFNFENVSRNVALEGLLEGGHTKAPKPMKTGTTIAGVVCKDGVVLGAD
TRATSGEVVADKMCAKIHYISPNMYCCGAGTAADTEKTTDMLSSNLTIFSMTSGRNPRVV
MAVNILQDMLFRYRGQIGASLILGGVDCTGNHLYTVGPYGSIDNVPYLAMGSGDLAALGI
LEDRFKPNMELEEAKELVRDAIHSGIMSDLGSGNNIDICVITKQGVDYIRPYQESEYKDK
RQRRYKYRPGTTSILTEKIVPLELEVVQETVQRMDTA"
>SalmonPSMB12b_#C XP_013997223.1 NC_027313:59.103.305-59.111.118
MDSQIKGVSTGTTILAVTFNGGVIIGSDSRASIGGSYVSSKTINKLIQVHDRIFCCIAGS
LADAQAVTKAAKFQISFHSIQMESPPLVKAAASVLKELCYNNKEELQAGFITAGWDRKKG
PQVYTVALGGMLLSQPFTIGGSGSTYIYGYADAKYKPDMSKEECLQFATNALALAMGRDN

VSGGVAHLVVITEEGVEHVVIPGDKLPKFHDE"
>SalmonPSMB9b_#C NP_001117186.1 NC_027313:59.112.113-59.116.449
MLEESSEPGWLSEEVKTGTTIIAIEFDGGVVLGSDSRVSAGETVVNRVMNKLSLLHDKIY
CALSGSAADAQTIAEMVNYQLDVHSIEVGEDPQVRSAATLVKNISYKYKEELSAHLIVAG
WDKRGGGQVYVTLNGLLSRQPFAVGGSGSAYVYGFVDAEYRKAMSKEDCQQFVVNTLSLA
MSRDGSSGGVAYLVTIDEKGAEEKCILGNELPTFYDQ"
>SalmonTAP2b_#C NP_001117161.1 NC_027313:59.118.496-59.123.144
MMRRTCVFTMAVGLCIDITTFCATGLGASISKTGPISFDVFGNLVRLWVEAGIRLVLLFG
LSLLTLGSIKPVLKRWLAVHCFLAPVYETGKLMLHGSSPESPYGSLGGPSLWLLCTAAAA
AAALFWEKTFPDSKEESNGKEKTQKARALFMRVLYFYRPDTLLLVGAFIFLALAVLSETF
IPFYTGKVIDILASQYKWNDFLTAIILMGLYSLGSSFSAGCRGGLFMCAINSFTCRMKVE
LFGALVKQEISFFETIKTGDITSRLSTDTTKMARALALNVNVLLRTLIKTVGMLSLMMSL
SWKLTLLMLMETPITGLLQGVYDNYYLRLSKEMQDSMARANEAAGETVAGIRTVRSFNTE
RSEAGRYDHRLMDTHNLKTRRDTVRAVYLLLKRLTALVMQVAMLYYGRLFIQRGQMSTGN
LVSFILYQSNLGANIRTLIYIFGDMLNSVGAAGKVFEYLDREPQVSTKGTLQPETLTGHV
HFHNLSFSYPTRQGRKVLQGFSLELRPGQLTALVGPSGGGKSTCVSLLERFYQPQQGEIL
LDGQPLHSYQHHYLHRKVAMVGQEPVLFSGSIKDNIAYGLADCSLERVQEAARRANAHSF
ISQLEKGYDTDVGERGGQMSGGEKQRIAIARALIREPQVLILDEVTSALDTESEHMVQEA
LASCPSQTLLVIAHRLKTIERADRIILIDRGSVLEQGTHQELMDRKGGYYKLRERLFTED
DTSH
>SalmonPSMB8Fb_#Cψ No predicted ORF NC_027313:59.133.717-59.133.797 pseudogene
HMRETGWINVSQEDVADLYHHLYNEKK
>SalmonPSMB8b_#Cψ XP_013997205.1 NC_027313:59.496.446-59.497.368 pseudogene
MNTFDFDLTWDVIKSLFIFKTALKETNKIIEINHYLLGTMSGSAADCQYWERLLAKECRL
YKLRNKQRISVSAASKLLCNMMLGYRGMGLSMGSMIVGWDNKVQGGRE
>SalmonUGA_#C XP_013997204.1 NC_027313:59.497.676-59.507.931
MKTRLISAMKIYFVLLSCIHGALSVIHSLRYFYTSSSGISDFPEFVDMGMVNDQVISHYD
SITKRKVPKQSWMGKVFDQQYWDSTTEDLRGAEKVFKNNLQTAQKRFNQTGGMHISQDMY
GCEWDDETGLTEGFHHIGYDGQDLLVFDLKRATWIASVPQALHSKMKWEGDPSSIESEKR
YLTQDCIVWLKKYLEYGKTTLQRTVPPSVSLLQKTPSSPVTCHATGFYPSGVMVFWQKDG
QDHHEDVENGETLHNDDGTFQKRTHLKVTSEEWKNNKYQCVVQVTGIKEDFIKVLTESEI
QTNRGVNTIGSAPIIGVVVALLVVVVVVVGLVMWRRKKSKKGFVPASTSDTDSENSGKGA
QKI
>SalmonPSMB10b_#C XP_013997188.1 NC_027313.1: 59.678.647-59.684.589
MLNNSRPYQPQSAGFSFENTRRNAVLEGNLSELGYSSPKARKTGTTIAGIVFKDGVILGA
DTRATDDMVVADKNCMKIHYIAPNICCGAGVAADAEVTTQMMSSNVELHSLSTGRPPLVV
TVTRQLKQMLFRYQGHIGSSLIVGGVDVTGAHLYSVYPHGSYDKLPFLTMGSGAGAAISI
FEDRYRPNMELEEAKKLVRDAIAAGIFCDLGSGSNVDLCVITQAGVQYLRSYDQPAQKGK
KEGQYKYKPGTTAVLTKTVTPLPLHVVDESIQLMDTQ

Other deduced Atlantic salmon amino acid sequences used in this study identified in the Atlantic salmon genome [3]

>SalmonPSMB7ps_ssa11 NC_027310.1:48.402.865-48.410.166 keep
TLSVCQTQLGGFSFENGKRLGLVEDGLVLGADTRATEGMIVADKNCSKIHYICPSSCCGA
GTAADTEMTTQIISSNLELHSLSTGRLPRVATANRMLKQMLFRYTTH
>SalmonCANXa calnexin ssa05 NC_027304.1:27.384.003-27.403.278
XP_014054814.1
MELNVRCVVLLAVALCSTLLLTTVAAHQEEEEPIMELAEDMGVEDELEDLGLGEELLDGE
VEPEDADTPPGPPPAPKVTYKAPEPMGEHFFAESFDRGTLDSWVLSKAKKEDIDEDIAKY
DGKWEVEDMKDSKLPGDKGLVLKSRAKHHAISAQLLRPFIFDTKPLIIQYEVNFQQGIDC
GGAYVKLLSQTPDLNLDEFVDKTPYTIMFGPDKCGEDYKLHFIFRHKNPKTGEYEEKHAK
KPDADLRTYYTDKKTHLYTLVVNPDNSFEVLVDQTVVNSGNLLTDMTPPINPAAEIEDPD
DHKPEDWDERPKIQDPDAVKPEDWDEDAPKQIPDEDAVKPDGWLDDQPEYTSDPDAVKPE
DWDEDMDGEWEAPQIPNALCETAPGCGAWQRPMIDNPNYKGKWKAPMIDNPNYQGVWKPR
KIANPDFFEDLHPFRMTPFNAVGLELWSMSSDIFFDNFFITNERHTAERWANDGWGLKKA
AEGAAEPGLVNQMMTAADERPWLWVVYVLTVAVPLILIVVFFCTGKKAAAPAAADYKKTD
EPQPDVKEEEEKAEEDQVKEEKSQPAAAEKKSDAEDSPAEKEEEEEEEEEEEEEEEEEEE

EAAVNEEEEAATDQVRDKTTTR
>SalmonCANXb ssa09 NC_027308.1:57.626.097-57.639.090 XP_014067456.1
MELKVRYVLLLAVGLWSTLLLTTVTAHQDEEDEPIVEMGGDMDVEDDMEELDHGEELLDG
EVEADMPPGPPSVPKVTYKVPEPTGEHFFAESFDMGTLDSWVLSKAKKEDIDEDIAKYDG
KWEVEDMKDGKLPGDKGLVLKSRAKHHAISAQLLRPFIFDTKPLIVQYEVNFQQGIDCGG
AYVKLLSQTPDLNLDEFVDKTPYTIMFGPDKCGEDYKLHFIFRHKNPKTGEYEEKHAKKP
DADLRTYYTDKKTHLYTLVVNPDNSFEVLVDQAVVNSGNLLTDMTPPINPAAEIEDPDDH
KPEDWDERPKIQDPDAVKPEDWDEDAPKQIPDEDAVKPDGWLDDESEYTSDPDAVKPEDW
DEDMDGEWEAPQVPNALCETAPGCGAWQRPMIDNPSYKGKWKAPMIDNPNYQGVWKPRKI
ANPAFFEDLHPFRMTPFNAVGLELWSMSSDIFFDNFFITNERHTADRWANDGWGLKKAAE
GALEPGLVNQMMTAADERPWLWVVYVLTVAVPLVLIIVFCCTGKKTAAADYKKTDEPQPD
VKEEEVVEKAEADQVKEEKSQPAAEKNSDAEDSPAEKEEVNEEEEDEEEEEDEVTEEVRE
Q
>SalmonB2m1 XP_014044855.1 NW_012376632.1:2.680-3.763 3,8 kb unplaced
MKSILSIVVLVLIYSAVESKESPPKVQVYSRNPGNFGDKNTLICHVSGFHPPDISIQLLK
NGVEIPDAKQTDLAFEQGWQFHLTKSVGFTPDSGEEYTCRVRHLKNLKTYTWEPDM
>Salmonb2m2 NC_027306.1:57788614-57788364
YSRNPGEHGKDNTLICHVSGFHPPDISIQLLKNGVEIPDAKQTDLAFEQGWQFHLTKSVG
FTPDSGEEYTCRVRHLKNLKTYTW
>Salmonb2m3 NP_001117171.1 NC_027306.1: 57.826.485-57.828.480 ssa07
MKSILSIVVLVLIYSAVESKESPPKVQVYSRNPGNFGDKNTLICHVSGFHPPDISIQLLK
NGVEIPDAKQTDLAFEQGWQFHLTKSVGFTPDSGEEYTCRVRHLKNLKTYTWEADM
>Salmonb2m4 XP_014064343.1 NC_027306.1: 58.302.043-58.303.127 ssa07
MKSILSIVVLVLIYSAVESKESPPKVQVYSRNPGNFGDKNTLICHVSGFHPPDISIQLLK
NGVEIPDAKQTDLAFEQGWQFHLTKSVGFTPDSGEEYTCRVRHLKNLKTYTW
>Salmonb2m5 NW_012366138.1: 15-1.099 XP_014044161.1 unplaced
MKSILSIVVLVLIYSAVESKESPPKVQVYSRNPGNFGDKNTLICHVSGFHPPDISIQLLK
NGVEIPDAKQTDLAFEQGWQFHLTKSVGFTPDSGEEYTCRVRHLKNLKTYTW
>Salmonb2m6 NW_012360375.1:3.156-4.240 XP_014043232.1 unplaced
MKSILSIVVLVLIYSAVESKESPPKVQVYSRNPGNFGDKNTLICHVSGFHPPDISIQLLK
NGVEIPDAKQTDLAFEQGWQFHLTKSVGFTPDSGEEYTCRVRHLKNLKTYTWEPDM
>Salmonb2m7 NW_012349147.1: 7.171-7.154 XP_014039773.1 unplaced
MKTVLSVIAFCVFLGFINAKESPPKVQVYSRNPGNFGDKNTLICHVSGFHPPDISIQLLK
NGVEIPDAKQTDLAFEQGWQFHLTKSVGFTPARGEEYTCRVRHLKNLKTYTWESNM
>Salmonb2m8 NW_012349147.1:10.766-11.950 XP_014039774.1 unplaced
MKTVLSVIAFCVFLGFINAKESPPKVQVYSRNPGNFGDKNTLICHVSGFHPPDISIQLLK
NGVEIPDAKQTDLAFEQGWQFHLTKSVGFTPDSGEEYTCRVRHLKNLKTYTWEADM
>Salmonb2m9 NW_012394841.1:962-1.615 XP_014045653.1 unplaced
MKTVLSVVAFCVVLVTINAKESPPKVQVYSRNPGEHGKDNTLICHVSGFHPPDISIQLLK
NGVEIPDAKQTDLAFEQGWQFHLTKSVGFTPDSGEEYTCRVRHLKNLKTYTW
>Salmonb2m10 NW_012394112.1: 494-1.164 XP_014045626.1 unplaced
MKTVLSVVAFCVVLVTINAKESPPKVQVYSRNPGEHGKDNTLICHVSGFHPPDISIQLLK
NGVEIPDAKQTDLAFEQGWQFHLTKSVGFTPDSGEEYTCRVRHLKNLKTYTWGELLV
>Salmonb2m11 NW_012347820.1:16.383-18.779 NP_001232842.1
MKTVLSVIAFCVFLGFINAKESPPKVQVYSRNPGEHGKDNTLICHVSGFHPPDISIQLLK
NGVEIPDAKQTDLAFEQGWQFHLTKSVGFTPDSGEEYTCRVRHLKNLKTYTWESNM
>Salmonb2m12 NC_027316.1: 57.631.152-57.632.033 XP_014009395.1 ssa17
MKTVLSVIAFCVFLGFINAKESPPKVQVYSRNPGEHGKDNTLICHVSGFHPPDISIQLLK
NGVEIPDAKQTDLAFEQGWQFHLTKSVGFTPDSGEEYTCRVRHLKNL
>Salmonb2m13 NW_012571237.1:861-1.007 Pseudogene
KQTDLAFEQGWQFHLTKSVGFTPDSGEEYTCRVRHMKNLKTYTWGELLV
>SalmonCALR1a ssa14 NC_027313.1:15.482.204-15.485.137 XP_013995146.1
MLVSVLLMIALASAKPSVYFREQFEDDAWNTRWVESSHRSDYGKFVLTAGKFYGDAEKDK
GLQTSQDAHFYSSSARFEPFSNQGKTLVIQFTVKHEQNIDCGGGYIKLFPADLDQADMHG
DSNYNIMFGPDICGPATKKIHVIINYKGKNHLIRKDIRCKDDEYTHLYTLILNPDNTYEV
KIDNKKVESGSLEEDWDILPPKKVKDPEAVKPDHWDERERMEDPDDKKPEDWDRPENIAD
PDAKQPEDWDDEMDGEWEPPMVSNPDYKGEWKPRTIDNPDYKGKWLHPEIDNPDYSADSE
IYRFDSIGVIGLDLWQVKSGTIFDNFLITDDATLAEEVGNETWGQTKDPEKKMKESQEEK
ERKKLEAEEMARKEETKDEPGEEEEEEEEEELEHEEEEDEEEGETGAQEEEEESDSIKDE
L
>SalmonCALR1b ssa03 NC_027302.1:16.022.537-16.025.752 XP_014045728.1

MRGSMLFSALIALASAEPSLYFKEQFEDGDAWTTRWVESSHRSDYGKFVLTPGKFYGDPE
KDKGLQTSQDARFYSSSARFEPFSNQGKTLVIQFTVKHEQNIDCGGGYIKLFPADLDQAD
MHGDSKYNIMFGPDICGPGTKKVHVIINYKGKNHLISKDVRCKDDEYTHLYTLILNPDNT
YEVKIDNKKVESGSLEEDWDILPPKKVKDPEAVKPDDWDERERVEDPDDKKPEDWDRPEN
IADPDAKKPEDWDNEMDGEWEPPMVSNPDYKGEWKPRKIDNPDYKGKWVHPEIDNPEYSA
DSEIYRFDSIGVIGLDLWQVKSGTIFDNFLITDDATLAEEVGNETWGQTKDPEKKMKVSQ
EEQERKKLEAEEMGRKEQTKDEPEEEEEEEEEKEEELEDREEEEEGTGAEEEEEEETDSIK
DEL
>SalmonCALR1.2 ssa23 : NC_027322.1:26.361.479-26.364.218 XP_014024943.1
MTTMLILLMTVLVASIFGESSVYFREEFEDGDAWKSRWVESKHRSDYGKFVHTAGKFYGD
VERSKGLQTSQDARFYSASSRFESISNKDQTLVIQFTVKHEQNIDCGGGYIKLFPADLNQ
EEMHGDSTYNIMFGPDICGPGTKKVHVIFNYKGKNHLINKDIRCKDDEYTHLYTLIVNPD
NTYEVKIDNKKVESGSLEEDWDFLPLKKIKDPDAEKPDDWDEKENIDDPEDKKPEDWDVA
ENIPDPDAKKPDDWDDEMDGEWEPPMVTNPDYKGEWKPKQIDNPAYKGKWVHPEIDNPEY
TADPEIYQYASIGVIGLDLWQVKSGTIFDNFLITNDPKLAEEVGNETWGATKDPEKKMKD
RLEEEERKKREAEEVNKKEEDEDEDEDKEERDEEEDYDDEEEEEEEEETDSKLKDEL
>SalmonCALRL1 ssa14 NC_027313.1:41.509.871-41.518.596 XP_013996614.1
MQVLGSFAIILSIFSVHSTVYFQEQFLDGDAWKTRWLDSKHKADYGEWKLTAGNFYGDAE
KDKGLQTSQDARFYAASARFEPFSNEGKTLVIQFTVKHEQKIDCGGGYVKVFPSTLDQAD
MHGDSQYYIMFGPDICGYSTKKVHVIFNYKGKNHLIKKEVKCKDDELTHLYTLILNPNQT
YEVKIDNEKVESGTLEEDWDFLPAKTIKDPEAKKPDDWDDRPKMDDAEDAKPEDWDVAEN
IPDPDAKKPDDWDEDMDGEWEPPVITNPEYKGEWKPKQIDNPNYKGAWIHPEIDNPEYAA
DSTIYKFDDISVLGLDLWQVKSGTIFDNFLVSDDVKEAEKFGAETWGVTKEPEKKMKQEE
DDKKRKEEDEKNKEQATEAEEEGEEEEGEDEGEEEETPEEGTEEEEAPGKDEL
>SalmonCALRL2a ssa10 NC_027309.1:16.605.321-16.609.148 XP_014071687.1
MRVAVAILAVFASVAVTIDATVYFKEQFQ
DGDAWKSRWLVSEHKSDYGEWKLTAGKFYGDAEADKGLQTSQDARFYALSSRFEPFSNEG
KSLVVQFTVKHEQKIDCGGGYVKIFPADLDQAAMHGDSQYYIMFGPDICGYSTKKVHVIF
NYKGKNHLIKKEIKCKDDELTHLYTLILNPDQTYEVKINNEKVESGTLEDDWDILPPKTV
KDPEAKKPEDWDDRAKIDDPTDTKPEDWEKPENIPDPDAKIPDDWDVDMDGEWEPPMIPN
PEYQGEWKAKQIDNPEYKGAWVHPEIDNPEYTADASIYKFDNIGVLGLDLWQVKSGTIFD
NFLIGDDIKEAEEFGNETWGATKDPEKKMKDAQEEEERKAREEEEKSKDTADDEGDEDED
EPEEEDDDSPTEEEEGEDPKKDKDEL
>SalmonCALRL2b ssa16 NC_027315.1:40.259.148-40.267.862 XP_014004651.1
MRVAVAIFSVFASVAVTIDATVYFKEQFQDGDAWKSRWLVSKHKTDYGEWKLTAGKFYGD
AEADKGLQTSQDARFYAMSSRFEPFSNEGKPLVVQFTVKHEQKIDCGGGYVKIFPANLDQ
AAMHGDSQYYIMFGPDICGYSTKKVHVIFNYKGKNHLIKKEIKCKDDELTHLYTLILNPD
QTYEVKINNEKVESGTLEDDWDILPAKTIKDPEAKKPEDWDDRPKIDDPTDTKPEGWEKP
ENIPDPDAKKPDDWDVDMDGEWEPPVIPNPEYQGEWKPKQIDNPDYKGTWVHPEIDNPEY
TADTSIYKFDNIGVLGLDLWQVKSGTIFDNFLIGDDVKEAEEFGNETWGTTKEPEKKMKD
AQEEEERKAREEEEKSKKDTADDEGDEDEEEDESKEEEEDSPTEEGEEEIPMKDKDEL
>SalmonERp57a (PDIA3a) ssa23 NC_027322.1:41.701.278-41.708.848
XP_014025621.1
MLKLFFFVVLAGAALASDVIEFTDDDFDSKIGDHGMILVEFFAPWCGHCKKLAPEYEVAA
TRLKGIVGLAKVDCTVHNNVCQKYGVSGYPTLKIFRDGEDAGPYDGPRTADGIVSHLKKQ
AGPASVELKTEADFTKYVGDRDASVVGFFADDGSPAKAEFLKSASALRESFRFAHTNSEE
LLQKHSVEGEGIILFRPSRLNNKFEEGSVKFSEDTFTNAKIKQFIQDNIFGMCPHMTDDN
KDQMKGKDLLVAYYDVDYEKNPKGSNYWRNRVMKVAKGFLDQGNKLNFAVASKNSFSQDI
AEMGLDASSGELPVVGIRTAKGDKYVMTEEFSRDGKALERFLQDYFDGKLKRYLKSEPIP
ENNDGPVKTVVAENFDAIVNEEDKDVLIEFYAPWCGHCKSLEPKWKELGEKLSSDPNIVI
AKMDATANDVPSQYEVRGFPTIFFAPAGQKMSPKKYEGGREVSDFISYLKKEATNPLVAQ
EEETSKKKKKNEL
>SalmonERp57b (PDIA3b)ssa10 NC_027309.1:65.889.946-65.898.140
NP_001161991.1
MLKLFFFIVLAGAARASDVIEFSDDDFDSKIGDHGMILVEFFAPWCGHCKRLAPEFEVAA
TRLKGIVALAKVDCTVQNNVCQKYGVSGYPTLKIFKDGEDAGAYDGPRTADGIVSHLKKQ
AGPSSIELKTEADFTKYVGDRDASVVGFFADGGSPAQAEFLKSASALRESFRFAHTNSGE
LLQKNGVEGEGIILFRPARLSNKFEESVIKFSEDKFTNAMIKKFIQDNIFGMCPHMTDDN
KDQMKDKDLLVAYYDVDYEKNPKGSNYWRNRVMKVAKSFLDQGKTLNFAVASKNSFSHDI
SEMGLDASSGELPVVGIRTAKGDKYVMAEEFSRDGKALERFLQDYFDGKLKRYLKSEPSP
ENNDGPVKTVVAENFDAIVNNEEKDVLIEFYAPWCGHCKSLEPKWKELGEKLSSDPNIVI

AKMDATANDVPSQYEVRGFPTIFFAPAGQKMSPKKYEGAREVSDFISYLKREATNPLVAQ
EEETSKKNIQIEL
>SalmonERp57L1 ssa02 NC_027301.1:21.616.513-21.621.161 XP_014016726.1
MGTLGPFRMFLLFALAQQNVFVAASDVLELGDSDFHYTVAEYETVLVEFFAPWCGHCQQL
APEYETAATKLKGTVSLAKVDCTVNSETCGRFGVNGYPTLKIFRNGEDFAAYDGPRSADG
IVSYMKKQAGPSSVPLHNGRDLDAFVNNFDASVVGFFSGVDSSQMAEFLKASSAMRDSHR
FAHTTDLSLGLKHGVESDTVVLFRPPRLNSKFEDSLVKSDEAVSTASLRQFIRDNVFGLC
PHLTAENRENMRGRDLLVAYYDVDYLRNIKGTNYWRNRVMKVATQFQSRGLSYAVANRAE
FQEELEEEFGLGPSDGGELPLITIRNREGHKYSMQEEFTRDGKSLERFLEDYFAGKLKRQ
VKSEAASENNDGPVKVVVADNFEEIVNNPSKDVLVEFYAPWCGHCKSLEPKYTELGEQLS
ADTHIVIAKMDATANDVPPTYDVQGFPTIFFVPAGQKDQPRKYEGGREVNDFLNYLKEEA
THPLVLGTAREDL
>SalmonERp57L2a ssa14 NC_027313.1:61.089.980-61.110.809 XP_013997083.1
MASFLSLIPPFLLSVLIFSGAAVARGDVLELGDADFDYLAAEHETMLVKFYAPWCGHCKK
LAPDFETAATRLKGTVPLAKVDCTANPDTCGRFGVTGYPTLKIFRNGEDASSYDGPRSAD
GIVHFMKKQAGPNSVTLRREADLEAFVNHFDASVVGFFSGPDSGQLAEFLKAASVMREHF
RFAHTIDMTLGLKHGVDTERVLLFRPPRLSSKFEESVLHFTETITTHTLRRFIRDNIFGM
CPHLTNENRDKLKGQDLLTAYYDLDYLQNPKGSNYWRNRVMKVGSQFASQGLSFAVANRR
DFVDELEEEFGLGASDGGDLPFVTIRTRQGFKYTMREEFTRDGKSLERFLVDYFAGRLKR
YIKSEPIPEKNKGPVKVVVAESFEEIVNDPEKDVLIEFYAPWCGHCKSLEPKYKELAEQL
YSDPNIVIAKMDATANDVPQGFDVQGFPTIYFAQASKKDQPKRYEGAHEVKDFIKYLKRE
ASHVPVVSGVREDL
>SalmonERp57L2b ssa27 NC_027326.1:12.040.425-12.049.378 XP_014032674.1
MASFLSLIPAFTLSVVIFCGAVVARGDVLELGDADFDYLAAEHETMLVKFYAPWCGHCKK
LAPDFETAASRLKGTVPLAKVDCTASPDTCGRFGVTGYPTLKIFRNGEDSSSYDGPRSAD
GIVHYMKKQAGPNSVTLRSEADVEAFVNHFDASVVGFFSGPDTAQLAEFLKAAGAMRDHF
RFAHTINMTLGLKHGVDTESVLLFRPPRLSGKFEESVLRFTETITTHTLRRFIRDNIFGM
CPHLTNENRDKLKGQDLLTAYYDLDYLRNPKGSNYWRNRVMKVGYQFASQGLSFAVANRR
DFVDELEEEFGLGASDGGDLPFVTIRTRQGFKYTMREEFTRDGKSLERFLEDYFAGRLKR
YIKSEPIPEKNKGPVKVVVAESFEEIVNDPEKDVLIEFYAPWCGHCKSLEPKYKELAEQL
YSDPNIVIAKMDATANDVPQGFDVQGFPTIYFARADKKDQPKRYEGAREVKDFIKYLKRE
ASHIPVVSGVREDL
>SalmonPDIA1 ssa02 NC_027301.1:45.673.995-45.687.434 XP_014032240.1
MFKFFLLCTLAVASRADIGEEDDVLVLKKSNFEEALKAHPNILVEFYAPWCGHCKALVPE
YAKAASMLKAEGSEIRLAKVDATEEADLAQEYGVRGYPTIKFFKGGDKESPKEYSAGRQA
DDIVNWLKKRTGPAATTLGEVAQAESMIAENEVAVIGFFKDAESEGAKAFLKAAEAVDDV
PFGITSNDAVFSKFEVSKDGVVLFKKFDEGRNTFDGELSKADLLAFIKANQLPLVIEFTE
QTAPKIFGGEIKSHILMFVPKAASDFNDKMAEFKKASEGFKGKILFIFIDSEVDDNQRIL
EFFGLKKEECPAIRLITLEDEMTKYRPESEAITADNIIAFCTLFTEGKLKPHLMSQDIPE
DWDKNPVRVLVGKNFEEVVFDPKKNVFVEFYAPWCGHCKQLDPIWTKLGEKYQDSADIVV
AKMDSTANEIETVKVHSFPTLKFFPAGDEHKVVDYNGERTLEGFTKFLESGGKDGGAPAG
EEGEEDEGIDDMEDLDEQDSDSDGDGGDHDEL
>SalmonPDIA2.L1 XP_014038534.1 NW_012347234.1:27.515-33.804
MRFCVVLAAVVLVLRVSWTQTAEDTSPEQTDAAEQETDLEKKEKTTEIEEEKNVMVLHIN
NFQRALSENKFLLVEFYAPWCGHCRQLEPVYAEAARVLKGEREEERGEEEGFGLAKVDAV
EENQLAEEFDVGSFPTIKLFTDGDRNNPVDFTGKRTVQGIVQWMKRRSGPVAVALETTDA
AAEHINLHNVTVLGFFTSLESEEAKVFYSVAMEMVDMEFGVTTSPEVFQKYEIENNRVVL
FKKFDEGRVDLSVSEEVKVGEEELTVFIRTNSLELVIEFNEQNADKIFGSKIHSHSLLFI
NSTVQEQKNLLPEYRTAAKDFKGKVLFIIIDVTGPVSHVLKYFGLSEGDAPAVRIINTDT
TKKFALIGQITAATLQTFCQGVLDGNVKSHLLSEEVPEDWDKGPVKVLVGKNFEAVALEN
NKNVFVEFYAPWCGHCKELAPVWEKLAEKYADRDDIIIAKMDATTNEVEGVSVSGFPTLR
YYPAGEDSKVVEYSGTRDLETFAMFLDNGGQLPKAEEEEDDGDEEEVKDDEEDVKDDEEA
VKDDEEDDEEEVTDESSPPPANETSKDEL
>SalmonPDIA2.L2 XP_014042341.1 NW_012355960.1:107-7.487
SLESEEAKVFYSVAMEMVDMEFGVTTSPEVFQKYEIENNRVVLFKKNADKIFGSKIHSHS
LLFINSTVQEQKNLLPEYRTAAKDFKGKVLFIIIDVTGPVSHVLKYFGLSEGDAPAVRII
NTDTTKKFALIGQITAATLQTFCQGVLDGNVKSHLLSEEVPEDWDKGPVKVLVGKNFEAV
ALENNKNVFVEFYAPWCGHCKELAPVWEKLAEKYADRDDIIIAKMDATTNEVEGVSVSGF
PTLRYYPAGEDSKVVEYSGTRDLETFAMFLDNGGQLPKAEEEEDDGDEEEVKDDEEDVKD
DEEDDEEEVKDDEEDVKDDEEAVKDDEEDDEEEVTDESSPPPANETSKDEL
>SalmonTAPBPR ssa02 NC_027301.1:10.225.492-10.231.249 NP_001133983.1

MLEILLFGYLITCVSGQGGADVVLSCSLVEEGSGMGGMGGGALFSRTPAMLVLRDLAVTP
DLSPDTLTPFNPPAVPDPDNIILEAKVESPEIPEADLLLHADCNEQEVTCEISRYFPRNA
KEGSTEPAYFIGSLQIEGGGLSLTLILQTLPLDQSDRPALMQSKLELPLSQSGTLLTEVV
FLVFSRSESHSAPLGGVALLDCGFRQQAPPPGWELGLEWRLQHRGSGRKVLEIRAGQTET
EEGPAVHVEREGSSVDAALLVGQGNASLTLARLKVSDEGTYICTVSTGLYQAQQVIQLHV
TQPPRVSLSEEKLVFRDELPQKLSCHCKNYYPLDVQMEWISVSSTDSEPSVLSDQVSLSS
HRQHSDRTMSISSHLTLHPSTFPPGTTVTCRVTHPALDTPLSLSLTVETPEPDSYWMVLG
FLVITVLFFYQVMK
>SalmonTAPBPL1a ssa09 NC_027308.1:102.783.830-102.789.947 XP_014069540.1
MSLNVNILLCLFLCGEVPGIQSFEQVPWLPCQLVDESVTFNDEGHAETEYQHRDAGLQFG
HPGDSALNPNTITFLVTGSKVDMRKYIEGVVEHQLQCEIRRYSTESPQMRWPGLGAQEHD
IWFTCTIRHTDGVFIITSFLRHTPATPTPGQAHYLNWAAIADRAALTTSTVMLVLTRSPS
VRVGLVKQQSLHCQFDVDHKAADLTVEWRFQRRGERTTLFSHSSRSGQTEGSGVPLNAIG
RGDASLTLPLTKQSSEGTYVCHVSVPPLFGSHDITLQIMESPRVSLNVDSTISLVDRGEQ
KVVCEAEGYYPLDVEMEWFREPSGGGLLPEKLDTVLYSSHRHHQDGTYSLSAFFLLHASL
HDSGSKYFCRVSHSSQRMPIRKSFTLNVTEYDSWNAALWFFSGFGFILVMVATLFVMLPR
LSSARKANQRKPY
>SalmonTAPBPL1b ssa20 NC_027319.1:46.797.073-46.803.184 XP_014017660.1
MSLNVNIVICLFLCAEVPGIQSLEQVPWLPCMLVDERVKFNDEGHAETQYQHRNAGLQFG
HPGDSALNPNAITFLVTGSKVDMRKYIEGAVVEHQLQCEIRRYSTEGIQVRWPGLGAQDH
DIWFTCTLRHTDGLFVITSFLRHTPATPTPGQADYRNWAAIADREMLTTSTVMLVFTRTP
SVWVGLMKQSSLHCQFDVDHKAADLTVEWRLQRRSERTTLFSHSSRSGQREGGGVELKGI
GRGDASLTLPLTKQSSEGTYVCLVSVPPLFGSHEIALHIMEPPRVSLNVDSVISLVDTER
QKMVCEAVGYYPLDVEMEWFREPSGGGGGGLLPEKLDTVLYSSHRHHQDGTYSLSAFFLLH
ASLHDSGSKYFCRVSHSSLRMPIRKSFTLIVTEVDSWNFLWLFFGCGFILVMVATLCVML
PRLSSARKANKRKPY
>SalmonTAPBPL2 ssa07 NC_027306.1:12.926.171-12.930.490 XP_014062182.1
MKTFTVLLCLIPYAGVLGFLQVQWLRCRLKDEYVWTNDEGHIETNNSYRDAVLQFENSGN
SALLSESITFLVAASKVDMRKFVEGPVDQLQCDIHRYSKGMSRVRWPSLGGTGHDIWFTC
TLRHTAGLFNITSFLRVTPATTMSAHQPDFLSWLTIGVKEQISASAVMVLMTRSPSVRVG
LQEERTLHCQFAVDHKVPHLTVEWHLQRHGARRTKLFSYSSSSGQTEGSGVAVKGIAGGD
ASLTVPITKVSSEGTYVCSVRVPPLNGSVDIALHIIEPPNVSLTTKEEQIPRVVCEANGF
YPLDVDIDLFKETSSGQRPEKLGNVLHSSHIQHHDGTHSVAAFVRLRPSPQDSGCETYSC
RVSHVSLQGLYIHKSIIGPGCWTWIHLTLPGLVCLIFVIIFIAVTRQRFF
>SalmonERAP1 XP_013998888.1 ssa15:2.525.189-2.544.613
MRTLTVTILVLLHVSFAPSLAAQLPGDHDTNDKSSSLLPPIATNGQPFPWIHMRLPETVS
PIHYDLLVHPNLTSLDFTGEVQIQLQVFEDTSTIILHSKDLQIAKAELLAPEGPGSLPVP
LQVLEYPAFHQLALMSDVLLVRGGMYKVRLEFSANLSDSFHGFYKSSYRTTKGEVRFMAS
TQFEATSARAAFPCFDEPAFKANFTIQIRRESRHIALSNMPKVKTVELPGGVLEDHFDTS
VRMSTYLVAFIVSDFQSVSKTTSHGVKISVYAVPDKINQTDFALNAAVRLLDFYDDYFDI
PYPLPKQDLAAIPDFQSGAMENWGLTTYREAGLLFDPNKSSASDKLGITMVIAHELAHQW
FGNLVTMQWWNDLWLNEGFAKFMEFVSVNITNPELQVNDFFLGKCFEAMEVDSLSSSHPV
SSQVDTPTQIQEMFDDVSYDKGACILNMLRDFLTPEAFKIGIVRYLRRYSYQNTVNSHLW
ESLTNICQSDDLDEGRLKDEGFCSQEKAQSGAPKWYSGDQLDVRAIMDTWTLQEGFPLVT
VEVRGRQVRLSQERYLKTDDPSQTHGFLWQVPLTYITSSSSTVHRFLLKTRTDVLYLPEE
VGWIKFNVDMSGYYMVHYEGEGWRSLTSLLLTNHRALSSNDRASLINNAFQLVSVGKVGL
DTALDLSLYLSKETDIMPVTQGLGELVPLYKLMEKRDMEGLENQMKGYIVELFRGLIDRQ
TWSDDGSVSQRVLRSYLLLFGSVRNHPPCVATATHLFNKWRASDGNMSLPSDVSLAVFAI
GARDPEGWDFLFEKYRHSQHTSVKSRIKSALSISPLQHKLKRLMEQSLAGEVMKTQDLPH
VVISVSRNPKGYKLAWDFLRHNWHTLVKKFDLGSHSISGMVTGVTNQYSTREMLDEVRGF
FDSLSEETGSGLRCIQQTYESIEENIRWMDQHLPQLKAWLDRQAQGARTETQGHEDL
>SalmonERAP2a XP_014026228.1 ssa24:15.056.418-15.064.045
MVRFLVLALLSLAGVTQTSASPTQASEPPNPTEEQPPLGTGSLSFPWSHLRLPGYIVPLH
YHLLLHPNLTMLSYSGTVRIELQVQNNTNWVVLHSKGLRITTATMLDQNLAHLSDRVLPV
LHNPTHEQTAIFSPRVLSGGQKYFLFLEFGAELGEGFYGFYRSTYRTSAGETRNLASTHF
EPTSARMAFPCFDEPSFKANYSISIRRSLAHTALSNMPVEQTVVLDDGLMEDRFAVSVRM
SSYLVAFIVCDFRSVSATTASGVKVSVYAAPEKWQQTHYALKAAVKLLEFYEKYFNIKYP
LPKQDLVAIPDFQAGAMENWGLITFRETSLLYDPATSSASDRVWVTMVIAHELAHQWFGN
LVTMEWWNDIWLNEGFARYMEYISVNATYPKLRVEDYLVDTCFAAIGRDSLNSSRPISSA
AESPTQIAEMFDTVSYDKGACVLHMLRHYLTDQVFQSGIVRYLRRYSYSNAHNQDLWDSL
ANTCSEEEFTSGEHCYSSRQAAKNAYLYAGEHLDLTTMMNTWMLQTGVPLVTVTRQGSRL

LLKQERFLRTAHPSDPAWPSLQQGFLWHIPLTYRTDTSTSIHRHLMTTLTDSVDVGEEVG
WVKVNVDMAGYYLVHYDGSGWDNLIQLLKDNHTALSFMDRTHLIHNAFQLTTAGRLSLDK
ALDLIGYLRSESHTVPLLEGLGYLEAFYRMVERRDIPDVTQNLRTYILWYFRDVIDRQTW
SDKGSVSERRLRSELLSLACHLGDLPCLKQAQRSFTHWLDSNSTLNLPADVAETVYSVGA
QEDTGWASLLQTYTHSLSETHKRKILSALASSRDTNKLTRLLELGVEGEVIRTQDLDSLI
VMVARNPRGHHLAWSYVQKYWSTLVDKFQLGSFSIRNIIGTTAQFSSTEELTEVRVFFE
SIHEQASQLRVTEVAMDNVQKNILWLQRNLGTLRSWLDQQID
>SalmonERAP2b XP_014016590.1 ssa20:28.634.401-28.645.787
MFWVRFLFLALLSLAGVTQTSSSPTQASEPPNPTEEQPPLNTGSLSFPWSHLRLPEYIVP
LHYHLLLHPNLTILSYIGTVRIELQVQNNTNWVVLHSKGLRITTATVLDQNLAHLSDQVL
PVLHNPTHEQVAIFSPRALTGGQKYFLFLEFGADLGEGFYGFYRSTYRTSTGETRTLAST
HFEPTSARMAFPCFDEPSIKANYSISIRRSPAHTALSNMPVEQTEVLDDGLMEDRFAVSV
RMSSYLVAFIVCDFRSVSATTASGVKVSVYAAPEKWQQTHYALKAAVKLLEFYEKYFNIS
YPLPKQDLVAIPDFQSGAMENWGLITFRETSLLYDPTTSSASDRLWVTKVIAHELAHQWF
GNLVTMEWWNDIWLNEGFATYMEYISVDTTYPKLRVEDYLLDTCFVAIGRDSLNSSRPIS
SVAESPTQIKEMFDTVSYNKGACVLHMLRHYLTDQVFQSGIMRYLRRYSYSNARNQDLWD
SLANTCPEEEFTSGGHCYSNSQAAKNAYLYAGEHLDLTTMMNTWTLQTGVPLVTVARQGS
RLVLKQERFLRTTHPSDPAWPSLQQGYLWHIPLTYRTDTSTSIHRHLMTTLTDSVEVGEE
VGWVKVNVDMAGYYLVHYDGSGWDDLIQLLKNNHTALSFMDRTHLIHNAFQLTTAGRLSL
DKALDLIGYLRSESHTVPLLQGLAYLEAFYRMVERMDIPDVTQNLSTYILWYFRGVIDRQ
TWSDKGSVSERRLRSELLSLACHLGDLPCLEQAQRSFTHWLDSNSTLSLPADVTETVFSV
GAQEDSGWASLLHIYTLSLSETHKHKILSALASSRDTNKLHRLLELGLEGEVIRTQDLDS
LIVMVARNPRGHHLAWSYVQKYWSTLVDKFQLGSFSIRNIIGTTGQFFSTEELTEVRVF
FESIHEQASQLRVTQVAMDNIQKNILWMQRNLGTLRSWLNQHID
>SalmonPSME1a ssa29 NC_027328.1: 20.102.332-20.105.959 XP_014036554.1
MTSIDIRPESKKMVDDFCTRLTKEAETLVTSFFPQKIAEMEMLLKKSFSTDGLAALKSPL
DIPMPDPAKEEAKRKKKEEKEAKEGKKEKDSDKEDEDAGPACGPIPCNERVESLLKEIKP
QIQILKEKLNTVSMWVQLQIPKIEDGNNFGVAVQEKVFELLTNTRTKIEGFQTQISKYYS
ERGDAVDKASKEPHVGDYRQLVHELDQYQYCELRIVVLEIRNTYAVLLDIINKNYDKIKK
PRGDCKALIY
>SalmonPSME1b ssa19 NC_027318.1: 74.398.814-74.401.718 XP_014015182.1
MTSIDIRPESKKQVDDFCTQLTKEAETLVTSFFPQKIAEMDMLLKTSLSTEGLAALKAPL
DIPIPDPAKEEAKQKKKEEKEAKEGKKDKDSEKEDEDAGPPCGPIPCNERVESLLKEIKP
QIQLLKEKLNTVSMWVQLQIPKIEDGNNFGVAVQEKVFELLTNTRTKIEEFQTQISKYYS
ERGDAVAKASKQPHVGDYRQLVHELDQYQYCELRIVVLEIRNTYALLFDIINKNYDKIKK
PRGDCKALIY
>SalmonPSME2a ssa29 NP_001135126.1 NC_027328.1: 17.457.855-17.464.336
MSRSSVLKIKSANAVKVENFRQSLYQQAEDLFSNYIPLKITQLDNLLKEEDLSITDLSTL
HAPLDIPIPDPPTPEDEEMETDKNDDDEKKKKAPKCGFIKGNEKIVKLLDRVKPEILALR
ETIITVSCWIQHLIPKIEDGNDFGVAIQEKILERIVAVKTKVDGFHTNINKYFSERGDAV
SKASKLTHVMDYRSLVHEKDEAVYSDIRVILLDIRGFYAELYDIISKNLEKVTNPKGEEK
PSMY
>SalmonPSME2b ssa19 NC_027318.1: 77.929.964-77.936.657 XP_014015323.1
MSKSSVMKIKSVNAVKVEHFHHSLYQQADDLFSNYIPLKISQLDNLLKEDDLNIPDLSTL
QAPLDIPIPDPPTAEDEEMETDKNDDKKKKKKAPSCGLIKGNEKIVKLLDRVKPEILSLR
ETIITVSCWIQHLIPKIEDGNDFGVAIQEKILERIAAVKTKVDGFHTNINKYFSERGDAV
AKASKSTHVMDYRSLVHEKDEAVYSDIRVILLDIRGFYVELYDIISKNLEKVTNPKGEEK
PSMY

Rainbow trout (*Oncorhynchus mykiss*) MHC Ia region Haplotype Ia_#A; BAC (AB162342) deduced amino acid sequences

BAC sequence reference: see Shiina et al.[4].

>TroutUBA*0501_#A BAD89550.1 AB162342.1:52.087-74.224
MKGFILLLLGIGLLHTASAATHSLRYVYTATSGVPDFPEFVSLGIVDGMQIDYYDSNTKR
VVPKQDWMAKTEGSDYWERQTQNSIGDEQTFKANIDVAKQRFNQTGGVHTFQLMYGCELG
DDGITRGDFQLGYDGADFLSLDKSTLTWTAANQKAVITKLKWDATGAEANFQKNYLENTC
IEWLKKYVNYGKDTLERKVRPSVSLLQKTPSSPVTCHATGFYPSGVMVFWQKDGQEQHGD
VEHGEILQNDDGTFQKSTHLTVTPEEWKNNKYQCVVQLAGIEDDITKVLIESEIQTNFGK

TNRGSNDPITIGLIIGGVIALLVIIVVGVVIWKKKNKKGFVPASTSDTDSENSGKGIQKI
>TroutPSMB8a_#A pseudogene AB162342.1:37.694-38.613
LYKLRNRQRISVSAASKLLCNMMLGYRGMGLSMGSMIVGWDNKGPGLYYVDDNATRLSGR
MFSTGCGSSYAYGVIDSGYREDMTVEEAYELGRRGITHATHRDAYSEGVVNLYHMQEDGW
IKVCKEDVSELIHR*
>TroutPSMB13a_#A BAD89549.1 AB162342.1:33.054-36.174
MALSNVVETPASGFNFENVCRNVALEGLLQGGQTKAPKPMKTGTTIAGVLCKNGVVLGAD
TRATSGEVVADKMCAKIHYISPNIYCCGAGTAADTEKTTDLLSSNLTIFSMNSGRNPRVV
MAVNILQDMLFRYRGQIGASLILGGVDCTGNHLYKVGPYGSIDNVPYLAMGSGDLAALGI
LEDRFKANMEMEEAKELVRDAIHSGIMSDLGSGNNIDICVITKQGVDYIRPYQESEYKDK
RQRRYKYRPGTTSILTEKIVPLELEVVQETVQRMDTA
>TroutPSMB12a_#A BAD89548.1 AB162342.1:23.244-32.906
MEKRFMDSQIKGVSTGTTILAVTFNGGVIIGSDSRASIGGYYVSSKTINKLIQVHDRIFC
CIAGSLADAQAVTKAAKFQISFHSIQMESPPLVKAAASVLKELCYNNKEELQAGFITAGW
DRKKGPQVYTVALGGMLLSQPFTIGGSGSTYIYGYADAKYKPDMSKEECLQFATNALALA
MGRDNVSGGVAHLVVITEEGVEHIVIPGDKLPKFHDE
>TroutPSMB9a_#A PSMB9da BAD89547.1 AB162342.1:19.534-21.449
MLDESLEPGWLSEEVKTGTTIIAIEFDGGVVLGSDSRVSAGETVVNRVMNKLSLLHDKIY
CALSGSAADAQTIAEMVNYQLDVHSIEVGEDPQVRSAATLVKNISYKYKEELSAHLIVAG
WDKRGGGQVYVTLNGLLSRQPFAVGGSGSSYVYGFVDAEYRKAMSKEDCQQFVVNTLSLA
MSRDGSSGGVAYLVTIDEKGAEEKCILGNELPTFYDQ
>TroutTAP2a_#A BAD89546.1 AB162342.1:12.473-18.254
MLRTCAFAVAVVLCVDITTFCAPGFGESISKTGPKTFDTFGNVVRLWVVSGIRLVLLLGL
SLLTLGSIKPVFKRWLAVHCFLAPVYETGRLILYGGSTESACGSLGGPSLWLLCTAAAAA
AALFWEKTFPDSNGESNGKEKTQKARVLFMRVLHFYRPDTLLLVGAFIFLSLAVLCEMFI
PFYTGKVIDILGTQYKWNNFLTAIILMGLYSLGSSFSAGCRGGLFMCAINSFTSRMKVEL
FGALVKQDIGFFETIKTGDITSRLSTDTTLMARAVALNVNVLLRTLIKTVGMLSLMMSLS
WKLTLLMLMETPVTGLLQSVHDNYYLRLSKEVQDSIARANEAAGETVAGIRTVRSFKTEQ
HEAGRYNDRLMDTHNLKTRRDTVRAVYLLLRRLTALVMQVAMLYYGRLFIQRGQMSTGNL
VSFILYQSDLADNIRTLIYIFGDMLNSVGAAGKVFEYLDREPQVSTKGTLQPETLTGHVQ
FHNLSFSYPTRQERKVLQGFSLELRPGQLTALVGPSGGGKSTCVSLLERFYQPQQGEILL
DGLPLQSYQHHYLHRKVAMVGQEPVLFSGSIKDNIAYGLADCSLERVQEAARRANAHSFI
SHLEKGYDTDVGERGGQLSGGEKQRIAIARALIREPQVLILDEVTSALDTESEHMVQEAL
ASCPSQTLLVIAHRLKTIERADQIILIDQGTVQEQGTHQELMDRKGSYYKLRERLFTEDD
MSHCPKQC

Rainbow trout *(Oncorhynchus mykiss)* MHC Ia region Haplotype Ia_#B; genome (GCA_002163495.1) Chr.18 (CM007952.1) deduced amino acid sequences

Unpublished rainbow trout genome assembly GCA_002163495.1.

>TroutTAPBPa_#B CM007952.1:33.137.898-33.141.781 NP_001118025.1
MANISTIYKLSFLAFTYFIQVYGTSCPVLECWFVQEKPGRGGGFPAAMIQEKSLLYINTD
PESEQTKSQQGPSADISHDRVYYITDPAAILCSSSLHPPEGSVHKPQCEINPFMPQPSTV
QWAVPLTDSAHSPIYLQADWYSAALQGLDGQLALSSVMRAPTATKEPTVVLSVSSRTPLV
RSRLGEPVVLDCGFWAETSSPLSGSGFAVEWRYQFRGDGRLVLAYDGKTDRFAETKEKGA
GLDFTALHETGNASLILQEAEVRHTGTYICTVYLPYLLAQVAVELEIVEPPSLSIFPSPL
PLSVPGQVVKVQCEASGFFPLSLEFYWELTGPDGKVRPLGQGSVTGHRQGPDNTYSQTTR
LELDSAKLDLGRGGEVTCVAVHPGGTRRASVTLNVIGVNGPSIEDSMAMVAVALGLYGLI
KIVSWTFSSGSDDTNSQAKKVK
>Trout_UBA*3301_#B CM007952.1:33.177.839-33.203.608 (New trout UBA allele)
XP_021427673.1
MNVKMNGFISLVLGIGLLHTASAATHSLKYFYTAVSGDIDFPEFTAVGLVDKGQFMYFDS
STKTAVPKTEWMKREGADYWDRQTQGLIGAHQTFKVNIQTLKDRFNQSKSTGVHVFQWMY
GCEWDDEAGVTEGFDQYGYDGEDFIAFDLKTTKWIAPTPQAVITKHKWDSNTARNEQNKN
YYTQICIEWLKKYVDYGKSTLMRTVPPSVSLLQKAPSSPVTCHATGFYPRDVMVSWQKDG

QDHHEDVEYGEILQNDDGTFQKSSHLTVTPEDRKNSKYQCVVQVKGIKEDFIGVLPDQDA
ANVVPIIVGVVALLLVVVAVVVGVVIWRKRSKKGFVPASRTESDLENQRRSKDQTNVQRG
IVKN
>TroutPSMB8a_#B pseudogene CM007952.1:33.205.845-33.206.762 XP_021427676.1
LYKLRNRQRISVSAASKLLCNMMLGYRGMGLSMGSMIVGWDNKGPGLYYVDDNATRLSGR
MFSTGCGSSYAYGVIDSGYREDMTVEEAYELGRRGITHATHRDAYSGGVVNLYHMQEDGW
IKVCKEDVSELIHRYRKGMF
>TroutPSMB13a_#B CM007952.1:33.208.283-33.212.648 XP_021427674.1
MALSNVVETPASGFNFENVCRALEGLLQGGQTKAPKPMKTGTTIAGVLCKNGVVLGADTR
ATSGEVVADKMCAKIHYISPNIYCCGAGTAADTEKTTDLLSSNLTIFSMNSGRNPRVVMA
VNILQDMLFRYRGQIGASLILGGVDCTGNHLYKVGPYGSIDNVPYLAMGSGDLAALGILE
DRFKANMEMEEAKELVRDAIHSGIMSDLGSGNNIDICVITKQGVDYIRPYQESEYKDKRQ
RRYKYRPGGTTSILTEKIVPLELEVVQETVQRMDTA
>TroutPSMB12a_#B CM007952.1:33.213.355-33.218.282 XP_021427675.1
TTILAVTFNGGVIIGSDSRASIGGYVSSKTINKLIQVHDRIFCCIAGSLADAQAVTKAAK
FQISFHSIQMESPPLVKAAASVLKELCYNNKEELQAGFITAGWDRKKGPQVYTVALGGML
LSQPFTIGGSGSTYIYGYADAKYKPDMSKEECLQFATNALALAMGRDNVSGGVAHLVVIT
EEGVEHIVIPGDKLPKFHDE
>TroutPSMB9a_#B omy18:33.220.076-33.221.979 XP_021426203.1
MLDESLEPGWLSEEVKTGTTIIAIEFDGGVVLGSDSRVSAGETVVNRVMNKLSLLHDKIY
CALSGSAADAQTIAEMVNYQLDVHSIEVGEDPQVRSAATLVKNISYKYKEELSAHLIVAG
WDKRGGGQVYVTLNGLLSRQPFAVGGSGSSYVYGFVDAEYRKAMSKEDCQQFVVNSLSLA
MSRDGSSGGVAYLVTIDEKGAEEKCILGNELPTFYDQ
>TroutTAP2a_#B CM007952.1:33.223.235-33.229.012 XP_021427678.1
MLRTCAFAVTVGLCIDITTFCAPGFGESISKTGPITFDTFGNVVRLWVVSGIRLVLLLGL
SLGSFTLGSIKPVFKRWLAVHCFLAPVYETGRLILYGGSPESACGSLGGPSLWLLCTAAA
AAAALFWETTFPDSNGESNGKEKTQKARVLFMRVLHFYRPDTLLLVGAFIFLSLAALCEM
FIPFYTGKVIDILGTQYKWNNFLTAIILMGLYSLGSSFSAGCRGGLFMCAINSFTSRMKV
ELFGALVKQDIGFFETIKTGDITSRLSTDTTLMARAVALNVNVLLRTLIKTVGMLSLMMS
LSWKLTLLMLMETPVTGLLQSVHDNYYQRLSKEVQDSIARANEAAGETVAGIRTVRSFKT
EQHEAGRYNDRLMDTHNLKTRRDTVRAVYLLLRRLTALVMQVAMLYYGRLFIQRGQMSTG
NLVSFILYQSDLADNIRTLIYIFGDMLNSVGAAGKVFEYLDREPQVSTKGTLQPETLTGH
VQFHNLSFSYPTRQERKVLQGFSLELRPGQLTALVGPSGGGKSTCVSLLERFYQPQQGEI
LLDGLPLQSYQHHYLHKKVAMVGQEPVLFSGSIKDNIAYGLADCSLERVQEAARRANAHS
FISHLEKGYDTDVGERGGQLSGGEKQRIAIARALIREPQVLILDEVTSALDTESEHMVQE
ALASCPSQTLLVIAHRLKTIERADQIILIDQGTVQEQGTHQELMDRKGSYYKLRERLFTE
DDMSH
>TroutPSMB8F_#B CM007952.1:33.247.717-33.255.385 NP_001158688.1
MALLDVCGISDWVKEDGFNAERASVDKVNHFNFAAQTPELAVPVGVDPAEFLRPLVDSEE
GVDGVKINLEHGTTTLAFKFQHGVMVAVDSRASAGSYVSTQMFKKVIEINPFLLGTMSGS
AADCVYWERVLAKECRIYKLRNKERISVSAASKLLANMVVNYRGMGLSMGTMICGWDKKG
PGLYYVDDNGLRLCGNMFSTGSGNTYAYGVMDSGYRYDLSVPEAYDLAQRAIFHATHRDA
YSGGTVNVYHMRETGWIKVSQEDVGDLYHRFSNEKK*
>TroutPSMB10a_#B 18:33.608.302 -33.611.763 XP_021427701.1
MLHNSRPPQPQSAGFSFENTRRNAVLEGNLSELGYSSPKARKTGTTIAGIVFKDGVILGA
DTRTTDDMVVADKNCMKIHYIAPNISCCGAGVAADAEVTTQMMSSNVELHSLSTGRPPLV
VTVTRQLKQMLFRYQGHIGSSLIVGGVDVTGAHLYSVYPHGSYDKLPFLTMGGSGAGAAI
SIFEDRYRPNAQLEEAKKLVRDAIAAGIFCDLGSGSNVDLCVITHAGVQYLRGYDQPAQK
GKSRGQYTYKPGTTAVLTKTVTPLPLDIVDESIQLMDTQ

Rainbow trout (*O. mykiss*) MHC Ib region Haplotype Ib_#A; BAC sequence (AB162343) deduced amino acid sequences

BAC sequence reference: see Shiina et al.[4].

>TroutUDA*0105_#A BAD89551.1
MKGFILMVLGIVHLHEAFGVTHSLTHFYTASSDIPNFPEFVFVGMVDGVQMVHYDSNIQK
VVPEQDWMKQTDAEFCEKERENLFYSQQSFKAEVATLKQHFNQSGGVHILQYMYGCSRDD
ETEQTEGFGQLGYNGENFLEYDMKTLTWKSLKQQPNFMQDEWNSDTSRLLFWKNYFTQTC

IECLKKQVDNRKSTLMRTVPPSVSLLQKSPSSPVTCHATGFYPSGVMVFWQKDGQGHQEG
VENGETLPNNDRTFQKSTHLTVTPEEWKNNKYQCVVQVTGIKEDFIKVLTESEIQTNWKD
PAPNIVPIIGGVVALLLVVVVVVVVVVIWKKKKKKGFVPASTNDTDSAYSGEGLLKTSVL
SALLKQLKTVTK
>TroutUCA*0101_#A BAD89552.1
MKGFMLMFMGIGHLYEAFAVTHSLTHFYTASSEVYNFPEFMVVGMVDGVQMVHYDSNSQK
AVPEQDWMKQTDAEFWEKERENFFYSQQSFKAEVATLKQHFNQSGGVHILQYMYGCSRDD
ETEQTEGFGQLGYNGENFLEYDMKTLTWKSRKQQANFMQDEWNSDISRLGFWKIYFSQTC
IECLKKQVDNGKRTLRRTVPPSVSLLQKTPSSPVTCHATGFYPSGVMVSWQKDGQDHHED
VEHGEILFNDDGTFQKSTHLTVMHEERKNKNYQCVVQVTGIKEDFIKVLTESEIQTNWKD
PAPNIIPIIGGMVAPFLVVVVVGVVVIWKKKKSKKGFVPASTSDTDSDNSGKGLLK
>TroutUEA*0101_#A BAD89553.1
MISFCLLFLWIPSSSADPHSLKYLYTAVSGDTDFPEFTVVGLLDDQQFVHFGSNTKTLVN
DAEWMNKTGKYYNDLYNEPLINQYEGFKNLITFAKKQFNQTQSKGVHTIQNLYGCEWNDE
TELQDYFHHYGYDGEDFISLDMKTVRWITSVQQADTIKQKWDDNSKDLHYLKWYFTKECI
DTLKKYVDFASSVLKKIVPPSVSLLQKTPSSPVTCHATGFYPSGVMVSWQKDGQEQHEDV
LHGEILLNGDGTFQKSAHLTIDSEKWENNNYTCVVEHKENIIAIRLNQSVIKTNSVKPSE
YVPIIIGLGVVAVILLVLVPIITFIMWNRKSTASDDGSNPSMTQQNQIQLEVSLSLIKQE
EAEKRGPLLKDSEAS
>TroutPSMB8b_#A BAD89554.1
MALFDVSGYKSHAGLRGQILGTGVGHLVDRPNQEFAVPVGVDPSGFLKSCSREGGVSIDL
NHGTTTLAFKFRHGVIVAVDSRASAGSYIASKEANKVIEINPYLLGTMSGSAADCQYWER
LLAKECRLYKLRNKQRISVSAASKLLCNMMLGYRGMGLSMGSMIVGWDNKGPGLYYVDDN
ATRLSGRMFSTGCGSSYAYGVMDSGYREDMTVEEAYELGRRGITHATHRDAYSGGVVNLY
HMQEDGWIKVCKEDVSELIHRYRKGMF
>TroutPSMB13b_#A BAD89555.1
MALSNVVETPASGFNFENVSRNVALEGLLEGGHTKAPKPMKTGTTIAGIVCKDGVVLGAD
TRATSGEVVADKMCAKIHYISPNMYCCGAGTAADTEKTTDLLSSNLTIFSMNSGRNPRVV
MAVNILQDMLFRYRGQIGASLILGGVDCTGNHLYTVGPYGSIDNVPYLAMGSGDLAALGI
LEDRFKPNMELEEAKQLVRDAIHSGIMSDLGSGNNIDICVITKQGVDYIRPYQESEYKDK
RQRRYKYRPGTTSILTEKIVPLELEVVQETVQRMDTA
>TroutPSMB12b_#A BAD89556.1
MDSQIKGVSTGTTILAVTFNGGVIIGSDSRASIGGNYVSSKTINKLIQVHDRIFCCIAGS
LADAQAVTKAAKFQISFHSIQMESPPLVKAAASVLKELCYNNKEELQAGFITAGWDRKKG
PQVYTVALGGMLLSQPFTIGGSGSTYIYGYADAKYKPDMSREECLQFATNALALAMGRDN
VSGGVAHLVVITEEGVEHVVIPGDKLPKFHDE
>TroutPSMB9b_#A BAD89557.1
MLDESSEPGWLSEEVKTGTTIIAIEFDGGVVLGSDSRVSAGETVVNRVMNKLSLLHDKIY
CALSGSAADAQTIAEMVNYQLDVHSIEVGEDPQVRSAATLVKNISYKYKEELSAHLIVAG
WDKRGGGQVYVTLNGLLSRQPFAVGGSGSSYVYGFVDAEYRKAMSKEDCQQFVVNTLSLA
MSRDGSSGGVAYLVTIDEKGAEEKCILGNELPTFYDQ
>TrourTAP2b_#A BAD89558.1
MLRTCVFAMAVGLCIDITTFCATGFGASIPKTGPISFDVFGSLLRLWVVAGIRLVILFGV
SLLTLGSIKPVLKRWLAVHCFLAPVYETGQLMLHGSSPESTHGSLGGPSLWLLCTAAAAA
AALFWEKTFPDSSEESNGKEKTQKARVLFMRVLYFYRPDTLLLVGAFIFLSLAVLCEMFI
PFYTGKVIDILASQYKWNDFLTAIILMGLYSLGSSFSAGCRGGLFMCAINSFTSRMKVEL
FGALVKQEISFFETIKTGDITSRLSTDTTKMARAVALNVNVLLRTLIKIVGMLSLMMSLS
WKLTLLMLMETPVTGLLQGVYDNYYLRLTKEVQDSMARANEAAGETVAGIRTVRSFNTER
SEASHYDHRLMDTHTLKTRRDTVRAVYVLLRRLTALVMQVAMLYYGRLFIQRGQMSTGNL
VSFILYQSNLGTNIRTLIYIFGDMLNSVGAAGKVFEYLDREPQVSTKGTLQPETLTGHVH
FHNLSFSYPTRQERKVLQGFSLELRPGQLTALVGPSGGGKSTCVSLLERFYQPQQGEILL
DGQPLHSYQHHYLHRKVAMVGQEPVLFSGSIKDNIAYGLADCSLERVQEAARRANAHSFI
SQLEKGYDTDVGERGGQMSGGEKQRIAIARALIREPQVLILDEVTSALDTESEHMVQEAL
TSCPSQTLLVIAHRLKTIERADQIILIDQGTVLEQGTHQELMDRKGGYYKLRERLFTEDD
TSH

Rainbow trout (*O. mykiss*) MHC IB region Haplotype Ib_#B; Genome Chr.14 (CM007948.1) deduced amino acid sequences

Unpublished rainbow trout genome assembly GCA_002163495.1.

```
>TroutUDA_#B CM007948.1:28.624.919-28.630.481 XP_021416252.1
MSLTHSLTHFYTASSDIPNFPEFVFVGMVDGVQMVHYDSNIQKVVPEQDWMKQTDAEFCE
KERENLFYSQQSFKAEVATLKQHFNQSGGVHILQYMYGCSRDDETEQTEGFGQLGYNGEN
FLEYDMKTLTWKSLKQQPNFMQDEWNSDTSRLLFWKNYFTQTCIDCLKKQVDNRKSTLRR
TVPPSVSLLQKTPSSPVTCHATGFYPSGVMVFWQKDGQEQHEDVENGETLPNDDGTFQKS
THLTVTPKEWKNNKYQCVVQVTGIKEVFIKVLTESEIQTNWKDPAPNIVLHIGGVVALLL
VVVVVVGVVIWKKKKKKGFVPASRSS
>TroutTAPBPb_#B CM007948.1:28.615.561-28.617.272 XP_021416251.1
MDVYGTSCPVLECWFVQEKPGGGFHAAMSQEKSLLYINTDPDSEETRSKQGPPTDHDRVY
YVTDPAATLCSSSLHPPEGSVQKSQCEINPFMPQPSTVQWTVPLTDSAHSPIYLQADWYT
AALQGFDGQLRLSNVLRAPTATKEPKVVLSVSSRTFMIRSRLGEPVVLDCGFWVDASSPL
SGSGFAVEWRYQFRGDGRLVLAYDGKSDRFAETQEEGVGLDFTALHETGDASLILQEAQV
RHSGTYICTVYLPYLLAQVAVELEIVGEER
>TroutUCA CM007948.1:28.598.666-28.609.318 XP_021416249.1
MKGLMLMFMGIGHLYEAFGVTHSLKHFYTASSDIPNFPEFVFVGMVDDVQMVHYDSNSQK
AVPKQDWMKQTDAEFWEKERENFFYSQQSFKAEVATLKQHFNQSGGVHILQYMYGCSRDD
ETEQTEGFGQLGYNGENFLEYDMKTLTWKSRKQQANFMQDEWNRDISRLGFWKIYFSQTC
IDCLKKQVDNGKSTLMRTVSLLQKTPSSPVTCHATGFYPSGVMVSWQKDGQDHHEDVENG
ETLPNDDGTFQKSTHLTVTPKEWKNNKYQCVVQVTGIKEDFIKVLTESEIQTNWKDPAPN
IVPIIGGMVALLLVVVVVVGVVIWKKKSKKGFVPASRSNKLLKNITYNTYNITHSIYIEQ
KYKRNSLKDFAELKFI
>TroutPSMB8bps_#B chr.14 CM007948.1:28.592.051-28.592.188
LYKLRNKQRISVSAASKLLCNMMLRYRGMGLSMGSMIVGWDNKVQG
>TroutUEA_#B CM007948.1: 28.580.630-28.582.037 XP_021416247.1
PHSLKYLYTAVSGDTDFPEFTVVGLLDDQQFVHFGSNTKTLVNDAEWMNKTGKYYNDLYN
EPLINQYEGFKNLITFAKKQFNQTQSKGVHTIQNLYGCEWNDETELQDYFHHYGYDGEDF
ISLDMKTVRWITSVQQADTIKQKWDDNSKDLHYLKWYFTKECIDTLKKYVDFASSVLKKI
VSLLQKTPSSPVTCHATGFYPSGVMVSWQKDGQEQHEDVLHGEILLNGDGTFQKSAHLTI
DSEKWENNNYTCVVEHKENIIAIRLNQSVIKTNSVKPSEYVPIIIGVGVVAVILLVLVPI
ITFIMWNRKST
>TroutUFA_#B chr.14 CM007948.1:28.552.026-28.552.825 No predicted orf
VSWMMTADFLSLDKSTRTWTAANQKAAIS*LKWDATGDNANYWKNYLENTCIEWLKKYVN
YGKDTLERSVSLLQETPSSPVTCHATGFYPSGVMVFWQKDGQGHQEGVENGETLPNNDGT
FQKSTHLKVTPEEWKNNKYQCVVQVTGIKEDFIKVLTESETQPIWR
>TroutPSMB8b_#B chr.14 CM007948.1:28.546.790-28.549.183 XP_021416243.1
MALFDVSGYKSHAGLRGQILGTGVGHLVDRPNQEFAVPVGVDVSPSGFLKSCSREGGVSI
DLNHGTTTLAFKFRHGVIVAVDSRASAGSYIASKEANKVIEINPYLLGTMSGSAADCQYW
ERLLAKECRLYKLRNKQRISVSAASKLLCNMMLGYRGMGLSMGSMIVGWDNKGPGLYYVD
DNATRLSGRMFSTGCGSSYAYGVMDSGYHEDMTVEEAYELGRRGITHATHRDAYSGGVVN
VYHMQEDGWIKVCKEDVSELIHRYRKGMF
>TroutPSMB13b_#B CM007948.1:28.542.425-28.549.187 XP_021416241.1
MALSNVVETPASGFNFENVSRNVALEGLLEGGHTKAPKPMKTGTTIAGIVCKDGVVLGAD
TRATSGEVVADKMCAKIHYISPNMYCCGAGTAADTEKTTDLLSSNLTIFSMNSGRNPRVV
MAVNILQDMLFRYRGQIGASLILGGVDCTGNHLYTVGPYGSIDNVPYLAMGSGDLAALGI
LEDRFKPNMELEEAKQLVRDAIHSGIMSDLGSGNNIDICVITKQGVDYIRPYQESEYKDK
RQRRYKYRPGTTSILTEKIVPLEME
>TroutPSMB12b_#B CM007948.1:28.539.879-28.542.248 XP_021416245.1
MDSQIKGVSTGTTILAVTFNGGVIIGSDSRASIGGNYVSSKTINKLIQVHDRIFCCIAGS
LADAQAVTKAAKFQISFHSIQMESPPLVKAAASVLKELCYNNKEELQAGFITAGWDRKKG
PQVYTVALGGMLLSQPFTIGGSGSTYIYGYADAKYKPDMSREECLQFATNALALAMGRDN
VSGGVAHLVVITEEGVEHVVIPGDKLPKFHDE
>TroutPSMB9b_#B CM007948.1:28.534.744-28.538.439 XP_021416244.1
MLDESSEPGWLSEEVKTGTTIIAIEFDGGVVLGSDSRVSAGETVVNRVMNKLSLLHDKIY
```

```
CALSGSAADAQTIAEMVNYQLDVHSIEVGEDPQVRSAATLVKNISYKYKEELSAHLIVAG
WDKRGGGQVYVTLNGLLSRQPFAVGGSGSSYVYGFVDAEYRKAMSKEDCQQFVVNTLSLA
MSRDGSSGGVAYLVTIDEKGETFVVP
>TroutTAP2b_#B 14:28.526.642-28.532.384 XP_021416240.1
MLRTCVFAMAVGLCIDITTFCATGFGASIPKTGPISFDVFGSLLRLWVVAGIRLVILFGV
SLLTLGSIKPVLKRWLAVHCFLAPVYETGQLMLHGSSPESTHGSLGGPSLWLLCTAAAAA
AALFWEKTFPDSSEESNGKEKTQKARVLFMRVLYFYRPDTLLLVGAFIFLSLAVLCKYFI
EMFIPFYTGKVIDILASQYKWNDFLTAIILMGLYSLGSSFSAGCRGGLFMCAINSFTSRM
KVELFGALVKQEISFFETIKTGDITSRLSTDTTKMARAVALNVNVLLRTLIKIVGMLSLM
MSLSWKLTLLMLMETPVTGLLQGVYDNYYLRLTKEVQDSMARANEAAGETVAGIRTVRSF
NTERSEASHYDHRLMDTHTLKTRRDTVRAVYVLLRRLTALVMQVAMLYYGRLFIQRGQMS
TGNLVSFILYQSNLGTNIRTLIYIFGDMLNSVGAAGKVFEYLDREPQVSTKGTLQPETLT
GHVHFHNLSFSYPTRQERKVLQGFSLELRPGQLTALVGPSGGGKSTCVSLLERFYQPQQG
EILLDGQPLHSYQHHYLHRKVAMVGQEPVLFSGSIKDNIAYGLADCSLERVQEAARRANA
HSFISQLEKGYDTDVGERGGQMSGGEKQRIAIARALIREPQVLILDEVTSALDTESEHMV
RHPSTHKLECFSLPSQTLLVIAHRLKTIERADQIILIDQGTVLEQGTHQELMDRKGGYYK
LRERLFTEDDTSH
>TroutUGAps_#B CM007948.1: 28.280.757-28.280.987
VPASLSLLQKTHSSPVTCHATGFYPSGVMVFWQKDGPDHHEDVEYGETLPNKDGTFQKNT
HQWSSGKATINVWFSSQYTGSNRQPSRPIQSDE
>TroutUGA_#B CM007948.1: 28.275.281-28.276.500   MHCI
MCYMSVRHSLRYFYTTSSEIPAFPEFVDMGMVNDQVISHYDSITKRKVPKQSWMETFFDQ
QYWDSTTENLKSAESIFKTNIQIAQKRFNQTGGIHISQDMYGCTWDDETGFTEGFHHIGY
DGQDLLTFDVKTATWIALVPQALHSKMKWEMDLSGIKSKKSYLTQDCIEWLKKYLDYGKT
TLQRTVPPSVSLLQKTPSSPVTCHATGFYPSGVMVFWQKDGQEQHEDVENGETLPNDDGT
FQKSTHLTVTPEEWKNNKYQCVVQVTGIKEDFIKVLTEFEIQTNWGDPAPNCIPIIGGVV
ALLLVVVVVGVVIWKKKSKKGF
>TroutPSMB10b_#B CM007948.1:28.118.268-28.125.740
MLNNSRPCQPQSAGFSFENTRRNAVLEGNLSELGYSSPKARKTGTTIAGIVFKDGVILGA
DTRATDDMVVADKNCMKIHYIAPNIYCCGAGVAADAEVTTQMMSSNVELHSLSTGRPPLV
VTVTRQLKQMLFRYQGHIGSSLIVGGVDVTGAHLYSVYPHGSYDKLPFLTMGSGAGAAIS
IFEDRYRPNMELEEAKKLVRDAIAAGIFCDLGSGSNVDLCVITQAGVQYLRGYDQPAPGT
TAVLTKTVTPLPLDVVDESIQLMDTQ
```

Coho salmon (*O. kisutch*) MHCIa region; Genome GCA_002021735.1 LG17 NC_034190: 25.300.000-26.000.000 deduced amino acid sequences

Coho salmon genome assembly unpublished.

```
>CohoTAPBPa NC_034190:25.979.910-25.984.738 XP_020361139.1
MANISTIYKLSFLAFTYFIQVYGTSCPVLECWFVQEKPGRGGGFPAAMIQEKALLYINTD
PESEQTKSQQRPSADISHDRVYYITDPAAILCSSSLHPPEGSVHKPQCEINPFMTQPSTV
QWAVPLTDSAHSPIYLQADWYSAALQGLDGQLALSSVMRAPTATKEPTVVLSVSSRTPLV
RSRLGEPVVLDCGFWAEASSPLSGSGFAVEWRYQFRGDGRLVLAYDGKTDRFAETKEKGA
GLDFTALHETGNASLILQEAEVRHTGTYICTAYLPYLLAQVAVELEIVEPPSLSIFPSPL
PLSVPGQVVKVQCEASGFFPLSLEFYWELTGPDGKVRPLGQGSVTGHRQGPDNTYSQTTR
LELDSAKLDLGRGGEVTCVAVHPGGTRRASVTLNVIGVNGPSIEDSMAMVAVALGLYGLI
KIVSWTFSSGSDDTNSQEKKVK
>CohoUBA NC_034190:25.919.304-25.940.726 XP_020361135.1
MKGIILLVLGIGLLHTASAVTHSLKYFYTASSEVPNFPEFVVVGMVDGVQMVHYDSNSQR
AVPKQDWVNKAADPQYWERNTGNFKGSQQTFKANIDIVKQRFNQSGGVHIVQMMYGCEWD
DETGVTEGFEQHGYDGEDFIAFDLKTTKWIAPMPQAVITKHKLDSNTANNEYKKNYYTQT
CIDWLKKYVDYGKSTLMRTVCPSVSLFQKTSSSLVTCHATGFYPSDVMVSWQKDGQDHHE
DVEYGETLPNDDGTFQKSSHLTVTPEDRKNSKYQCVVQVKGIKEDFIKVLPDLDAANVVP
IIVGVVALLLVVVAVVVGVVIWRKRSKKGFVPTSTSDTDSDNSGRAAPQI"
>CohoPSMB8a NC_034190:25.910.797-25.913.925 XP_020361133.1
```

MALFDVSGYKSHSELRGQIIGTGVGHFIDRPNQQFSVPVGVDPSGFLKSCSREGGVSIDL
NHGTTTLAFTFRHGVIVAVDSRASAGSYIESKEANKVIEINPYLLGTMSGSAADCQYWER
LLAKECRLYKLRNKQRISVSAASKLLCNMMLGYRGMGLSMGSMIVGWDNKGPGLYYVDDN
ATRLSGRMFSTGCGSSYAYGVIDSGYREDMTVEEAYELGRRGITHATHRDAYSGGVVNLY
HMQEDGWIKVCKEDVSELIHRYRKGMF"
>CohoPSMB13a NC_034190: 25.904.598-25.910.466 XP_020361130.1
MALSNVLEIPTSGFNFENVARNVALEGLLEGGQTKTPKPMKTGTTIAGLVCKEGVVLGAD
TRATSGEVVADKMCAKIHYISPNIYCCGAGTAADTEKTTDLLSSNLTIFSMNSGRNPRVV
MAVNILQDMLFRYRGQIGASLILGGVDCSGNHLYTVGPYGSIDNVPYLAMGSGDLAALGI
LEDRFKPNMEMEEAKELVRDAIHSGIMSDLGSGNNIDICVITKQGVDYIRPYQESEYKDK
RQKRYKYGPGTTSILTEKIVPLELEVVQETVQQMDTA
>CohoPSMB12a NC_034190: 25.901.088-25.904.637 XP_020361131.1
MERHLMDSQIKGVSTGTTILAVTFNGGVIIGSDSRASIGGYYVSSKTINKLIQVHDRIFC
CIAGSLADAQAVTKAAKFQISFHSIQMESPPLVKAAASVLKELCYNNKEELQAGFITAGW
DRKKGPQVYTVALGGMLLSQPFTIGGSGSTYIYGYADAKYKPDMSKEECLQFATNALALA
MGRDNVSGGVAHLVVITEEGVEHIVIPGDKLPKFHDE"
>CohoPSMB9a NC_034190: 25.895.415-25.899.072 XP_020361128.1
MLEESSEPGWLSEEVKTGTTIIAIEFDGGVVLGSDSRVSAGETVVNRVMNKLSLLHDKIY
CALSGSAADAQTIAEMVNYQLDVHSIEVGEDPQVRSAATLVKNISYKYKEELSAHLIVAG
WDKRGGGQVYVTLNGLLSRQPFAVGGSGSAYVYGFVDAEYRKAMSKEDCQQFVVNTLSLA
MSRDGSSGGVAYLVTIDEKGAEEKCILGNELPTFYDQ"
>CohoTAP2a NC_034190: 25.890.101-25.894.811 XP_020361127.1
MTKMMLRTCVFAMAVGLCIDITTLAFGASISKTGTRTSDAFGNVVRLWVVAGIRLVLLLG
LSLLTLGSIKPVFKRWLAVHCFLAPVYETGRLMLYGGSPERVYRSLGLGSPSLWPLCTVA
AAAAALFWETTFPDSNGESNGKQKKQKARVLFMRVLIFYRPDILLLVGAFIFLSLAVLCE
MFIPFYTGKVIDILGTQYKWNNFLTAIILMGLYSLGSSFSAGCRGGLFMCAINSFTCRMK
VKLFGALVKQEIGFFETIKTGDITSRLSTDTTLMGRAVALNVNVLLRTLIKTVGMLSLMM
SLSWKLTLLMLMETPITGLLQSVHDNYYQRLSKEVQDSIARANEAAGETVGGIRTVRSFK
TEQHEAGRYNDRLMDTHNLKTRRDTVMAVYLLLRRLTAVVMQVAMLYYGRLFIQRGQMTT
GNLVSFILYQSDLADNIRTLIYIFGDMLNSVGAAGKVFEYLDREPQVSTKGTLQPETLTG
HVQFHNLSFSYPTRQERKVLQGFSLELRPGQLTALVGPSGGGKSTCVSLLERFYQPQQGE
ILLDGLPLQSYQHHYLHRKIAMVGQEPVLFSGSVKDNIAYGLADCSLERVQEAARRANAH
SFISQLEKGYDTDVGERGGQLSGGEKQRIAIARALIREPQVLILDEVTSALDTESEHMVQ
EALASCPSQTLLVIAHRLKTIERADQIILIDQGTVQEQGTHQELMDRKGSYYKLKERLFT
EDDVPH
>CohoPSMB8Fa NC_034190.1:25.878.698-25.879.229
RIYKLRN*ERISVSAASTLLANMVVNYRGMGLRGTMICGCDQGPGLYYVDDNGLRLGNML
TTGSGNYGMMDSGSQYDLSVPEAYDLAQRAIFHATHRDAYSGGTVN
>CohoPSMB10a LG17 NC_034190.1: 25.541.569 -25.544.041 XP_020361094.1
MLHNSRPPQPQSAGFSFENTRRNAVLEGNLSELGYSSPKARKTGTTIAGIVFKDGVILAA
DTRATDDMVVADKNCMKIHYIAPNIYCSCCGAGVAADAEVTTQMMSSNVELHSLSTGRPP
LVVTVTRQLKQMLFR*ES-MNESVTMGK*DLYSGHSHNMAAISIFEDRYRPNLEEAKKLV
RDAIAAGIFCDLGSGSNVDLCVITQAGVQYLRGYDQPAQKGKKEGQYKYKPGTTAVLTKT
VTPLPLDIVDESIQLMDTQ

Coho salmon (*O. kisutch*) MHCIb region; LG14, NC_034187 22.700.000-23.500.000 deduced amino acid sequences

>CohoTAPBPb NC_034187:23.335.122-23.343.564 XP_020356015.1
MTNISIILQSFLAFSYVMHVYGASCPVLECWFVQEKPGGGFHAAMSQEKSLLYINTDPDS
EETRSKQGPPTDHDRVYYVTDPAATLCSSSLHPPEGSVQKSQCEINPFMPQPSTVQWTVP
LTDSAHSPIYLQADWYTATLQGFDGQLRLSNVLRAPTATKEPKVVLSVSSRTFMIRSRLG
EPVVLDCGFWVDASSPLSGSGFAVEWRYQFRGDGRLVLAYDGKSDRFAETQEEGSGLDFT
ALHETGDASLILQEAQVRHSGTYICMVYLPYLLAQVAVELEIVEPPSLSIFPSPLPLSMP
GQVVTVQCEASGFYPLSLEFRWMLTGPDGKVRPLGQGSVTGHRQGPDSTFSQTSRLELDS
AKLDLGGEVTCVAVHHGGTRRASVTLNIAGVSAPSIEDYMAMVAVALGIYGLIQVVSWTF
>CohoUDA NC_034187:23.304.841-23.315.855 XP_020356011.1
MKAFMLMFMGIGHLHEAFAVTHSLEHFYTASSELHNFPEFMVVGMVDGVQMVHYDSNIQK
AVPEKDWMKQTDTEFWEKEKENFFYSQKSFKAEVANLKQHLNQSGGVHILQYMYGCSRDD

ETEQTEGFGQLGYNGENFLEYDMETLTWKSRKPQANFMQDEWNSDISRLGFWKNYFSQTC
IDCLKKQVDNGKSTLMRTVPPSVSLLQKTPSSPVTCHATGFYPSGVMVFWQKYGKYQHEG
VEHGEILFNDDETFQKSTHLTVMPEEWKNNKYQCVVQVTGIKKDFIKVLTESEIQTNWKD
PAPNIVLYIGGVVAFLLVVVVVVVGVIIWKKKSKKGFVPASTSNTDWDNSG
>CohoPSMB8b3 NC_034187.1:23.299.625-23.299.750
SKLLCNMMLGYRGMGLSMGSMIVGWDNKVQG
>CohoPSMB8b2 NC_034187.1:23.289.363 23.296.651
ALKEANKVIEINPYLLGTMSGSAADCQYWERLLAKECRLYKLRNKQRISVSAASKLLCNMMLGYRGMGLS
>CohoUEA NC_034187:23.276.214-23.279.219 XP_020356010.1
MISFCLLFLWIPTMSADPHSLKYIYTAVSGDTDFPEFTVVGLLDDQQFVHFGSNTKTLVN
DAEWMNKTGKYYRELYNETLINQYEGFKNLITFAKKQFNQTQSKGVHTIQNLYGCEWNDE
TELQDYFHHYGYDGEDFISLDMKTVRWITSVQQADTIKQKWDDNSKDLHYLKWYFTKECI
DTLKEYVNFASSVLKKIVPPSVSLLQKTPSSLVTCHATGFYPSGVMVSWQKDGQEQHEDV
LHGEILPNGDGTFQKSAHLTIDSEKWENNNYTCVVEHKENIIAIRLNQSVIKTNSVKLLE
TVPIIIGGGVVAVILLVTFIITRFIMWNRKSTASDDGSNPSMTQQNQIQLEVSLSLIKQE
RSREKRTFIEGQ
>CohoPSMB8b NC_034187:23.256.442-23.259.352 XP_020356008.1
MALFDVSGYKSHAGLRGQILGTGVGHLVDRPNQEFAVPVGVDPSGFLKSCSREGGVSIDL
NHGTTTLAFKFRHGVIVAVDSRASAGSYIASKEANKVIEINPYLLGTMSGSAADCQYWER
LLAKECRLYKLRNKQRISVSAASKLLCNMMLGYRGMGLSMGSMIVGWDNKGPGLYYVDDN
ATRLSGRMFSTGCGSSYAYGVMDSGYREDMTVEEAYELGRRGITHATHRDAYSGGVVNLY
HMQEDGWIKVCKEDVSELIHRYRKGMF
>CohoPSMB13b NC_034187:23.251.688-23.255.606 XP_020356007.1
MALSNVVETPASGFNFENVSRNVALEGLLEGGHTKAPKPMKTGTTIAGVVCKDGVVLGAD
TRATSGEVVADKMCAKIHYISPNMYCCGAGTAADTEKTTDLLSSNLTIFSMNSGRNPRVV
MAVNILQDMLFRYRGQIGASLILGGVDCTGNHLYTVGPYGSIDNVPYLAMGSGDLAALGI
LEDRFKPNMELEEAKELVRDAIHSGIMSDLGSGNNIDICVITKQGVDYIRPYQESEYKDK
RQRRYKYRPGTTSILTEKIVPLELEVVQETVQRMDTA
>CohoPSMB12b NC_034187:23.248.913-23.251.681 XP_020356009.1
MDSQIKGVSTGTTILAVTFNGGVIIGSDSRASIGGYYVSSKTINKLIQVHDRIFCCIAGS
LADAQAVTKAAKFQISFHSIQMESPPLVKAAASVLKELCYNNKEELQAGFITAGWDRKKG
PQVYTVALGGMLLSQPFTIGGSGSTYIYGYADAKYKPDMSREECLQFATNALALAMGRDN
VSGGVAHLVVITEEGVEHVVIPGDKLPKFHDE
>CohopSMB9b NC_034187:23.243.647-23.248.046 XP_020356006.1
MLDESSEPGWLSEEVKTGTTIIAIEFDGGVVLGSDSRVSAGETVVNRVMNKLSLLHDKIY
CALSGSAADAQTIAEMVNYQLDVHSIEVGEDPQVRSAATLVKNISYKYKEELSAHLIVAG
WDKKGGGQVYVTLNGLLSRQPFAVGGSGSSYVYGFVDAEYRKAMSKEDCQQFVVNTLSLA
MSRDGSSGGVAYLVTIDEKGAEEKCILGNELPTFYDQ
>CohoTAP2b NC_034187:23.236.849-23.242.173 XP_020356005.1
MMLRTCVFAMAVGLCIDITTFCATGFGASISKTGPISFDLFGNLLRLWVVAGIRLVILFG
VSLLTLGSIKPVLKRWLTVHCFLAPVYETGQLMLHGSSPESTHGSLGGPSLWLLCTAAAA
AAALFWEKTFPDSNEESNGKEKTQKARVLFMRVLYFYRPDTLLLVGAFIFLSLAVLCEMF
IPFYTGKVIDILASQYKWNDFLTAIILMGLYSLGSSFSAGCRGGLFMCAINSFTSRIKVE
LFGALVKQDISFFETIKTGDITSRLSTDTTKMARAVALNVNVLLRTLIKIVGMLSLMMSL
SWKLTLLMLMETPITGLLQGVYDNYYLRLTKEVQDSMARANEAAGETVAGIRTVRSFNTE
RSEAGHYDHRLMDTHNLKTRRDTVRAVYVLLRRLTALVMQVAMLYYGRLFIQRGQMSTGN
LVSFILYQSNLGTNIRTLIYIFGDMLNSVGAAGKVFEYLDREPQVSAKGTLQPETLTGHV
HFHNLSFSYPTRQERKVLQGFSLELRPGQLTALVGPSGGGKSTCVSLLERFYQPQQGEIL
LDGQPLHSYQHHYLHRKVAMVGQEPVLFSGSIKDNIAYGLADCSLERVQEAARRANAHSF
ISQLEKGYDTDVGERGGQMSGGEKQRIAIARALIREPQVLILDEVTSALDTESEHMVQEA
LASCPSQTLLVIAHRLKTIERADQIILIDQGTVLEQGTHQELMDRKGGYYKLRERLFTED
DTSH
>CohoUGA NC_034187:22.955.555..22.971.186 XP_020355985.1
MLLYHFSPRCILRHSLRYFYTTSSEIPAFPEFVDMGMVNDQVISHYDSITKRKVPKQSWM
EPFFNQQYWDSTTENLRSAESVFKTNIQIAQKRFNQTGGIHISQDMYGCTWDDETGFTEG
FHHIGCDGQDLLAFDVKTATWIASVPQALHSKMKWEMDLSSIESKKSYLTQDCIEWLKKY
LDYGMTTLQRTVPPSVSLLQKTPSSPVTCHATGFYPSGVMVSWQKDGQEQHEDVENGETL
PNDDGTFQRSTHLKVMPEEWKNNKYQCVVQVTGIKKDFIKVLTEFEIQTNWGDPAPNIIP
IIGGVVALLLVVVVVVVVVVGVVIWKMKSKKGFVPARTSDTDSENSGERSPDDLKNFCNIR
ELKRTATEITNRNFPRL
>CohoPSMB10b NC_034187:22.819763-22.825.755 XP_020355973.1

MLNNSRPCQPQSAGFSFENTRRNAVLEGNLSELGYSSPKARKTGTTIAGIVFKDGVILGA
DTRATDDMVVADKNCMKIHYIAPNIYCCGAGVAADAEVTTQMMSSNVELHSLSTGRPPLV
VTVTRQLKQMLFRYQGHIGSSLIVGGVDVTGAHLYSVYPHGSYDKLPFLTMGSGAGAAIS
IFEDRYRPNMELEEAKKLVRDAITAGIFCDLGSGSNVDLCVITQAGVQYLRGYDQPAQKG
KREGQYKYKPGTTAVLTKTVTPLPLDVVDESIQLMDTQ

Northern pike (*Esox Lucius*) genome GCA_000721915.3 MHC Ia region LG10 (NC_025977.3) deduced amino acid sequences

Northern pike genome assembly: Rondeau et al.[5].

>PikeTAPBP NC_025977.3:7.006.557-7009.875 XP_010899738.2
MAXISTIYKLSIIAFIYFTNVYGTSCPVLECWFVQERPGRGGGFPAAMSQEKSLLYINTD
PEWKRPESQQRPSTDINXDRLYYITDPAATFCSSSLHPPEGSVQKPQCEINPFMPQPSSV
QWTVSLTDSAHSPIYLQADWYSASLQGLNGQLSLSSVMRAPTATKEPAVLLSVSSRTPVV
RSRLGERVVLDCGFWAEASSPLSGSGFTVEWRYQFRGDGRLVLAYDGKNDRLAETQEKGA
ELDFTALHERGNASLILQEAQVRHSGTYICTVYLPYILXQVAVELEIVEPPSLSIFPXPL
PLSVPGQVVTVQCEASGFSPLSLELHWELMETDGNVRPLGQGSVSGHRQGPDGTYSQTTR
LQLDSAKLDLGRGGEVTCVAVHPGGTRRARVTLNVIGVSAPSIEDSMAMVAVALCLYGLI
KVISWTFISSGSEDAEKKAK
>PikeUBA1 NC_025977.3:7.021.021-7.034.658 XP_019905630.1
MSGVQTTHSLRYVYTATSGIPDFPEFVTVGLVNGEPISYYDSIIRTETPRQDWMXKAVDP
EYWSRNTQLSIGTEKTFKDNIDVVNQRFNQTMGVHINQLMYGCEWDDETGVTEGFDQFGF
DGEDFISLDLMTLTWIAPKPQAVISKLRLDNNIVHSNNYLKNYFTQECIDWLKKFLDYGK
SSLMRTVPPSVSLLQKTPSSPVTCHATGFYPSEVIVTWKKDGQEQHEDVEVGETLHNDDG
TFQKSVHLTVKPEEWKNIKYQCVVQVSGINEDFIKVLTEDQIQTNCRKTNRDPTSTGLII
GVVVGVSILVIMAAVFGVVIWKNKSKKVSDGGSNSSNNSSPKP
>PikeUBA2 NC_025977.3:7.043.107-7.060.011 XP_010899737.2
MKVIILSILGIGFLPTVSAVTHSLKYFYTGVSGXINFPEFTTVGLVDNGQFIYFDSNTKT
AVPKTEWMKREGPDYWDSETQTGINLHQVFKNSIQSTMDRFNQSMSTGVHVVQMMYGCEW
DDETEATEGFDRYGYDGEDFLLLDLKTLSWIAPKSQAFITKNKFDSNKDTNLYLRKYFTQ
TCVDWLKKYVDYGKSSLMRTVPPTVSLLQKTPSSPVTCHATGFYPSRVNVTWKKNGQEQH
EDVEMGEILPNDDGTFQTSVRLNVKPEERKNNKYQCVVQVAGINEDFIKDLTEDTTSIGP
IIGVVVAGLLVIVAAVIIGVVIWKKKSKKGFVPANTSDDGSNSSNNTAPKA
>PikePSMB8F NC_025977.3:7.084.970-7.085.777 XP_019905769.1 GATF01015276.1
match
SVSAGSKLLANMVVNYRGMGLSMGTMICGCDKKGPGLYYVDDNGMRLCGNMFSTGSGNSY
AYGVMDSGYRYDLSVPEAYDLAQRAIFHATHRDAYSGGTVNMYHMKETGWVKVSQEDVGD
LYHRFNNENK
>PikePSMB8 NC_025977.3:7.095.708-7.109.763 XP_010866033.1
MALFDVSGYKSHLELRGNTLGTGLRHLNFGTTNKEFAVPAGMDPSGFLKSCSHEGQGGVS
IDLNHGTTTLAFKFRHGVIVAVDSRASAGSYIASKEANKVIEINPYLLGTMSGSAADCQY
WERLLAKECRLYKLRNKKRISVSAASKLLSNMMLGYRGMGLSMGSMIIGWDNKGPGLYYV
DDNGTRLSGQMFSTGCGNSYAYGVIDSGYREDMTVEEAYELGRRGITHATHRDAYSGGVV
NLYHMQEDGWIKVCKEDVSELIHRYRKGMF
>PikePSMB13 NC_025977.3:7.111.771-7.114.933 XP_010866031.1
MALPNVLETPPTGFNFENVFRNVALEGLLEGGRTKAPKPMKTGTTIAGVVCKDGVVLGAD
TRATSSEVVADKMCAKIHYIAPNIYCCGAGTAADTEKTTDLLSSNLTIFSMDSGRNPRVV
MAANILQDMLFRYRGQIGAHLILGGVDCTGSHLYTVGPYGDIDKVTYLAMGSGNLAALGI
LEDRFKPNMELEEAKELVRDAIHSGIMSDLGSGNNIDICVITKQGVDYLRPYQESEFKDK
RQMRYKYHPGTTPILTEKIVPLQMEVVQETVQRMDTA
>PikePSMB12 NC_025977.3:7.115.084-7.119.208 XP_010866032.1
MERHYMDSKIKGVSTGTTILAVTFNGGVIIGSDSRASMGGNYVCSKTINKLIQVHDRIFC
CIAGSLADAQAVTKTAKFQISLHSIQMESPPLVKAAASVMKELCYNNKEELQAGFITAGW
DKKKGPQVYTVALGGMLLSLPFTIGGSGSTYIYGYTDAKYKPDMSREECLQFATNALALA
MGRDNVSGGVAHLVVITEGGVEHVVIPGDKLPKFHDE
>PikePSMB9 NC_025977.3:7.121.547-7.131.354 NP_001297802.1
MLEESTESEWQSEVKTGTTIIAVEFNGGVVLGSDSRVSAGETVVNRVMNKLSLLHDKIYC
ALSGSAADAQTIAEMVNYQLDVHSIEIGEDPQVRSAATLVKNISYKYKEELSAHLIVAGW

DRRGGGQVFVTLNGLLSRQPFAVGGSGSSYVYGFVDAEYQKGMSKEDCQQFVVNTISLAM
SRDGSSGGVAYLVTIDEKGAEEKCILGNELPTFYDQ"
>PikeTAP2 NC_025977.3:7.133.087-7.143.465 XP_010866026.3 polished with TSA sequence GATF01026336.1
MKMIIKTCLLAIALGLDITIFSAPGFGASISKIGQITYTFGYIVSVWIVAVIRFVFLVSV
SVTTLESIKPIFKRWLAAHCLLVPVYETGRLILHGRSLESSCGPLGGPRLWLVCTVAAAT
AAFFWETTFPDSNGESNEKEKKQKARVLFLRVVRFCKHDSLILICAFIFLSLAVLCEMFI
PIYTGKVIDILGTKYMWNDFRTVIIFMGLCSLGSSFSAGCRGGLFMCAINSFTCRIKVQL
FGALVKQEIGFFETIKTGDVTSRLSTDAPKMGRAVALNVNVLLRTAIKTVGMLSLMMSLS
WKLTLLMLMETPITGLLQSIYDNYYQRLDKEVQDSLARANETAGETVAGIRTVRSFKTEQ
IEAGRYDNRLMDTHNIKTRRDTVRAVYLLLRRLIALVTQVAMLYFGRLFVQQGQMTTGNL
VSFILYQSDLADNIRTLIYVYGDMLNSVGAAGKVFEYLDREPHVSTKGTLQPETLTGHIC
FHNLSFSYPTRQEHKILQDFSLELKPGKLTALVGPSGGGKSTCVSLLERFYQPQQGEILL
DGQPLQSYQHHYLHRKVAMVGQEPVLFSGSIKDNIAYGLKDCSLERVQEAARRANAHGFI
SKLEKGYYTDVGERGNQLSGGEKQRIAIARALIREPQVLILDEVTSALDTESEHMVQEAL
ASCPSQTVLVIAHRLKTIERADQIILIDQGTVLEQGTHKVLMEMKGSYYKLRERIFTEDD
VPK
>PikePSMB10 NC_025977.3:7.488-404-7.493-157 NP_001291088.1
MLNTSRPCQPQSGGFSFEDSRRNAVLEGNLSELGYSSPKARKTGTTIAGIVFKDGVILGA
DTRATDDMVVADKNCMKIHYIAPNIYCCGAGVAADAEVTTQMMSSNVELHSLSTGRPPLV
VTVTRQLKQMLFRYQGHIGSSLIVGGVDVTGAHLYSAYPHGSYDKLPFLTMGSGAGPAIS
IFEDRFRPNMELEDAKQLVRDAIVAGIFCDLGSGSNVDLCIITQAGVQYLRAYDQPAQKG
KKEGQYKYKPGTTAVLTKTVTPLPLDIVDESVQLMDTQ

Esox Lucius unplaced genomic scaffold Un_scaffold0763 NW_017859271.1 deduced amino acid sequences

>PikeMHCI.3_NW71 NW_017859271.1:13.681-29.329 XP_012986808.1
MKVFILLIMGIGLLHSVSAATHSLCYVYTATSGIPDFPEFVTVGLVNREPISYYDSIIRT
ETPRQDWMANNEGSDYWESQTQISNGTEQTFKANIDILKQRFNQTGGVHVVQNMYGCEWD
DETGVTDGFDQYGYDGEDFIVFDLKRLTWIAPKPQAFINKNKWDNDRGWIEQTKNYLTQT
CIEWLKKYVDYGKSSLMRTVPPSVFLLQRTPSSPVTCHATGFYPSRVMVTWMKDGQEQYE
DVEVGETLQNDDGTFQKSVHLTVKPEEWKKNKYQCVVQVSGIKEDFIKDLTEDQIQTNTG
FVPANTSDDGSNSSNNTAPKA
>PikeMHCI.4_NW71 NW_017859271.1:69.714-85.450 XP_012986807.1
MKVIILLILGIGLLPTVSAASHSIRYVYTATSGIPDFPEFVNLGILDGMQIDYYDSNTKK
VVPKQDWMAKAVGSDYWDRETQNSIGREKSFKANIDTAKQRFNQTGGVHVAQMMFGCEWD
DETGVTRGFNQQGFDGEDFISFDLKTLTWIAPKPQAVITKNKWDNNKAGNEYWKNYLTQE
CIDWVKKYVDYEKSSLMRTVPPSVSLLQKTPSSPVTCHATGFYPSGVHVTWKKDGQDHHE
DVEMGETLQNDDGTFQKSVRLNVKPEEWKNNKYQCVVQISGTKEDFIKDLTEDELVSNWG
KTNRGVNDQTSIGPIIGVVVAGLLVIVAAVIIGVVIWKKNRKGFVPANTSDGGSSTNSAP
EA
>PikeMHCI.5_NW71 NW_017859271.1:149.722-177.971 XP_010866362.2
MKVFILLILGXGHLHTLSAAFHSLKHTCTAVSGDTHLPEFTALGLLDNVQVAYFDSNTKT
AVPKTEWIRREGPDYLNSLTNLGINAHQRLKAHLQIFKDQFNQSMSTGVHTLQVMMGCEW
DDETGVTEGYMQFGYDGEDFISFDLKTLTWITSKPQAVITKDRWDNDKADNEYWKNYLTQ
ECIDSLKNFLDYGKSSLMRTVPPSVSLLQKTPSSPVTCHATGFYPSEVIVTWKKNGQDHH
EDVEMGETLHNDDGTFQKSVHLTVKPEEWKNKKYQCVVQVSGINEVFIKVLTEDEIETNW
GKTQK"
>PikeMHCI.6_NW71 NW_017859271.1:189.068-206.991 XP_012986804.1
MYFFVLLILIIGHLHTVSAVTHSLKYFYTGVSGDINFPEFTTVGLVDNVTFIYFDSNTKT
AVPKTEWIKRVGPGYWVSQTKTCINLHQFFKNNIQVAKDRFNQSMSTGVHVLQWMYGCDW
DDETGVIQGFDQYGYDGENFISLDLKTLSWIAPKPQAFITKNKWDSDKADNEYLKHYFTL
ECIEWLKKYLQYEETSQMRTVPPSVSLLQKTPSSPVTCHATGFYPSEVNILWQKDGPFQH
EDVETGHTLPNGDGTFQKSVLLTVKPEEWKNNKYQCVVQVSGINKDFIKDLTESEIQTNR
DTSIGHIITVVVAGLLAIGAAVIGVVNRKKKSKKGFVPPNTSDDGSNSSNKSSSNV
>PikeMHCI.7_NW71 NW_017859271.1: 215.029-249.012 XP_012986801.1
MKVIVLLILGIGLPTVSTVTHSLKYFYTGVSGGINFPEFTVVGLVDNGQFIYFDSNTKTA
VPKTEWMKREGPDYWDSQTQILIGSQPVFKNNIQVAKDRFNQSMSTGVHVVQMMYGCEWD
DETGVIEGFDQHGYDGEDFISFDLKTLKWVAPTPQAFISKNKLDYNEAGNEGEKHYLTQE

CIEWLKKYVDYGKSSLMRTVPPSVSLLQKTPSSPVTCHATGFYPSGVHVAWKKDGQDHHE
DVEMGETLQNDDGTFQKSVLLTVKPEEWKNNKYQCVVQVSGINKDFIKDLTESEIQTNRE
PTSIGPIIGAVAGLLVIAVVVIGVVIWKKKSKKGFIPANTSDDGSNSSNNTAPKA
>PikePSMB8_NW71 NW_017859271.1:275.209-276.945
MAIFCVSGYNSHSQLRGEITGKGFSSVLSL*PSGFLKSYSHEGQGGGSIDLNHGTTTLAQ
FRHGVLTLTHGVIVAVDSRASAVSYIASKEANKVIEINPYLLGTMSGSAADCQYWERLLA
KECRAEKLRNKKRISVSAASKLLSNMMLGYR

Esox lucius unplaced genomic scaffold Un_scaffold1132 NW_017859580.1 deduced amino acid sequences from pike genome [5]

>PikePSMB8_NW80 XP_010866810.3 NW_017859580.1:27.821-34.344
MALFDVSGYKSHLELRGKTLGTGLRHFNFGTTNQEFAVPAGMDPSGFLKSWRHEGQGGVS
IDLNHGTTTLAFKFRHGVIVAVDSRASAGSYIASKEANKVIEINPYLLGTMSGSAADCQY
WERLLAKECRLYKLRNKKRISVSAASKLLSNMMLGYRGMRLSMGSMIIGWDNKGPGLYYV
DDNGTRLSGQMFSTGCGNSYAYGVIDSGYREDMTVEEAYELGRRGITHATHRDAYSGGVV
NLYHMQEDGWIKVCKEDVSELIHHYKKGMF
>PikePSMB13_NW80 XP_010866807.1 NW_017859580.1:21.631-26.326
MALPNVLETPPSGFNFDNVFRNVALEGLLEGGRTKAPKPMKTGTTIAGVVCKDGVVLGAD
TRATSSEVVADKMCAKIHYIAPNIYCCGAGTAADTEKTTDLLSSNLTIFSMDSGRNPRVV
MAANILQDMLFRYRGQIGAHLILGGVDCTGSHLYTVGPYGDIDKVTYLSMGSGDLAALGI
LEDRFKPNMELEEAKELVRDAIHSGIMSDLGSGNNIDICVITKQGVDYIRPYQESEFKDK
RQRRYKYHPGTTPILTEKIVPLQMEVVQETVQRMDTA
>PikePSMB12_NW80 XP_012988529.1 NW_017859580.1:16.159-21.594
MERHYMDSKIKGVSTGTTILAVTFNGGVIIGSDSRASMGGSYVCSKTINKLIQVHDRIFC
CIAGSLADAQAVTKTAKFQISLHSIQMESPPLVKAAASVMKELCYNNKEELQAGFITAGW
DKKKGPQVYTVALGGMLLSLPFTIGGSGSTYIYGYTDAKYKPDMTREECLQFATNALALA
MGRDNVSGGVAHLVVITEGGVEHVVIPGDKLPKFHDE
>PikePSMB9_NW80 XP_010866806.1 NW_017859580.1:12.295-15.497
MLEESSEPGWPSEEVKTGTTIIAVEFDGGVVLGSDSRVSAGETVVNRVMNKLSLLHDKIY
CALSGSAADAQTIAEMVNYQLDVHSIEIGEDPQVRSAATLVKNISYKYKEELSAHLIVAG
WDKRGGGQVFVTLNGLLSRQPFAVGGSGSSYVYGFVDAEYQKGMSKEDCQQFVVNTISLA
MSRDGSSGGVAYLVTIDEKGAEEKCILGNELPTFYDQ
>PikeTAP2_NW80 XP_019900791.1 NW_017859580.1:4.666-11.424
MIKACVLAFALISIDIILTTFSAPGSGSSKTGPITCDAFGNLVNVWIVAVIRLVLLVTVS
VSTLGSIKPIFKRWLTVHCLLAPVYVTGRLILHGSSLESAYGPLGGPRLWLVCTVAAAAA
ALFWETTFPDSNGESNGKEKKEKKQKALVLFMKVLRFYKPDILFLTGAFIFLSLAVLCQM
CIPFYTGKVIDILGAQYKWKDFRTAIIFMGFFSLGSSFCAGCRGGLFMCAINSFTCRMKV
QLFGTLVKQEIGFFETNKLGDITSRLSTDTTLMGRAVALNVNVLLRTAIKTVGMLSLMTS
LSWKLTLLMLMETPITGLLQSIYDSYYQRLAKEVQDSLARANETAGETVAGIRTVRSFKT
EQSEAGRYDDRLMDTHNIKSRRDTVRAVYLLLRRLIALVMQVAMLYFGRLFIQQGQMTTG
NLVSFILYQSDLADNIRTLIYIFGDMLNSVGAAGKVFEYLEREPQVSTNGTLQPETLTGH
IFFHNLSFSYPTHKERKILQDFSLELKPGKLTALVGPSGGGKSTCVSLLERFYQPQQGEI
LLDEQPLQNYQHHYLHRKVAMVGQEPVLFSGSIKDNXAYGLKECSLERVQEAAXRANAQG
FIRXLEKGYYTDVGERGNQLSGGEKQRIAIARALIREPQVLILDEVTSALDTESEHMIQE
ALASCPSQTVLVIAHRLKTIERADQIILIDQGTVLEQGTHKVLMEMKGSYYKLRERLFTV
DDDPK

Deduced amino acid sequences from Spotted gar (*Lepisosteus oculatus*)

>Spotted gar TAPBP GFIM01016833 No genomic match Lepisosteus oculatus
MSDTATIYRLTLLALWGLAAVCGSRCPVLECWYVQEKAGQGGGFPAAMTQEKSLLYVRTE
PDPVGLQQNPPSDIDPKRIYYVTDAGASLCSPSLRAAEGAVEKPQCEVSPFLPQPALVRW
AAPLTAAGSSPAHLQADWLSAALRGLQGQLVLSSLLRAPSASDQASVVLSVFSRRLVVRT
RLGAPALLDCGFWARPGSPLWGAGFAVEWRYQFRGEGRLVLAYDGRGDRLEEAEPGAELD
AAEAHRTGNVSLRLGGAEVRHAGTYICTVYLPYLLAQVAMELEVVEPPALLLSPSPLWAL
PGHSAPVQCEASGFFPLALELQWEFLPAGGAGEALALEGATVSGHRQGRAGTFTQTSRLE
LDLGRLRLEHGGQLVCVAKHEGGTRRPGVTLNIAGVGGPTVEDSMAMVAVALLLYGLIKI

VSWTLKTTDSSVPEEPEKKQE*
>Spotted gar TAPBPR ENSLOCG00000006844 LG26:11.560.611-11.567.557
XP_015193320.1 Lepisosteus oculatus
MRLAFGVLVLASHLMSSVSGSVADVVLACSLVEEGGGMGGRLAGAGALFTRDEATLVLRD
LPVTADESLDTVTPFAPPAPDPENLIFEATVTSLEIPEADSLLHADCNEQEVTCEISRYF
PRGPEGTEPSPEDAFFIGSLQLEGGGVSLTLVLRTQPIPAEQEEDGGRPLRQSKLDLPLS
PSGTILNEVVFVVFTRLQSLTAPLGGSALIDCGYKEATPSQEVALEWRLQHKGHGRRILQ
LRAGREGEEPTVHPEREGASVEPGLVLEEGNVSLTLTNVKVADEGTYICTVGSGVYQAQQ
VIQLHITQTPSVTLTPNQLVFQDDTPQRVICHCDHYYPLDVQVEWFSLSPSASEPVPISN
GVYFSSHRQHSDGTYSLSAYISVSPSEDLTGATFSCIVSHHSLSEPITASITVSAPEESQ
LWSMVGGVLSFVLFLFGLFMLLR
>Spotted gar TAPBPL LG22: 10.745.093-10.749.329 ENSLOCG00000006827
GFIM01040944.1 Lepisosteus oculatus
MITTAVLLCVLICSGLRCVRSESLAEAGLLACELVEERVSSGLSGAPETQFVNRKAALLL
ARPGREADTAQRLQEAQRAGVVTFLATGSAVDVTRHVPGGTEQLQCEIRRYSTGGIQVPW
KGTAGPDSAWFTCTLRHSANEFIVTAFLLYPSPAPQQPIGDADTLNTSVAMVVLTRTPTV
QAGLLQDARLDCGFAVDHAQPDVTVEWRFRHRGEQRKLFSYSRRSGRAETEVPGATAAAR
DVSRGDASLRLSAVKVKSEGTYVCSVYVPPLYGSHDVQLQIMESPRVSLSVATALALQEE
EEQKVVCEITGYYPLDVQVDWLHEQGGGGRLPQVIKNILFTSHRHHADGTYSISAFFILR
PSLADHDARYTCRVSHVSLRTPIRKSFTLTVTERLRIGSILFWIFVAFIVMMLLLLLVIL
LPYLHSARREAKKKKPY*
>Spotted gar ERp57 LG3: 45.308.360-45.321.947 ENSLOCG00000014242
MRRDNMIRHGMNGWQTVPCTSLVLYLGVPRTSPCFLAVAVSASDVLEFTDDDFESRIADH
DLVLVEFFAPWCGHCKRLAPEYESAATRLKGIVPLAKVDCTANSNVCNKFGVSGYPTLKI
FRGGEESGAYDGPRTADGIVSHLKKQAGPASVEIKTVEEFEKFIGDRDASVIGFFAEGGS
TTQAEFLKSASALRESYRFAHTNSEELLQKHGIDGEGIVLFRPPRLSNKFEESSVKYSED
KYTSGKIKKFIQDNILGICPHMTEDNKDQLKGKDLLVAYFEVDYEKNPKGTNYWRNRVMK
VAKSFLDQGKKLSFAVANKNSFSHEISEFGLDSSSGELPVVGIRTAKGDKYVMQEEFSRD
GKALERFLQDYFDGKLKRYLKSEPVPESNDGPVKVLVAENFEEIVNDDSKDVLIEFYAPW
CGHCKSLEPKYKELGEKLSDDPNIVIAKMDATANDVPSPYEVRGFPTIYFSPAGKKQSPK
KYEGGREVSDFISYLKKEASNPLVIQEEEKKSKKKKSELSCLLERMKEQCLVDVN
>Spotted gar ERp57L LG24:5.612.549-5.621.013 FGENESH predicted
MARLSVRLLCPAALLALAALGAVAVAGGDVLELGDADFDGAVAQHETVLVEFFAPWCGHC
QKLAPEYEEAATRLKGAVPLAKVDCTAHSETCSRFGVNGYPTLKIFRNGQESSAYDGPRT
ADGIVSYMKKQAGPSSVELRTEEDLDSFVSHFDGSVVGFFLGPESADLAEFLRAASSMRD
SYRFAHTSAESLRQRHGVQGEAVLLFRAPQLASKFEESVVRHRGAVRPDALRRFIRENIF
GMCPHLTHENREQLMRQDLLTAFYELDYLRNPKGSNYWRNRVMMVASRFAGRGLSFAVAD
RRDFPDELEEFGLEGAEGELPVAAVRTVLGQKYVMREEFTRDGKALERFLEDYFAGRLKR
YLKSEPVPESNDGPVKVVVAETFDEIVNDASKDVLIEFYAPWCGHCKNLEPRYTELGQQL
SGDPNIVIAKMDATANDVPEGYDIQGFPTIYFAPVGQKAAPRRYEHAFHFHCISRWLKTR
QVCPL
>Spotted gar PDIA4 LG9: 50.559.604-50.567.699 ENSLOCG00000013319
MKVKKAFLLLALLGLAHLALLASCEESVKPESIRSEMTQRAVSHRLYFSSTTRDFLWVRK
TGLVILKDSNFDSFIADKDTVLVEFYAPWCGHCKQFAPEYEKIAETLKEHDPPIPVAKVD
ATAESGLGSRFEVSGYPTIKILKRGQPVDYDGERTEQAIVSRVKEVARPDWTPPPEATLV
LTKDNFDNVVNEADIILVEFYAPWCGHCKRLAPEYEKAAKELSARTPPVPLAKVDATAEA
ELATRFDVSGYPTLKIFRKGKAFEYNGPREKYGIVDYMIEQAGPPSKQVQALKQVQESVK
DGDDVFIIGVFASDEDAAYEIYQEACNNLRDDYKFHHTFSDEIAKFLKASPGQVIMMQPE
KFQSKYEPKSSTFQIKDSTTASEVKDFFTQHALPLVGHRKQSNDAKRYTKRPLVVVYYGV
DFSFDYRVATQFWRSKVLEVAQDFPEYTFAIADEEDYAEELKALGLAESGEEVNAGILGE
GGKKYAMEPEEFDSDALRDFVTAFKKGKLKPIIKSQPVPKNNKGPVKTVVGKTFEEIVLD
PKKDVLIEFYAPWCGHCKKLEPEYLELGKKYKNEKGLVIAKMDATANDVLHESYKAEGFP
TIYFAPSGDKQHPIKFEDGKRDLEGLSKFLEQHATKLSQKRDEL

Deduced TAPBP-like amino acid sequences from other species

>Frog TAPBPL XP_018100952.1 tapasin-related protein-like [*Xenopus laevis*]
MDSRLYLIIILNLLIVCNDIQSFEDLTSRKSRKLSCMYVDMRNKTLLGYNDIMKQKAFLVL
SSRKQLSKEYFDSEGITFILKDSPVNTMKFLKEHTLDLVCEVNPYFTDNTQIIWPGIHVI

ENMEEAWFVSRFKHPNDKFQFTLFFTHISQKSLEEQDGSQLQAFPNAEIRYVLPVFMLST
QTPHVQTRLQEDALLNCDFSIDHHADVNIVWKLLKKGGQEMKLLTYKGSEKSVVYHVKGI
AMYVDEVPKGSASLVVKNVDLEKEGLYTCSVSVNSLFGDQVIHLEVVEPPIVNLNVKSLL
LTEGQEQKLVCEASKYYPLDVNIEWLREGRDQTLLPTVLKNVLYSSHKHNNDGTYSLSSF
FIFTASQLDNGAVLTCRVEHMSLKHPIRKSVKVRVEESPSYQEFFLVILIIFLSVVLLTL
ILHLNRGRSSSKKETMTDKLAAFDANVSEMEEFAQSECFYSF
>Chicken TAPBPL Merged ESTs BU342879.1+ BU369515.1+ BX257449.3 (*Gallus gallus*)
MSGDVLSLMAQRTLELPPCGHGSPQDGGAQCSNHKDPKQHCFCIPRLRRILQLPCMFEMK
KNIPLSSKTETVLLNIRLLLPVAGAGGAQRLQTESLPAGNLPSFIVQEPSVNILQHTDED
INMLDCRISPYFTANTQILWPGRKVKAHGLDTWFTCTIEHAAGKYTATAFLVQGHEDREH
QTPGQLHQGIAEQTRVSAVLAARTRLPRVRTALGKDVVLDCAFAADPRAAVAVRWALRKK
GRHERYIATSGGRAEMFPQEPRGNASLLIRRVELSDEGTYICTVEAAALVLEQAIQLQIT
EKPTVTVNVNSLSLVEGEQQKLVCDVRNYYPADIHVQWLREPQSAGQLPDTVPNVLTSSH
LRSSNGTYSFSRFFLLTATLRDNGHTYTCRVEHSSLQAPIRRSITVAVREATSTTWLLLL
LLLGLTGCLVASLHHLHQVRSTTKPKPY
>Turtle TAPBPL XP_005298961.1 tapasin-related protein-like [*Chrysemys picta bellii*]
MNFRIYTAVYCFLCTGILKHHNGVATAGSTFRQSRRLSCLFETTETIPLLSEFTYIRQNA
WLLLSGAERPHQENPPAADTLTFIVQESSSLDILQHVDEDTDKLECKINRYFTDNTQILW
PGLQTQLHKLDSWFTGTIKHLADKFTVTVFLVQSSASKEAENRVPEDVSQGNAERFYLSG
VFLVRTGPSLIQSGLNKDVLLSCAFSVDHHTDVTIKWVLQQKGGHKKLIFAYNGFTRQVE
HEDKRAEMFLAELPKGNASLLLRTVGVRDAGTYSCSVLVSSLLWAQNIQLEIVEKPTVTL
NADVLSLVEGDEHKLVCDISHFYPHDADAQWLQEPMEQGMLPHVVTHVVSSSHRHNSDGT
YSFSSYFLLKASLRDDGRRYTCRVEHQSLKHPIKKSLTVKVTESTSATWFLVILLVLLAG
CLVVTLCYLYRGMGPNKPKPY
>Kiwi TAPBPL XP_013817376.1 tapasin-related protein-like [*Apteryx australis mantelli*]
MNYRTYLVAGCFLCIGIAKNLNGIATAGSSFRKSRQLACVFETSKIIPLTKELEIIRLNV
RLLLSGTESEEAQRSHSENHPQDNIPSFFVKESSLDILQHANEDINTLDCRISAYFTANT
QIIWPGREIRASSLDSWFICTIKHTAEKYMTTAFLVQTHKENENHSQGQLSPGIAEQSHV
SAVFLVHTRPSVVRSALSKDVLFDCAFSIDHQADVTIQWVLHRKGGHKKLMFTYNGSSKQ
VEHMADRAEMFLEEIPKGNASLLLRNVEMRDEGTYSCSVSVSSLTGEQAIQLQIEEKPTV
IVNVNSLSLVEGEQYKLICDIRNYYPLDAQAQWLRELKGSRKVPDVVKNVLSSNHRQSSN
GTYSFSRYFLLTASLEDNEHTYTCRVDHQSLQAPIRRSVIVQVRGTSIIWVLLLLLGLSV
CLAVVLHYFYKVKSTAKPKPY
>Opossum TAPBPL XP_007485846.1: tapasin-related protein-like [*Monodelphis domestica*]
MRWITLEGTRSPGNVLGMLGRGMLALRLLSPGLLWILGWHMVESGMTLGKAGQLPCLLEV
ALDPSETLNGERKWQHIQLVLSGSESQSLSHQGNSATFLLQDRAGVLLPHMDEDVDKLEC
KVSRYFTANTQILWPGLPPRQAQLPTWHLVTISHTDAQFKASTFCFQSAESAQGTGPAIF
SGVFSLYTKASRVQVELYGTVLLACSFVMDHAPEALDVRWALRQKGGKQREILKYDGKQR
QVTHLHEQVEAFPSEIPGGDASIRLNNVAVNDQGDYFCSVSTAGLYWELSIEVAVVEAPK
VRFSPQTPILTLEEGEEEKLVCDVRHYFPLEAHVFWLRERLEGRMMPEGVKNVLFSSHRK
NGDETFSFSSYYLLKASLQDDGFRYTCLVEHEGLRFPIRKSIIIRVTEKSGSSWGLLIIL
IILMVVIFVLLRYLHQVKSMNKTKPY

Zebrafish gene sequences

Zebrafish (*Danio rerio*) Haplotype 19D amino acid gene sequences from McConnell et al.[6].

>ZebrafishUGA Q6PH40 NP_956879.1 Major histocompatibility complex class I UXA2 gene
MQQVLLFLLGAHLAYAGTHSLRYFYTAVSGDIDFPEFTMVGLVDGGQFIYFDSKKMEAVP
KTEWIRQNEGADYWDINTQRLIATHQAFKNNIQVAKERFNQSQGVHTFQVMYGCELEDDG
STRGYWQYGYDGEDFLSLDKSTLTWTATKPQAVITKNKWDADNADRQYTKSYLENECIEW
VKKYVDYGKDTLERKDAPEVFMLQKDPSSPVVCQATGFYPSNIMMTWQKNKEEHFEDVDV
GATLTNADGTFQKTVTLSVKPEEWKNNKEAYRCVVQHVGAKNDVIVTVKDIRSNEGSDNT

IAIIVGCVAAVALLAVIAGLIYWRRSNGYGKASSKDTDSEQSDPRAVEVK
>ZebrafishPSMBF_19D NP_001017791.1
MALMDVCLSSELKQSCFTANAWQSEPTNCGPSHYTFGLNANELAVPTGMDPEQFLGPLTE
GDSDIKIEFLHGTTTLAFKFQHGVMVAVDSRASAGSYIDTKDFKKVIEINPYLLGTMSGN
AADCVYWERRLAKECRIYKLRNKTRISVAAASKLLANMVAEYRGMGLSMGTMVCGWDQRG
PGLYYVSSSGTRLAGDMFSTGSGCNYAYGVMDSGHRWDLTVPEAYDLAERAIYHATHRDA
YSGGVVNMYHMQKEGWIKVSQNDVGDLHHKYVAERKK
>ZebrafishPSMB9b_19D NP_571753.1
MQNGGEHEGTNGGVGMGTTIIAVEFDGGVVVGSDSRVSAGASVVNRVMNKLSPLHDKIYC
ALSGSAADAQTIAEIVNYQLDVHSIEVDEDPLVCSAATLVKNISYKYKEELSAHLIVAGW
DRREGGQVYATLSGLLTRQPFAVGGSGSFYIYGFVDAEYRAGMTKKECQEFVINSLSLAM
GRDGSSGGVAYLVTIDSESVEEKCILGNQLPTFYDPDTVEPSKAVKV
>ZebrafishPSMB13b_19D
MFATVLKTPEPGFCFENSLRNEALDSLVKGKKPKALKTGTTIAGVVFKDGVVLGADTRAT
SNKVVADKMCEKIHYLAPNIYCCGAGTAADTQKITELISSNLTIFSMNSGRNPRVIMAVS
VLQDMLFRYHGQIGANLLLGGVDCTGGHLYDIGPYGDMDKVPFLAMGSGNLPAMGILEDG
FKSNMDLESAKHLVRDAIYAGVMNDLGSGHNIDLCVITRDGVDYIRPYQESQHIGKREGQ
YKYKPGTTAVLTEKLHHIEMDLIEESVQKMETS
>ZebrafishTAP2d_19D
MELKMQHAKALLVDLAMSCVLHYSTNAFIRINSNVFLLAQLWLVSGVKWIGLRCFLRGSW
REAAVQRCVAVSCLLCPMYESGQTLLINTQPENWSGCLSCPGKTIISAAATVLACLFWEV
SFSDTNRKAESSESVEKKEKNRALFMRVVRYSKPDVALLSGAFVFLSLAVICDMCIPFYT
GLVIDILGEHYQPNSFMSAIFLMGLLSLGSSLSSGLRGGLFMCTLSRLNKRVRLMLFNAL
VKQEIGFFEDKKTGDLTSRLSVDTKLMSQSVAMNVNILLRSLIKSVGILYLMLSLSWKLT
LLTFIEAPLIAIAQKIYNTHYEQLSKDVQDSVARANETAGEAVAGVRTVRSFCMEPSEAG
RYDERLTDTHNLKTRRDTVRAVYLLVRRLKSLGMQVLMLYCGRQLIKSGAMSTGNLVSFI
LYQGDLGSYIRTLVYMYSDMLNSVGAAAKVFEYLDRKPLVDTDGSLHPKTLAGQVHFKNL
TFFYPSRPDQAALKDFSLELKPGQMTALVGMSGGGKSTCVSLLERFYQPQQGQILLDGQP
LQKYQHKYLHQKVAMVGQDPVLFSGSVRDNIAYGLTDYDQKKVEDAAKEANAHDFICRLE
KTYDTDVGERGCLLSAGQKQRIAIARALIRQPQILILDEVSSSLDTESEKMVQDALARRP
NQTLLVIAHRLKTIERADQIIVIDKGEVLETGTHQELMERKGNYYKLRERLFSEEKEADE
KEIKQG
>ZebrafishTap2e_19D
METADSRHLKTIFRLLSLSRPDLLPICGAFLFLVAAVIGDMFMPAFTGKIIDALNSTFDH
GTFTSAIFFMGLTSLGGSFSAGCRGGLFMLTISRLTKRVRERLFSCFVKQDIAFFEKTTK
GDLTTCLSSDASLMSRSLAANVNILLRSLIMTIGIYCFMIQLCWPLALLSAFESPITITA
EKIYNKYYQGLVSSVKKSIVKSNQVAGESVYNIRAVRSQGAETVEQRLYDTSLEETHYFK
IHRDSVRATYLLFMRLFQLCMRVLILWYGHQMITSGQMTPGNLVSFILYQMEIGGHVQTL
VELHIDLIESLESADKVFEFMDHKPSVLSGDLAPDQLKGHVMFKNISFSYSSCPDKKVLE
NVSFEMKPGTITALVGVSGGGKSTCVALLKHLYEPQSGDIFLDGRPLKEYDPKYFHQKVA
VVSQEPELFARSIQKNITYGLDSYSEDMVQTAVKQANINSFVLSLEDGYNTEVGERGGHL
SVGEKQRIAIARALIRQPQVLILDEVTSSLDTENEKMIQNALACFATQTRLIVAHRLKTI
EKADQIIVIDKGSVSEKGTHVELMKKKGIYYKLRESRFNEDSLET

Zebrafish (*Danio rerio*) haplotype 19B amino acid gene sequences from McConnell et al.[6].

>Zebrafish PSMB8a_19b NP_571467.3 D. rerio
MALLDVSGYKYNSASQFGFKQTLLDRSNHYSFGTKCQEFAVPVGVDPSKFLKSCSCEDGV
CIDLNHGTTTLAFKFRHGVIVAVDSRASAGKYIASKEANKVIEINPYLLGTMSGSAADCQ
YWERLLAKECRLYKLRNKQRISVSAASKLLSNMMLGYRGMGLSMGSMICGWDKQGPGLYY
VDDNGTRLSGRMFSTGCGNSYAYGVVDSGYREDMTVEEAYELGRRGIAHATHRDAYSGGV
VNLYHMQEDGWIKVCKEDVSELIHRYKKGMF
>Zebrafish PSMB9a_(19b) NP_571466.1 D. rerio
MSEELFPEPGWLSEEVKTGTTIIAVTFDGGVVIGSDSRVSAGESVVNRVMNKLSPLHDKI
YCALSGSAADAQTIAEIVNYQLDVHSIEVEDDPLVCSAATLVKNISYKYKEELSAHLIVA
GWDKKGGGQVYATLSGLLTKQPFAIGGSGSFYINGFVDAEYKKNMTKRECQEFVVNALTL
AMGRDGSSGGVAYVVTIDKDGTEEKCVLGNELPKFFDE
>Zebrafish PSMB12_(19b) NP_571751.1 D. rerio
MDRHHPYSQVNGVSTGTTILAVKFNGGVIIGSDSRASMGESYVSSKTINKLIQVHDRIFC

CIAGSLADAQAVTKMAKFQLSFHSIQMESPPLVKAAASIMRELCYSNKEELRAGFITAGW
DRKKGPQIYVVSLGGMLLSQPFTIGGSGSTYIYGYVDAKFKPDMTLEEATQFSTNALALA
MGRDNVSGGVVHLVVITEAGVKHIVVPGDELPKFHDE
>Zebrafish PSMB13a_(19b) NP_571752.1 D. rerio
MALTSHVLEPSLCGFNFENATRNIVLENGAEEGKIKPPKALKTGTTIAGVVFKDGVVLGA
DTRATSDEVVADKMCAKIHYIAPNIYCCGAGTAADTEKTTDMLSSNLTIFSMNSGRNPRV
VMAVNIIQDMLFRYRGMIGANLILGGVDCTGSHLYTVGPYGSMDKVPYLAMGSGNLAAMG
ILEDRFKVNMDLEQAKALVSDAIQAGIMCDLGSGNNIDLCVITKEGVDYIRPHKESPYNY
KRQAKYKYKSGTTPILTKTVNKLELDLVHETVQMMETAASS
>ZebrafishTAP2a_(19b) NP_001006594.1 D. rerio
MRKVLVFACMLCFDILIVLILDLAETLLKSPTSSLDYNLFRQWAESGLRCTLLYAGSTLS
KDAADPLIRRWITVHCFIGSVYETGRLAMFNNYRLDKFTVWLVGTVAAALACLFWEITLP
DTNEESNGKERKQKARVLFIRVIRLYRPDYILLFGAFVFLALAVLCEMFIPLYTGEVIDI
LGSHYQWDNFRSAIIFMGLFSLGSSFSAGCRGGLFMCAINSFTCRVKVQLFGSLIRQDIG
FFETIKTGDITSRLSTDTTLMGRAVALNVNVLLRTLVKTLGMLYLMVSLSWKLTLLMLME
TPLTGLLQNIYDTHYQKLSKEVQDSMAQANDAAGEAVSGIRTVKSFKTELGEAHRYDGRL
METHNLKTRRDTVRAIYLLIRRMTELGMKVAMLYYGRLFIQYGQMSTGNLVSFILYQQDL
GDNIRTLIYIFGDMLNSVGAAGKVFEYQDRKSEVSIDGNLMPKDLKGHVKFQKLTFSYPR
RPDHNVLKDFSLELKPGQITALVGMSGGGKSTCVSLLERFYQPQQGTILLDGKPLQDYQH
KYLHSKVAMVGQEPVLFSGTVRDNIAYGLQGCSMERVKEAASKANAHSFISKLEKGYDTD
VGERGNLLSGGEKQRIAIARALIREPQVLILDEVTSSLDTESEQMVQQALSCCPTQTLLV
IAHRLKTIERADQIVVIDSGELVEKGTHEELMEKKGSYYKLRERLFSDDKTTKQEKEKSD
TVKTQ

Other zebrafish deduced amino acid sequences from McConnell et al.[6]

>Zebrafish TAPBP_CG2 (Danio_rerio) TSA GDQH01003123.1
MSDISTVFKISVIAFTLFHGFYGSACPVLECWFVQEKPGHGGGLSTPMSQEKSLMFIRTE
AYSEETKAELHPPADISSSRVYYVTDPAGTFCSAALNPPKGSVNKPKCEINPFMPHASMV
RWASALTDSAQSPVYLQADWFSVAAQGLDEQLTLSNIMRAPSASKEPEVILSVSSKTPVV
RCRLGEPVLLDCGFWIDPSSPLHGSGFSIEWRYQFRGEGRLVLAYDGKNDRFAETSESGA
EMDITGLYQTGNASLILEESQVRHSGTYICTVYLPHLLAQVAVDLEIVEPPSLSILPSPL
PLLVPGQVLSVQCEASGFAPHTLDLGWEFTGADGKSLSLGQGSVTGHRRASDGTFSQSSR
LELDSTKLRLARGGEISCVAKHDGGTRRASAALNVIGVAAPSIEDSMAMVAVALLLYGMI
KFLSWTFSSSDSGDSELNDKKEK*
>Zebrafish Psmb7_21 NP_001039029.2 ENSDARG00000037962 Chr.21: 8.249.235-8.259.556
MATVSVCQYQPGGFSFENCRRNALLEADITKLGFSSPAARKTGTTICGIVYKDGVVLGAD
TRATEGMIVADKNCSKIHYISPNIYCCGAGTAADTEMTTQIISSNLELHSLSTGRLPRVA
TANRMLKQMLFRYQGYIGAALVLGGVDCTGPHLYSIYPHGSTDKLPYVTMGSGSLAAMAV
FEDRYRPDMEEEDAKSLVRDAIAAGIFNDLGSGSNIDVCVITKGKVDYLRPHDIANKKGV
RTGSYRYKHGTTGVLSKAVTPLNLDMVEESVQTMDTS
>Zebrafish PSMB7/10_4 Chr.4 NP_001315481.1 Chromosome 4: 75.630.955-75.639.988
MLNTSTKTLTGGFSFENTRRNAVLEANLSEKGYSAPNARKTGTTIAGLVFKDGVILGADT
RATDDMVVADKNCMKIHYIAPNIYCCGAGVAADAEVTTQMMSSNVELHSLSTGRPPLVAM
VTRQLKQMLFRYQGHIGSSLIVGGVDVNGAQLYSVYPHGSYDKLPFLTMGSGAASAISVF
EDRYKPNMELEEAKQLVRDAITAGIFCDLGSGSNVDLCVITDKKVDYLRTYDQPVHKNQR
GGTYRYKPGTTAVLSKTVTPLTLDVVDESVHVMDTE

Previously published Medaka (*Oryzias latipes*)sequences

Sequences originate from HdrR[7], HN1 [8], cab [9].

>HdrR_PSMB9 BA000027:216010-218097 BAB83845.1
MLGEAEPQWISEEVKTGTTIIAIEFNGGVVLGSDSRVSAGDSVVNRVMNKLSPLHDKIYC
ALSGSAADAQTIAEMVNYQLDVHSLEIDEDPQVRSAATLVKNISYKYKEELSAHLIVAGW
DRRDGGQVFATLGGLLTRQPFAIGGSGSSYVYGFVDAEYRRGMTKEECQKFVVNTLALAM
NRDGSSGGVAYIVTIDEHSTDEKVILGNDLPTFFDQ
>HdrR_PSMB12 BA000027:222454-226075 BAB83846.1

MEKHFTDSRVKGVSTGTTILAAVFDGGVVIGSDSRASIGGEYVSSKTINKVIQVHDRIFC
CMAGSLADAQAVTKTAKFQLSFHSIQMESPPLVISAASVLKQLCYNNKEELQAGFITAGW
DKKKGPQVYVVSLGGMMISQPVTIGGSGSTYIYGYVDAKYKVNMTREECLQFATNALALA
MGRDNVSGGVANLVVITKEGVEHIVIPGDKLPRFSDE
>HdrR_PSMB13 BA000027:226185-228725 BAB83847.2
MALSNVLDSPAAGFNFDNAARNAAFEGLFEGGQTPKPLKTGTTIAGVVFKDGVVLGADTR
ATSSEVVADKMCAKIHYISPNIYCCGAGTAADTEKTTDLLSSNLTVFSLNSGRNPRVVMA
VNILQDMLYRYHGQIGANLILGGVDCTGNHLYTVGPYGSVNKVPYLAMGSGDLAALGILE
DRFKHDLELEKAKELVRDAIHAGIMSDLGSGNNIDICVITKQGVDYIRPFQESEYKETRK
PKYKYRPGTTPVLTKKVVPLKLEVVEEIQQQMDTA
>HdrR_PSMB8A BA000027:230073-234656 BAB83848.1
MALAAVCGGQSSSEHFGQLFSGKQARLFDRPNHFSFGTKIQEFAVPVGNEPSGFLRSCNR
EEGVRIDLNHGTTTLAFKFRHGVIVAVDSRASAGNYLASNDVNKVIEINPYLLGTMSGSA
ADCQYWERLLAKECRLYRLRNNHRISVAAASKLLCNMMLGYRGMGLSVGSMICGWDKEGP
GLYYVDDNGTRLSGRMFSTGCGNSYAYGVVDSGYKEDMTVEEAYELGCRGIAHATHRDAY
SGGSVNMYHMREDGWIKVCKEDVSELIHRYREGMF
>HN1_PSMB9 AB183488:235856-237954 BAD93261.1
MLGEEAEPQWISEEVKTGTTIIAIEFNGGVVLGSDSRVSAGDSVVNRVMNKLSPLHDKIY
CALSGSAADAQTIAEMVNYQLDVHSLEIDEDPQVRSAATLVKNISYKYKEELSAHLIVAG
WDRRDGGQVFATLGGLLTRQPFAIGGSGSSYVYGFVDAEYRRGMTKEECQKFVVNTLALA
MNRDGSSGGVAYIVTIDEHSTDEKVILGNDLPTFFDQ
>HN1_PSMB12 AB183488:241217..245420 BAD93262.1
MEKHFTDSRVKGVSTGTTILAAVFDRGVV-SDSRASIGGEYVSSKTINKVIQVHDRIFC
CMAGSLADAQAVTKTAKFQLSFHSIQMESPPLVISAASVLKQLCYNNKEELQAGFITAGW
DKKKGPQVYVVSLGGMMISQPVTIGGSGSTYIYGYVDAKYKVNMTREECLQFATNALALA
MGRDNVSGGVANLVVITEAGVEHIVIPGDKLPRFNDE
>HN1_PSMB13 AB183488:245530-251792 BAD93263.1
MALSNVLDSPAAGFNFDNAARNAAFEGLFEGGQTPKPLKTGTTIAGVVFKDGVVLGADTR
ATSSEVVADKMCAKIHYISPNIYCCGAGTAADTEKTTDLLSSNLTVFSLNSGRNPRVVMA
VNILQDMLYRYHGQIGANLILGGVDCTGNHLYTVGPYGSVNKVPYLAMGSGDLAALGILE
DGFQHDLELEKAKKLVRDAVHSGIMNDLGSGNNIDICVITAEGVDYIRPFQESEFKDRRE
IKYKYRQGTTPVLTKKITPLKMEVVQETTQKMETV
>HN1_PSMB8A AB183488:253199-259873 BAD93264.1
MALANVCGLKNYSEQSGQMFATRRLIDRPNHYSFGTKIQEFAVPVGEEPVGFLKSCNTEG
GARFELHHGTTTLSFKFKHGVIVAVDSRASTGSYIATCEYNKVIEINPYLLGTMSGSAAD
CKYWERLLAKECRLYRLRNNHRISVAAASKLLCNMMLGYRGMGLSVGSMICGWDKEGPGI
YYVDDNGNRLSGRMFSTGSGSNYAYGVLDSGYKEDMTVEEAYELGRRGIVHATHRDSYSG
GVVNMYHIQEDGWIKVCKDDVSELLHHYKKGMF
>cab_PSMB9 AB450971:56863-59061 BAJ07258.1
MLGEEAEPQWISEEVKTGTTIIAIEFNGGVVLGSDSRVSAGDSVVNRVMNKLSPLHDKIY
CALSGSAADAQTIAEMVNYQLDVHSLEIDEDPQVRSAATLVKNISYKYKEELSAHLIVAG
WDRRDGGQVFATLGGLLTRQPFAIGGSGSSYVYGFVDAEYRRGMTKEECQKFVVNTLALA
MNRDGSSGGVAYIVTIDEHSTDEKVILGNDLPTFFDQ
>cab_PSMB12 AB450971:63356-67035 BAJ07259.1
MEKHFTDSRVKGVSTGTTILAAVFDGGVVIGSDSRASIGGEYVSSKTINKVIQVHDRIFC
CMAGSLADAQAVTKTAKFQLSFHSIQMESPPLVISAASVLKQLCYNNKEELQAGFITAGW
DKKKGPQVYVVSLGGMLISQPVTIGGSGSTYIYGYVDAKYKVNMTREECLQFATNALALA
MGRDNVSGGVANLVVITEEGVEHIVIPGDKLPRFSDE
>cab_PSMB13 AB450971:67055-69667 BAJ07260.1
MALSNVLDSPAAGFNFDNAARNAAFEGLFEGGQTPKPLKTGTTIAGVVFKDGVVLGADTR
ATSSEVVADKMCAKIHYISPNIYCCGAGTAADTEKTTDLLSSNLTVFSLNSGRNPRVVMA
VNILQDMLYRYHGQIGANLILGGVDCTGNHLYTVGPYGSVNKVPYLAMGSGDLAALGILE
DRFKHDLELEKAKELVRDAIHAGIMSDLGSGNNIDICVITKQGVDYIRPFQESEYKETRK
PKYKYRPGTTPVLTKKVVPLKLEVVEEIQQQMDTA
>cabPSMB8 AB450971:70934-75613 BAJ07261.1
MALAAVCGGQSSSEHFGQLFSGKQARLFDRPNHFSFGTKIQEFAVPVGNEPSGFLRSCNR
EEGVRIDLNHGTTTLAFKFRHGVIVAVDSRASAGNYLASNDVNKVIEINPYLLGTMSGSA
ADCQYWERLLAKECRLYRLRNNHRISVAAASKLLCNMMLGYRGMGLSVGSMICGWDKEGP
GLYYVDDNGTRLSGRMFSTGCGNSYAYGVVDSGYKEDMTVEEAYELGCRGIAHATHRDAY
SGGSVNMYHMREDGWIKVCKEDVSELIHRYREGMF

References

1. Lukacs MF, Harstad H, Bakke HG, Beetz-Sargent M, McKinnel L, Lubieniecki KP, Koop BF, Grimholt U: **Comprehensive analysis of MHC class I genes from the U-, S-, and Z-lineages in Atlantic salmon**. *BMC genomics* 2010, **11**:154.
2. Lukacs MF, Harstad H, Grimholt U, Beetz-Sargent M, Cooper GA, Reid L, Bakke HG, Phillips RB, Miller KM, Davidson WS *et al*: **Genomic organization of duplicated major histocompatibility complex class I regions in Atlantic salmon (*Salmo salar*)**. *BMC genomics* 2007, **8**:251.
3. Lien S, Koop BF, Sandve SR, Miller JR, Kent MP, Nome T, Hvidsten TR, Leong JS, Minkley DR, Zimin A *et al*: **The Atlantic salmon genome provides insights into rediploidization**. *Nature* 2016, **533**(7602):200-205.
4. Shiina T, Dijkstra JM, Shimizu S, Watanabe A, Yanagiya K, Kiryu I, Fujiwara A, Nishida-Umehara C, Kaba Y, Hirono I *et al*: **Interchromosomal duplication of major histocompatibility complex class I regions in rainbow trout (*Oncorhynchus mykiss*), a species with a presumably recent tetraploid ancestry**. *Immunogenetics* 2005, **56**(12):878-893.
5. Rondeau EB, Minkley DR, Leong JS, Messmer AM, Jantzen JR, von Schalburg KR, Lemon C, Bird NH, Koop BF: **The genome and linkage map of the northern pike (*Esox lucius*): conserved synteny revealed between the salmonid sister group and the Neoteleostei**. *PloS one* 2014, **9**(7):e102089.
6. McConnell SC, Hernandez KM, Wcisel DJ, Kettleborough RN, Stemple DL, Yoder JA, Andrade J, de Jong JL: **Alternative haplotypes of antigen processing genes in zebrafish diverged early in vertebrate evolution**. *Proceedings of the National Academy of Sciences of the United States of America* 2016, **113**(34):E5014-5023.
7. Matsuo M, Asakawa S, Shimizu N, Kimura H, Nonaka M: **Nucleotide sequence of the MHC class I genomic region of a teleost, the medaka (*Oryzias latipes*).** *Immunogenetics* 2002, **53**:930-940.
8. Tsukamoto K, Hayashi S, Matsuo M, Nonaka M, Kondo M, Shima MI, Asakawa S, Shimizu N, Nonaka M: **Unprecedented intraspecific diversity of the MHC class I region of a teleost medaka, *Oryzias latipes***. *Immunogenetics* 2005, **57**:420-431.
9. Nonaka MI, Nonaka M: **Evolutionary analysis of two classical MHC class I loci of the medaka fish, *Oryzias latipes*: haplotype-specific genomic diversity, locus-specific polymorphisms, and interlocus homogenization**. *Immunogenetics* 2010, **62**(5):319-332.

Additional file 3. Text S2. Alignment of deduced PSMB8 amino acid sequences.

Sequences can be found in Additional file 2: Text S1 and phylogeny of bona fide sequences including accession numbers is shown in main text Figure 2. Aligned sequences are as follows: OnmyPSMB8F= rainbow trout genome Ia region, SasaPSMB8F= Genbank ACI66984.1, DarePSMB8F_19D= NP_001017791.1, SasaPSMB8a_#C =Atlantic salmon genome Ia region, SasaPSMB8a_#A=Atlantic salmon Ia region BAC 92I04, SasaPSMB8a_#B=Atlantic salmon Ia region BAC 714P22, OnkiPSMB8a= coho salmon genome Ia region, SasaPSMB8b_#A= Atlantic salmon Ib region BAC 8I14, SasaPSMB8b_#B= Atlantic salmon Ib region BAC 439J08, OnmyPSMB8a/omPSMB8aψ= rainbow trout BAC and genome, SasaPSMB8b_#C= Atlantic salmon genome Ib region, OnmyPSMB8b_#B= rainbow trout genome Ib region, OnmyPSMB8b_#A= rainbow trout Ib region BAC, OnkiPSMB8b= coho salmon genome Ib region, medaka OrlaPSMB8_Hd-rR= BAB83848., medaka OrlaPSMB8_HN1= BAD93264.1, medaka OrlaPSMB8_cab= BAJ07261.1, EsluPSMB8_NW71=Northern pike genome unplaced scaffold NW_017859271.1, EsluPSMB8_NW80= Northern pike genome unplaced scaffold NW_017859580.1, EsluPSMB8 = Northern pike genome chr.10, DarePSMB8a_19b= NP_571467.3, OnkiPSMB8b2/3=coho salmon genome, OnmyPSMB8bψ=rainbow trout genome Ib region, HosaPSMB8= NP_683720.2.

```
                           *        20         *        40         *        60         *
OnkiPSMB8a     : MALFDVSG---YKSHSEL--RGQIIGTG-VGHFIDRPNQQFSVPVGVD--PSGFLKSCSREG--GVSIDLNH :  62
OnmyPSMB8a_#Aψ : ------------------------------------------------------------------------ :   -
OnmyPSMB8a_#Bψ : ------------------------------------------------------------------------ :   -
SasaPSMB8a_#A  : ........---...Y...--........-.........K.........--............--........ :  62
SasaPSMB8a_#B  : ........---...Y...--........-.........K.........--............--........ :  62
SasaPSMB8a_#C  : ........---...Y...--........-.........K.........--............--........ :  62
SasaPSMB8b_#A  : ........---....AG.--....L...-...LV.....E.A......--............--........ :  62
SasaPSMB8b_#B  : ........---....AG.--....L...-...LV.....E.A......--............--........ :  62
SasaPSMB8b_#C  : ........---....AG.--....L...-...LV.....E.A......--............--........ :  62
SasaPSMB8b_#Cψ : ---------------------------------------------------------------------M.- :   2
OnmyPSMB8b_#A  : ........---....AG.--....L...-...LV.....E.A......--............--........ :  62
OnmyPSMB8b_#B  : ........---....AG.--....L...-...LV.....E.A......VS............--........ :  64
OnmyPSMB8b_#Bψ : ------------------------------------------------------------------------ :   -
OnkiPSMB8b     : ........---....AG.--....L...-...LV.....E.A......--............--........ :  62
OnkiPSMB8b2    : ------------------------------------------------------------------------ :   -
OnkiPSMB8b3    : ------------------------------------------------------------------------ :   -
EsluPSMB8_NW71 : ..I.C...---.N...Q.--..E.T.K.------------..SVLSL---......Y.H..QG.G...... :  52
EsluPSMB8_NW80 : ........---....L..--..KTL...LRHFNFGTT..E.A..A.M.--.......WRH..QG........ :  65
EsluPSMB8      : ........---....L..--..NTL...LRHLNFGTT.KE.A..A.M.--.........H..QG........ :  65
OrlaPSMB8_HdrR : ...AA.C.GQSSSE.FGQLFS.KQARLFDRPNHFSFGTKIQEFA.P.GNE.....R..N..E--..R..... :  70
OrlaPSMB8_HN1  : ...AN.C.--LKNYSEQSGQMFATRRLIDRPNHYSFGTKIQEFA.P.GEE.V......NT..--.ARFE.H. :  68
OrlaPSMB8_cab  : ...AA.C.GQSSSE.FGQLFS.KQARLFDRPNHFSFGTKIQEFA.P.GNE.....R..N..E--..R..... :  70
DarePSMB8a     : ...L....YK-.N.A.QFGFKQTLLDRS-NHYSFGTKC.E.A......--..K......C.D--..C..... :  66
OnkiPSMB8a.F   : ------------------------------------------------------------------------ :   -
OnmyPSMB8Fa_#B : ...L..C.IS-DWVKEDGFNAERASVDKVNHFNFAAQTPELA......PAEFLRPLVD.E..VD..K.N.E. :  71
SasaPsmb8F     : ...L..C.IS-DWVKEDGFNAERASVDKVNHFKFAAQTPELA......PAEFLRPLVD.E..VD..K.N.E. :  71
EsluPSMB8F     : ------------------------------------------------------------------------ :   -
Dare8F_19D     : ...M..CLSS-ELKQ.CFTANAWQSEPTNC.PSHYTFGLNANELAVPTGMDPEQFLGPLT..DSDIK.EFL. :  71
SasaPSMB8Fa_#A : ------------------------------------------------------------------------ :   -
SasaPSMB8Fa_#B : ------------------------------------------------------------------------ :   -
SasaPSMB8Fb_#A : ------------------------------------------------------------------------ :   -
SasaPSMB8Fb_#B : ------------------------------------------------------------------------ :   -
HosaPsmb8A     : ...L..C.APRGQRPESALPVAGSGRRSD-PGHYSFSMRSPELALPRGMQ.TE.FQ.LGGD.ERN.Q.EMA. :  71
```

```
                           80         *       100         *       120         *       140
OnkiPSMB8a     : GTTTLA------FTFRHGVIVAVDSRASAGSYIESKEANKVIEINPYLLGTMSGSAADCQYWERLLAKECRL : 128
OnmyPSMB8a_#Aψ : -----------------------------------------------------------------------. :   1
OnmyPSMB8a_#Bψ : -----------------------------------------------------------------------. :   1
SasaPSMB8a_#A  : ......------.....................A...................................... : 128
SasaPSMB8a_#B  : ......------.....................A...................................... : 128
SasaPSMB8a_#C  : ......------.....................A...................................... : 128
SasaPSMB8b_#A  : ......------.....................A...................................... : 128
SasaPSMB8b_#B  : ......------.....................A...................................... : 128
SasaPSMB8b_#C  : ......------.....................A...................................... : 128
SasaPSMB8b_#Cψ : ---.FD------.DLTWD..KSLFIFKT.-----L..T..I...H........................... :  60
OnmyPSMB8b_#A  : ......------.K...................A...................................... : 128
OnmyPSMB8b_#B  : ......------.K...................A...................................... : 130
OnmyPSMB8b_#Bψ : -----------------------------------------------------------------------. :   1
OnkiPSMB8b     : ......------.K...................A...................................... : 128
OnkiPSMB8b2    : ---------------------------------AL..................................... :  39
OnkiPSMB8b3    : ------------------------------------------------------------------------ :   -
EsluPSMB8_NW71 : ......QFRHGVL.LT.............V...A.....................................A : 124
EsluPSMB8_NW80 : ......------.K...................A...................................... : 131
EsluPSMB8      : ......------.K...................A...................................... : 131
OrlaPSMB8_HdrR : ......------.K................N.LA.NDV.................................. : 136
OrlaPSMB8_HNI  : .....S------.K.K............T....ATC.Y.....................K............ : 134
OrlaPSMB8_cab  : ......------.K................N.LA.NDV.................................. : 136
DarePSMB8a     : ......------.K................K..A...................................... : 132
OnkiPSMB8aF    : -----------------------------------------------------------------------R :   1
OnmyPSMB8Fa_#B : ......------.K.Q...M............VSTQMFK.......F............V....V......I : 137
SasaPsmb8F     : ......------.K.Q...M............VSTQMFK.......F............V....V......I : 137
EsluPSMB8F     : ------------------------------------------------------------------------ :   -
DarePSMB8F_19D : ......------.K.Q...M.............DT.DFK...............N....V....R......I : 137
SasaPSMB8Fa_#A : ------------------------------------------------------------------------ :   -
SasaPSMB8Fa_#B : ------------------------------------------------------------------------ :   -
SasaPSMB8Fb_#A : ------------------------------------------------------------------------ :   -
SasaPSMB8Fb_#B : ------------------------------------------------------------------------ :   -
HosaPsmb8A     : ......------.K.Q....A............SALRV................C................. : 137

                       *       160         *       180         *       200         *
OnkiPSMB8a     : YKLRNKQRISVSAASKLLCNMMLGYRGMGLSMGSMIVGWDNKGPGLYYVDDNATRLSGRMFSTGCGSSYAYG : 200
OnmyPSMB8a_#A  : .....R.................................................................. :  73
OnmyPSMB8a_#Bψ : .....R.................................................................. :  73
SasaPSMB8a_#A  : ........................................................................ : 200
SasaPSMB8a_#B  : ........................................................................ : 200
SasaPSMB8a_#C  : ........................................................................ : 200
SasaPSMB8b_#A  : ....................................I................................... : 200
SasaPSMB8b_#B  : ....................................I................................... : 200
SasaPSMB8b_#C  : ....................................I................................... : 200
SasaPSMB8b_#Cψ : ..........................................VQ.GRE------------------------ : 108
OnmyPSMB8b_#A  : ........................................................................ : 200
OnmyPSMB8b_#B  : ........................................................................ : 202
OnmyPSMB8b_#Bψ : .......................R..................VQ.--------------------------- :  46
OnkiPSMB8b     : ........................................................................ : 200
OnkiPSMB8b2    : ...............................----------------------------------------- :  70
OnkiPSMB8b3    : --------------............................VQ.--------------------------- :  31
EsluPSMB8      : ......K...........S.................I...............G.....Q.......N..... : 203
EsluPSMB8_NW71 : E.....K...........S.......---------------------------------------------- : 150
EsluPSMB8_NW80 : ......K...........S.........R.......I...............G.....Q.......N..... : 203
OrlaPSMB8_HdrR : .R...NH....A...................V....C...KE..........G.............N..... : 208
OrlaPSMB8_HNI  : .R...NH....A...................V....C...KE...I......GN..........S..N.... : 206
OrlaPSMB8_cab  : .R...NH....A...................V....C...KE..........G.............N..... : 208
DarePSMB8a     : ..................S.................C...KQ..........G.............N..... : 204
OnkiPSMB8aF    : IYKLRNE........T..A..VVN......RG-T..C.C.Q-..........GL..G-N.LT..S.N-.--. :  67
OnmyPSMB8Fa_#B : ......E...........A..VVN.........T..C...K...........GL..C.N.....S.NT.... : 209
SasaPsmb8F     : .N....E...........A..VVN.........T..C...K...........GL..C.N.....S.NT.... : 209
EsluPSMB8F     : ---------....G....A..VVN.........T..C.C.K...........GM..C.N.....S.N..... :  63
DarePSMB8F_19D : ......T....A......A..VAE.........T.VC...QR.......SSSG...A.D.....S.CN.... : 209
SasaPSMB8Fa_#A : ------------------------------------------------------------------------ :   -
SasaPSMB8Fa_#B : ------------------------------------------------------------------------ :   -
SasaPSMB8Fb_#A : ------------------------------------------------------------------------ :   -
SasaPSMB8Fb_#B : ------------------------------------------------------------------------ :   -
HosaPsmb8A     : .Y...GE...........S...CQ............C...K.........EHG.....N.....S.NT.... : 209
```

```
                   220       *         240       *         260       *         280
OnkiPSMB8a      : VIDSGYREDMTVEEAYELGRRGITHATHRDAYSGGVVNLYHMQEDGWIKVCKEDVSELIHRYRKGMF- : 267
OnmyPSMB8a_#Aψ  : .................................E...........................------- : 134
OnmyPSMB8a_#B   : ...................................................................- : 140
SasaPSMB8a_#A   : ...................................................................- : 267
SasaPSMB8a_#B   : ...................................................................- : 267
SasaPSMB8a_#C   : ...................................................................- : 267
SasaPSMB8b_#A   : .V.................................................................- : 267
SasaPSMB8b_#B   : .V.................................................................- : 267
SasaPSMB8b_#C   : .V.................................................................- : 267
SasaPSMB8b_#Cψ  : -------------------------------------------------------------------- :   -
OnmyPSMB8b_#A   : .M.................................................................- : 267
OnmyPSMB8b_#B   : .M....H...............................V............................- : 269
OnmyPSMB8b_#Bψ  : -------------------------------------------------------------------- :   -
OnkiPSMB8b      : .M.................................................................- : 267
OnkiPSMB8b2     : -------------------------------------------------------------------- :   -
OnkiPSMB8b3     : -------------------------------------------------------------------- :   -
EsluPSMB8_NW71  : -------------------------------------------------------------------- :   -
EsluPSMB8_NW80  : ............................................................H.K....- : 270
EsluPSMB8       : ...................................................................- : 270
OrlaPSMB8_HdrR  : .V....K............C...A...........S..M...R....................E...- : 275
OrlaPSMB8_HN1   : .L....K................V......S.......M..I..........D.....L.H.K....- : 273
OrlaPSMB8_cab   : .V....K............C...A...........S..M...R....................E...- : 275
DarePSMB8a      : .V.....................A......................................K....- : 271
OnkiPSMB8a.F    : MM...SQY.LS.P...D.AQ.A.F...........T..------------------------------ : 105
OnmyPSMB8Fa_#B  : .M.....Y.LS.P...D.AQ.A.F...........T..V...R.T.....SQ...GD.Y..FSNEKK- : 276
SasaPsmb8F      : .M.....Y.LS.P...D.AQ.A.F...........T..M...R.T.....SQ...GD.Y..FYNDKK- : 276
EsluPSMB8F      : .M.....Y.LS.P...D.AQ.A.F...........T..M...K.T..V..SQ...GD.Y..FNNENK- : 130
DarePSMB8F_19D  : .M...H.W.L..P...D.AE.A.Y..............M....KE.....SQN..GD.H.K.VAERKK : 277
SasaPSMB8Fa_#Aψ: ----------------------------------------.KRNT.....SQ...GD.Y..FYNDKK- :  27
SasaPSMB8Fa_#Bψ: ----------------------------------------.KRNT.....SQ...GD.Y..FYNDKK- :  27
SasaPSMB8Fa_#Cψ: ----------------------------------------.KRNT.....SQ...GD.Y..FYNDKK- :  27
SasaPSMB8Fb_#Aψ: ----------------------------------------..R.T...N.SQ...AD.Y.HLYNEKK- :  27
SasaPSMB8Fb_#Bψ: ----------------------------------------..R.T...N.SQ...AD.Y.HLYNEKK- :  27
SasaPSMB8Fb_#Cψ: ----------------------------------------..R.T...N.SQ...AD.Y.HLYNEKK- :  27
HosaPsmb8A      : .M.....PNLSP....D....A.AY.....S.......M...K....V..EST...D.L.Q..EANQ- : 276
```

Additional file 4: Figure S2. Teleost MHCI data

Table of Contents		Page
S2a	Phylogeny of MHCI alpha 1 domain amino acid sequences from selected species.	2
S2b	Phylogeny of deduced MHCI alpha 2 domain sequences from selected species.	3
S2c	Phylogeny of deduced MHCI alpha3 domain sequences from selected species.	4
S2d	Alignment of selected MHC class I amino acid sequences	5

Figure S2a. Phylogeny of MHCI alpha 1 domain amino acid sequences from selected species.

The evolutionary history was inferred by using the Maximum Likelihood method based on the JTT matrix-based model (1). The percentage of trees in which the associated taxa clustered together is shown next to the branches. The tree is drawn to scale, with branch lengths measured in the number of substitutions per site. The percentage of trees in which the associated taxa clustered together in the bootstrap test (100 replicates) is shown next to the branches. All positions with less than 95% site coverage were eliminated. Evolutionary analyses were conducted in MEGA7 (2). Red font shows MHCI genes linked to PSMB8F sequences and colour boxes show alpha 1 domain lineage categories (3). Sequence references not shown in figure can be found in Additional file 2: Text S1.

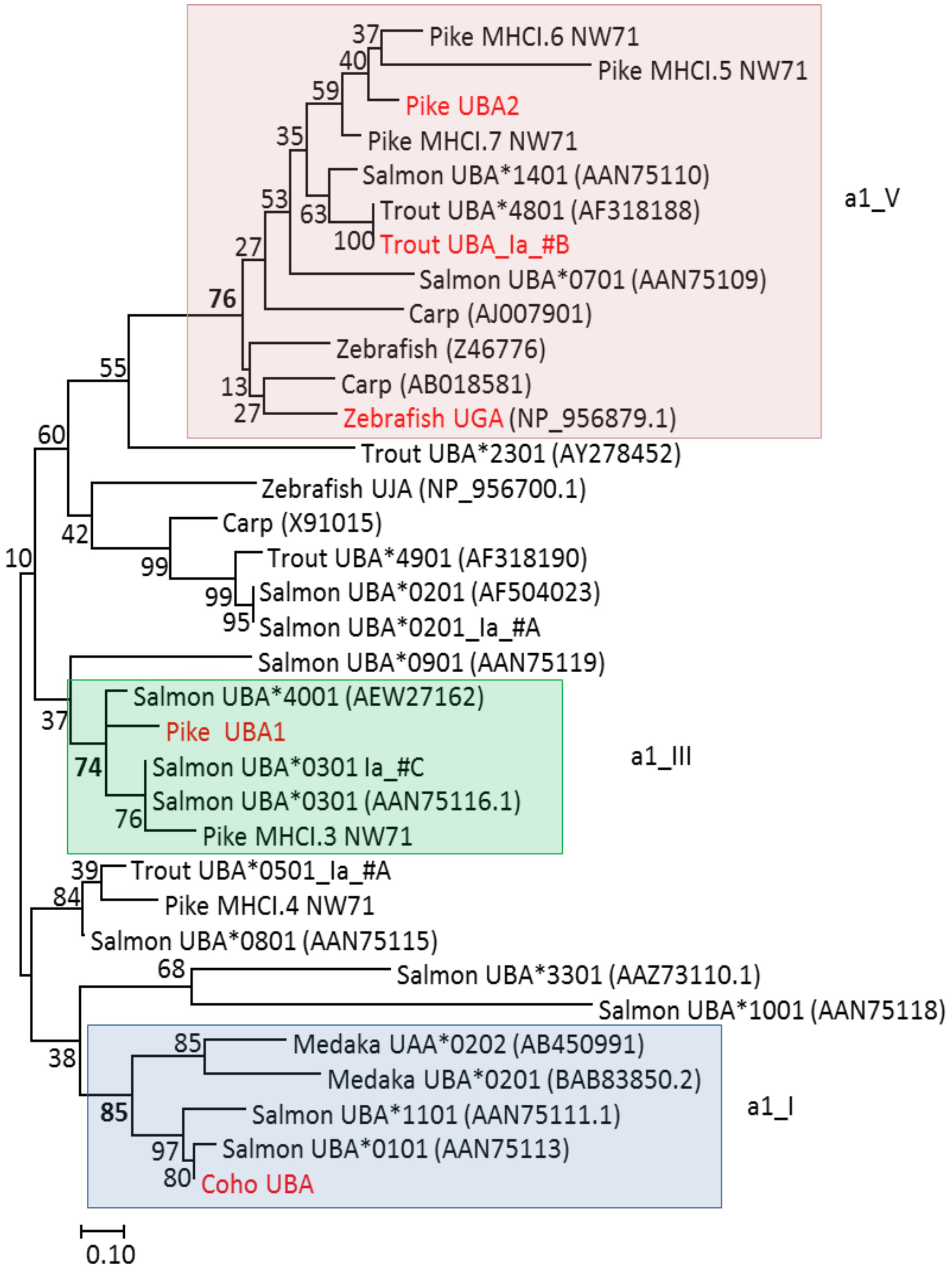

Figure S2b. Phylogeny of deduced MHCI alpha 2 domain sequences from selected species.

The evolutionary history was inferred by using the Maximum Likelihood method based on the Jones et al. w/freq. model (1). The percentage of trees in which the associated taxa clustered together in the bootstrap test (100 replicates) is shown next to the branches. The tree is drawn to scale, with branch lengths in the same units as those of the evolutionary distances used to infer the phylogenetic tree. All positions with less than 95% site coverage were eliminated. Evolutionary analyses were conducted in MEGA7 (2). Red font shows MHCI genes linked to PSMB8F sequences and colour boxes show alpha 2 domain lineage categories (3). Sequence references not shown in figure can be found in Additional file 2: Text S1.

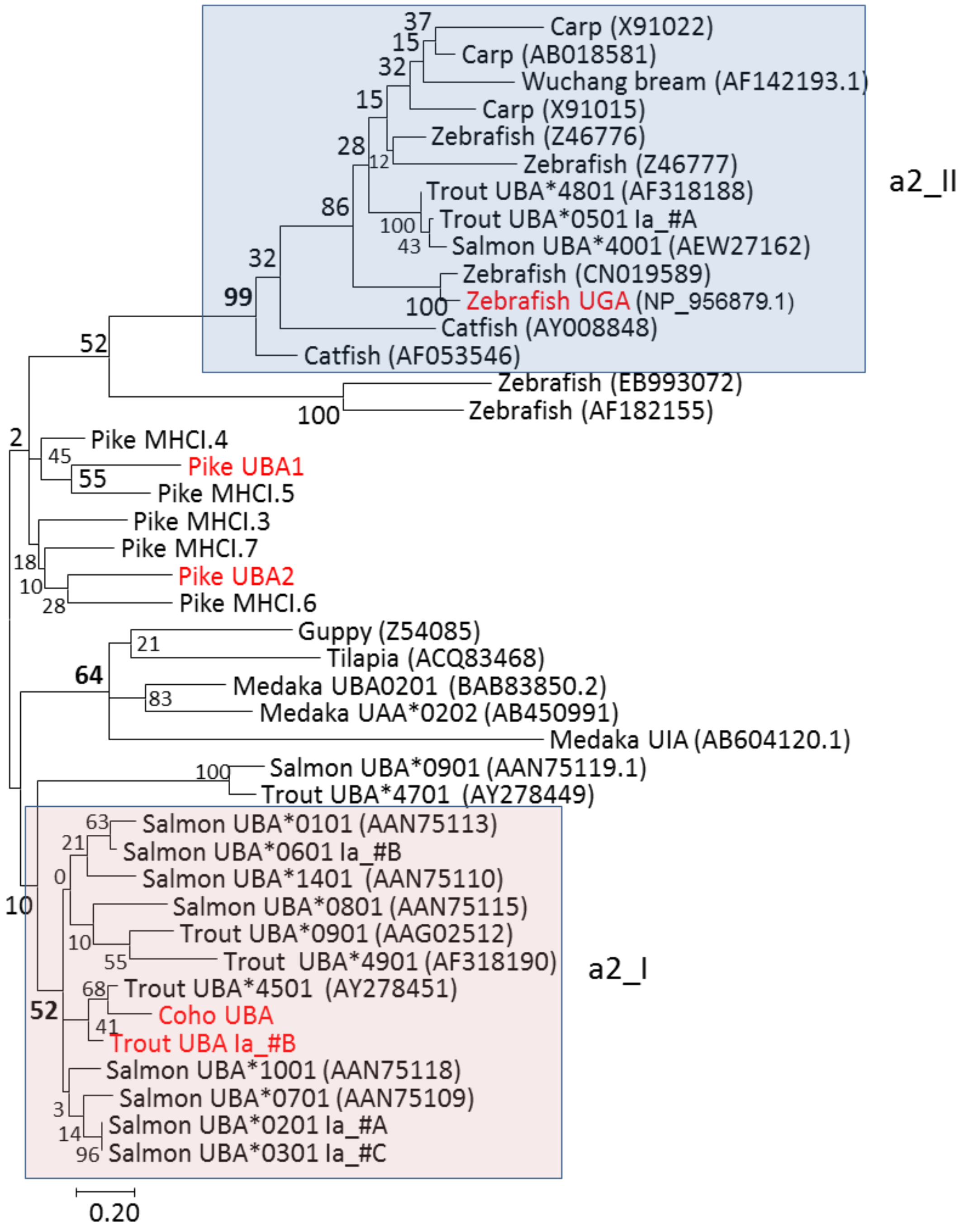

.

Figure S2c. Phylogeny of deduced MHCI alpha3 domain sequences from selected species.

Deduced MHCI alpha 3 domain sequences from selected species were used in the unrooted phylogenetic tree. The evolutionary history was inferred by using the Maximum Likelihood method based on the Jones et al. w/freq. model The percentage of trees in which the associated taxa clustered together in the bootstrap test (100 replicates) is shown next to the branches. The tree is drawn to scale, with branch lengths in the same units as those of the evolutionary distances used to infer the phylogenetic tree. All positions with less than 95% site coverage were eliminated. Evolutionary analyses were conducted in MEGA7 (2). Red font shows MHCI genes linked to PSMB8F sequences. Sequence references not shown in figure can be found in Additional file 2: Text S1.

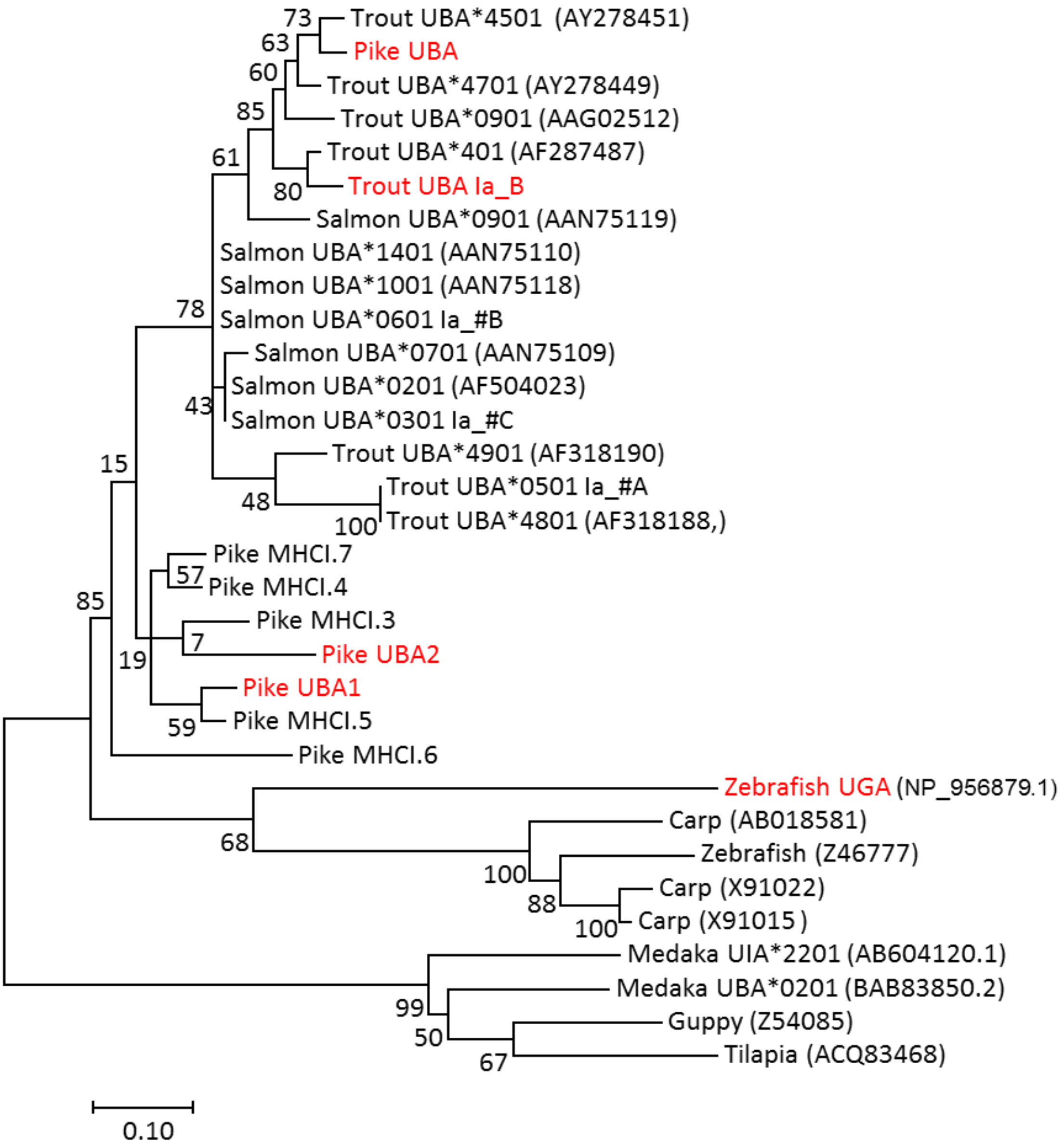

Figure S2d. Alignment of selected MHC class I amino acid sequences

Aligned sequences mostly originate from the haplotypes described in main text Figure 1, Additional file 1: Figure 1 and Additional file 2: Text S1 and are as follows: *onmyUBA*3301*= rainbow trout genome haplotype Ia_#B, *onmyUBA*0501*= rainbow trout BAC haplotype Ia_#A, *sasaUBA*0201*= Atlantic salmon BAC 92I04 haplotype Ia_#A , sasaUBA*0601= Atlantic salmon BAC 714P22 haplotype Ia_#B, *sasaUBA*0301*= Atlantic salmon genome Ia region haplotype Ia_#C, *sasaULA*= Atlantic salmon genome Ia region haplotypes Ia_A and C, onkiUBA= coho salmon genome, *dareUGA*= zebrafish haplotype 19D (4), esluUBA1/-2= the Northern pike genome Chr.10, esluMHCI.3-I.7=unplaced genomic scaffold NW_017859271 and HLA-A2= AAA76608.2. Numbering according to the mature HLA-A2 sequence. Sequence references not shown in figures S2a-d can be found in Additional file 2: Text S1.

```
                          Leader sequence     Alpha 1 domain
OnmyUBA*Ia_#B   : ------------------------ATHSLKYFYTAVSG-DIDFPEFTAVGLVDKGQ :  31
OnmyUBA*0501_#A : ----MK-GFILLLLGIGLLHTASA.....R.V...T..-VP.....VSL.I..GM. :  50
SasaUBA*0201_#A : ----MKSCILLLFLGIVLLHTVSA..NT.Q.....T..-IDN....VTM.I.NGH. :  51
SasaUBA*0601_#B : -------------------------------------------------------- :   -
SasaUBA*0301_#C : ----MKC-FILLLLG-IALHSSSA.....R.V...T..-IP.....VT....NGEP :  49
SasaULA_#A/C    : ----MKC-FILLLLS-ISLHAASA.M...R.V...T..-MP.....VT....NGEP :  49
OnkiUBA         : ----MK-GIILLVLGIGLLHTASAV..........S.E-VPN....VV..M..GV. :  50
EsluUBA1        : -------------------MSGVQT....R.V...T..-IP.....VT....NGEP :  36
EsluUBA2        : ----MK-VIILSILGIGFLPTVSAV.........G...-X.N.....T.....N.. :  50
EsluMHCI.3      : ----MK-VFILLIMGIGLLHSVSA.....C.V...T..-IP.....VT....NREP :  50
EsluMHCI.4      : ----MK-VIILLILGIGLLPTVSA.S..IR.V...T..-IP.....VNL.IL.GM. :  50
EsluMHCI.5      : ----MK-VFILLILGXGHLHTLSA.F...HTC......-.THL.....L..L.NV. :  50
EsluMHCI.6      : ----M-YFFVLLILIIGHLHTVSAV.........G...-..N.....T.....NVT :  50
EsluMHCI.7      : ----MK-VIVLLILGIG-LPTVSTV.........G...-G.N.....V.....N.. :  49
DareUGA         : --------MQQVLLFLLGAHLAYAG....R........-........M.....G.. :  47
HLA-A2          : MAVMAPRTLVLLLSGALALTQTWAGS..MR..F.S..RPGRGE.R.I...Y..DT. :  56
                                          1        *        20        *
                                                                  Glycosylation site
OnmyUBA*Ia_#B   : FMYFDS--STKTAVPKTEWMKR-EGADYWDRQTQGLIGAHQTFKVNIQTLKDRFNQ :  84
OnmyUBA*0501_#A : ID.Y..--N..RV...QD..AKT..S...E....NS..DE....A..DVA.Q.... : 104
SasaUBA*0201_#A : IDHY..--I..R.IQ.A..ISGAVDP...KTN..IYA.TETV.VN..NVA.S.... : 105
SasaUBA*0601_#B : -------------------------------------------------------- :   -
SasaUBA*0301_#C : IS.Y..--IIRRET.RQD..AKT..S...ES...VS..SE....A..DVA.Q.... : 103
SasaULA_#A/C    : IS.Y..--IIRSET.RQD...EAVDP...N.N..TS..DE....A..DVA.Q.... : 103
OnkiUBA         : MVHY..--NSQR....QD.VNKAADPQ..E.N.GNFK.SQ....A..DIV.Q.... : 104
EsluUBA1        : IS.Y..--IIR.ET.RQD..XKAVDPE..S.N..LS..TEK...D..DVVNQ.... :  90
EsluUBA2        : .I....--N.............-..P....SE..TG.NL..V..NS..STM..... : 103
EsluMHCI.3      : IS.Y..--IIR.ET.RQD..ANN..S...ES...ISN.TE....A..DI..Q.... : 104
EsluMHCI.7      : .I....--N.............-..P....S...I...SQPV..N...VA...... : 102
EsluMHCI.6      : .I....--N..........I..-V.PG..VS..KTC.NL..F..N...VA...... : 103
EsluMHCI.5      : VA....--N..........IR.-..P..LNSL.NLG.N...RL.AHL.IF..Q... : 103
EsluMHCI.4      : ID.Y..--N..KV...QD..AKAV.S.....E..NS..REKS..A..D.A.Q.... : 104
DareUGA         : .I....--KKME.......IRQN.......IN..R..AT..A..N...VA.E.... : 101
HLA-A2          : .VR...DAASQRME.RAP.IEQ-..PE...GE.RKVKAHS..HR.DLG..RGYY.. : 111
                         40        *          60        *         80
```

Additional file 4: Figure S2

```
                      Alpha 2 domain        TAPBP dependency (5)
OnmyUBA*Ia_#B   : SKSTGVHVFQWMYGCEWDDEAGVTEGFDQYGYDGEDFIAFDLKTTKWIAPTPQAVI : 140
OnmyUBA*0501_#A : TG--...T..L.....LG.D-.I.R.DF.L....A..LSL.KS.LT.T.ANQK... : 157
SasaUBA*0201_#A : T---....N.K.........T........D.......L......LT.......... : 158
SasaUBA*0601_#B : -----...N..................E.W..............KS.......... :  51
SasaUBA*0301_#C : TG--....N.K.........T........D.......L......LT.......... : 157
SasaULA_#A/C    : TG--....Y.N..........................L......L........SL. : 157
OnkiUBA         : .G--...IV.M.........T......E.H....................M..... : 158
EsluUBA1        : TM--...IN.L.........T........F.F......SL..M.LT....K..... : 144
EsluUBA2        : .M......V.M.........TEA.....R........LLL....LS....KS..F. : 159
EsluMHCI.3      : TG--....V.N.........T...D.............V....RLT....K...F. : 158
EsluMHCI.7      : .M......V.M.........T..I.....H........S.....L..V......F. : 158
EsluMHCI.6      : .M......L......D....T..IQ..........N..SL....LS....K...F. : 159
EsluMHCI.5      : .M.....TL.V.M.......T.....YM.F........S.....LT..TSK..... : 159
EsluMHCI.4      : TG--....A.M.F.......T...R..N.Q.F......S.....LT....K..... : 158
DareUGA         : .Q--...T..V.....LE.DG-S.R.YW.........LSL.KS.LT.T.TK..... : 154
HLA-A2          : .E-A.S.TV.R....DVGSDWRFLR.YH..A...K.Y..LKEDLRS.T.ADMA.QT : 166
                     *         100       *         120       *         140

                                                            Alpha 3 domain
OnmyUBA*Ia_#B   : TKHKWDSNTARNE-QNKNYYTQICIEWLKKYVDYGKSTLMRTVPPSVSLLQK---A : 192
OnmyUBA*0501_#A : ..L...ATG.EAN-FQ...LENT.........N...D..E.K.R........---T : 209
SasaUBA*0201_#A : ..L.......Q..-YR...L..T........L....................---T : 210
SasaUBA*0601_#B : ..L....D..Q..-HR....................................---T : 103
SasaUBA*0301_#C : ..L.......Q..-YR...L..T........L....................---T : 209
SasaULA_#A/C    : ..L...N.M.QIQ-.D.H.L..TS.......L....................---T : 209
OnkiUBA         : ....L.....N..-YK......T..D.................C.....F..---T : 210
EsluUBA1        : S.LRL.N.IVHSNNYL...F..E..D....FL.....S..............---T : 197
EsluUBA2        : ..N.F...KDT.L-YLRK.F..T.VD...........S.......T......---T : 211
EsluMHCI.3      : N.N...NDRGWI.-.T...L..T..............S.........F...R---T : 210
EsluMHCI.7      : S.N.L.Y.E.G..-GE.H.L..E..............S..............---T : 210
EsluMHCI.6      : ..N....DK.D..-YL.H.F.LE........LQ.EETSQ.............---T : 211
EsluMHCI.5      : ..DR..NDK.D..-YW...L..E..DS..NFL.....S..............---T : 211
EsluMHCI.4      : ..N...N.K.G..-YW...L..E..D.V......E..S..............---T : 210
DareUGA         : ..N...AD-NADRQYT.S.LENE....V........D..E.KDA.E.FM..---KD : 206
HLA-A2          : .....EAA-HVA.QLR-A.LEGT.V...RR.LEN..E..Q..DA.KTHMTHHAVSD : 220
                         *           160       *         180       *

OnmyUBA*Ia_#B   : PSSPVTCHATGFYPRDVMVSWQKDGQDHHEDVEYGEILQNDDGTFQKSSHLTVTPE : 248
OnmyUBA*0501_#A : ..............SG...F......EQ.G...H..............T....... : 265
SasaUBA*0201_#A : ..............SG.................H..T................... : 266
SasaUBA*0601_#B : ..............SG....................T................... : 159
SasaUBA*0301_#C : ..............SG.................H..T................... : 265
SasaULA_#A/C    : ..............SG....................T................... : 265
OnkiUBA         : S..L..........S.....................T.P................. : 266
EsluUBA1        : ..............SE.I.T.K....EQ.....V..T.H.........V....K.. : 253
EsluUBA2        : ..............SR.N.T.K.N..EQ.....M....P.......T.VR.N.K.. : 267
EsluMHCI.3      : ..............SR...T.M....EQY....V..T...........V....K.. : 266
EsluMHCI.7      : ..............SG.H.A.K...........M..T...........VL...K.. : 266
EsluMHCI.6      : ..............SE.NIL.....PFQ.....T.HT.P.G.......VL...K.. : 267
EsluMHCI.5      : ..............SE.I.T.K.N.........M..T.H.........V....K.. : 267
EsluMHCI.4      : ..............SG.H.T.K...........M..T...........VR.N.K.. : 266
DareUGA         : .....V.Q......SNI.MT...NKEE.F...DV.AT.T.A......TVT.S.K.. : 262
HLA-A2          : HEATLR.W.LS...AEITLT..R..E.QTQ.T.LV.TRPAG......WAAVV.PS- : 275
                     200      *         220       *         240       *
```

```
                                                  Connecting peptide/ transmembrane region
OnmyUBA*Ia_#B    : DRKNSK--YQCVVQVKGIKEDFIGVLPD--------------QDAANVVPIIVGVV : 288
OnmyUBA*0501_#A : EW..N.--......LA..ED.ITK..IESEIQTNFGKTNRGSN.PITIGL..G..I : 319
SasaUBA*0201_#A : EW..N.--.......T.LQ....K..TESEIKTN--------WNDP.I.L..GV.. : 312
SasaUBA*0601_#B : EW..N.--.......T.V.....K..TESEIKTN--------WNDP.I.L...V.. : 205
SasaUBA*0301_#C : EW..N.--.......T.LQ....K..TESEIKTN--------WNDP.I.L..GV.. : 311
SasaULA_#A/C     : EW..N.--.......T.V.....K..TESEIKTNWGNT-----NIGF.P------- : 307
OnkiUBA          : ......--...............K....--------------L............. : 306
EsluUBA1         : EW..I.--.......S..N....K..TEDQIQTNCRKTNR---.PTSTGL..GV.. : 304
EsluUBA2         : E...N.--.......A..N....KD.TE---------------.TTSIG...GV.. : 306
EsluluMHCI.3     : EW.KN.--.......S.......KD.TEDQIQT-------------.TGFVP---- : 303
EsluMHCI.7       : EW..N.--.......S..NK...KD.TESEIQTNR--------EPTSIG...G-A. : 311
EsluMHCI.6       : EW..N.--.......S..NK...KD.TESEIQTN---------RDTSIGH..TV.. : 312
EsluMHCI.5       : EW..K.--.......S..N.V..K..TEDEIETN---------WGKTQK------- : 305
EsluMHCI.4       : EW..N.--......IS.T.....KD.TEDELVSNWGKTNRGVN.QTSIG...GV.. : 320
DareUGA          : EW..N.EA.R....HV.A.N.V.VTVK.IRSNEG---------SDN---T.AII.G : 306
HLA-A2           : -GQEQR--.T.H..HE.LP--KPLT.RWEPSSQP------------TIPIVGIIAG : 314
                              260         *         280                   *

                                                 Cytoplasmic region
OnmyUBA*Ia_#B    : ALLLVVVAVVV--GVVIWRKRSKKGFVPASRTESDLENQRRSKDQTNVQRGIVKN : 341
OnmyUBA*0501_#A : ...VII.V-----.....K.KN........TSDT.S..SGKGIQKI--------- : 360
SasaUBA*0201_#A : ...........--.....K.K.........TSDT.SD.SG.AAQM.--------- : 356
SasaUBA*0601_#B : ....L......--.....K.K.........TSDT.SD.SG.AAQM.--------- : 249
SasaUBA*0301_#C : ...........--.....K.K.........TSDT.SD.SG.AAQM.--------- : 355
SasaULA_#A/C     : ----------------------------.NTSDVGSNSSHNTAPKE--------- : 325
OnkiUBA          : ...........--...............T.TSDT.SD.SG.AAP.I--------- : 350
EsluUBA1         : GVSIL.IMAA.F-.....KNK...VSDGG.NSSNNSSPKP--------------- : 343
EsluUBA2         : .G...I..A.II-.....K.K........NTSDDGSNSSNNTAPKA--------- : 351
EsluMHCI.3       : ----------------------------ANTSDDGSNSSNNTAPKA--------- : 321
EsluMHCI.7       : .G...IAV..-I-.....K.K.....I..NTSDDGSNSSNNTAPKA--------- : 355
EsluMHCI.6       : .G..AIG.A.I--...NRK.K.......PNTSDDGSNSSNK.SSNV--------- : 356
EsluMHCI.5       : ------------------------------------------------------- :   -
EsluMHCI.4       : .G...I..A.II-.....K.NR.-.....NTSDGG--SSTN.APEA--------- : 362
DareUGA          : CVAA.ALLA-.IA.LIY..RSNGY.KASSKD.D.EQSDP.AVEVK---------- : 350
HLA-A2           : LV.FGA.ITGAVVAA.M..RK.SDRKGGSYSQAASSDSAQG.DVSLTACKV---- : 365
                            300      *         320        *         340
```

References:

1. Jones DT, Taylor WR, & Thornton JM (1992) The rapid generation of mutations data matrixes from protein sequences. *Computer Applications in the Biosciences* 8:275-282.
2. Kumar S, Stecher G, & Tamura K (2016) MEGA7: Molecular Evolutionary Genetics Analysis Version 7.0 for Bigger Datasets. *Molecular biology and evolution* 33(7):1870-1874.
3. Kiryu I, *et al.* (2005) New MHC class Ia domain lineages in rainbow trout (*Oncorhynchus mykiss*) which are shared with other fish species. *Fish & shellfish immunology* 18:243-254.
4. McConnell SC*, et al.* (2016) Alternative haplotypes of antigen processing genes in zebrafish diverged early in vertebrate evolution. *Proceedings of the National Academy of Sciences of the United States of America* 113(34):E5014-5023.
5. Park B, Lee S, Kim E, & Ahn K (2003) A single polymorphic residue within the peptide-binding cleft of MHC class I molecules determines spectrum of tapasin dependence. *Journal of immunology* 170(2):961-968.

Additional file 5: Figure S3. PSMB9 and PSMB12 data

Table of Contents		Page
S3a	Phylogeny of deduced PSMB9 and PSMB12 amino acid sequences from selected species.	2
S3b	Alignment of deduced PSMB9 amino acid sequences	3
S3c	Alignment of deduced PSMB12 amino acid sequences	5

Figure S3a. Phylogeny of deduced PSMB9 and PSMB12 sequences from selected species.

Salmonid haplotype and pike deduced amino acid sequence references can be found in Additional file 2: Text S1 while remaining references are shown in the figure. Zebrafish sequences originate from McConnell et al(1). The evolutionary history was inferred by using the Maximum Likelihood method based on the Le and Gascuel 2008 model (2). The tree with the highest log likelihood is shown. The percentage of trees in which the associated taxa clustered together is shown next to the branches. The tree is drawn to scale, with branch lengths measured in the number of substitutions per site. The percentages of replicate trees in which the associated taxa clustered together in the bootstrap test (100 replicates) are shown next to the branches. All positions with less than 95% site coverage were eliminated. Evolutionary analyses were conducted in MEGA7 (3). Red font indicated gene sequences originating from PSMB8F gene containing haplotypes. Salmon is Atlantic salmon, Trout is rainbow trout and Pike is Northern pike.

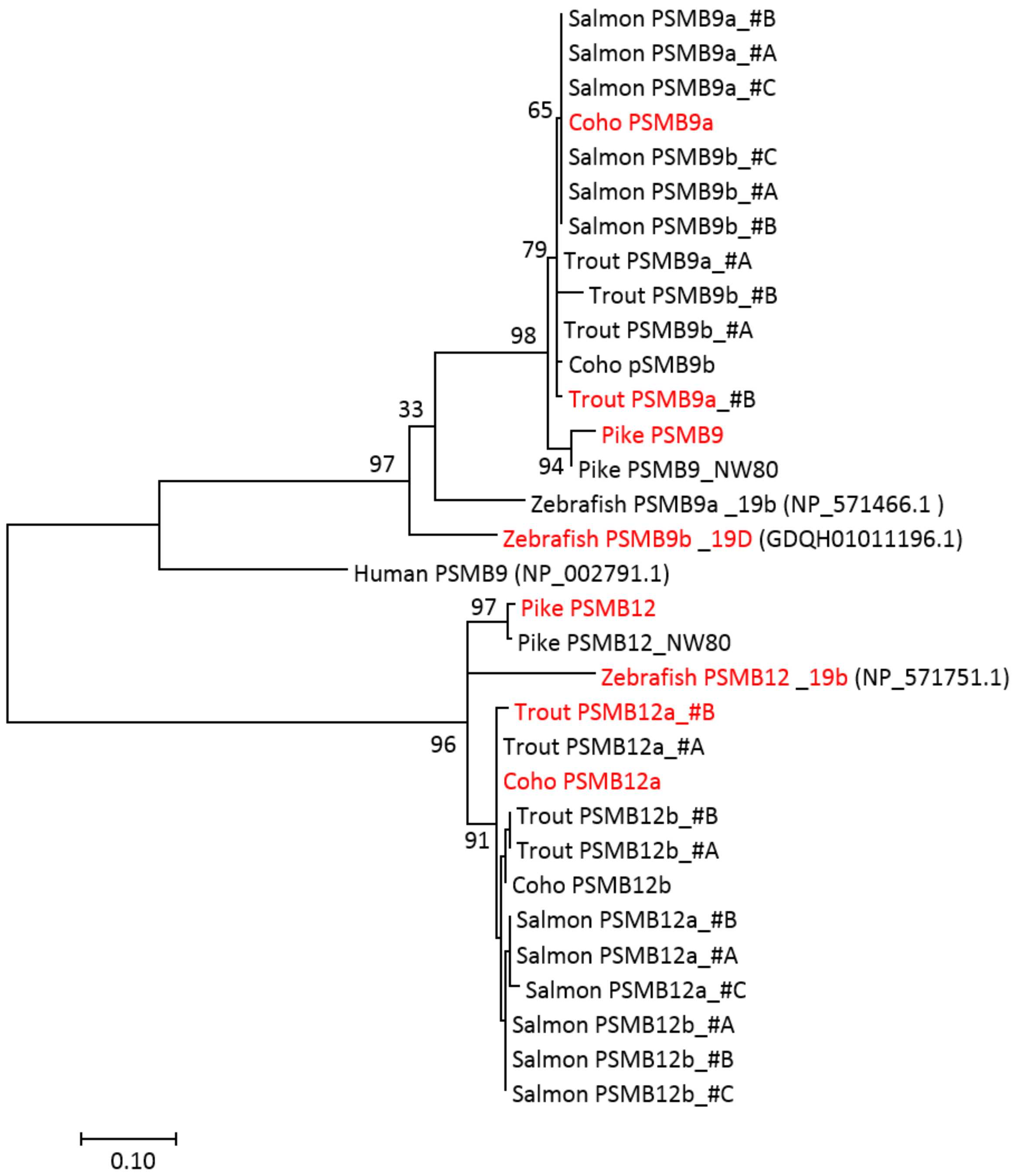

Figure S3b. Alignment of deduced PSMB9 amino acid sequence sequences

Alignment of deduced PSMB9 amino acid sequences from coho salmon (Onki), Atlantic salmon (Sasa), rainbow trout (Onmy), Northern pike (Eslu), zebrafish (Dare) and human sequences. Sequence references can be found in Additional file 2: Text S1 or in Figure S3a. The two Northern pike sequences are assumed allelic variants where EsluPSMB9_NW80 originates from scaffold NW_017859580.1 and EsluPSMB9 from the assembled region on Chr.10. For zebrafish haplotype PSMB9 sequences, see McConnell et al. (1). PSMB12 gene sequences originating from PSMB8F containing haplotypes are shown using red font. Amino acid residues are colour coded according to physiochemical properties.

```
                           *        20         *        40         *
OnkiPSMB9a     : MLEESS-EPGWLSE-EVKTGTTIIAIEFDGGVVLGSDSRVSAGETVVN-RVMNKLSLL :  55
SasaPSMB9a_#A  : ......-.......-.................................-......... :  55
SasaPSMB9a_#B  : ......-.......-.................................-......... :  55
SasaPSMB9a_#C  : ......-.......-.................................-......... :  55
SasaPSMB9b_#A  : ......-.......-.................................-......... :  55
SasaPSMB9b_#B  : ......-.......-.................................-......... :  55
SasaPSMB9b_#C  : ......-.......-.................................-......... :  55
OnmyPSMB9a_#A  : ..D..L-.......-.................................-......... :  55
OnmyPSMB9a_#B  : ..D..L-.......-.................................-......... :  55
OnmyPSMB9b_#A  : ..D...-.......-.................................-......... :  55
OnmyPSMB9b_#B  : ..D...-.......-.................................-......... :  55
OnkipSMB9b     : ..D...-.......-.................................-......... :  55
EsluPSMB9      : .....T-.SE.Q.--..........V..N...................-......... :  54
EsluPSMB9_NW80 : ......-....P..-..........V......................-......... :  55
DarePSMB9a     : .S..LFP.......-..........VT......I..........S...-.......P. :  56
DarePSMB9b     : -MQNGG-.HEGTNG-G.GM......V.......V.........AS...-.......P. :  54
Human PSMB9    : ..RAGAPTGDLPRAG..H.....M.V.......M..........A...-..FD...P. :  57

                 60         *        80         *       100         *
OnkiPSMB9a     : HDKIYCALSGSAADAQTIAEMVNYQLDVHSIEVGEDPQVRSAATLVKNISYKYKEELS : 113
SasaPSMB9a_#A  : .......................................................... : 113
SasaPSMB9a_#B  : .......................................................... : 113
SasaPSMB9a_#C  : .......................................................... : 113
SasaPSMB9b_#A  : .......................................................... : 113
SasaPSMB9b_#B  : .......................................................... : 113
SasaPSMB9b_#C  : .......................................................... : 113
OnmyPSMB9a_#A  : .......................................................... : 113
OnmyPSMB9a_#B  : .......................................................... : 113
OnmyPSMB9b_#A  : .......................................................... : 113
OnmyPSMB9b_#B  : .......................................................... : 113
OnkiPSMB9b     : .......................................................... : 113
EsluPSMB9      : ................................I......................... : 112
EsluPSMB9_NW80 : ................................I......................... : 113
DarePSMB9a     : ....................I............ED..L.C.................. : 114
DarePSMB9b     : ....................I............D...L.C.................. : 112
Human PSMB9    : .ER.............AV.D.AA...EL.G..LE.P.L.LA..NV.R......R.D.. : 115
```

```
                        120         *        140         *        160         *
OnkiPSMB9a     : AHLIVAGWDKRGGGQVY-VTLNGLLSRQPFAVGGSGSAYVYGFVDAEYRKAMSKEDCQ : 170
SasaPSMB9a_#A  : .................-........................................ : 170
SasaPSMB9a_#B  : .................-........................................ : 170
SasaPSMB9a_#C  : .................-........................................ : 170
SasaPSMB9b_#A  : .................-........................................ : 170
SasaPSMB9b_#B  : .................-........................................ : 170
SasaPSMB9b_#C  : .................-........................................ : 170
OnmyPSMB9a_#A  : .................-...................S.................... : 170
OnmyPSMB9a_#B  : .................-...................S.................... : 170
OnmyPSMB9A_#A  : .................-...................S.................... : 170
OnmyPSMB9b_#B  : .................-...................S.................... : 170
OnkipSMB9b     : ..........K......-...................S.................... : 170
EsluPSMB9      : .........R......F-...................S..........Q.G....... : 169
EsluPSMB9_NW80 : ................F-...................S..........Q.G....... : 170
DarePSMB9a     : ..........K......-A..S...TK....I.....F.IN.......K.N.T.RE.. : 171
DarePSMB9b     : .........R.E.....-A..S...T...........F.I.........AG.T.KE.. : 169
Human PSMB9    : ...M.....Q.E.....-G..G.M.T....I.....TFI..Y...A.KPG..P.E.R : 172

                          180         *        200         *        220         *
OnkiPSMB9a     : QFVVNTLSLAMSRDGSSGGVAYLVTIDEKGAEEKCILGNELPTFYDQ----------- : 217
SasaPSMB9a_#A  : ...............................................----------- : 217
SasaPSMB9a_#B  : ...............................................----------- : 217
SasaPSMB9a_#C  : ...............................................----------- : 217
SasaPSMB9b_#A  : ...............................................----------- : 217
SasaPSMB9b_#B  : ...............................................----------- : 217
SasaPSMB9b_#C  : ...............................................----------- : 217
OnmyPSMB9a_#A  : ...............................................----------- : 217
OnmyPSMB9a_#B  : .....S.........................................----------- : 217
OnmyPSMB9a_#A  : ...............................................----------- : 217
OnmyPSMB9b_#B  : ..............................--.TFVVP-------------------- : 206
OnkipSMB9b     : ...............................................----------- : 217
EsluPSMB9      : ......I........................................----------- : 216
EsluPSMB9_NW80 : ......I........................................----------- : 217
DarePsmb9a     : E....A.T...G..........V....KD.T....V......K.F.E----------- : 218
DarePsmb9b     : E..I.S.....G...............SESV........Q......PDTVEPSKAVKV : 227
Human PSMB9    : R.TTDAIA............I.....TAA.VDHRV.......K...E----------- : 219
```

Figure S3c. Alignment of deduced PSMB12 amino acid sequences

Alignment of deduced PSMB12 amino acid sequences from coho salmon (Onki), Atlantic salmon (Sasa), rainbow trout (Onmy), Northern pike (Eslu), zebrafish (Dare) against the human PSMB9 sequences. Sequence references can be found in Additional file 2: Text S1 or in Figure S3a. The two Northern pike sequences are assumed allelic variants where EsluPSMB12_NW80 originates from scaffold NW_017859580.1 and EsluPSMB12 from the assembled region on Chr.10. The zebrafish darePSMB12 sequence is from McConnell et al. (1). PSMB12 gene sequences originating from PSMB8F containing haplotypes are shown using red font. Amino acid residues are colour coded according to physiochemical properties.

```
                                       *        20         *        40         *
OnkiPSMB12a     : -MERHL-MDSQIKG--VSTGTTILAVTFNGGVIIGSDSRASIGGYYVSSKTINKLI :  52
SasaPSMB12a_#A  : -.....-.......--............................S........... :  52
SasaPSMB12a_#B  : -.....-.......--............................S........... :  52
SasaPSMB12a_#C  : -.....-.......--............................S........... :  52
OnmyPSMB12a_#A  : -..KRF-.......--........................................ :  52
OnmyPSMB12a_#B  : --------------------........................-........... :  35
SasaPSMB12b_#A  : -....F-.......--............................S........... :  52
SasaPSMB12b_#B  : -....F-.......--............................S........... :  52
SasaPSMB12b_#C  : -------.......--............................S........... :  47
OnmyPSMB12b_#A  : -------.......--............................N........... :  47
OnmyPSMB12b_#B  : -------.......--............................N........... :  47
OnkiPSMB12b     : -------.......--........................................ :  47
EsluPSMB12      : -....Y-...K...--.........................M..N..C........ :  52
EsluPSMB12_NW80 : -....Y-...K...--.........................M..S..C........ :  52
DarePSMB12      : -.D..H-PY..VN.--..........K..............M.ES........... :  52
Human PSMB9     : MLRAGAPTGDLPRAGE.H.....M..E.D...VM.....V.A.EAV.N-RVFD..S :  55
                    60         *        80         *       100         *
OnkiPSMB12a     : QVHDRIFCCIAGSLADAQAVTKAAKFQISFHSIQMESPPLVKAAASVLKELCYNNK : 108
SasaPSMB12a_#A  : ........................................................ : 108
SasaPSMB12a_#B  : ........................................................ : 108
SasaPSMB12a_#C  : ........................................................ : 108
OnmyPSMB12a_#A  : ........................................................ : 108
OnmyPSMB12a_#B  : ........................................................ :  91
OnmyPSMB12b_#A  : ........................................................ : 103
OnmyPSMB12b_#B  : ........................................................ : 103
SasaPSMB12b_#A  : ........................................................ : 108
SasaPSMB12b_#B  : ........................................................ : 108
SasaPSMB12b_#C  : ........................................................ : 103
OnkiPSMB12b     : ........................................................ : 103
EsluPSMB12      : ......................T......L.................M........ : 108
EsluPSMB12_NW80 : ......................T......L.................M........ : 108
DarePSMB12      : ......................M....L..................IMR....S.. : 108
Human PSMB9     : PL.E..Y.ALS..A......ADM.AY.LEL.G.EL.E....L...N.VRNIS.KYR : 111
                       120         *       140         *       160
OnkiPSMB12a     : EELQAGFITAGWDRKKGPQVYTVALGGMLLSQPFTIGGSGSTYIYGYADAKYKPDM : 164
SasaPSMB12a_#A  : ........................................................ : 164
SasaPSMB12a_#B  : ........................................................ : 164
SasaPSMB12a_#C  : VM...................................................... : 164
OnmyPSMB12a_#A  : ........................................................ : 164
OnmyPSMB12a_#B  : ........................................................ : 147
OnmyPSMB12b_#A  : ........................................................ : 159
OnmyPSMB12b_#B  : ........................................................ : 159
SasaPSMB12b_#A  : ........................................................ : 164
SasaPSMB12b_#B  : ........................................................ : 164
SasaPSMB12b_#C  : ........................................................ : 159
OnkiPSMB12b     : ........................................................ : 159
EsluPSMB12      : .............K.................L...............T........ : 164
EsluPSMB12_NW80 : .............K.................L...............T........ : 164
DarePSMB12      : ...R...............I.V.S.......................V...F.... : 164
Human PSMB9     : .D.S.HLMV....QRE.G...-GT.....TR...A.......F....V..A...G. : 166
```

```
                    *       180         *       200         *       220
OnkiPSMB12a    : SKEECLQFATNALALAMGRDNVSGGVAHLVVITEEGVEHIVIPGDKLPKFHDE : 217
SasaPSMB12a_#A : .........K.............................V............. : 217
SasaPSMB12a_#B : .........K.............................V............. : 217
SasaPSMB12a_#C : .........K.............................V............. : 217
OnmyPSMB12a_#A : ..................................................... : 217
OnmyPSMB12a_#B : ..................................................... : 200
OnmyPSMB12b_#A : .R.....................................V............. : 212
OnmyPSMB12b_#B : .R.....................................V............. : 212
SasaPSMB12b_#A : .......................................V............. : 217
SasaPSMB12b_#B : .......................................V............. : 217
SasaPSMB12b_#C : .......................................V............. : 212
OnkiPSMB12b    : .R.....................................V............. : 212
EsluPSMB12     : .R................................G....V............. : 217
EsluPSMB12_NW80: TR................................G....V............. : 217
DarePSMB12     : TL..AT..S.................V.......A..K...V...E....... : 217
Human PSMB9    : .P...RR.T.D.I....S..GS....IY..T..AA..D.R..L.NE....Y.. : 219
```

References:

1. McConnell SC, *et al.* (2016) Alternative haplotypes of antigen processing genes in zebrafish diverged early in vertebrate evolution. *Proceedings of the National Academy of Sciences of the United States of America* 113(34):E5014-5023.
2. Le SQ & Gascuel O (2008) An improved general amino acid replacement matrix. *Molecular biology and evolution* 25(7):1307-1320.
3. Kumar S, Stecher G, & Tamura K (2016) MEGA7: Molecular Evolutionary Genetics Analysis Version 7.0 for Bigger Datasets. *Molecular biology and evolution* 33(7):1870-1874.

Additional file 6: Figure S4. PSMB7, PSMB10, PSMB13 data

Table of Contents		Page
S4a	Phylogeny of deduced PSMB7, PSMB10 and PSMB13 amino acid sequences	2
S4b	Alignment of deduced PSMB7, PSMB10 and PSMB13 amino acid sequences	3

Figure S4a. Phylogeny of deduced PSMB7, PSMB10 and PSMB13 amino acid sequences

Phylogenetic analysis of PSMB7, PSMB10 and PSMB13 sequences from selected species. Haplotype sequences originating from Atlantic salmon (Salmon), Rainbow trout, (Trout), Coho, Northern pike (Pike), and zebrafish are shown using extensions a/b_#A-C or _19. The remaining sequences have accession numbers in parenthesis. The evolutionary history was inferred by using the Maximum Likelihood method based on the Jones et al. with frequency model (1). The tree is drawn to scale, with branch lengths measured in the number of substitutions per site. The percentages of replicate trees in which the associated taxa clustered together in the bootstrap test (100 replicates) are shown next to the branches. All positions with less than 95% site coverage were eliminated. Evolutionary analyses were conducted in MEGA7 (2). Salmonid and Northern pike haplotypes sequences are described in Materials and Methods while Zebrafish haplotype sequences are from McConnell et al.(3) and medaka haplotype sequences from Hd-rR (4), HN1 (5) and cab (6). Other sequences have Genbank references in parenthesis. PSMB10 and -13 sequences residing in PSMB8F containing haplotypes are shown using red font. The tree is unrooted. Sequences can be found in Additional file 2: Text S1.

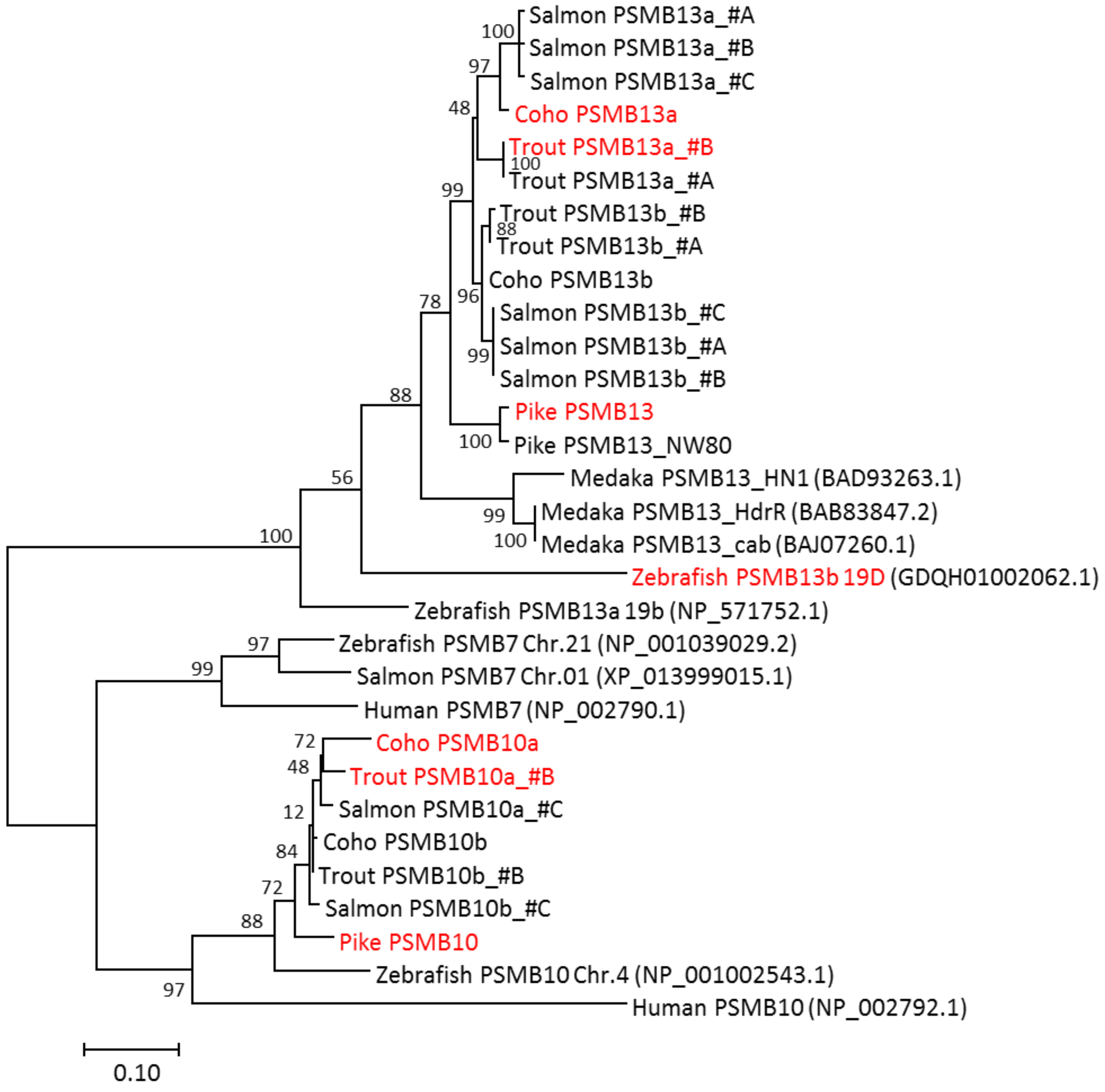

Figure S4b. Amino acid sequence alignment PSMB7_10_13 sequences

The SasaPSMB13a_#C sequence is incomplete but most likely does not represent a pseudogene. Sequence references can be found in Figure S4a or in Additional file 2: Text S1. Gene sequences originating from PSMB8F containing haplotypes are shown using red font. Onki is Coho salmon, Sasa is Atlantic salmon, Onmy is rainbow trout, Eslu is Northern pike, Orla is medaka and Dare is zebrafish.

```
                           *         20         *         40         *         60         *
OnkiPSMB13a      : MALS-NVLEIPTSGFNFENVARNVALEGLLEGGQTKTPKPMKTGTTIAGLVCKEGVVLGADTRATSGEVVAD :  71
OnmyPSMB13a_#A   : ....-..V.T.A........C.........Q.....A............VL..N.................. :  71
OnmyPSMB13a_#B   : ....-..V.T.A........C.--......Q.....A............VL..N.................. :  69
SasaPSMB13a_#A   : ....-............K...............H...L.................................. :  71
SasaPSMB13a_#B   : ....-.......T....................H...L.................................. :  71
SasaPSMB13a_#C   : ....-............................H...L.................................. :  71
SasaPSMB13b_#A   : ...T-..V.T.A........S............H..A............V...D.................. :  71
SasaPSMB13b_#B   : ...T-..V.T.A........S............H..A............V...D.................. :  71
SasaPSMB13b_#C   : ...T-..V.T.A........S............H..A............V...D.................. :  71
OnmyPSMB13b_#A   : ....-..V.T.A........S............H..A............I...D.................. :  71
OnmyPSMB13b_#B   : ....-..V.T.A........S............H..A............I...D.................. :  71
OnkiPSMB13b      : ....-..V.T.A........S............H..A............V...D.................. :  71
EsluPSMB13       : ...P-....T.PT.......F............R..A............V...D............S..... :  71
EsluPSMB13 NW80  : ...P-....T.P.....D..F............R..A............V...D............S..... :  71
OrlaPSMB13 HdrR  : ....-...DS.AA....D.A...A.F...F...--Q....L........V.F.D............S..... :  69
OrlaPSMB13 HN1   : ....-...DS.AA....D.A...A.F...F...--Q....L........V.F.D............S..... :  69
OrlaPSMB13 cab   : ....-...DS.AA....D.A...A.F...F...--Q....L........V.F.D............S..... :  69
DarePSMB13a_19b  : ...TSH...PSLC......AT..IV..NGA.E.KI.P..AL........V.F.D............D..... :  72
DarePSMB13b_19D  : .FAT--..KT.EP..C...SL..E..DS.VK.---.K..AL........V.F.D............NK.... :  67
SasaPSMB10a_#C   : .LHNSRPPQPQSA..S...TR..AV...N.SELGYSS..AR........I.F.D..I........DDM.... :  72
SasaPSMB10b_#C   : .LNNSRPYQPQSA..S...TR..AV...N.SELGYSS..AR........I.F.D..I........DDM.... :  72
OnmyPSMB10b      : .LNNSRPCQPQSA..S...TR..AV...N.SELGYSS..AR........I.F.D..I........DDM.... :  72
OnkiPSMB10b      : .LNNSRPCQPQSA..S...TR..AV...N.SELGYSS..AR........I.F.D..I........DDM.... :  72
OnmyPSMB10a      : .LHNSRPPQPQSA..S...TR..AV...N.SELGYSS..AR........I.F.D..I......T.DDM.... :  72
OnkiPSMB10a      : .LHNSRPPQPQSA..S...TR..AV...N.SELGYSS..AR........I.F.D..I.A......DDM.... :  72
EsluPSMB10       : .LNTSRPCQPQSG..S..DSR..AV...N.SELGYSS..AR........I.F.D..I........DDM.... :  72
DarePSMB10_4     : .LNTST--KTL.G..S...TR..AV..AN.SEKGYSA.NAR..........F.D..I........DDM.... :  70
Human PSMB10     : .L---KPALE.RG..S...CQ..AS..RV.P.L--.V.HAR..........FQD..I........NDS.... :  67
DarePSMB7_21     : -MATVS.CQYQPG..S...CR..AL..ADITKLGFSS.AAR......C.I.Y.D...........E.MI... :  71
SasaPSMB7_LG01   : -MATLS.CQPQLG..S...CK..AV..AEVTKLGCNI.AAR......C.V.F.D.L.........E.MI... :  71
SasaPSMB7_LG11   : ---TLS.CQTQLG..S...GK.LGLV.D--------------------------.L.........E.MI... :  43
Human PSMB7      : -MAAVS.YAP.VG..S.D.CR..AV..ADFAKRGY.L..VR........V.Y.D.I.........E.M.... :  71
```

Additional file 6. Figure S4

```
                          80         *       100         *       120         *       140
OnkiPSMB13a     : KMCAKIHYISPNIY--CCGAGTAADTEKTTDLLSSNLTIFSMNSGRNPRVVMAVNILQDMLFRYRGQIGASL : 141
OnmyPSMB13a_#A  : ..............--........................................................ : 141
OnmyPSMB13a_#B  : ..............--........................................................ : 139
SasaPSMB13a_#A  : ..............--........................................................ : 141
SasaPSMB13a_#B  : ..............--........................................................ : 141
SasaPSMB13a_#C  : .............S--......................................--------.......... : 133
SasaPSMB13b_#A  : ............M.--...............M..........T............................. : 141
SasaPSMB13b_#B  : ............M.--...............M..........T............................. : 141
SasaPSMB13b_#C  : ............M.--...............M..........T............................. : 141
OnmyPSMB13b_#A  : ............M.--........................................................ : 141
OnmyPSMB13b_#B  : ............M.--........................................................ : 141
OnkiPSMB13b     : ............M.--........................................................ : 141
EsluPSMB13      : .........A....--..........................D..........A................H. : 141
EsluPSMB13_NW80 : .........A....--..........................D..........A................H. : 141
OrlaPSMB13 HdrR : ..............--......................V..L...................Y..H.....N. : 139
OrlaPSMB13 HN1  : ..............--......................V..L...................Y..H.....N. : 139
OrlaPSMB13 cab  : ..............--......................V..L...................Y..H.....N. : 139
DarePSMB13a_19b : .........A....--...............M........................I.........M...N. : 142
DarePSMB13b_19D : ...E....LA....--..........Q.I.E.I.................I...SV........H.....N. : 137
SasaPSMB10a_#C  : .N.M.....A....--.....V...A.V..QMM...VELH.LST..P.L...VTRQ.KQ.....Q.H..S.. : 142
SasaPSMB10b_#C  : .N.M.....A...---.....V...A.V..QMM...VELH.LST..P.L..TVTRQ.KQ.....Q.H..S.. : 141
OnmyPSMB10a     : .N.M.....A...--S.....V...A.V..QMM...VELH.LST..P.L..TVTRQ.KQ.....Q.H..S.. : 142
OnmyPSMB10b     : .N.M.....A....--.....V...A.V..QMM...VELH.LST..P.L..TVTRQ.KQ.....Q.H..S.. : 142
OnkiPSMB10a     : .N.M.....A....CS.....V...A.V..QMM...VELH.LST..P.L..TVTRQ.KQ....ES--MNE.V : 142
OnkiPSMB10b     : .N.M.....A....--.....V...A.V..QMM...VELH.LST..P.L..TVTRQ.KQ.....Q.H..S.. : 142
EsluPSMB10      : .N.M.....A....--.....V...A.V..QMM...VELH.LST..P.L..TVTRQ.KQ.....Q.H..S.. : 142
DarePSMB10_4    : .N.M.....A....--.....V...A.V..QMM...VELH.LST..P.L.A.VTRQ.KQ.....Q.H..S.. : 140
Human PSMB10    : .S.E...F.A.K..--.....V...A.M..RMVA.KMELHALST..E...ATVTR..RQT....Q.HV.... : 137
Dare PSMB7_21   : .N.S..........--...........M..QII....ELH.LST..L...AT.NRM.KQ.....Q.Y...A. : 141
SasaPSMB7_LG01  : .N.S..........--...........M..QII....ELH.LSTS.L...AT.NRM.KQ.....Q.H...A. : 141
SasaPSMB7_LG11  : .N.S.....-CPSS--...........M..QII....ELH.LST..L...AT.NRM.KQ.....TTH----- : 107
Human PSMB7     : .N.S...F......--..........DM..Q.I....ELH.LST..L....T.NRM.KQ.....Q.Y...A. : 141

                         *       160         *       180         *       200         *
OnkiPSMB13a     : ILGGVDCSGNHLYTVGPYGSIDNVPYLAMGSG-DLAALGILEDRFKPNMEMEEAKELVRDAIHSGIMSDLGS : 212
OnmyPSMB13a_#A  : .......T.....K..................-.............A......................... : 212
OnmyPSMB13a_#B  : .......T.....K..................-.............A......................... : 210
SasaPSMB13a_#A  : .......T................Q.......-....................................... : 212
SasaPSMB13a_#B  : .......T................Q.......-....................................... : 212
SasaPSMB13a_#C  : .......T................Q.....--------------------...................... : 185
SasaPSMB13b_#A  : .......T........................-.................L..................... : 212
SasaPSMB13b_#B  : .......T........................-.................L..................... : 212
SasaPSMB13b_#C  : .......T........................-.................L..................... : 212
OnmyPSMB13b_#A  : .......T........................-.................L....Q................ : 212
OnmyPSMB13b_#B  : .......T........................-.................L....Q................ : 212
OnkiPSMB13b     : .......T........................-.................L..................... : 212
EsluPSMB13      : .......T.S.........D..K.T.......-N................L..................... : 212
EsluPSMB13_NW80 : .......T.S.........D..K.T..S....-.................L..................... : 212
OrlaPSMB13 HdrR : .......T............VNK.........-.............HDL.L.K..........A........ : 210
OrlaPSMB13 HN1  : .......T............VNK.........-..........G.QHDL.L.K..K.....V.....N.... : 210
OrlaPSMB13 cab  : .......T............VNK.........-.............HDL.L.K..........A........ : 210
DarePSMB13a_19b : .......T.S..........M.K.........-N...M........V..DL.Q..A..S...QA...C.... : 213
DarePSMB13b_19D : L......T.G...DI....DM.K..F......-N.P.M.....G..S..DL.S..H......YA.V.N.... : 208
SasaPSMB10a_#C  : .V....VT.A...S.Y.H..Y.KL.F.T....-AG..IS.F...YR....L....K......AA..FC.... : 213
SasaPSMB10b_#C  : .V....VT.A...S.Y.H..Y.KL.F.T....-AG..IS.F...YR....L....K......AA..FC.... : 212
OnmyPSMB10a     : .V....VT.A...S.Y.H..Y.KL.F.T..GSGAG..IS.F...YR..AQL....K......AA..FC.... : 214
OnkiPSMB10a     : TM.KAAI.------IFEDR---YR.N.EVRCS------SF.L--YLSLAQL....K......AA..FC.... : 197
OnmyPSMB10b     : .V....VT.A...S.Y.H..Y.KL.F.T....-AG..IS.F...YR....L....K......AA..FC.... : 213
OnkiPSMB10b     : .V....VT.A...S.Y.H..Y.KL.F.T....-AG..IS.F...YR....L....K......TA..FC.... : 213
EsluPSMB10      : .V....VT.A...SAY.H..Y.KL.F.T....-AGP.IS.F....R....L.D..Q......VA..FC.... : 213
DarePSMB10_4    : .V....VN.AQ..S.Y.H..Y.KL.F.T....-AAS.ISVF...Y.....L....Q......TA..FC.... : 211
Human PSMB10    : .V....LT.PQ..G.H.H..YSRL.FT.L...-QD...AV.....Q...TL.A.QG.LVE.VTA..LG.... : 208
DarePsmb7_21    : V......T.P...SIY.H..T.KL..VT....-S...MAVF...YR.D..E.D..S......AA..FN.... : 212
SasaPSMB7_LG01  : V......N.P...SIY.H..T.KL..VT....-S...MAVF...Y.QD..E.D..R......AA..FN.... : 212
SasaPSMB7_LG11  : ------------------------------------------------------------------------ :   -
Human PSMB7     : V.....VT.P...SIY.H..T.KL..VT....-S...MAVF..K.R.D..E....N..SE..AA..FN.... : 212
```

```
                         220         *       240         *       260         *       280
OnkiPSMB13a       : GNNIDICVITKQGVDYIRPYQESEYKDKRQKRYKYGPG-TTSILTEKIVPLELEVVQETVQQMDTA--- : 277
OnmyPSMB13a_#A    : ..............................R....R..-......................R....--- : 277
OnmyPSMB13a_#B    : ..............................R....R..G......................R....--- : 276
SasaPSMB13a_#A    : .............................R......T.-..................K...R....--- : 277
SasaPSMB13a_#B    : .............................R......T.-..................K...R....--- : 277
SasaPSMB13a_#C    : ........................-----------.T.-..................K...R....--- : 239
SasaPSMB13b_#A    : ..............................R....R..-......................R....--- : 277
SasaPSMB13b_#B    : ..............................R....R..-......................R....--- : 277
SasaPSMB13b_#C    : ..............................R....R..-......................R....--- : 277
OnmyPSMB13b_#A    : ..............................R....R..-......................R....--- : 277
OnmyPSMB13b_#B    : ..............................R....R..-.............M.--------------- : 265
OnkiPSMB13b       : ..............................R....R..-......................R....--- : 277
EsluPSMB13        : ................L.......F.....M....H..-..P.........QM........R....--- : 277
EsluPSMB13_NW80   : ........................F.....R....H..-..P.........QM........R....--- : 277
OrlaPSMB13 HdrR   : ...................F......ET.KPK...R..-..PV..K.V...K....E.IQ......--- : 275
OrlaPSMB13 HN1    : ..........AE.......F....F..R.EIK...RQ.-..PV..K..T..KM......T.K.E.V--- : 275
OrlaPSMB13 cab    : ...................F......ET.KPK...R..-..PV..K.V...K....E.IQ......--- : 275
DarePSMB13a_19b   : .....L.....E.......HK..P.NY...AK...KS.-..P...KTVNK...DL.H....M.E..ASS : 281
DarePSMB13b 19D   : .H...L....RD...........QHIG..EGQ...K..-..AV....LHHI.MDLIE.S..K.E.S--- : 273
SasaPSMB10a_#C    : .S.V.L....QA..Q.L.S.DQPAQ.G.KEGQ...K..-..AV..KTVT..P.D..D.SI.L..AQ--- : 278
SasaPSMB10b_#C    : .S.V.L....QA..Q.L.S.DQPAQ.G.KEGQ...K..-..AV..KTVT..P.H..D.SI.L...Q--- : 277
OnmyPSMB10a       : .S.V.L....HA..Q.L.G.DQPAQ.G.SRGQ.T.K..-..AV..KTVT..P.DI.D.SI.L...Q--- : 279
OnkiPSMB10a       : .S.V.L....QA..Q.L.G.DQPA------------..-..AV..KTVT..P.DI.D.SI.L...Q--- : 250
OnmyPSMB10b       : .S.V.L....QA..Q.L.G.DQPA------------..-..AV..KTVT..P.D..D.SI.L...Q--- : 266
OnkiPSMB10b       : .S.V.L....QA..Q.L.G.DQPAQ.G..EGQ...K..-..AV..KTVT..P.D..D.SI.L...Q--- : 278
EsluPSMB10        : .S.V.L.I..QA..Q.L.A.DQPAQ.G.KEGQ...K..-..AV..KTVT..P.DI.D.S..L...Q--- : 278
DarePSMB10_4      : .S.V.L....DKK...L.T.DQPVH.NQ.GGT.R.K..-..AV.SKTVT..T.D..D.S.HV...E--- : 276
Human PSMB10      : .G.V.A.....T.AKLL.TLSSPTEPV..SG..HFV..-..AV..QTVK..T..L.E....A.EVE--- : 273
DarePSMB7_21      : .S...V.....GK...L..HDIANK.GV.TGS.R.KH.-..GV.SKAVT..N.DM.E.S..T...S--- : 277
SasaPSMB7_LG01    : .S...L.....GR...L..HDMANK.GV.TGN...KQ.-..GV..KSVIK.D....E....T..----- : 276
SasaPSMB7_LG11    : --------------------------------------------------------------------- :   -
Human PSMB7       : .S...L...S.NKL.FL...TVPNK.GT.LG..RCEK.-..AV.....T...I..LE....T...S--- : 277
```

References:

1. Jones DT, Taylor WR, & Thornton JM (1992) The rapid generation of mutations data matrixes from protein sequences. *Computer Applications in the Biosciences* 8:275-282.
2. Kumar S, Stecher G, & Tamura K (2016) MEGA7: Molecular Evolutionary Genetics Analysis Version 7.0 for Bigger Datasets. *Molecular biology and evolution* 33(7):1870-1874.
3. McConnell SC*, et al.* (2016) Alternative haplotypes of antigen processing genes in zebrafish diverged early in vertebrate evolution. *Proceedings of the National Academy of Sciences of the United States of America* 113(34):E5014-5023.
4. Matsuo M, Asakawa S, Shimizu N, Kimura H, & Nonaka M (2002) Nucleotide sequence of the MHC class I genomic region of a teleost, the medaka (*Oryzias latipes*). *Immunogenetics* 53:930-940.
5. Tsukamoto K*, et al.* (2005) Unprecedented intraspecific diversity of the MHC class I region of a teleost medaka, *Oryzias latipes*. *Immunogenetics* 57:420-431.
6. Nonaka MI & Nonaka M (2010) Evolutionary analysis of two classical MHC class I loci of the medaka fish, *Oryzias latipes*: haplotype-specific genomic diversity, locus-specific polymorphisms, and interlocus homogenization. *Immunogenetics* 62(5):319-332.

Additional file 7: Text S3. Alignment of deduced TAP2 amino acid sequences

Salmonid and pike sequence references can be found in Additional file 2: Text S1. The assumed allelic Northern pike variants are from the genome (EsluTAP2) or from the unplaced genomic scaffold EsluNW80= NW_017859580.1) where some residues in the Pike TAP2 sequence are polished using the TSA sequence GATF01026336.1. Zebrafish sequences DareTAP2a_19B= NP_001006594.1 and DareTAP2d/e_19D are described in (1). Remaining sequence references are frog (*Xenopus laevis*) Xela TAP2_I=NP_001081860.1, Xela TAP2_II=AAI70327.1, human TAP2= AHW47941.1 and the chicken /Gaga) sequences are shown with accession numbers in the alignment. See rat (2), chicken (3) and frog (4) references for individual TAP polymorphisms. Polymorphic residues within species are shown using yellow shading. Variable residue positions in Northern pike TAP2 sequences coinciding with variable positions in chicken or rat sequences are shown using green shading. Cyan shading shows human TAP2 residues that crosslink with bound peptide (5) while predicted transmembrane regions (http://proteinformatics.charite.de/rhythm/index.php?site=home) are shown using grey shading in the coho (Onki) TAP2a sequence.

```
OnkiTAP2a     : -MTKMMLRTCVFAMAVG-LCIDITTLA---FGASISKTGTRTSDAFGNVVRLWVVAGIRLVLLL :  59
SasaTAP2a_#A  : -.........AL.....-.........---.........P..F..................... :  59
SasaTAP2a_#B  : -.........AL.....-.........---.........P..F..................... :  59
SasaTAP2a_#C  : ----....M.A......-.......FCAPC..E...E..PI.FGT................... :  59
OnmyTAP2a_#A  : -----.....A..V..V-..V....FCAPG..E......PK.F.T..........S........ :  58
OnmyTAP2a_#B  : -----.....A..VT..-.......FCAPG..E......PI.F.T..........S........ :  58
SasaTAP2b_#A  : ----..R.....T....-.......FCATGL........PISF.V...L.....E........F :  59
SasaTAP2b_#B  : ----..R.....T....-.......FCATGL........PISF.V...L.....E........F :  59
SasaTAP2b_#C  : ----..R.....T....-.......FCATGL........PISF.V...L.....E........F :  59
OnmyTAP2b_#A  : -----............-.......FCATG.....P...PISF.V..SLL...........I.F :  58
OnmyTAP2b_#B  : -----............-.......FCATG.....P...PISF.V..SLL...........I.F :  58
OnkiTAP2b     : ----.............-.......FCATG.........PISF.L...LL...........I.F :  59
EsluTAP2NW80  : -----.IKA..L.F.LISID.IL..FSAPGS.S--....PI.C.....L.NV.I..V......V :  57
EsluTAP2      : --M..IIK..LL.I.L.---L...IFSAPG.......I.QI.Y-T..YI.SV.I..V..F.F.V :  58
OrlaTAP2_cab  : MADR.IRKVVALIL.LC---L.LSLFY-----------ASDRFSF..H.A...LS.AL.WSAVS :  50
OrlaTAP2_HN1  : MADR.IRKVVALIL.LC---L.LSLFY-----------ASDRFSF..H.A...LS.AL.WSAVS :  50
OrlaTAP2_HdrR : MADR.IRKVVALML.LC---L.LSLFY-----------ASDRFSF..H.A...LS.AL.WSAVS :  50
DareTAP2a_19B : -----.RKVL...CMLC---F..LIVLILDLAETLL.SP.SSL.YN--LF.Q.AES.L.CT..Y :  54
DareTAP2d_19D : -----.ELKMQH.K.LL---V.LAMSCVLHYSTN---AFI.INSNVFLLAQ..L.S.VKWIG.R :  53
DareTAP2e_19D : ---------------------------------------------------------------- :   -
XelaTAP2_I    : ------------MVMLQ---LPLIV.LLDVSLNYL.AHLVYKYCPDKLLAA..L.SL.K.PM.G :  49
XelaTAP2_II   : ------------MASLS---FPVILFMADF.LNYFAAKLIYKYYPYEILGA..LLSL.K.PV.A :  49
GagaBAG69399  : ------------MAMPPYILRLSC..LLADLALMLALAHFFPAL.HLGW.GS.LE..L..LV.G :  52
GagaAEE25620  : ------------MAMPPYILRLSC..LLADLALMLALAHFFPAL.HLGW.GS.LE..L..LV.G :  52
GagaBAG69412  : ------------MAMPPYILHLSC..LLADLALMLALAHFFPAL.HLGS.GS.LE..L..LV.G :  52
GagaBAG69426  : ------------MAMPPYILRLSC..LLADLALMLALAHFFPAL.HLGW.GS.LE..L..LV.G :  52
Rat TAP2_I    : ------------MALS--YPRPWAS.LLVDLALLGLLQRSLGTLLPPGLPG..LEGTL..GV.W :  50
Rat TAP2_II   : ------------MALS--HPRPWAS.LLVDLALLGLLQSSLGTLLPPGLPG..LEGTL..GV.W :  50
Human TAP2    : ------------MRLP--DLRPW.S.LLVDAALLWLLQ.PLGTLLPQGLPG..LEGTL..GG.W :  50
                                        *        20         *        40        *
```

```
OnkiTAP2a     : GLSL--LTLGSIKPVFKRWLAVHCFLAPVYETGRLMLYGGSPERVYRSLGLGSPSLWPLCTVAA : 121
SasaTAP2a_#A  : ..T.--............................R....S......G..........L...A.. : 121
SasaTAP2a_#B  : ..T.--............................R....S......G..........L...A.. : 121
SasaTAP2a_#C  : ..T.--............................R....S......G..........L...A.. : 121
OnmyTAP2a_#A  : ....--.............................I.....T.SACG..--.G....L...A.. : 118
OnmyTAP2a_#B  : ....GSF............................I.......SACG..--.G....L...A.. : 120
SasaTAP2b_#A  : ....--.........L.................K...H.S...SP.G...--G....L...A.. : 119
SasaTAP2b_#B  : ....--.........L.................K...H.S...SP.G...--G....L...A.. : 119
SasaTAP2b_#C  : ....--.........L.................K...H.S...SP.G...--G....L...A.. : 119
OnmyTAP2b_#A  : .V..--.........L.................Q...H.S...STHG...--G....L...A.. : 118
OnmyTAP2b_#B  : .V..--.........L.................Q...H.S...STHG...--G....L...A.. : 118
OnkiTAP2b     : .V..--.........L....T............Q...H.S...STHG...--G....L...A.. : 119
EsluTAP2NW80  : TV.VS--.......I.....T...L.....V....I.H.S.L.SA.GP..--G.R..LV..... : 117
EsluTAP2      : SV.VT--..E....I......A..L.V........I.H.R.L.SSCGP..--G.R..LV..... : 118
OrlaTAP2_cab  : FTVQ--VI..DL...LI.YITA.SL.QA.F.S.SRT.H.E--DSLCGRAA--DARC.LMF.G.. : 108
OrlaTAP2_HN1  : FTVQ--VI..DL..ALI.YITA.SL.QA.F.S.SRT.H.E--DSLCGRAA--DARC.LMF.G.. : 108
OrlaTAP2_HdrR : FTVQ--VI..DL...LI.YITG.SL.QA.F.S.SRT.H.E--DSLCGRAA--DARC.LMF.G.. : 108
DareTAP2a_19B : AG.T--.SKDAAD.LIR..IT....IGS.......AMFNNYRLD--------KFTV.LVG.... : 108
DareTAP2d_19D : CFLRG----SWREAAVQ.CV..S.L.C.M..S.QTL.INTQ..NWSGC.S--C.GKTIISAA.T : 111
DareTAP2e_19D : ---------------------------------------------------------------- :   -
XelaTAP2_I    : TV..R-.QDTPWLSSSRMYIFTMSLCQ.L.Q.L..VFISH...LPKSFEH------NLYSHLIV : 106
XelaTAP2_II   : IV.VR-.KCPAWVTSQLTFVLTL.LSI.LHQ.L.FLFSSHV..MHSSFP.------SF.SHLPP : 106
GagaBAG69399  : .AGQ----.LAPRGPPGG-AVLLSLGPAIFL.V.GYVG-LPGAAPVLLAM-AT..WLV.THGT. : 109
GagaAEE25620  : .AGQ----.LAPRGPRGA-AVLLSLGPAIFL.L.GYVG-LPGAAPVLLAM-AT..WLV.THGT. : 109
GagaBAG69412  : .AGQ----.LAPRGPHGA-AVLLSLGPAIFL.L.GYVG-LPGAAPVLLAM-AT..WLV.THGT. : 109
GagaBAG69426  : .AGQ----.LAPRGPHGA-AVLLSLGPAIFL.L.GYVG-LPGAAPVLLAM-AT..WLV.THGT. : 109
Rat  TAP2_I   : ..LK----V.GLLRLVGTF.PLL.LTT.LFFSL.ALVGSTMSTS.L.VAS-A.WGWLLAGY... : 109
Rat  TAP2_II  : ..LK----V.GLLRLVGTF.PLL.LTN.LFFSL.ALVGSTMSTS.V.VAS-A.WGWLLADYG.V : 109
Human  TAP2   : ..LK----.RGLLGFVGTL.LPL.LAT.LTVSL.ALVA.A.RAPPA.VAS-APW.WLLVGYG.. : 109
                              60         *         80         *         100

OnkiTAP2a     : AAAALFWETTFPDSNGESNG---KQKKQKARVLFMRVLIFYRPDILLLVGAFIFLSLAVLC--- : 179
SasaTAP2a_#A  : ....................---...T...........Y.....T................--- : 179
SasaTAP2a_#B  : ....................---...T...........Y.....T................--- : 179
SasaTAP2a_#C  : ....................---...T...........Y.....T................--- : 179
OnmyTAP2a_#A  : ........K...........---.E.T...........H.....T................--- : 176
OnmyTAP2a_#B  : ....................---.E.T...........H.....T.............A..--- : 178
SasaTAP2b_#A  : ........K.....KE....---.E.T....A......Y.....T..........A....S--- : 177
SasaTAP2b_#B  : ........K.....KE....---.E.T....A......Y.....T..........A....S--- : 177
SasaTAP2b_#C  : ........K.....KE....---.E.T....A......Y.....T..........A....S--- : 177
OnmyTAP2b_#A  : ........K.....SE....---.E.T...........Y.....T................--- : 176
OnmyTAP2b_#B  : ........K.....SE....---.E.T...........Y.....T................KYF : 179
OnkiTAP2b     : ........K......E....---.E.T...........Y.....T................--- : 177
EsluTAP2NW80  : ....................KEK.E.....L....K..R..K....F.T............--- : 178
EsluTAP2      : .T..F..............---E.E.........L..VR.CKH.S.I.IC...........--- : 176
OrlaTAP2_cab  : LG........I..ADDAAET---.EE...S........RLFK..Y...LAGLL........--- : 166
OrlaTAP2_HN1  : LG........I..ADEAAET---.EE...S........RLFK..Y...LAGLL........--- : 166
OrlaTAP2_HdrR : LG........I..ADDAAET---.EE...S........RLFK..C...LAGLL..C.....--- : 166
DareTAP2a_19B : .L.C....I.L..T.E....---.ER........I..IRL....YI..F...V..A.....--- : 166
DareTAP2d_19D : VL.C....VS.S.T.RKAESSESVE..E.N.A.....VRYSK..VA..S...V......I.--- : 172
DareTAP2e_19D : --------------METADS--------RHLKTIF.L.SLS...L.PIC...L..VA..IG--- :  39
XelaTAP2_I    : FLSCML.DLL..YIFPKKTIKEEDNESK.E..N.I.L.KYSK..W.Q.S...SC.T..LIG--- : 167
XelaTAP2_II   : IF.CFL.DLVS.KLLPK.TEGEANN.-KRE.DN.I.LVKYSI..W.P.C...V..T..LIG--- : 166
GagaBAG69399  : VV.L.T.SLLV.TVATG-------A.EAE.W.PLR.L.ALAW.EWPF.GC..L..A..A.G--- : 163
GagaAEE25620  : VV.L.T.SLLV.TVATG-------A.EAE.W.PLR.L.ALAW.EWPF.GC..L..A..A.G--- : 163
GagaBAG69412  : VV.L.T.SLLV.TVATG-------A.EAE.W.PLR.L.ALAW.EWPF.GC..L..A..A.G--- : 163
GagaBAG69426  : VV.L.T.SLLV.TVATG-------A.EAE.W.PLR.L.ALAW.EWPF.GC..L..A..A.G--- : 163
Rat  TAP2_I   : .LSLAV.AVLS.AGAQ.KE-----PGQENN.A.MI.L.RLSK..LPF.IV.....AM..WG--- : 165
Rat  TAP2_II  : .LSLAV.AVLS.AGAQ.KE-----PGQENN.A.MI.L.RLSK..LPF.IV.....AM..WW--- : 165
Human  TAP2   : GLSWSL.AVLS.PGAQ.KE-----.DQVNNK..MW.L.KLS...LP...A..F..V....G--- : 165
                *         120         *         140         *         160
```

```
OnkiTAP2a     : -EMFIPFYTGKVIDILGTQYKWNNFLTAIILMGLYSLGSSFSAGCRGGLFMCAINSFTCRMKVK : 242
SasaTAP2a_#A  : -..............................................................E : 242
SasaTAP2a_#B  : -..............................................................E : 242
SasaTAP2a_#C  : -..............................................................E : 242
OnmyTAP2a_#A  : -.........................................................S....E : 239
OnmyTAP2a_#B  : -.........................................................S....E : 241
SasaTAP2b_#A  : -.T.............AS.....D.......................................E : 240
SasaTAP2b_#B  : -.T.............AS.....D.......................................E : 240
SasaTAP2b_#C  : -.T.............AS.....D.......................................E : 240
OnmyTAP2b_#A  : -...............AS.....D..................................S....E : 239
OnmyTAP2b_#B  : I...............AS.....D..................................S....E : 243
OnkiTAP2b     : -...............AS.....D..................................S.I..E : 240
EsluTAP2NW80  : -Q.C.............A....KD.R....F..FF......C.....................Q : 241
EsluTAP2      : -.....I...........K.M..D.R.V..F...C.........................I..Q : 239
OrlaTAP2_cab  : -.........R......SHFEQ.E..S.LLF.....V...V.......VL....S......... : 229
OrlaTAP2_HN1  : -.........R......SHFEQ.E..S.LLF.....V...V.......VL....S......... : 229
OrlaTAP2_HdrR : -.........R......SHFEQ.E..S.LLF.....V...V.......VL....S......... : 229
DareTAP2a_19B : -.....L...E......SH.Q.D..RS...F...F.........................V..Q : 229
DareTAP2d_19D : -D.C......L......EH.QP.S.MS..F....L.....L.S.L.......TLSRLNK.VRLM : 235
DareTAP2e_19D : -D..M.AF...I..A.NSTFDHGT.TS..FF...T...G............LT.SRL.K.VRER : 102
XelaTAP2_I    : -.L.V.Y...R.....S.K.NESV.ME..LF.AIF.IT...F..S......FSHS.L.Q.LRLL : 230
XelaTAP2_II   : -.....Y.M.R.....S.N.NERE..V.MFF.ATF.IT..IFS........FSLARL.R.LRLL : 229
GagaBAG69399  : -.TSV.YC..RAL.V.RQGDGLAA.TA.VG..C.A.AS..LF........TFIRFR.VL.TRDQ : 226
GagaAEE25620  : -.TSV.YC..RAL.V.RQGDGLAA.TA.VG..C.A.AS..LF........TFIRFR.VL.TRDQ : 226
GagaBAG69412  : -.TSV.YC..RAL.V.RQGDGLAA.TA.VG..C.A.AS..LF........TFIGFR.VL.TRDQ : 226
GagaBAG69426  : -.TSV.YC..RAL.V.RQGDGLAA.TA.VG..C.A.AS..LF........TFIRFR.IL.TRDQ : 226
Rat_TAP2_I    : -.TL..H.S.R......GDFDPDA.AS..FF.C.F.V...L.......S.LFTMSRINL.IREQ : 228
Rat_TAP2_II   : -.....H.S.R......GDFDPDA.AS..FF.C.F.V...L.......S.LF.ESRINL.IREQ : 228
Human_TAP2    : -.TL..H.S.R......GDFDPHA.AS..FF.C.F.F...L.......C.TYTMSRINL.IREQ : 228
                     *         180       *        200         *       220

OnkiTAP2a     : LFGALVKQEIGFFETIKT--GDITSRLSTDTTLMGRAVALNVNVLLRTLIKTVGMLSLMMSLSW : 304
SasaTAP2a_#A  : ...............M..--............................................ : 304
SasaTAP2a_#B  : ..................--............................................ : 304
SasaTAP2a_#C  : ..................--............................................ : 304
OnmyTAP2a_#A  : ........D.........--..............A............................. : 301
OnmyTAP2a_#B  : ........D.........--..............A............................. : 303
SasaTAP2b_#A  : ..........S.......--............K.A..L.......................... : 302
SasaTAP2b_#B  : ..........S.......--............K.A..L.......................... : 302
SasaTAP2b_#C  : ..........S.......--............K.A..L.......................... : 302
OnmyTAP2b_#A  : ..........S.......--............K.A................I............ : 301
OnmyTAP2b_#B  : ..........S.......--............K.A................I............ : 305
OnkiTAP2b     : ........D.S.......--............K.A................I............ : 302
EsluTAP2NW80  : ...T...........N.L--............................A..........T.... : 303
EsluTAP2      : ..................--..V.......APK...............A............... : 301
OrlaTAP2_cab  : ..E..T............--.E......K.....A.T.C..A......F......I........ : 291
OrlaTAP2_HN1  : ..E..T............--.E......K.....A.T.C..A......F......I........ : 291
OrlaTAP2_HdrR : ..E..T............--.E......K.....A.T.C..A......F......I........ : 291
DareTAP2a_19B : ...S.IR.D.........--.............................V..L...Y..V.... : 291
DareTAP2d_19D : ..N...........DK..--..L.....V..K..SQS..M...I...S...S..I.Y..L.... : 297
DareTAP2e_19D : ..SCF...D.A...KTTK--..L.TC..S.AS..S.SL.A...I...S..M.I.IYCF.IQ.C. : 164
XelaTAP2_I    : ..RS.I..D.A...LT..--........Q..ARVS..I.A...II....V.CI.VY.F.F.... : 292
XelaTAP2_II   : ..RSVI....A...DN.S--.......TH..ARVS.SI.A...IT..M.V.C..VY.F.F.I.. : 291
GagaBAG69399  : ..SS..YRDLA..QKTTA--AELA...T..V..ASNVL...I..M..N.GQVL.LCAF.LG..P : 288
GagaAEE25620  : ..SS..YRDLA..QKTTA--AELA...T..V..ASNVL...I..M..N.GQVL.LCAF.LG..P : 288
GagaBAG69412  : ..SS..YRDLA..QKTTA--AELS...T..V..ASNVLT..I..M..N.GQVL.LCAF.LG..P : 288
GagaBAG69426  : ..SS..YRDLA..QKTTA--AELA...T..V..ASNVL...I..M..N.GQVL.LCAF.LG..P : 288
Rat_TAP2_I    : ..SS.LR.DLA..QET..--.ELN...S..S..S.WLPF.A.I...S.V.V..LYYF.LQV.P : 290
Rat_TAP2_II   : ..SS.LR.DLA..QET..--.ELN...S..S..SQWLS..A.I...S.V.V..LYYF.LQV.P : 290
Human_TAP2    : ..SS.LR.DL...QET..--.ELN...S.....SNWLP..A.....S.V.V..LYGF.L.I.P : 290
                 *         240       *        260         *       280
```

```
OnkiTAP2a     : KLTLLMLMETPITGLLQSVHDNYYQRLSKEVQDSIARANEAAGETVGGIRTVRSFKTEQHEAGR : 368
SasaTAP2a_#A  : ..................................M............................. : 368
SasaTAP2a_#B  : ..................................M............................. : 368
SasaTAP2a_#C  : ..................................M............................. : 368
OnmyTAP2a_#A  : ...........V............L.....................A................. : 365
OnmyTAP2a_#B  : ...........V............L.....................A................. : 367
SasaTAP2b_#A  : .................G.Y....L.....M...M...........A........N..RS.... : 366
SasaTAP2b_#B  : .................G.Y....L.....M...M...........A........N..RS.... : 366
SasaTAP2b_#C  : .................G.Y....L.....M...M...........A........N..RS.... : 366
OnmyTAP2b_#A  : ...........V.....G.Y....L..T......M...........A........N..RS..SH : 365
OnmyTAP2b_#B  : ...........V.....G.Y....L..T......M...........A........N..RS..SH : 369
OnkiTAP2b     : .................G.Y....L..T......M...........A........N..RS...H : 366
EsluTAP2NW80  : ..................IY.S.....A......L.....T.....A............S.... : 367
EsluTAP2      : ..................IY.......D......L.....T.....A............I.... : 365
OrlaTAP2_cab  : .....V.........I.NIY.TK....FQDM.N.M..T.DT.N.V.F..QV....H..T...S. : 355
OrlaTAP2_HN1  : .....V.........I.NIY.TK..S.FQDM.N.M..T.DT.N.V.F...V....H..T...S. : 355
OrlaTAP2_HdrR : .....V.........I.NIY.TK....FQDM.N.M..T.DT.N.V.F...V....H..TQ..S. : 355
DareTAP2a_19B : ...........L.....NIY.TH..K........M.Q..D....A.S.....K.....LG..H. : 355
DareTAP2d_19D : .....TFI.A.LIAIA.KIYNTH.EQ...D....V.....T...A.A.V......CM.PS.... : 361
DareTAP2e_19D : P.A..SAF.S...ITAEKIYNK...G.VSS.KK..VKS.QV...S.YN..A...QGA.TV.QRL : 228
XelaTAP2_I    : Q....TFISA.F.WIT.K.YN..H.K.VQK......SSS.L.R.MFESV...Y..AA.EE..K. : 356
XelaTAP2_II   : Q....TFISS.L.WII.KMYNH.H.D.V.K.......SS.L.K.IIESV...Y..AA.EE..K. : 355
GagaBAG69399  : R..M.A.L.V.LAVTARK.Y.TRH.M.QRA.L.AA.DTGA.VQ.SISS.EM..V.NG.EE.EH. : 352
GagaAEE25620  : R..M.A.L.V.LAVTARK.Y.TRH.M.QRA.L.AA.DTGA.VQ.SISS.EM..V.NG.EE.EH. : 352
GagaBAG69412  : R..M.A.L.V.LAVTARK.Y.TRH.M.QRA.L.AA.DTGA.VQ.SISS.EM..V.NG.EE.EH. : 352
GagaBAG69426  : R..M.A.L.V.LAVTARK.Y.TRH.M.QRA.L.AA.DTGA.VQ.SISS.EM..V.NG.EE.EH. : 352
Rat TAP2_I    : R..F.S.LDL.L.IAAEK.YNPRH.AVL..I..VV.K.GQVVR.A...LQ.....GA.EQ.VS. : 354
Rat TAP2_II   : R..F.S.LDL.L.IAAEK.YNPRH.AVL..I..AV.K.GQVVR.A...LQ.....GA.EQ.FR. : 354
Human TAP2    : R....S.LHM.F.IAAEK.YNTRH.EVLR.I..AV...GQVVR.A...LQ.....GA.E..VC. : 354
                         300      *        320      *        340      *

OnkiTAP2a     : YNDRLMDTHNLKTRRDTVMAVYLLLRRLTAVVMQVAMLYYGRLFIQRGQMTTGNLVSFILYQSD : 432
SasaTAP2a_#A  : ..................R...............................S............. : 432
SasaTAP2a_#B  : ..................R...............................S............. : 432
SasaTAP2a_#C  : ..................R...............................S............. : 432
OnmyTAP2a_#A  : ..................R...........L...................S............. : 429
OnmyTAP2a_#B  : ..................R...........L...................S............. : 431
SasaTAP2b_#A  : .DH...............R......K....L...................S............N : 430
SasaTAP2b_#B  : .DH...............R......K....L...................S............N : 430
SasaTAP2b_#C  : .DH...............R......K....L...................S............N : 430
OnmyTAP2b_#A  : .DH......T........R...V.......L...................S............N : 429
OnmyTAP2b_#B  : .DH......T........R...V.......L...................S............N : 433
OnkiTAP2b     : .DH...............R...V.......L...................S............N : 430
EsluTAP2NW80  : .D........I.S.....R.........I.L........F......Q................. : 431
EsluTAP2      : .DN.......I.......R.........I.L.T......F....V.Q................. : 429
OrlaTAP2_cab  : .DK...E..S..K.....R.I...A....GLA...FI.........K................. : 419
OrlaTAP2_HN1  : .DK...E..S..K.....R.I...A....GLA...FI.........K................. : 419
OrlaTAP2_HdrR : .DK...E..S..K.....R.I...A....GLA...FI.........K................. : 419
DareTAP2a_19B : .DG...E...........R.I...I..M.ELG.K............Y...S...........Q. : 419
DareTAP2d_19D : .DE..T............R.....V...KSLG...L...C..QL.KS.A.S...........G. : 425
DareTAP2e_19D : .DTS.EE..YF.IH..S.R.T...FM..FQLC.R.LI.W..HQM.TS....P..........ME : 292
XelaTAP2_I    : .EKS.RE.Y..Q.C..F.R.L...VIK.INLST...L..C.QQL.HS.NISI.KM.......ME : 420
XelaTAP2_II   : .EKS.RE..V.Q....F.R.L...VT..VNLGP.IT.....QIL.RS.FISS.KM.......ME : 419
GagaBAG69399  : .SQV.DR.LR.RDQ...ER.IF..IQ.VLQLAV.ALV..C.HQQLRE.TL.A.S..A.....TK : 416
GagaAEE25620  : .SQV.DR.LR.RDQ...ER.IF..IQ.VLQLAV.ALV..C.HQQLRE.TL.A.S..A.....TK : 416
GagaBAG69412  : .SQV.DR.LR.RDQ...ER.IF..IQ.VLQLAV.ALV..C.HQQLRE.TL.A.S..A.....TN : 416
GagaBAG69426  : .SQV.DR.LR.RDQ...ER.IF..I..VLQLAV.ALV..C.HQQLRE.TL.A.S..A.....TK : 416
Rat TAP2_I    : .KEA.ERCRQ.WW...LEKEL..VI..VM.LG...LI.NC.VQQ.LA.EV.R.G.L..L...EE : 418
Rat TAP2_II   : .KEA.ERCRQ.WW...LEKSL..VIQ.VM.LG...LI.NV.VQQ.LA.EV.R.G.L..L...EE : 418
Human TAP2    : .KEA.EQCRQ.YW...LER.L...V..VLHLGV.ML..SC.LQQM.D.EL.Q.S.L..MI..ES : 418
                     360      *        380      *        400      *
```

```
OnkiTAP2a      : LADNIRTLIYIFGDMLNSVGAAGKVFEYLDREPQVSTKGTLQPETLTGHVQFHNLSFSYPTRQ- : 495
SasaTAP2a_#A   : ..................................................H.N..........- : 495
SasaTAP2a_#B   : ..................................................H.N..........- : 495
SasaTAP2a_#C   : ..................................................H.N..........- : 495
OnmyTAP2a_#A   : ...............................................................- : 492
OnmyTAP2a_#B   : ...............................................................- : 494
SasaTAP2b_#A   : .GA...............................................H............- : 493
SasaTAP2b_#B   : .GA...............................................H............- : 493
SasaTAP2b_#C   : .GA...............................................H............- : 493
OnmyTAP2b_#A   : .GT...............................................H............- : 492
OnmyTAP2b_#B   : .GT...............................................H............- : 496
OnkiTAP2b      : .GT.................................A.............H............- : 493
EsluTAP2NW80   : .............................E.......N...........IF..........HK- : 494
EsluTAP2       : ..........VY.....................H...............IC............- : 492
OrlaTAP2_cab   : .G......T..........V...........T..I..D.K.K.DEMK...TYCH...A..SNP- : 482
OrlaTAP2_HN1   : .G......T..........V...........K..I..D.K.K.DEMK...TYRH...A..SNP- : 482
OrlaTAP2_HdrR  : .GV.....T..........V...........T..I..D.K.K.DEMK...TYRH...A..SNP- : 482
DareTAP2a_19B  : .G..........................Q..KSE..ID.N.M.KD.K...K.QK.T....R.P- : 482
DareTAP2d_19D  : .GSY....V.MYS.........A........K.L.D.D.S.H.K..A.Q.H.K..T.F..S.P- : 488
DareTAP2e_19D  : IGGHVQ..VELHI.LIE.LES.D....FM.HK.S.LS-.D.A.DQ.K...M.K.I....SSCP- : 354
XelaTAP2_I_    : SG.Y....VHMLSEVTH.A..GH...H...QV.E..NT.K.C.DN.R.QFE.K.VT....S.P- : 483
XelaTAP2_II    : SNSY....VHMLSEITH.A...E...Q....K.K..AS.C.C.DK.Q.RFE.R.VT....S.P- : 482
GagaBAG69399   : AGSCVQA.A.SY..L.SNAV..C...D...W.RP.GAG..YV.TR.R..IT..RV..A....P- : 479
GagaAEE25620   : AGSCVQA.A.SY..L.SNAV..C...D...W.RP.GAG..YV.TR.R..IT..RV..A....P- : 479
GagaBAG69412   : AGSCVQA.A.SY..L.SNAA..C...D...W.RA.GAG..YM.TR.R..IT..RV..A....P- : 479
GagaBAG69426   : AGSCVQA.A.SY..L.SNAA..C...D..NW.RA.GAA..YM.TR.R...T..RV..A....P- : 479
Rat TAP2_I     : VGHHV.N.V.MY...SN....E...S....R.NLPKP...A.PRVE.R.E.QDV.....S.P- : 481
Rat TAP2_II    : VGHHVQN.V.MY...SN....E...S....R.NLPNP...A.PR.E.R.E.QDV.....S.P- : 481
Human TAP2     : VGSYVQ..V..Y....SN....E...S.M..Q.NLPSP...A.T..Q.V.K.QDV..A..N.P- : 481
                   420      *         440       *         460       *         480

OnkiTAP2a      : ERKVLQGFSLELRPGQLTALVGPSGGGKSTCVSLLERFYQPQQGEILLDGLPLQSYQHHYLHRK : 559
SasaTAP2a_#A   : ................................................................ : 559
SasaTAP2a_#B   : ................................................................ : 559
SasaTAP2a_#C   : ................................................................ : 559
OnmyTAP2a_#A   : ................................................................ : 556
OnmyTAP2a_#B   : ..............................................................K. : 558
SasaTAP2b_#A   : G.................................................Q..H.......... : 557
SasaTAP2b_#B   : G.................................................Q..H.......... : 557
SasaTAP2b_#C   : G.................................................Q..H.......... : 557
OnmyTAP2b_#A   : ..................................................Q..H.......... : 556
OnmyTAP2b_#B   : ..................................................Q..H.......... : 560
OnkiTAP2b      : ..................................................Q..H.......... : 557
EsluTAP2NW80   : ...I..D.....K..K.................................EQ...N......... : 558
EsluTAP2       : .H.I..D.....K..K..................................Q............. : 556
OrlaTAP2_cab   : S.T...EL....K...M........E.........Q...E..D.......N..R..D...F.K. : 546
OrlaTAP2_HN1   : SKT...EL....K...M........E.........Q...E..D.......N..R..D...F.K. : 546
OrlaTAP2_HdrR  : S.T...EL....K...M........E.........Q...E..N.......N..R..D...F.K. : 546
DareTAP2a_19B  : DHN..KD.....K...I.....M.....................T.....K...D...K...S. : 546
DareTAP2d_19D  : DQAA.KD.....K...M.....M.....................Q.....Q...K...K...Q. : 552
DareTAP2e_19D  : DK...ENV.F.MK..TI.....V.........A..KHL.E..S.D.F...R..KE.DPK.F.Q. : 418
XelaTAP2_I     : DAPS..NV.FK.P..SV...........T..........E..I.A.....I..GE.K.Q...S. : 547
XelaTAP2_II    : .FLT.KNV.FA.P..SV...........T..........E..E.D.....KS.KE.K.E...S. : 546
GagaBAG69399   : ..L...DVTF....SEV...A.LN.S......A......E.GA..V....V..RD.E.R....Q : 543
GagaAEE25620   : ..L...DVTF....SEV...A.LN.S......A......E.GA..V....V..RD.E.R....Q : 543
GagaBAG69412   : ..L...DVTF.....EV...A.LN.S......A......E.GA..V....V..RD.E.R....Q : 543
GagaBAG69426   : ..L...DVTF.....EV...A.LN.S......A......E.GA..V....V..RD.E.R....Q : 543
Rat TAP2_I     : .KP....LTFT.H..KV......N.S....VAA..QNL...TG.KV....E..VQ.D......Q : 545
Rat TAP2_II    : .KP....LTFT.H..KV......N.S....VAA..QNL...TG.QL....E..VQ.D......Q : 545
Human TAP2     : D.P..K.LTFT....EV......N.S....VAA..QNL...TG.QV...EK.ISQ.E.C...SQ : 545
                         *         500      *         520       *         540
```

```
OnkiTAP2a     : IAMVGQEPVLFSGSVKDNIAYGLADCSLERVQEAARRANAHSFISQLEKGYDTDVGERGGQLSG : 623
SasaTAP2a_#A  : ..............I................................................. : 623
SasaTAP2a_#B  : ..............I................................................. : 623
SasaTAP2a_#C  : ..............I................................................. : 623
OnmyTAP2a_#A  : V.............I..............................H.................. : 620
OnmyTAP2a_#B  : V.............I..............................H.................. : 622
SasaTAP2b_#A  : V.............I..............................................M.. : 621
SasaTAP2b_#B  : V.............I..............................................M.. : 621
SasaTAP2b_#C  : V.............I..............................................M.. : 621
OnmyTAP2b_#A  : V.............I..............................................M.. : 620
OnmyTAP2b_#B  : V.............I..............................................M.. : 624
OnkiTAP2b     : V.............I..............................................M.. : 621
EsluTAP2NW80  : V.............I...x....KE..........x....QG..Rx.....Y.......N.... : 622
EsluTAP2      : V.............I........K.................G...K.....Y.......N.... : 620
OrlaTAP2_cab  : VTA.S.....L...IR...T...SN.TM.EIE...CK....D..MK.DQ....E...G....AK : 610
OrlaTAP2_HN1  : VTA.S.....L...IR...T...SN..M.EIK...CK....D..MK.DQ....E...G....AK : 610
OrlaTAP2_HdrR : VTA.S.....L...IR...T...SN..M.EIK...CK....D..MK.DQ....E...G....AK : 610
DareTAP2a_19B : V............T.R.......QG..M...K...SK........K.............NL... : 610
DareTAP2d_19D : V.....D.........R......T.YDQKK.ED..KE....D..CR...T.........CL..A : 616
DareTAP2e_19D : V.V.S...E..AR.IQK..T...DSY.EDM..T.VKQ..IN..VLS..D..N.E......H..V : 482
XelaTAP2_I    : V.L.A......A.TIRE..G...K.I.EKQLK....K.K.DT...EM..L.....LDS.A...A : 611
XelaTAP2_II   : ..L.A......A...RG..S...QNIPE.QLKA.....K.ED..EGMD.A......DS.A...A : 610
GagaBAG69399  : V.L...........IR......ME..QE.EIIA...A.G.LG...A..Q.FG...........A : 607
GagaAEE25620  : V.L...........IR......ME..EE.EIIA...A.G.LG...A..Q.FG...........A : 607
GagaBAG69412  : V.L...........IR......ME..EE.EIIA...A.G.LG...A..Q.FG...........A : 607
GagaBAG69426  : V.L...........IR......ME..EE.EIIA...A.G.LG...A..Q.FG...........A : 607
Rat TAP2_I    : VVL....................R..EDAQ.MA..QA.C.DD..GEMTN.IN.EI..K.S..AV : 609
Rat TAP2_II   : VVL....................R..EDAQ.MA..QA.C.DD..GEMTN.IN.EI....S..AV : 609
Human TAP2    : VVS............RN......QS.EDDK.MA..QA.H.DD..QEM.H.IY.....K.S..AA : 609
                    *         560       *         580       *         600

OnkiTAP2a     : GEKQRIAIARALIREPQVLILDEVTSALDTESEHMVQEALA------SCPSQTLLVIAHRLKTI : 681
SasaTAP2a_#A  : .........................................------................. : 681
SasaTAP2a_#B  : .........................................------................. : 681
SasaTAP2a_#C  : .........................................------................. : 681
OnmyTAP2a_#A  : .........................................------................. : 678
OnmyTAP2a_#B  : .........................................------................. : 680
SasaTAP2b_#A  : .........................................------................. : 679
SasaTAP2b_#B  : .........................................------................. : 679
SasaTAP2b_#C  : .........................................------................. : 679
OnmyTAP2b_#A  : ........................................T------................. : 678
OnmyTAP2b_#B  : ....................................RHPSTHKLECF.L............... : 688
OnkiTAP2b     : .........................................------................. : 679
EsluTAP2NW80  : ...................................I.....------.......V......... : 680
EsluTAP2      : .........................................------.......V......... : 678
OrlaTAP2_cab  : S.R..G........K........I..S..I.N.NK.LQ..-------NR.N...V....S.... : 668
OrlaTAP2_HN1  : S.R..G........K........I..S..I.N.NK.LQ..-------N..N...V....S.... : 668
OrlaTAP2_HdrR : S.R..G........K........I..S..I.N.NK.LQ..-------NR.N...V....S.... : 668
DareTAP2a_19B : ..........................S......Q...Q..S------C..T............. : 668
DareTAP2d_19D : .Q............Q..I......S.S......K...D..-------RR.N............. : 674
DareTAP2e_19D : ..............Q...........S....N.K.I.N..-------CFAT..R.IV....... : 540
XelaTAP2_I    : .Q...V.L....A.K.KL.....AS.C..A.T..EI.QS.Q------TL.DVS..I.....R.. : 669
XelaTAP2_II   : .Q...V.L....A.K.KL.....AS.C..A.T..EI.KS.Q------.IEDLS..I.....R.V : 668
GagaBAG69399  : .Q..........V.R.T......A.....GD.DA.L.QWVR------NGGDR.V.L.T.QPRML : 665
GagaAEE25620  : .Q..........V.R.T......A.....GD.DA.L.QWVR------NGGDR.V.L.T.QPRML : 665
GagaBAG69412  : .Q..........V.R.T......A.....GD.DA.L.QWVR------NGGDR.V.L.T.QPRML : 665
GagaBAG69426  : .Q..........V.H.T......A.....GD.DA.L.QWVR------NGGDR.V.L.T.QPRML : 665
Rat TAP2_I    : .Q...L......V.N.R......A.....A.C.QAL.TWR-------.QEDR.M.......H.V : 666
Rat TAP2_II   : .Q...L......V.N.R......A.....A.C.QAL.TWR-------.QEDR.M.......H.V : 666
Human TAP2    : .Q...L......V.D.R......A.....VQC.QAL.DWN-------.RGDR.V.......Q.V : 666
                *         620       *         640            *         660
```

```
OnkiTAP2a     : ERADQIILIDQGTVQEQGTHQELMDRKGSYYKLKERLFTEDDVPH------------ : 726
SasaTAP2a_#A  : ..........................................A..------------ : 726
SasaTAP2a_#B  : ..........................................A..------------ : 726
SasaTAP2a_#C  : ..........................................A..------------ : 726
OnmyTAP2a_#A  : .................................R........MS.------------ : 723
OnmyTAP2a_#B  : .................................R........MS.------------ : 725
SasaTAP2b_#A  : ....R.....R.S.L.............G....R........TS.------------ : 724
SasaTAP2b_#B  : ....R.....R.S.L.............G....R........TS.------------ : 724
SasaTAP2b_#C  : ....R.....R.S.L.............G....R........TS.------------ : 724
OnmyTAP2b_#A  : ..............L.............G....R........TS.------------ : 723
OnmyTAP2b_#B  : ..............L.............G....R........TS.------------ : 733
OnkiTAP2b     : ..............L.............G....R........TS.------------ : 724
EsluTAP2NW80  : ..............L....KV..EM........R.....V..D.K------------ : 725
EsluTAP2      : ..............L....KV..EM........R..I.......K------------ : 723
OrlaTAP2_cab  : ......VV.IG.K.K.K........L..V......E....RTN-------------- : 711
OrlaTAP2_HN1  : ......VV.IG.K.K.K........L..V......E....RTN-------------- : 711
OrlaTAP2_HdrR : ......VV.IG.K.K.K........L..V......E....RTN-------------- : 711
DareTAP2a_19B : ......VV..S.ELV.K...E...EK.......R....SD.KTTKQEKEKSDTVKTQ : 725
DareTAP2d_19D : .......V..K.E.L.T.......E...N....R....S.EKEADEKEIKQG----- : 726
DareTAP2e_19D : .K.....V..K.S.S.K...V...KK..I....R.SR.N..SLET------------ : 585
XelaTAP2_I    : QK..N.LVLEG.YLV.....E..VEK.---RECINGSAMGTYCKMEIMMWRQVI--- : 720
XelaTAP2_II   : QK....LVL.G.NLV.....EK.VQKQ.V.HR.LNGTSLQNSGKVN----------- : 714
GagaBAG69399  : .K..RVVVLEH...A.M..PA..RT.G.P.SR.LQHKKKK----------------- : 705
GagaAEE25620  : .K..RVVVLEH...A.M..PA..RT.G.P.SR.LQHKKKK----------------- : 705
GagaBAG69412  : .K..R.VVLEH...A.M..PA..RT.G.P.SR.LQHKKKK----------------- : 705
GagaBAG69426  : .K..R.VVLEH...A.M..PA..RT.G.P.SR.LQHKKKK----------------- : 705
Rat TAP2_I    : QN...VLVLK..QLV.H---DQ.R.EQDV.AH.VQQRLEA----------------- : 703
Rat TAP2_II   : QN...VLVLK..QLV.H---DQ.R.EQDV.AH.VQQRLEA----------------- : 703
Human TAP2    : Q..H..LVLQE.KL.KL---AQ.---------------------------------- : 686
                   *          680
```

References:

1. McConnell SC, *et al.* (2016) Alternative haplotypes of antigen processing genes in zebrafish diverged early in vertebrate evolution. *Proceedings of the National Academy of Sciences of the United States of America* 113(34):E5014-5023.
2. Rudolph MG, *et al.* (2002) Crystal structures of two rat MHC class Ia (RT1-A) molecules that are associated differentially with peptide transporter alleles TAP-A and TAP-B. *Journal of molecular biology* 324(5):975-990.
3. Walker BA, *et al.* (2011) The dominantly expressed class I molecule of the chicken MHC is explained by coevolution with the polymorphic peptide transporter (TAP) genes. *Proceedings of the National Academy of Sciences of the United States of America* 108(20):8396-8401.
4. Ohta Y, *et al.* (2003) Two highly divergent ancient allelic lineages of the transporter associated with antigen processing (TAP) gene in Xenopus: further evidence for co-evolution among MHC class I region genes. *European journal of immunology* 33(11):3017-3027.
5. Nijenhuis M & Hammerling GJ (1996) Multiple regions of the transporter associated with antigen processing (TAP) contribute to its peptide binding site. *Journal of immunology* 157(12):5467-5477.

Additional file 8: Figure S5. Tapasin data

Table of Contents		Page
S5a	Phylogeny of deduced TAPBP amino acid sequences	1
S5b	Alignment of deduced TAPBP amino acid sequences	2

Figure S5a. Phylogeny of deduced TAPBP amino acid sequences

Deduced TAPBP amino acid sequences from salmonid and pike haplotypes are described in Additional file 2: Text S1 and the human reference is shown in the figure. The evolutionary history was inferred by using the Maximum Likelihood method based on the Whelan And Goldman model (1). The tree is drawn to scale, with branch lengths measured in the number of substitutions per site. The percentages of replicate trees in which the associated taxa clustered together in the bootstrap test (100 replicates) are shown next to the branches. All positions with less than 95% site coverage were eliminated. Evolutionary analyses were conducted in MEGA7 (2). Sequences linked to the PSMB8F gene variant are shown with red font. Salmon TAPBPb_50 and _59 refers to the genomic location of these sequences on Atlantic salmon chromosome 14 (see main text Figure 1 and Additional file 1: Figure 1).

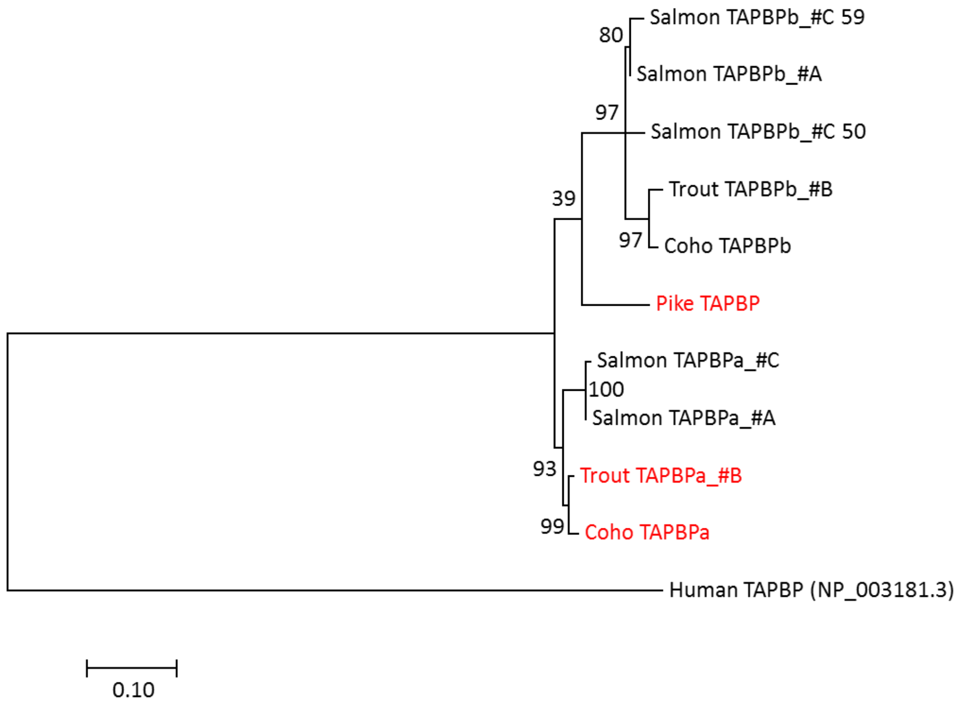

Figure S5b. Amino acid alignment of TAPBP sequences

Salmonid and Northern pike haplotype sequence references can be found in Additional file 2: Text S1. Other sequence references are: Human (Hosa) TAPBP AAG33061.1; Chicken (Gaga) TAPBP1 NP_001029988.2, TAPBP2 CAL47156.1, TAPBP3 CAL47157.1, TAPBP4 CAL47152.1, TAPBP5 CAL47155.1, TAPBP6 CAL47188; Zebrafish (Dare) Haplotype A DareTAPBP_AB strain AL672151:89.363-94.015 CAD58767.1, Haplotype B: DareTAPBP_Tue1 19: 7,119,769-7,124,430 ENSDARG00000079402, DareTAPBP_Tue2 19: 7,147,063-7,151,766 ENSDARG00000045011, DareTAPBP_CG2 GDQH01003123.1; Medaka (Orla) TAPBP_HdrR TAPBP BA000027:361.815-364.809 BAB83851.2, OrlaTAPBP_HN1 TAPBP AB183488:358.533-361.920 BAD93267.2, OrlaTAPBP_cab TAPBP AB450971:194.680-198.000 BAJ07263.1. Reference for the rainbow trout TAPBP sequence is (3), for zebrafish TAPBP sequences (4), medaka TAPBP sequences (5-7) and chicken TAPBP polymorphism (8). Numbering is according to human TAPBP. Residues known to interact with MHCI are indicated in cyan [TN3-TN6] while yellow shading shows human cysteine interacting with ERp57, lysine interacting with TAP and ER retention signal (9). Glycosylation sites are underlined. Sequences linked to the PSMB8F gene variant are shown with red font.

```
SasaTAPBPa_#C  : M-ANISTIYKLSFLAFTYFIHVY---GTSCP----VLECWFVQEKPGRGGGFPAAMIQEKSLL :  55
SasaTAPBPa_#A  : .-.....................---.....----............................ :  55
OnmyTAPBPa_#B  : .-..................Q..---.....----............................ :  55
OnkiTAPBPa     : .-..................Q..---.....----.........................A.. :  55
SasaTAPBPb_#C  : .-T.....LQ-.....S..M...---.A...----...........--....H...S...... :  52
SasaTAPBPb_#A  : .-T.....LQ-.....S..M...---.A...----...........--....H...S...... :  52
OnmyTAPBPb     : -------------------MD..---.....----...........--....H...S...... :  35
OnkiTAPBPb     : .-T...I.LQ-.....S.VM...---.A...----...........--....H...S...... :  52
EsluTAPBP      : .-.X........II..I..TN..---.....----.........R...........S...... :  55
DareTAPBP_AB   : .-SD...VF.I.VI...L.YGSC---.SG..----............H...LSTP.S.....I :  55
DareTAPBP_tue1 : .-SGF.....I.IIV..LCFG.C---.-A..----............H...LSTP.S.....M :  54
DareTAPBP_tue2 : .-SE......I.VF...LCFG.C---.SA..----............H...LSTP.S.....M :  55
DareTAPBP_CG2  : .-SD...VF.I.VI...L.HGF.---.SA..----............H...LSTP.S.....M :  55
OrlaTAPBP_HdrR : .-TDF.......LV.LWC...ACSSSSS...----L.........V.....LAG.TT...T.. :  58
OrlaTAPBP_cab  : .-TGF......YLV.LWC..NACSSSSS...----L.........V.....LAG.TT...T.. :  58
OrlaTAPBP_HN1  : .-TDF.......LV.LWC...AC---SS...----L.........V.....LAG.TT...T.. :  55
GagaTAPBP1     : .A.GLRL-----L..GLCWSQFRVEDAA.P.PPPAPVR.ALL-.GV..R..L.GGGNARPA.. :  57
GagaTAPBP2     : .A.GLRL-----L..GLCWSQFRVEDAA.P.PPPAPVR.ALL-.GV.....L.GGGNARPA.. :  57
GagaTAPBP3     : .A.GLRL-----L..GLCWFQFRVEDAA.P.PPPAPVR.ALL-.GV..R..L.GGGNARPA.. :  57
GagaTAPBP4     : .A.GLRL-----L..GLCWSQFRVEDAA.P.PPPAPVR.ALL-.GV.....L.GGGNARPA.. :  57
GagaTAPBP5     : .A.GLRL-----L..GLCWSQFRVEDAA.P.PPPAPVR.ALL-.GV.....L.GGGNARPA.. :  57
GagaTAPBP6     : .A.GLRL-----L..GLCWSQFRVEDAA.P.PPPAPVR.ALL-.GV.....L.GGGNARPA.. :  57
HosaTAPBP      : .-KSL.L-----L..------.ALGLA.AVSAGPA.I.....EDAS.K.------LAKRPGA. :  45
                                      *        20         *              40
                                                           TN4

SasaTAPBPa_#C  : YINTDPESEETKSQQGPSADINHDRVYYVTDPAAILCSSSLHPPEGSVHKPQCEINPFMPQPS : 118
SasaTAPBPa_#A  : ............................................................... : 118
OnmyTAPBPa_#B  : .........Q...........S......I.................................. : 118
OnkiTAPBPa     : .........Q.....R.....S......I..............................T... : 118
SasaTAPBPb_#C  : ......D....R.K..L.T.--............T.....M.......Q.............. : 113
SasaTAPBPb_#A  : ......D....R.K..L.T.--............T.....M.......Q.............. : 113
OnmyTAPBPb     : ......D....R.K...PT.--............T.............Q.S............ :  96
OnkiTAPBPb     : ......D....R.K...PT.--............T.............Q.S............ : 113
EsluTAPBP      : .......WKRPE...R..T...X..L..I.....TF............Q.............. : 118
DareTAPBP_AB   : F.R.EAY...I.TELH.P...SSS.........GTF..AA.N..K...N..K........HA. : 118
DareTAPBP_tue1 : F.R.EAF...I.TELH.P...SSS.........GTF..AA.N..K...N..K........HA. : 117
DareTAPBP_tue2 : F.R.EAY...I.TELH.P...SSS.........GTF..AA.N..K...N..K........HA. : 118
DareTAPBP_CG2  : F.R.EAY.....AELH.P...SSS.........GTF..AA.N..K...N..K........HA. : 118
OrlaTAPBP_HdrR : H.RK.ERDDGAA.SRA.-S...P..I.VI....ET..HR.FN..R..IK.....V...L.... : 120
OrlaTAPBP_cab  : H.RK.EHDDGAA.SRA.-S...P..I.VI....ET..HR.FN..R..IK.....V...L.... : 120
OrlaTAPBP_HN1  : H.RK.ERDDGAA.SRA.-S...P..I.VI....ET..HR.FN..R..IK.....V...Q.... : 117
GagaTAPBP1     : RFGG.-------AETP.EPGPEPEVTFN.S..WGT.TPLGV-..R---TP.S..L..TN..TG : 109
GagaTAPBP2     : RFGG.-------AETP.EPGPEPEVTFN.S..WGT.TPLGV-..R---TP.S..L..TN..TG : 109
GagaTAPBP3     : RFGG.-------AETP.EPGPEPEVTFN.S..WGT.TPLGV-..R---TP.S..L..TN..TG : 109
GagaTAPBP4     : RFGG.-------AETP.EPGPEPEVTFN.S..WGT.TPLGV-..R---TP.S..L..TN..TG : 109
GagaTAPBP5     : RFGG.-------AETP.EPGPEPEVTFN.S..WGT.TPLGV-..R---TP.S..L..TN..TG : 109
GagaTAPBP6     : LFGG.-------AETP.EPGPEPEVTFN.S..WGT.TPLGV-..R---TP.S..L..TN..TG : 109
HosaTAPBP      : LLRQG.------GEPP.RP.LDPELYLS.H...GA.QAAFRRY.R.-APA.H..MSR.V.L.A : 101
                     *               60        *         80         *        100
                                                                      TN3
```

```
SasaTAPBPa_#C  : TVQWVVPLTDSAHSPIYLQADWYSAALQGLDGQLGLSSVMRAPTATKEP-------TVVLSVS : 174
SasaTAPBPa_#A  : .................................................-------....... : 174
OnmyTAPBPa_#B  : ....A.............................A..............-------....... : 174
OnkiTAPBPa     : ....A.............................A..............-------....... : 174
SasaTAPBPb_#C  : ....T..................T.....F....R..N.....M.....-------K.L.... : 169
SasaTAPBPb_#A  : ....T..................T.....F....R..N...........-------K.L.... : 169
OnmyTAPBPb     : ....T..................T.....F....R..N.L.........-------K...... : 152
OnkiTAPBPb     : ....T..................T.T...F....R..N.L.........-------K...... : 169
EsluTAPBP      : S...T.S..................S....N...S..............-------A.L.... : 174
DareTAPBP_AB   : M.R.ASA.....Q..V......F.V.A....D..T..NI....S.S...-------K.I.... : 174
DareTAPBP_tue1 : M.R.ASA.....Q..V......F.V.A....E..T..NI....L.S.D.-------K.I.... : 173
DareTAPBP_tue2 : M.R.ASA.....Q..V......F.V.A....E..T..NI....S.S...-------K.I.... : 174
DareTAPBP_CG2  : M.R.ASA.....Q..V......F.V.A....E..T..NI....S.S...-------E.I.... : 174
OrlaTAPBP_HdrR : SLA..RS..EN.F..V......F..SFH.V.E.R.VATIT...SG...H-------S.I..LT : 176
OrlaTAPBP_cab  : SLA..RS..EN.F..V......F..SFHSV.D.R.VATIT...SG...H-------S.I..L. : 176
OrlaTAPBP_HN1  : SLA..RS..EN.F..V......F..SFH.V.DER.VATIT...SG...H-------S.I..L. : 173
GagaTAPBP1     : SDP.SR..HPD.R..PTAGGQ.WV..VG--TP.Y.VTALLQGGMG.EGT----ITAA.A.A.L : 166
GagaTAPBP2     : SDP.SR..HPD.R..PTAGGQ.WV..VG--TP.Y.VTALLQGGMG.EGT----ITAA.A.A.L : 166
GagaTAPBP3     : SDP.SR..HPD.R..PTAGGQ.WV..VG--TP.Y.VTALLQGGMG.EGT----ITAA.A.A.L : 166
GagaTAPBP4     : SDP.SR..HPD.R..PTAGGQ.WV..VG--TP.Y.VTALLQGGMG.EGT----ITAA.A.A.L : 166
GagaTAPBP5     : SDP.SR..HPD.R..PTAGGQ.WV..VG--TP.Y.VTALLQGGMG.EGT----ITAA.A.A.L : 166
GagaTAPBP6     : SDP.SR..HPD.R..PTAGGQ.WV..VG--TP.Y.VTALLQGGMG.ERT----ITAA.A.A.L : 166
HosaTAPBP      : SAK.ASG..PAQNC.RA.DGA.LMVSIS--SPV.S...LL.PQPEPQQEPVLITMA....T.L : 162
                          *         120         *         140         *         160
                              C115 (C95 mature protein) human ERp57 bond

SasaTAPBPa_#C  : SRTPLVRSRLGEPVVLDCGFW-MEATSPLSGS------GFAVEWRYQFRGDGRLVLAYDGKTD : 230
SasaTAPBPa_#A  : .....................-..........------......................... : 230
OnmyTAPBPa_#B  : .....................-A.TS......------......................... : 230
OnkiTAPBPa     : .....................-A..S......------......................... : 230
SasaTAPBPb_#C  : ...FMI...............-VD.S......------......................... : 225
SasaTAPBPb_#A  : ...FMI...............-VD.S......------......................... : 225
OnmyTAPBPb     : ...FMI...............-VD.S......------.......................S. : 208
OnkiTAPBPb     : ...FMI...............-VD.S......------.......................S. : 225
EsluTAPBP      : ....V.......R........-A..S......------..T....................N. : 230
DareTAPBP_AB   : .K..V..C......L......-IDPS...H..------..SI........E..........N. : 230
DareTAPBP_tue1 : .K..V..C......L......-VDPS...H..------..SI........E..........N. : 229
DareTAPBP_tue2 : .K..V..C......L......-IDPS...H..------..SI........E..........N. : 230
DareTAPBP_CG2  : .K..V..C......L......-IDPS...H..------..SI........E..........N. : 230
OrlaTAPBP_HdrR : .T.MTAQA...Q.FT...S..-ADPL....ET------......................... : 232
OrlaTAPBP_cab  : .T.MTAQA...Q..T...S..-ADPL....ET------......................... : 232
OrlaTAPBP_HN1  : .T.MTAQA...Q..T...S..-ADPL....ET------........................E : 229
GagaTAPBP1     : TH..AL.A.V.S.IH.H.A.A-APPS------------P.VL...H.N..A..VL....SS.A : 216
GagaTAPBP2     : TH..TL.A.V.S.IH.H.A.A-APPS------------P.VL...H.N..A..VL....SS.A : 216
GagaTAPBP3     : TH..TL.A.V.S.IH.H.A.A-APPS------------P.VL...H.N..A..VL....SS.A : 216
GagaTAPBP4     : TH..TL.A.V.S.IH.H.A.A-APPS------------S.VL...H.N..A..VL....SS.A : 216
GagaTAPBP5     : TH..TL.A.V.S.IH.H.A.A-APPS------------S.VL...H.N..A..VL....SS.A : 216
GagaTAPBP6     : TH..TL.A.V.S.IH.H.A.A-APPS------------P.VL...H.N..A..VL....SS.A : 216
HosaTAPBP      : TH..AP.V...QDAL..LS.AY.PP..EAAS.LAPGPPP.GL...R.HL.K.H.L..ATPGLN : 225
                         *         180         *         200         *         220
                                                          TN6   TN7

SasaTAPBPa_#C  : RFAETKEKRAGLDFTALHET-------GNASLILQEAQVRHTGTNICTVYLPYLLAQVAVELE : 286
SasaTAPBPa_#A  : ....................-------.................Y.................. : 286
OnmyTAPBPa_#B  : ........G...........-------..........E......Y.................. : 286
OnkiTAPBPa     : ........G...........-------..........E......Y...A.............. : 286
SasaTAPBPb_#C  : ....IQ.EG...........-------..........E...S..Y.................. : 281
SasaTAPBPb_#A  : .....Q.EG...........-------..............S..Y.................. : 281
OnmyTAPBPb     : .....Q.EGV..........-------.D............S..Y.................. : 264
OnkiTAPBPb     : .....Q.EGS..........-------.D............S..Y..M............... : 281
EsluTAPBP      : .L...Q..G.E........R-------..............S..Y........I.X....... : 286
DareTAPBP_AB   : .....S.SG.EM.I.G.YQ.-------.......E.S....S..Y.......H.......D.. : 286
DareTAPBP_tue1 : .....S.SG.EM.I.G.YQ.-------..V....E.S....S..Y.......H.......D.. : 285
DareTAPBP_tue2 : .....S.SG.EM.I.G.YQ.-------.......E.S....S..Y.......H.......D.. : 286
DareTAPBP_CG2  : .....S.SG.EM.I.G.YQ.-------.......E.S....S..Y.......H.......D.. : 286
OrlaTAPBP_HdrR : .L.D.L.EG.T...E....R-------.........TK.Q.S..Y..S.F.........M... : 288
OrlaTAPBP_cab  : .L.D.L.EG.T...E....R-------.........TK.Q.S..Y..S.F.........M... : 288
OrlaTAPBP_HN1  : .L.D.L.DG.T...E....R-------.........TK.Q.S..Y..S.F.........M... : 285
GagaTAPBP1     : .-.PRATPG.E.LLGTRDGD-----GVTAVT.R.AQPSPGDE..Y..S.F..HGHT.TVLQ.H : 273
GagaTAPBP2     : .-.PRATPG.E.LLGTRDGD-----GVTAVT.R.ARPSPGDE..Y..S.F..HGHT.TVLQ.H : 273
GagaTAPBP3     : .-.PRATPG.E.LLGTRDGD-----GVTAVT.R.AQPSPGDE..Y..S.F..HGHT.TVLQ.H : 273
GagaTAPBP4     : .-.PRATPG.E.LLGTRDGD-----GVTAVT.R.ARPSPGDE..Y..S.F..HGHT.TVLQ.H : 273
GagaTAPBP5     : .-.PRATPG.E.LLGTRDGD-----GVTAVT.R.ARPSPGDE..Y..S.F..HGHT.TVLQ.H : 273
GagaTAPBP6     : .-.PRATPG.E.LLGTRDGD-----GVTAVT.R.ARPSPGDE..Y..S.F..HGHT.TVLQ.H : 273
HosaTAPBP      : GQMPAAQEG.-VA.A.WDDDEPWGPWT..GTFW.PRV.PFQE..YLA.IH....QG..TL..A : 287
                       *         240         *         260         *         280
                                                                    TN5        TN6
                                      Glycosylation site N253 (N233 mature protein)
```

```
SasaTAPBPa_#C  : IVEPPSLSIFPSPLPLSVPGQV--VKVQCEASGFFPL-SLDFHWELT-GPDGKVRP----LGQ : 341
SasaTAPBPa_#A  : ......................VM.............-.........-........----... : 343
OnmyTAPBPa_#B  : ......................--.............-..E.Y....-........----... : 341
OnkiTAPBPa     : ......................--.............-..E.Y....-........----... : 341
SasaTAPBPb_#C  : .................M....--.T........Y..-..E...V..-..G.R.S.----... : 336
SasaTAPBPb_#A  : .................M....--.T........Y..-..E...V..-..G.R.S.----... : 336
OnmyTAPBPb     : ..------------------------------------------------------------- : 266
OnkiTAPBPb     : .................M....--.T........Y..-..E.R.M..-........----... : 336
EsluTAPBP      : ...........X..........--.T........S..-..EL....M-ET..N...----... : 341
DareTAPBP_AB   : .........L......L.....--LS........A.H-T..LG..F.-.A...SLS----... : 341
DareTAPBP_tue1 : .........L......L.....--LS........A.H-T..LG..F.-.A...SLS----... : 340
DareTAPBP_tue2 : .........L......L.....--LS........A.H-T..LG..FI-.A...ALS----... : 341
DareTAPBP_CG2  : .........L......L.....--LS........A.H-T..LG..F.-.A...SLS----... : 341
OrlaTAPBP_HdrR : V........H......A.....--LNI.......V..-PV.LS..FR-.A...S.S----..S : 343
OrlaTAPBP_cab  : V........H......A.....--LNI.......V..-PV.LS..FR-.A...S.S----..S : 343
OrlaTAPBP_HN1  : V........H......A.....--LNI.......V..-PV.LS..FR-.A...S.S----..S : 340
GagaTAPBP1     : VF...KVTLS.KN.-VVA..MS--AELR.HV...Y..-DVTVT.QRRA.SS.TSQSPRDTVMD : 332
GagaTAPBP2     : VF...KVTLS.KN.-VVA..TS--AELR.HV...Y..-DVTVT.QRRT.SS.TS.SPRDTVMD : 332
GagaTAPBP3     : VF...KVTLS.KN.-VVA..TS--AELR.HV...Y..-DVTVT.QRRT.SS.TS.SPRDTVMD : 332
GagaTAPBP4     : VF...KVTLS.KN.-VVA..MS--AELR.HV...Y..-DVTVT.QRRA.GS.TS.SPRDTVMD : 332
GagaTAPBP5     : VF...KVTLS.KN.-VVA..TS--AELR.HV...Y..-DVTVT.QRRA.GS.TSQSPRDTVMD : 332
GagaTAPBP6     : VF...KVTLS.KN.-VVA..MS--AELR.HV...Y..-DVTVT.QRRA.GS.TS.SPRDTVMD : 332
HosaTAPBP      : VYK..KV.LM.AT.ARAA..EA-PPELL.LV.H.Y.SGG.EVE...RG..G.RSQKA---E.. : 346
                   *         300        *         320       *         340

                                                     C362
SasaTAPBPa_#C  : GSVTGHRQGPDNTYSQTSRLELDSAKLDLGRGGEVTCVAVHP---GGTR--RASVTLNVIGIN : 399
SasaTAPBPa_#A  : ..........................................---....--............ : 401
OnmyTAPBPa_#B  : .................T........................---....--..........V. : 399
OnkiTAPBPa     : .................T........................---....--..........V. : 399
SasaTAPBPb_#C  : ...........S.......V.....................R---....--.......IT.VS : 394
SasaTAPBPb_#A  : ...........S.......V....................LR---....--.......IT.VS : 394
OnmyTAPBPb     : ----------------------------------------------.EE--.----------- : 270
OnkiTAPBPb     : ...........S.F...............--..........H---....--.......IA.VS : 392
EsluTAPBP      : ...S.......G.....T..Q.....................---....--..R.......VS : 399
DareTAPBP_AB   : .......RAS.G.F..S.......T..R.A....IS...K.D---....--...AA.....VA : 399
DareTAPBP_tue1 : .......RAS.G.F..S.......T..R.A....IS...K.D---....--...AA.....VA : 398
DareTAPBP_tue2 : ...V...RAS.G.F..S.......T..R.A....IS...K.D---....--...AA.....VG : 399
DareTAPBP_CG2  : .......RAS.G.F..S.......T..R.A....IS...K.D---....--...AA.....VA : 399
OrlaTAPBP_HdrR : ..MS....AW.G....ST..Q..TS.........IS.....R---....--......S...FS : 401
OrlaTAPBP_cab  : ..MS....AW.G....ST..Q..TS.........IS.....R---....--......S...FS : 401
OrlaTAPBP_HN1  : ..MS....AW.G....ST..Q..TS.........IS.....R---....--......S...FS : 398
GagaTAPBP1     : SWTS....AA.G...R.AAAR.IP.RPQ-HH.DVYS..VT.T---ALAKPM.V..R.LLA.TE : 391
GagaTAPBP2     : SWTS....AA.G...R.AAAR.IP.RPQ-HH.DVYS..VT.T---ALAKPM.V..R.LLA.TE : 391
GagaTAPBP3     : SWTS....AA.G...R.AAAR.IP.RPQ-HH.DVYS..VT.I---ALAKPM.V..R.LLA.TE : 391
GagaTAPBP4     : SWTS....AA.G...R.AAAR.IP.RPQ-HH.DVYS..VT.T---ALAKPM.V..R.LLA.TE : 391
GagaTAPBP5     : SWTS....AA.G...R.AAAR.IP.RPQ-HH.DIYS..VT.T---ALAKPM.V..R.LLA.TE : 391
GagaTAPBP6     : SWTS....AA.G...R.AAAR.IP.HPQ-HH.DVYS..VT.T---ALAKPM.V..R.LLA.TE : 391
HosaTAPBP      : RWLSAL.HHS.GSV.LSGH.QPPPVTTE-QH.ARYA.RIH..SLPASG.--S.E...E.A.LS : 406
                    *         360       *          380       *           400

SasaTAPBPa_#C  : GPSIEDSMAMVAVALGLYGLIKIVSWTF-SSGSDDTNSQEK--------------KVK : 442
SasaTAPBPa_#A  : ............................-............--------------... : 444
OnmyTAPBPa_#B  : ............................-..........A.--------------... : 442
OnkiTAPBPa     : ............................-............--------------... : 442
SasaTAPBPb_#C  : A.....Y.........I.....V.....-.....VAYT...--------------... : 437
SasaTAPBPb_#A  : A.....Y.........I.....V.....-.....VAYT...--------------... : 437
OnmyTAPBPb     : ---------------------------------------------------------- :   -
OnkiTAPBPb     : A.....Y.........I....QV.....-.....VA.T...FTHIGVIIILSQLQ... : 449
EsluTAPBP      : A..............C......VI....I....E.AEKKA.----------------- : 440
DareTAPBP_AB   : A..............L...M..FL....S..D.G.SELND.--------------.E- : 442
DareTAPBP_tue1 : A..............L...M..FL....S..D.G.SELND.--------------.E. : 442
DareTAPBP_tue2 : A..............L...M..FF....S.----.AELND.--------------.E. : 439
DareTAPBP_CG2  : A..............L...M..FL....S..D.G.SELND.--------------.E. : 443
OrlaTAPBP_HdrR : S..........G...V......FA....T.....EAAKPN.---------------D. : 444
OrlaTAPBP_cab  : S..........G...V......FA....T.....EAAKPN.---------------D. : 444
OrlaTAPBP_HN1  : S..........G...V......FA....T.....EAAKPN.---------------D. : 441
GagaTAPBP1     : ..HL..ITGLFL..FV.C...R---.LY-PKAARPKEETK.---------------SQ : 430
GagaTAPBP2     : ..HL..ITGLFL..FV.C...R---.LY-PKAARPKEETK.---------------SQ : 430
GagaTAPBP3     : ..HL..ITGLFL..FV.C...R---.LY-PKAAQPKEETK.---------------SQ : 430
GagaTAPBP4     : ..HL..ITGLFL..FV.C...R---.LY-PKAARPKEETK.---------------SQ : 430
GagaTAPBP5     : ..HL..ITGLFL..FV.C...R---.LY-PKAARPKEETK.---------------SQ : 430
GagaTAPBP6     : ..HL..ITGLFL..FV.C...R---.LY-PKAARPKEETK.---------------SQ : 430
HosaTAPBP      : ...L...VGLFLS.FL.L..F.ALG.AA-VYL.TCKD.KK.---------------AE : 448
                    *         420       *          440
                                      TAP accociation  ER retention signal
```

References:

1. Whelan S & Goldman N (2001) A general empirical model of protein evolution derived from multiple protein families using a maximum-likelihood approach. *Molecular biology and evolution* 18(5):691-699.
2. Kumar S, Stecher G, & Tamura K (2016) MEGA7: Molecular Evolutionary Genetics Analysis Version 7.0 for Bigger Datasets. *Molecular biology and evolution* 33(7):1870-1874.
3. Landis ED*, et al.* (2006) Identification and regulatory analysis of rainbow trout tapasin and tapasin-related genes. *Immunogenetics* 58(1):56-69.
4. McConnell SC*, et al.* (2016) Alternative haplotypes of antigen processing genes in zebrafish diverged early in vertebrate evolution. *Proceedings of the National Academy of Sciences of the United States of America* 113(34):E5014-5023.
5. Matsuo M, Asakawa S, Shimizu N, Kimura H, & Nonaka M (2002) Nucleotide sequence of the MHC class I genomic region of a teleost, the medaka (*Oryzias latipes*). *Immunogenetics* 53:930-940.
6. Nonaka MI & Nonaka M (2010) Evolutionary analysis of two classical MHC class I loci of the medaka fish, *Oryzias latipes*: haplotype-specific genomic diversity, locus-specific polymorphisms, and interlocus homogenization. *Immunogenetics* 62(5):319-332.
7. Tsukamoto K*, et al.* (2005) Unprecedented intraspecific diversity of the MHC class I region of a teleost medaka, *Oryzias latipes*. *Immunogenetics* 57:420-431.
8. van Hateren A*, et al.* (2013) A mechanistic basis for the co-evolution of chicken tapasin and major histocompatibility complex class I (MHC I) proteins. *The Journal of biological chemistry* 288(45):32797-32808.
9. Dong G, Wearsch PA, Peaper DR, Cresswell P, & Reinisch KM (2009) Insights into MHC class I peptide loading from the structure of the tapasin-ERp57 thiol oxidoreductase heterodimer. *Immunity* 30(1):21-32.

Additional file 9. Figure S6. Calnexin and calreticulin data

Table of Contents		Page
S6a	Phylogeny of deduced CANX, CALR, CALRL and CLGN amino acid sequences	4
S6b	Alignment of deduced CANX and CLGN amino acid sequences	1
S6c	Alignment of deduced CALR and CALRL amino acid sequences	5

Figure S6a. Phylogeny of deduced CANX, CALR, CALRL and CLGN amino acid sequences

The evolutionary history was inferred by using the Maximum Likelihood method based on the Le and Gascuel 2008 model (1). The tree with the highest log likelihood (-10176.0329) is shown. The percentage of trees in which the associated taxa clustered together is shown next to the branches. The tree is drawn to scale, with branch lengths measured in the number of substitutions per site. The percentage of trees in which the associated taxa clustered together in the bootstrap test (100 replicates) is shown next to the branches. All positions with less than 95% site coverage were eliminated. Evolutionary analyses were conducted in MEGA7 (2). Sequence references are shown in parenthesis and Atlantic salmon sequences are shown using red font. Atlantic salmon sequences are also presented in Additional file 2: Text S1. CALR is calreticulin, CALRL is calreticulin-like, CANX is calnexin and CLGN is calmegin.

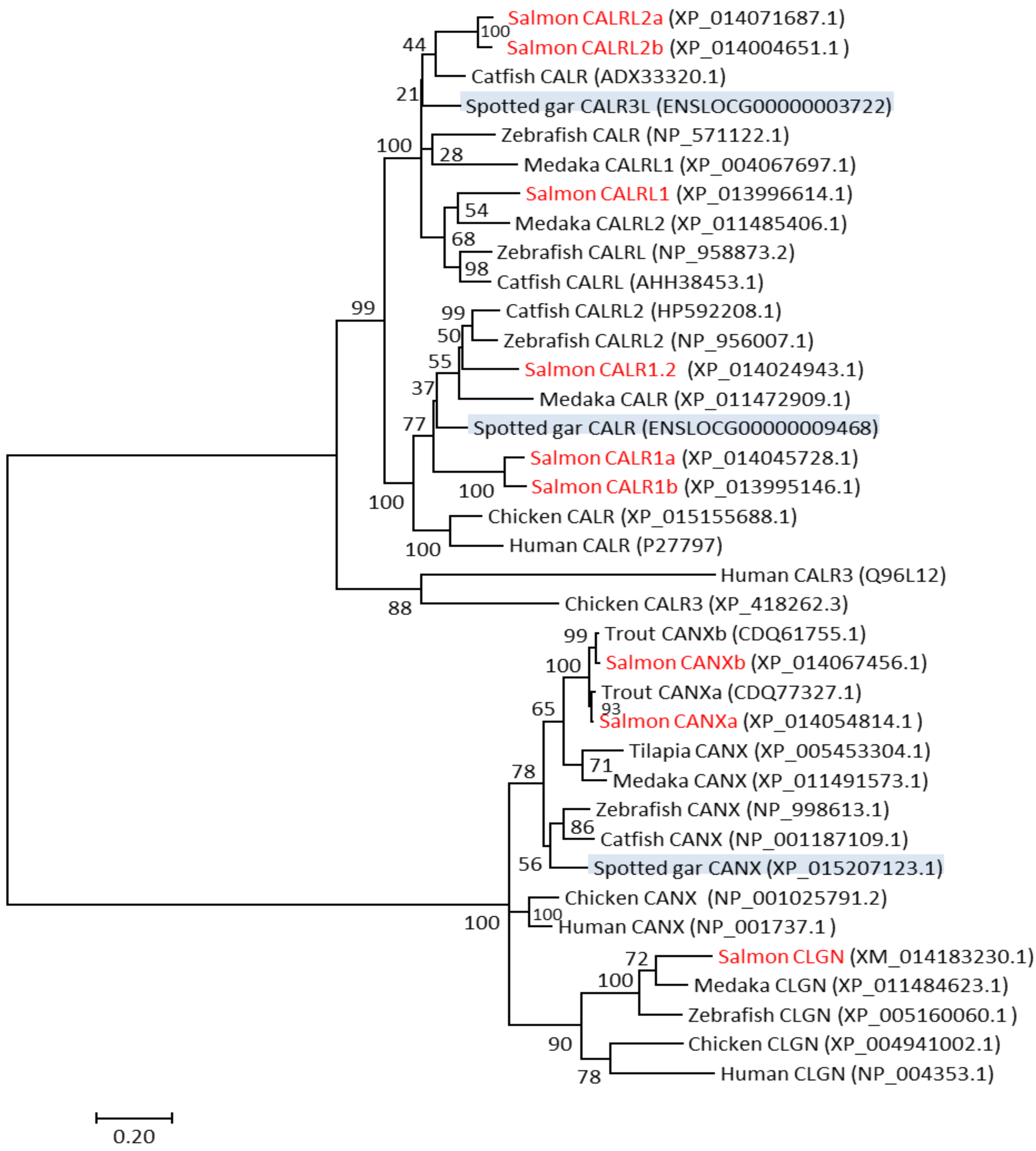

Figure S6b. Alignment of deduced CANX and CLGN amino acid sequences

Sequence references are shown in Fig. S6a. See Fuller et al. (3) for the catfish CANX gene sequence. Residues are color coded according to physiochemical properties and numbering is consecutive. Hosa is human, Gaga is chicken, Sasa is Atlantic salmon, Onmy is rainbow trout, Orla is medaka, Dare is zebrafish, Icpu is catfish and Leoc is Spotted gar. Numbering on top is consecutive. Amino acid residues are colour coded according to physiochemical properties.

```
                         *        20         *        40         *        60
HosaCANX   : --MEGKWL-LC-MLLVLG-TAIVE------AHDGHD-------DD--VIDIEDDLDDVIE---- :  40
GagaCANX   : --..M...-.YVT..A..-.LAAQ------E..VG.-------..GD..........GV.---- :  43
SasaCANXa  : --..LNVR--.VV..AVALCSTLLLTTVAAHQEEEE-PIMELAE.---MGV..E.E.LGL--GE :  54
OnmyCANXa  : --..LNVR--.VV..AV.LCSTLLLTTVAAHQEEEE-PIMELAE.---MGVD.E.E.LGL--GE :  54
SasaCANXb  : --..L.VR--YVL..AV.LWSTLLLTTVTAHQ.EE.EPIVEMGG.---M.V...MEELDH--GE :  55
OnmyCANXb  : --..L.VR--YVL..AV.LWSTLLLTTVTAHQEEE.EPIMEMGG.---M.V..EMEELDH--GE :  55
OrlaCANX   : --.HQRVT-.FLLVAAGLLFLSLA-PVSWAEGGLEE----GLD..---L.V..E..LGLAGTDD :  53
DareCANX   : --..L.MR-..VA..S.SLCLLLM-GPVRAQEEEA.------DME---M.V..AI..MQ----- :  46
IcpuCANX   : --..L.VR-..VL..A.SVCVTLQ---VRAQEEDE.-------E.---VHV....GNLG---DE :  45
LeocCANX   : QT..L..R-..ML..A..W.STLV------QA.SE.-------T.---..V.....VAG.---- :  43
SasaCLGN   : --.KLW.GWA.VL..S.LALSVSQ---AEAQD.----------L.---LEM.EAMVEDMVEDRE :  46
HosaCLGN   : --.HFQAFW..LG..FISI-----------NAEFM.-------..---VET..--------FEE :  33

                       *        80         *       100         *       120
HosaCANX   : -----------EVE-DSKPDTTAPPSSPKVTYKAPVPTGEVYFADSFDRGTLSGWILSKAKKDD :  92
GagaCANX   : -----------.I.-..N.ESST..PA.....R..........VE...K...D........... :  95
SasaCANXa  : --ELLDG----...PEDADTPPG..PA........E.M..HF..E.......DS.V......E. : 112
OnmyCANXa  : --ELLDG----...PEDADTPPG..PA........E.M..HF..E.......DS.V......E. : 112
SasaCANXb  : --ELLDG----...---ADMPPG...V......V.E....HF..E...M...DS.V......E. : 110
OnmyCANXb  : --ELLDG----...---ADMPPG..PV........E....HF..E...M...DS.V......E. : 110
OrlaCANX   : EGEGLEE----.G.-.EA.PAPKT.PV........E.V..HF..E.......D..V.......A : 112
DareCANX   : -----EE----DI.-EEEQKAP....A.T......E.M..H...EA..K...Q..V..Q....G : 100
IcpuCANX   : --ELLDGDG--..DLEDEEKP.PT.AA.T......E.K..HF..E...K...D..V..Q....G : 105
LeocCANX   : -----------.---EEE.KQAS...A.....E...A..DF...E.......D.........EG :  93
SasaCLGN   : DSAEIEGEAEM.G.-AEGEKG.DANV.FQ....T...I.....SET..D.S.DR.QV..TM.E. : 109
HosaCLGN   : N--------------SEEI.VNESEL.SEIK..T.Q.I......ET..S.R.A..V........ :  83

                    *       140         *       160         *       180         *
HosaCANX   : TDDEIAKYDGKWEVEEMKESKLPGDKGLVLMSRAKHHAISAKLNKPFLFDTKPLIVQYEVNFQN : 156
GagaCANX   : ..............QD..DT..........VT........S..S...V.......I........ : 159
SasaCANXa  : I.ED...........D..D...........K..........Q.LR..I.......I.......Q : 176
OnmyCANXa  : I.ED...........D..D...........K..........Q.LR..I...............Q : 176
SasaCANXb  : I.ED...........D..DG..........K..........Q.LR..I...............Q : 174
OnmyCANXb  : I.ED...........D..DG..........K..........Q.LR..I...............Q : 174
OrlaCANX   : A.E.........A.....D...........K..........R.LR..T...............S : 176
DareCANX   : I.ED.............QD...........K.K........L.LR..T...............T : 164
IcpuCANX   : I.ED..............DT..........K..........L.LR..T................ : 169
LeocCANX   : I.E.......Q......VD...........K............L...V...............T : 157
SasaCLGN   : A...........S..QL..N.V...M....K........ASL.DR...RDDA.V.........D : 173
HosaCLGN   : M.E..SI...R..I..L..NQV...R....K..........V.A...I.AD............D : 147

                   200         *       220         *       240         *
HosaCANX   : GIECGGAYVKLLSKTPELNLDQFHDKTPYTIMFGPDKCGEDYKLHFIFRHKNPKTGIYEEKHAK : 220
GagaCANX   : ........................................................K....... : 223
SasaCANXa  : ..D..........Q..D....E.V................................E....... : 240
OnmyCANXa  : ..D..........Q..D....E.V................................E....... : 240
SasaCANXb  : ..D..........Q..D....E.V................................E....... : 238
OnmyCANXb  : ..D..........Q..D....E.V................................E....... : 238
OrlaCANX   : ..D.........TE.........V.R..............................E....... : 240
DareCANX   : ..D..........Q..D.D.EE.V................................EF...... : 228
IcpuCANX   : ..D.....A....QSA.....E.V................................E....... : 233
LeocCANX   : ..D..........Q..D......V................................E....... : 221
SasaCLGN   : ..D.....I....DEDD.D.E..N.R.S......................RS.LNKDL...... : 237
HosaCLGN   : ..D.....I...AD.DD.I.EN.Y...S.I.....................H....VF...... : 211
```

```
                      260         *       280         *       300         *       320
HosaCANX   : RPDADLKTYFTDKKTHLYTLILNPDNSFEILVDQSVVNSGNLLNDMTPPVNPSREIEDPEDRKP : 284
GagaCANX   : ..................................M...........S.....P......N.Q.. : 287
SasaCANXa  : K....R..Y...........VV.......V....T........T.....I..AA.....D.H.. : 304
OnmyCANXa  : K..S.R..Y...........VV.......V....T........T.....I..AA.....D.H.. : 304
SasaCANXb  : K....R..Y...........VV.......V....A........T.....I..AA.....D.H.. : 302
OnmyCANXb  : K..S.R..Y...........VV.......V....A........T.....I..AA.....D.H.. : 302
OrlaCANX   : K....RS..........FS.VV.......V....T........K........PA.......H.. : 304
DareCANX   : K..S.RS.Y...........V.....T....I..T.....S....V......PA.....D.H.. : 292
IcpuCANX   : KA.S.R..Y...........V..........I..T.............A...PA.....D.H.. : 297
LeocCANX   : K....R..Y...........V..........I..T..........VS.A...PA.....D.... : 285
SasaCLGN   : .A.V...KFY..........V......Y.......S.SR.S..H.VV.....TK..D..T.S.. : 301
HosaCLGN   : P..V...KF...R.......VM...DT..V....T...K.S..E.VV..IK.PK.....N.K.. : 275

                   *        340         *       360         *       380
HosaCANX   : EDWDERPKIPDPEAVKPDDWDEDAPAKIPDEEATKPEGWLDDEPEYVPDPDAEKPEDWDEDMDG : 348
GagaCANX   : ............D...............A..N.V.............A................ : 351
SasaCANXa  : .........Q..D....E.......KQ....D.V..D.....Q...TS....V........... : 368
OnmyCANXa  : D........Q..D....E.......KQ....D.V..D.....Q...TS....V........... : 368
SasaCANXb  : .........Q..D....E.......KQ....D.V..D......S..TS....V........... : 366
OnmyCANXb  : .........Q..D....E.......KQ....D.V..D......S..TS....V..G........ : 366
OrlaCANX   : .........Q..D....E........Q...DN.V..D.........IG....V..D........ : 368
DareCANX   : .........Q..D....E..........A..D.V..D.........IS....V........... : 356
IcpuCANX   : D...........D...............A..D.V..D.........IS....V........... : 361
LeocCANX   : ............D....E.............................S....I........... : 349
SasaCLGN   : ......A...........N...E.....Q.PD.V...........F.A..A.N..D....E... : 365
HosaCLGN   : .E....A.....S....E....SE..Q.E.SSVV..A.......KFI...N....D..N..T.. : 339

                 *        400         *       420         *       440
HosaCANX   : EWEAPQIANPRCESAPGCGVWQRPVIDNPNYKGKWKPPMIDNPSYQGIWKPRKIPNPDFFEDLE : 412
GagaCANX   : ..........K........T....M..................VN................... : 415
SasaCANXa  : .......P.AL..T.....A....M...........A......N...V......A........H : 432
OnmyCANXa  : .......P.AL..T.....A....M...........A......N...V......A........H : 432
SasaCANXb  : ......VP.AL..T.....A....M....S......A......N...V......A..A.....H : 430
OnmyCANXb  : ......VP.AL..T.....A....M....S......A......N...V......A..A.....H : 430
OrlaCANX   : .......P..A........E.K..M.....H.....A......N.............A...... : 432
DareCANX   : .......P..V..T.....A.E..M...........S......N...V...............H : 420
IcpuCANX   : .......P.SA........K.E..M..............V...N...V..........Y....H : 425
LeocCANX   : .......P..K..T......................A......N...V...............H : 413
SasaCLGN   : ......VP..V..T.....Q.KP.T.N..Q......A.LV...H...V.S....Q...Y....A : 429
HosaCLGN   : .......L..A.RI--...E.KP.M....K...V.R..LV...N.....S........Y...DH : 401
              *        460         *       480         *       500         *
HosaCANX   : PFRMTPFSAIGLELWSMTSDIFFDNFIICADRRIVDDWANDGWGLKKAADGAAEPGVVGQMIEA : 476
GagaCANX   : ..K......V...................TE.AVA..........................MA. : 479
SasaCANXa  : .......N.V.......S........F.TNE.HTAER............E......L.N..MT. : 496
OnmyCANXa  : .......N.V.......S........F.TNE.HTAER............E......L.N..MT. : 496
SasaCANXb  : .......N.V.......S........F.TNE.HTA.R............E..L...L.N..MT. : 494
OnmyCANXb  : .......N.V.......S........F.TNE.HTA.R............E......L.N..MT. : 494
OrlaCANX   : .....A...V................F.TN..NTA.R..A.........E......LAA..LT. : 496
DareCANX   : .........V.......S........F.TS..NVAER............E......L.N...T. : 484
IcpuCANX   : .....AV..V.......S........F.TS..NVA.K..EE........E......L.N..MS. : 489
LeocCANX   : .........V.......S........M.SS..AVA.Q............E......L....MS. : 477
SasaCLGN   : ..K..S.R.V.........E.Y......TSYKEVA.R..G.S.....LVAS.N....FS.LTM. : 493
HosaCLGN   : ..LL.S...L...........Y.......SEKEVA.H..A...RW.IMIAN.NK...LK.LMA. : 465

                    520         *       540         *       560         *
HosaCANX   : AEERPWLWVVYILTVALPVFLVILFCCSGKKQT---SGMEYKKTDAPQPDVKE--EE--EEKEE : 533
GagaCANX   : .............I..F.....V.........P---.AA............VS--D.--R.E.- : 535
SasaCANXa  : .D.........V....V.LI.IVV.F.T...AAAP-AAAD.....E.......--..--.KA.- : 554
OnmyCANXa  : .D.........V....V.LI.I.V.F.T...AVAP-AAAD.....E.......--..-E.KA.- : 555
SasaCANXb  : .D.........V....V.LV.I.V...T...T----AAAD.....E.......--..VV.KA.- : 551
OnmyCANXb  : .D.........V....V.LV.I.V...T...TAA--AAAD.....E.......--..VV.KA.- : 553
OrlaCANX   : ........I..V...G..LV.LFV.......S.A--.PAD.....E.......--..--..E.- : 553
DareCANX   : ........I..V......LV.I.V...T...SSASTPAAK.....E.......--.A--..E.- : 543
IcpuCANX   : ...........V.......V.IFV...T...KPAT-.AA......E.......--..--..E.- : 547
LeocCANX   : .........I............VV.......KP---.AA......E......D--.Q--..E.- : 533
SasaCLGN   : ...............G..IG.TV...WPK.----SDDDYV...V.P.K...E.--..--.DDD- : 548
HosaCLGN   : ..GH....LI.LV.AGV.IA.ITS..WP-R.VKKKHKDT......ICI.QT.GVL.Q--....- : 525
```

```
                      580         *       600         *       620         *       640
HosaCANX    : EKDKG------DEEEE------------GEEKLE-EKQKSDAEEDGGTVSQ------------- : 565
GagaCANX    : -....------.K...E---------EAN....-....N..--.I.SA..----------EE-E : 570
SasaCANXa   : -E.Q-------VK..KS-------------QPAAA..-.....DSPAEKEE-------EEEEEE : 589
OnmyCANXa   : -E.Q-------VK..KS-------------QPEAAG.-.....DSPAEKE-------------- : 583
SasaCANXb   : -A.Q-------VK..KS-------------QPAA-..-N....DSPAEKEE-------VNEEEE : 585
OnmyCANXb   : -A.Q-------VK..KS-------------QPAAE..-N....DSPA--EE-------VNEEEE : 586
OrlaCANX    : -----------E....K-------------SPAA-..-N---------------------DE-D : 569
DareCANX    : -----------AKD.---------------.PEA--.-..EE.DST.DGDG---------DDTA : 569
IcpuCANX    : -----------E...ARG------------.ES--..-.A....SPAEAEA---------PE-E : 575
LeocCANX    : -.ED.EEEEKE.G...E---------KED...P.-..-...G..GA.EH..---------EE-E : 575
SasaCLGN    : -EEEEE-----E....KGDQAAEAKATESLTPA.-A.-.EK...ET.AAGGDSVEDEEEDEEEE : 604
HosaCLGN    : -EKAALEKPMDL...KK---QNDGEMLEK..ES.P.E-..EE.IE------------------I : 566

                     *       660         *       680         *
HosaCANX    : ----EEED-------------RKPK---AEEDEI--------LNRSPRNR-KPRRE : 592
GagaCANX    : EEEE....-------------..HA---S..E.T--------V.......-...KD : 601
SasaCANXa   : EEEE...EEEEEAAVNEEEEAATDQ---VRDKTT--------TR------------ : 622
OnmyCANXa   : -EEE...EEEEEAAVNEETEAATDQ---KQ..DV--------.R.....T-.G.KD : 626
SasaCANXb   : DEEE..DEVTEE---------VREQ------------------------------- : 601
OnmyCANXb   : -EEE...EVTEE-----NFLKSRES---QKPLDV--------CPM----------- : 614
OrlaCANX    : DEEE.QNE-------------AADE---QKLEDE--------.R..R...--T.KD : 599
DareCANX    : ENGDKDDN-------------TSNE---KS..D.--------.R......-.S.KD : 600
IcpuCANX    : EEADKQ..-------------KSDE---KQ..DV--------.R....SKARA.KD : 607
LeocCANX    : EEGE.RDADKTA-------KVAEEPLKNEKKEDV--------.RQ.....-...KD : 615
SasaCLGN    : DEGE...ETKSN-------EAASDDQ--K..ADEGGHSVGDGHKQ.V.K.-RV.KD : 650
HosaCLGN    : IEGQ..SNQSNK-------SGSEDE---MK.ADESTGS-GDGPIK.V.K.-RV.KD : 610
```

Figure S6c. Alignment of deduced CALR and CALRL amino acid sequences

Sequence references are shown in Figure S6a. Numbering below the alignment relates to the mature human CALR sequence. For catfish CALR sequences, see (4). Residues are color coded according to physiochemical properties. Cyan shaded residues are involved in glycan binding (5) and acidic region and ER-retention signals are shaded yellow (6).

```
SasaCALR1a   : ----MLVSVLL--MIALASAKPSVYFREQFED-DAWNTRWVESSHRSDYGKFVL :  47
SasaCALR1b   : ----.RG.M.FSAL......E..L..K.....G...T................. :  50
SasaCALR1.2  : --MTTMLIL.MTVLV.SIFGES......E...G...KS.....K.........H :  52
SasaCALRL1   : --MQV.G.FAIILS--IF.VHST...Q...L.G...K...LD.K.KA...EWK. :  50
SasaCALRL2a  : --MRVA.AI.AVFASVAVTIDAT...K...Q.G...KS..LV.E.K....EWK. :  52
SasaCALRL2b  : --MRVA.AIFSVFASVAVTIDAT...K...Q.G...KS..LV.K.KT...EWK. :  52
OrlaCALRL1   : --MKFCSAL.ALFA--GVAVEAT.....E.T.G.G.KS..L..K.K....QWK. :  50
OrlaCALR3    : --MNT.SLFVGLIA--.YCVHAKI....E.L.G.E.RS...N.K.K....EWK. :  50
OrlaCALRL2   : ----.GG------LLDIDR.S--------------------------------- :  11
DareCALR     : --MRITAA.CFISA-LAFI.HAD...K...L.G.G.KS.....K.K....QWK. :  51
DareCALRL    : MQISI.QLISAALL--ALA.NAE..LN...L.G...RS...N.E.K....Q.K. :  52
DareCALRL2   : --MTA.SLLFMAVSV..IT.ESN.........G.S.RS.....K.KT....... :  52
IcpuCALR     : --MKISLALFAVFA-VGITVDAT.F.K...L.GEGFKS..L..K.K....QWK. :  51
IcpuCALRL    : -MQSFK.FTIFSAALLAF.VDAT...K...L.G...TS...N.E.K....Q.K. :  53
IcpuCALRL2   : --MTA.CLLFISLSF..IT.ES..........G...KS..L..T.K........ :  52
HosaCALR3    : ---MARAL.Q.WAICM.RV.LAT...Q.E.L.GEH.RN..LQ.TND.RF.H.R. :  51
HosaCALR     : ---ML.SVP..LGLLG..V.E.A...K...L.G.G.TS..I..K.K..F..... :  51
                 -17     *         1        *         20         *
```

```
                                                              C105
SasaCALR1a  : TAGKFYGDAEKDKGLQTSQDAHFYSSSARFEPFSNQGKTLVIQFTVKHEQNIDC : 101
SasaCALR1b  : .P......P............R................................ : 104
SasaCALR1.2 : ........V.RS.........R...A.S...SI..KDQ................ : 106
SasaCALRL1  : ...N.................R..AA.........E..............K... : 104
SasaCALRL2a : ..........A..........R..AL.S.......E..S..V........K... : 106
SasaCALRL2b : ..........A..........R..AM.S.......E..P..V........K... : 106
OrlaCALRL1  : SS........A...V......R..AL....P....E..............K... : 104
OrlaCALR3   : ...N.................R..AA.........E..S...........K... : 104
OrlaCALRL2  : --......G...............A.....DD...K.QP...........S... :  63
DareCALR    : .S......P.L..........R..AL.S..DS..................K... : 105
DareCALRL   : ...N.................R..AT.V.......E..............K... : 106
DareCALRL2  : S.......................AM....GD.N.KDQP.....S......... : 106
IcpuCALR    : S.........L..........R..AL.S.......E..P...........K... : 105
IcpuCALRL   : ...D.................R..AT.........E..S..V........K... : 107
IcpuCALRL2  : S.......................AI.S...D...KDQP...........G... : 106
HosaCALR3   : SS.....HK........T.NGR..AI....K....K.......Y......KM.. : 105
HosaCALR    : SS......E............R..AL..S......K.Q...V............ : 105
                 40         *        60         *        80

                   K111                  C137
SasaCALR1a  : GGGYIKLFPADLDQADMHGDSNYNIMFGPDICGPATKKIHVIINYKGKNHLIRK : 155
SasaCALR1b  : .....................K............G...V.............S. : 158
SasaCALR1.2 : ............N.EE.....T............G...V...F.........N. : 160
SasaCALRL1  : ....V.V..ST..........Q.Y.........YS...V...F.........K. : 158
SasaCALRL2a : ....V.I........A.....Q.Y.........YS...V...F.........K. : 160
SasaCALRL2b : ....V.I...N....A.....Q.Y.........YS...V...F.........K. : 160
OrlaCALRL1  : ....V.V..S....S......K.YV........YS...V...F....Q....K. : 158
OrlaCALR3   : ....V.V..S....T......S.YV........YT...V...F.........K. : 158
OrlaCALRL2  : .........SE.N.E......V............G...V...F.........N. : 117
DareCALR    : ....V.V...EM..TE...E.Q.Y.........YS...V...F....Q....K. : 159
DareCALRL   : ....V.V.....N........Q.Y.........YS...V...F....Q....K. : 160
DareCALRL2  : .........S..N.E......T............G...V...F.........N. : 160
IcpuCALR    : ....V.I............E.Q.YL........YS...V...F....Q....K. : 159
IcpuCALRL   : ....V.V.....N...L....Q.Y.........YS...V...F.........K. : 161
IcpuCALRL2  : .........S..K.EE.N...T............G...V...F.........N. : 160
HosaCALR3   : ......V....I..KNLN.K.Q.Y.........FDI..V...LHF.N.Y.ENK. : 159
HosaCALR    : ....V....NS...T......E............G...V...F......V..N. : 159
                *         100       *         120       *         140

SasaCALR1a  : DIRCKDDEYTHLYTLILNPDNTYEVKIDNKKVESGSLEEDWDILPPKKVKDPEA : 209
SasaCALR1b  : .V.................................................... : 212
SasaCALR1.2 : ................V.........................F..L..I...D. : 214
SasaCALRL1  : EVK.....L..........NQ........E.....T......F..A.TI..... : 212
SasaCALRL2a : E.K.....L...........Q......N.E.....T..D........T...... : 214
SasaCALRL2b : E.K.....L...........Q......N.E.....T..D......A.TI..... : 214
OrlaCALRL1  : ..K.....L...........Q....R...E........D...M.....I...D. : 212
OrlaCALR3   : E.K.....F...........Q..Q.....E............F.....I..... : 212
OrlaCALRL2  : ........F......VV..................N......F.....I...D. : 171
DareCALR    : ..K.....L........R..Q........E............F.....I..... : 213
DareCALRL   : ..K.....L...........Q........E............F.....I..... : 214
DareCALRL2  : .........S......V.........................F..S..I..... : 214
IcpuCALR    : ........F.N......R..Q......N.E............F.....I..... : 213
IcpuCALRL   : ..K.....L...........Q........E............F.....I..... : 215
IcpuCALRL2  : .........S.....VV.....................D...F.....I...D. : 214
HosaCALR3   : L....V.GF........R..LS.D....GQSI....I.Y..NLTSL..ETS.AE : 213
HosaCALR    : ........F.......VR...........SQ.......D...F.....I...D. : 213
                   *         160       *         180       *
```

```
SasaCALR1a  : VKPDHWDERERMEDPDDKKPEDWDRPENIADPDAKQPEDWDDEMDGEWEPPMVS : 263
SasaCALR1b  : ....D......V.......................K.....N............ : 266
SasaCALR1.2 : E...D...K.NID..E........VA...P.....K.D...............T : 268
SasaCALRL1  : K...D..D.PK.D.AE.A......VA...P.....K.D...ED........VIT : 266
SasaCALRL2a : K..ED..D.AKID..T.T.....EK....P.....I.D...VD.........IP : 268
SasaCALRL2b : K..ED..D.PKID..T.T...G.EK....P.....K.D...VD........VIP : 268
OrlaCALRL1  : K..SD..D.ATID..S.T......Q..T.P.....K.D...ED.........IT : 266
OrlaCALR3   : K..ED..D.PKID..E.T.....EK..Y.P.....K....QED.........I. : 266
OrlaCALRL2  : K..ED..D..KIP...........K....P.....K................IA : 225
DareCALR    : K...D..D.AKID..E.T......K....P.....K.D...ED.......A.IP : 267
DareCALRL   : K..ED..D.AKID.ET.T......K....P.....K.....ED.......A.IP : 268
DareCALRL2  : K..ED.....KID..E.Q...E..K....P.....K.D...E...........T : 268
IcpuCALR    : K..ED..D.AKID..E.T......K....P.....K.D...ED.........IP : 267
IcpuCALRL   : K..ED..D.AKID.ET.T......K....P.....K.....ED.........IP : 269
IcpuCALRL2  : K..ED.....KIG...........K..T.P.....K.D...E...........T : 268
HosaCALR3   : S.--D.EQTK------.N.AQ..EK--HFL.ASTSKQS..NGDL..D.PA..LQ : 257
HosaCALR    : S..ED....AKID..T.S......K..H.P.....K.....E.........VIQ : 267
               200       *         220       *         240       *

SasaCALR1a  : NPDYKGEWKPRTIDNPDYKGKWLHPEIDNPDYSADSEIYRFDSIGVIGLDLWQV : 317
SasaCALR1b  : ...........K..........V.......E....................... : 320
SasaCALR1.2 : ..........KQ....A.....V.......E.T..P...QYA............ : 322
SasaCALRL1  : ..E.......KQ....N...A.I.......E.A...T..K..D.S.L....... : 320
SasaCALRL2a : ..E.Q....AKQ....E...A.V.......E.T..AS..K..N...L....... : 322
SasaCALRL2b : ..E.Q.....KQ........T.V.......E.T..TS..K..N...L....... : 322
OrlaCALRL1  : ..EF......KQMS..N...A.V...V...E.AP.ANM.L..N...L....... : 320
OrlaCALR3   : ..E.......KQ........A.V.......E..P.AN..K......L....... : 320
OrlaCALRL2  : ......D.NA.E....A.....I.......E.T..P...KY............. : 279
DareCALR    : ..E.......KQ....S...T.V.......E.A..DA..K......L....... : 321
DareCALRL   : ..E.......KQ....N...A.V.......EHVP.DQ..K..N...L....... : 322
DareCALRL2  : ...........Q....A.....V.......E.TP.....KY............. : 322
IcpuCALR    : ..E.......KQ....N...T.E.........TP.TS..KY.N...L....... : 321
IcpuCALRL   : ..E.......KQ....N...I.E.......E..P.....K..N........... : 323
IcpuCALRL2  : ..........KQ....A.....V.......E.TP.....KYS............ : 322
HosaCALR3   : K.P.QDGL..EG.H----.DV...RKMK.T..LTQYDLSE.EN..A...E.... : 307
HosaCALR    : ..E........Q........T.I.......E..P.PS..AY.NF..L....... : 321
                     260       *         280       *         300

                    D328
SasaCALR1a  : KSGTIFDNFLITDDATLAEEVGNETWGQTKDPEKKMKESQEEKERKKLEAEEMA : 371
SasaCALR1b  : .....................................V....Q..........G : 374
SasaCALR1.2 : ............N.PK...........A.........DRL..E....R....VN : 376
SasaCALRL1  : ..........VS..VKE..KF.A....V..E......--...DDK.RK.ED.KN : 372
SasaCALRL2a : ...........G..IKE...F......A.........DA...E...AR.E..KS : 376
SasaCALRL2b : ...........G..VKE...F......T..E......DA...E...AR.E..KS : 376
OrlaCALRL1  : ..............IKE....AK...EV..G.....RDD..DI...LR.E..KS : 374
OrlaCALR3   : ....V.........VKE..D..K....A..E..Q...--..QD.L.RKDE..KN : 372
OrlaCALRL2  : ............N.PN.......D...K...A.R......D.E....R.E.DKK : 333
DareCALR    : ..............VEE..KF.TD..VRQR-TREE..DQ...E....R.E..KS : 374
DareCALRL   : .........F.S..VKE...F......A..G......--E.QE.K.RK.E..KN : 374
DareCALRL2  : ..S.........N.PK.......T...A...A..........ED...HDE..KS : 376
IcpuCALR    : ..............VKE..DF.K....A..G......DK...E...QR.E..KN : 375
IcpuCALRL   : ...........A...KE...F.KN...V..G......DE.--D.K.RK.ED.KN : 375
IcpuCALRL2  : ............N.PK........S..A...A.....D....E....R.E.DKK : 376
HosaCALR3   : R.............EEY.DNF.KA...E..G..RE.DAI.AKE.M..ARE.--- : 358
HosaCALR    : ............N.EAY...F......V..AA..Q..DK.D.EQ.L.E.E.DKK : 375
                 *         320       *         340       *
```

```
                        Acidic region                       ER retention signal
SasaCALR1a  : RKEETKDEPGEEEEEEEEEELEHEEEEDEEEGETGAQEEEEESDSIKDEL : 421
SasaCALR1b  : ...Q.....E........K.E.L.DR.E....-...E.....T....... : 423
SasaCALR1.2 : K...D--.DED.DK..RD..EDYDD..E..---------...TDSKL.... : 416
SasaCALRL1  : KEQA.EA.EEG....G.D.GE.E.TP.----------EGT...EAPG.... : 413
SasaCALRL2a : K-DTAD..GD.D.--D.P..EDD-DSPT.--------E..G.DPKKD.... : 415
SasaCALRL2b : K.DTAD..GD.D...D.SK.E.E-DSPT.--------EG...IPMKD.... : 418
OrlaCALRL1  : K.DDEDEMDE.DD...KN..E.EDMDAG----------TDDD.DIKQ.... : 415
OrlaCALR3   : KDQD.DGDDEDID.D-...SK.EA..A----------LS.TD.DAQA.... : 412
OrlaCALRL2  : ..D.DEE.DK........D.E.EDG..P.L.E-----..D.SP.AV.... : 378
DareCALR    : K.D-DNE.DE.D.D.D.P..DD.T..PP.------EE..G.DDALP.... : 417
DareCALRL   : KEQN.EAADE...D.G....E.D.T..PQ-------ED.GD.DVLQ.... : 417
DareCALRL2  : ....A.-.EE...K...D..E.E....E..DE------...TDSKL.... : 419
IcpuCALR    : K..SEG..GDDD.P.D.Q..D.E..TPE.PNL--LDD..D..DKPS.... : 423
IcpuCALRL   : KEQD.TTDDDDDSDNDDDDDNDE..GKE.TTE.PDV-.G..DDAQP.... : 424
IcpuCALRL2  : .S..A...AD..DKD..D.DE.D.......-----DG...GTDSKL.... : 421
HosaCALR3   : -------.EE.LLSGKINRHEHYFNQFHR-----------------RN.. : 384
HosaCALR    : ....EEA.DK.DD.DKD.D.EDE.DK.ED.---------..DVPGQA.... : 417
             360       *         380               *
```

References:

1. Le SQ & Gascuel O (2008) An improved general amino acid replacement matrix. *Molecular biology and evolution* 25(7):1307-1320.
2. Kumar S, Stecher G, & Tamura K (2016) MEGA7: Molecular Evolutionary Genetics Analysis Version 7.0 for Bigger Datasets. *Molecular biology and evolution* 33(7):1870-1874.
3. Fuller JR*, et al.* (2004) Characterization of the molecular chaperone calnexin in the channel catfish, Ictalurus punctatus, and its association with MHC class II molecules. *Developmental and comparative immunology* 28(6):603-617.
4. Liu H*, et al.* (2011) Molecular responses of calreticulin genes to iron overload and bacterial challenge in channel catfish (*Ictalurus punctatus*). *Developmental and comparative immunology* 35(3):267-272.
5. Kozlov G*, et al.* (2010) Structural basis of carbohydrate recognition by calreticulin. *The Journal of biological chemistry* 285(49):38612-38620.
6. Raghavan M, Wijeyesakere SJ, Peters LR, & Del Cid N (2013) Calreticulin in the immune system: ins and outs. *Trends in immunology* 34(1):13-21.

Additional file 10. Figure S7. Beta2-microglobulin (b2m) data

Table of Contents		Page
S7a	Phylogeny of deduced b2m sequences from selected species.	1
S7b	Alignment of deduced b2m amino acid sequences	2

Figure S7a. Phylogeny of deduced b2m sequences from selected species.

The evolutionary history was inferred by using the Maximum Likelihood method based on the JTT matrix-based model (1). The tree is drawn to scale, with branch lengths measured in the number of substitutions per site. All positions with less than 95% site coverage were eliminated. Evolutionary analyses were conducted in MEGA7 (2). Bootstrap values from 100 trials are shown next to the branches. Sequence references are shown in parenthesis. Salmon b2m1 and b2m11 represent the two sequence groups found in the 13 Atlantic salmon gene sequences (Additional file 2: Text S1). Unique duplications in the Ostariophysi and neoteleost lineages are colour shaded while Atlantic salmon sequences are shown in red font.

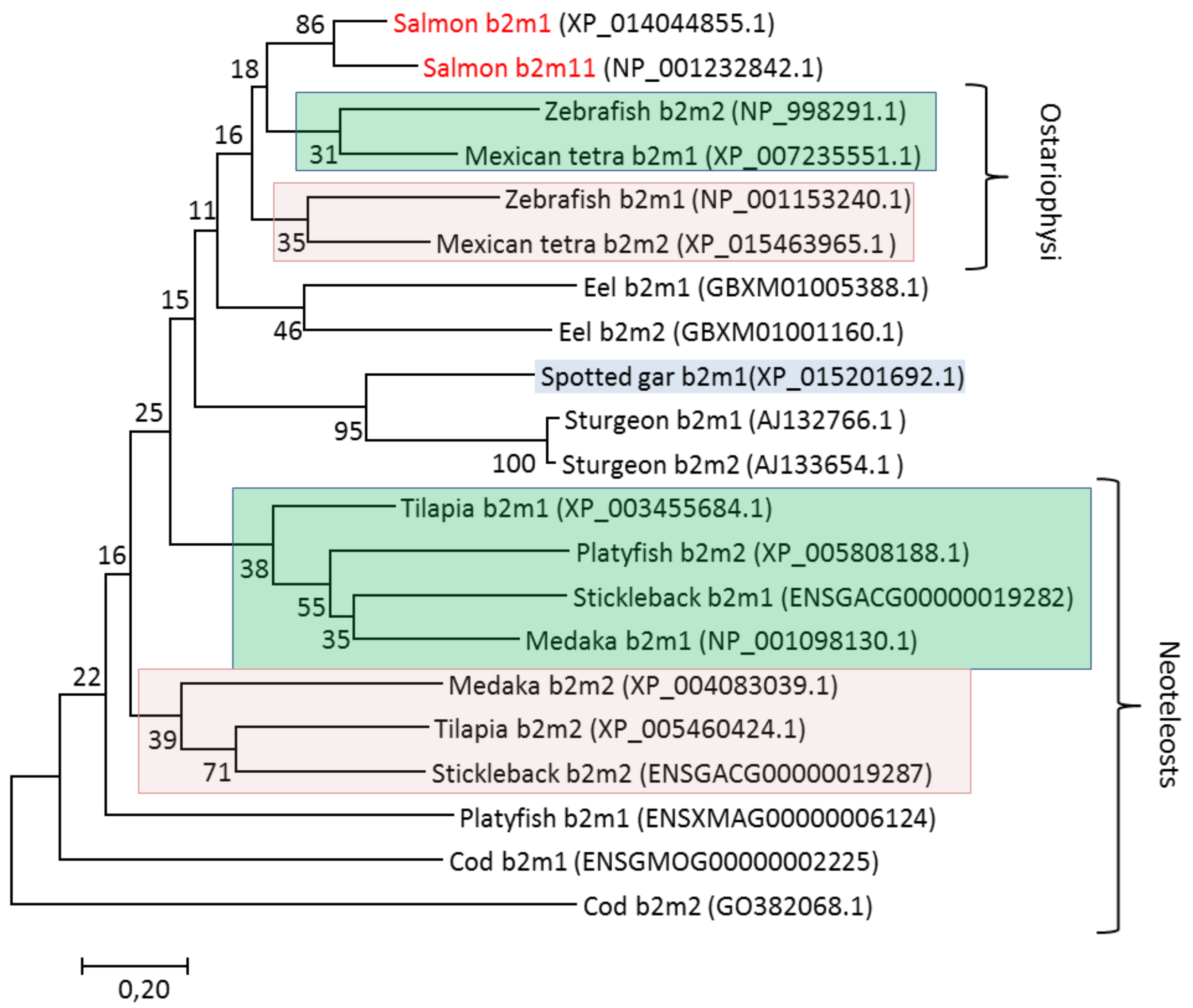

Figure S7b. Alignment of deduced b2m amino acid sequences

Sequence references are shown in Figure S7a. Abbreviations are as follows: Sasa is Atlantic salmon, Orni is Nile tilapia (*Oreochromis niloticus*), Gaac is stickleback (*Gasterosteus aculatus*), Xima is platyfish (*Xiphopherus maculatus*, Orla is medaka (*Oryzias latipes*), Gamo is cod (*Gadus morhua*), Dare is zebrafish (*Danio rerio*), Asme is Mexican tetra (*Astayanax mexicanus*), Anan is eel (*Anguilla anguilla*), Acba is sturgeon (*Acipencer baeri*), Leoc is spotted gar (*Lepisosteus oculatus*), Gaga is chicken (*Gallus gallus*), Hosa is human (*Homo sapiens*). Trout b2m sequences were described in Shum et al. (3) while Kondo et al. (4) describe two distinct b2m variant sequences in other teleosts. Sequences are color coded according to physiochemical potential.

```
                       *        20         *        40         *        60
Sasab2m1 : --MKSI---LSIVVLVLIYSA--VESKESPPKVQVYSRNPGNFGDKNTLICHVSGFHPPDISIQ :  57
Sasab2m11: --..TV---..VIAFCVFLGF--INA...............EH.KD.................. :  57
Ornib2m1 : --..AV---.CLA.IAAF.C.--..A.Y....T........EY.KE.V......N......T.T :  57
Ornib2m2 : --..TF---VFLA.VL.LGLTDLSAA.N.S......T.S..QY.TA.............E.T.E :  59
Gaacb2m1 : --.MLF---FTLAA.LA.SF.--QDPNHT........H...E.DQE...............T.. :  57
Gaacb2m2 : --..IL---VCAF.VG.LSL.S-SQD.T.S...E...KL..EY.KV.......TD....E.K.E :  58
Ximab2m1 : --.SQT---.AV...LPM.QSN-TFQIQFS....I......LY.N..V...........E.K.D :  58
Ximab2m2 : --..LL---.CLAA..A.FC.--.SC.I.....LI......E..KQ.S......N......T.D :  57
Orlab2m1 : --..EL---FF.AA.AAFCC.--AF..T...T......D..QY.KE........N......T.R :  57
Orlab2m2 : --..G.VVTVVLL.C.DLGVSI-K.RN.QA........L..TL.KE......T......E...K :  61
Gamob2m1 : --..IT---FCA.AVA.LLCT--......V.......SK.AEL.EA.....L.QA....E.T.E :  57
Gamob2m2 : --.GVFGVKMNPLMISVFCGLVLMSMAIT.......T.E.AEP.TG.S...YLNN.Q..EVKVD :  62
Dareb2m1 : --.RAL---ITFAL.C.L.IT--.QG.V.T...H...HF..EY.KP.....Y..S........E :  57
Dareb2m2 : --.WFK---.AV.A..FLNAS--CLA..T............EYDKD.....Y.........T.E :  57
AsmeB2m1 : --..LM---.FC..FAFLGVS--SFA...............E..KD.V.............T.D :  57
Asmeb2m2 : --..LL---..F..ACFL.IS--A.A...........YS..E..KP.H......E......N.E :  57
Ananb2m1 : --.N..---.TSFC.AVLLVG--.N.IH.A.......KL..Q.DQP.V.L.Y..N......K.T :  57
Ananb2m2 : --..LL---.CLA..AIALIS--.NGVH.A...........ML.VG.K.............K.. :  57
Acbab2m1 : MFLR.TF--VAAL.AC.A.IHL-GDAT.AM....I....V.EL.KP.............N.KL. :  61
Acbab2m2 : MFLR.TF--VAAL.AC.A.IHL-GDAT.AM....I....V.EL.KP..........Q..N.KL. :  61
Leocb2m1 : --..VLY--.TAL.AF.A.LDS-AAAAAAE....I......EYDKS........D......K.E :  59
Gagab2m  : --.GKA---AAV.LVT.VALLG-LAQADLT........F.ASA.T..V.N.FAA.....K...T :  58
Hosab2m  : --.SRS---VALA..A.LSLSG-L.AIQRT..I.....H.AEN.KS.F.N.Y......S..EVD :  58
                     *        80         *       100         *       120
Sasab2m1 : LLKNGVEIPDAKQTDLAFEQGWQFHLTKSVGFTPDSGEEYTCRVRHLK--NL-KTYTWEADM : 116
Sasab2m11: ................................................--..-......SN. : 116
Ornib2m1 : .........G......V.N.D.H.....H.A...KE..N.A.K.T.GQ--DT-..FG..SN. : 116
Ornib2m2 : ....DKV..GSQ.......EN.HY.M..H.P...TKEDVFS...T.TG--KK-R..I..... : 118
Gaacb2m1 : .M.D...L.N..L.....K...R.V...H.T...RR..K...K.T.GT--TV-RD.A..SN. : 116
Gaacb2m2 : ..R.RQA..G.M.......AS.HY....H.P...QP.DTFS.N.T.VG--IS-.I....P.. : 117
Ximab2m1 : .....Q..N.F........EN.HY....HAN...VE..R.M...T.VG--TT-TQHE....V : 117
Ximab2m2 : ..CD.TTL.NPV......K.D.H......AT...E..KTCI...N.AG--TS-ND.N..PN. : 116
Orlab2m1 : .MEDDQ.L.E........K.N.H......AP...KQ.AR...K.T.SG--LA-.D....SN. : 116
Orlab2m2 : .....N.L.GGG.......ES.HY....HAP...EK..S...V.T.TT--GT-......P.V : 120
Gamob2m1 : ...D.AVL.N.N....S.DKT.H...S.FADI..SKE.S.A.Q.T.IG--KR-TVHA..PN. : 116
Gamob2m2 : ..E..AV..G.V.S..M..SQ..Y....R.P.I.RE.AR.A...N.MG--RI-TDHG.DMND : 121
Dareb2m1 : .....QVMS.T........K..........A...EK.D....S...M.--ET-.KFS..PN. : 116
Dareb2m2 : .....QP.EKTT..........F.....FAA.K.RP..V...Q....T--ET-......P.. : 116
AsmeB2m1 : ..E..TP..N....................T...K..VM.S.K...MQ--TT-...N..S.E : 116
Asmeb2m2 : ..R...V.TE............K.......P...QKN...A...T.MQ--KAPQK.S.DP.. : 117
Ananb2m1 : ...DN..M.G.T........S.........P...QA..K.A...Q.QG--IT-.P.S..P.. : 116
Ananb2m2 : .ME..R...N.E........T......R..D...VE.K..S.V.K.QN-DAA-.S.Q..P.. : 117
Acbab2m1 : ....D...EG.Q.S....H...H.......A...KE..H.S.K.E.STLREA-TRF..TP.. : 122
Acbab2m2 : ....D...EG.Q.S....H...H.......A...KE..H.S.K.E.STLRGA-.SF..TP.. : 122
Leocb2m1 : ....KQQMEGV..S........H....QT.P...KK.DI.S...S.STLSVP-.EF...P.. : 120
Gagab2m  : .M.D..PMEG.QYS.MS.NDD.T.QRLVHAD...S..ST.A.K.E.ETLKEP-QV.K.DPEF : 119
Hosab2m  : .....ER.EKVEHS..S.SKD.S.Y.LYYTE...TEKD..A...N.VTLSQP-.IVK.DR.I : 119
```

References:

1. Jones DT, Taylor WR, & Thornton JM (1992) The rapid generation of mutations data matrixes from protein sequences. *Computer Applications in the Biosciences* 8:275-282.

2. Kumar S, Stecher G, & Tamura K (2016) MEGA7: Molecular Evolutionary Genetics Analysis Version 7.0 for Bigger Datasets. *Molecular biology and evolution* 33(7):1870-1874.
3. Shum BP*, et al.* (1996) Unexpected beta2-microglobulin sequence diversity in individual rainbow trout. *Proceedings of the National Academy of Sciences of the United States of America* 93(7):2779-2784.
4. Kondo H*, et al.* (2010) Identification of two distinct types of beta-2 microglobulin in marine fish, Pagrus major and Seriola quinqueradiata. *Veterinary immunology and immunopathology* 134(3-4):284-288.

Additional file 11: Figure S8. ERp57 and ERp57L data

Table of Contents		Page
S8a	Phylogeny of deduced ERp57 and ERp57L amino acid sequences	1
S8b	Alignment of deduced ERp57 and ERp57L amino acid sequences	2

Figure S8a. Phylogeny of deduced ERp57 and ERp57L amino acid sequences

The evolutionary history was inferred by using the Maximum Likelihood method based on the Le and Gascuel 2008 model (1). The tree is drawn to scale, with branch lengths measured in the number of substitutions per site. All positions with less than 95% site coverage were eliminated. Evolutionary analyses were conducted in MEGA7 (2). Bootstrap values resulting from 100 trials are shown next to the branches and accession numbers are shown in parenthesis. Salmon sequences are shown with red font. The tree was rooted using PDIA4 sequences. The Spotted gar ERp57L sequence was predicted using FGENESH (3) and can be found in Additional file 2: Text S1.

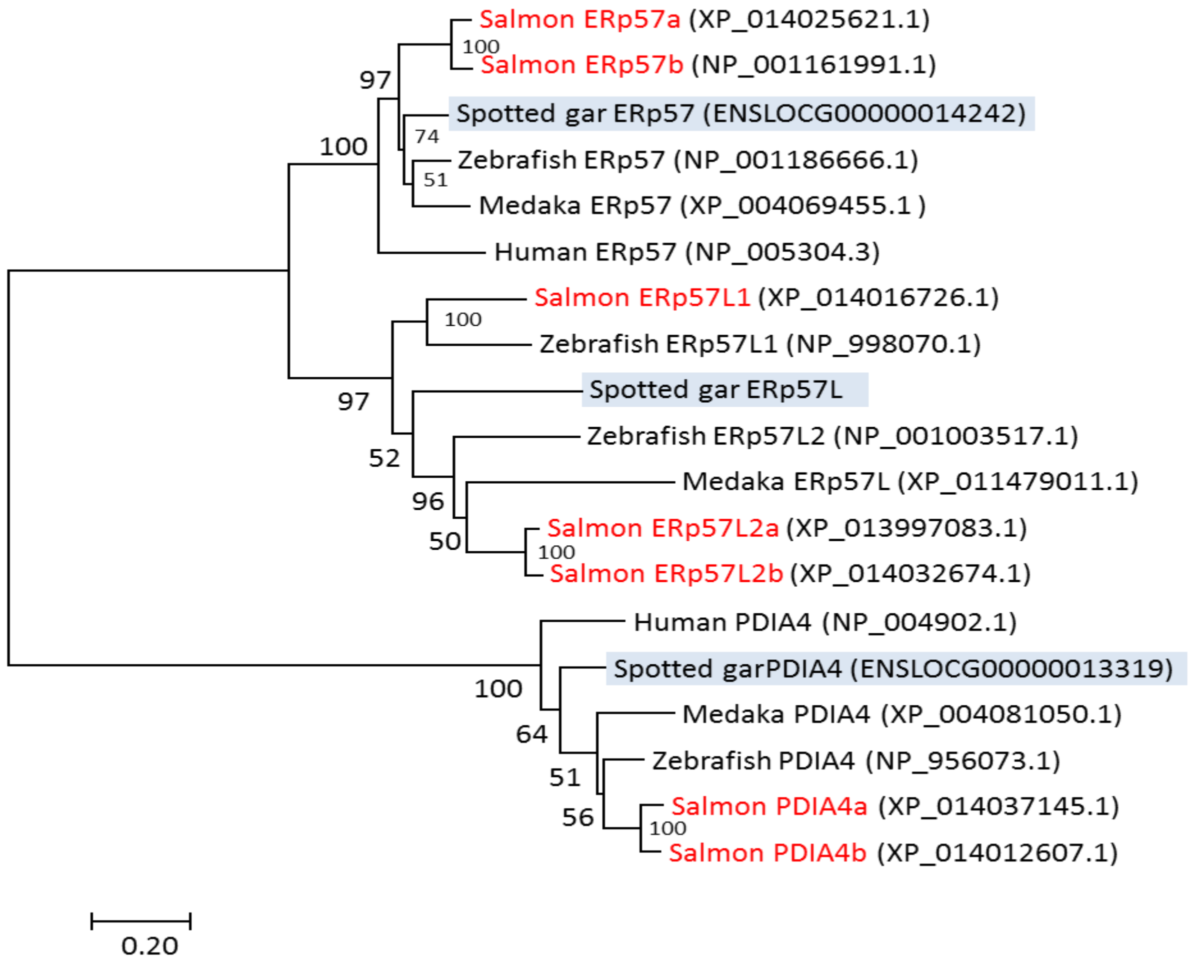

Figure S8b. Alignment of deduced ERp57 and ERp57L amino acid sequences

Sequence references are shown in Figure S8a. Residues are color coded according to physiochemical properties. Residues interacting with Calnexin and Calreticulin are shaded cyan while those interacting with Tapasin are shaded green (4). Numbering according to the mature human ERp57 sequence.

```
                     -24           *         1        *         20
HosaERp57    : --------MRLRRLALFPGVALLLAAARLAAASDVLELTDDNFESRISDTGSAGLMLVE :  51
SasaERp57a   : ----------MLK.FF.VV------L.GA.L....I.F...D.D.K.G.H.---MI... :  40
SasaERp57b   : ----------MLK.FF.IV------L.GA.R....I.FS..D.D.K.G.H.---MI... :  40
DareERp57    : ---------MMLA.LFLVA------F.AA.R......Y...D.D...G.HD---.I... :  41
LeocERp57    : MNGWQTVPCTSLV.Y.GVPRTSPCFL.VAVS......F...D.....A.HD---.V... :  56
SasaERp57L1  : -------MGT.GPFRM.LLF..AQQNVFV.-.......G.SD.HYTVAEYE---TV... :  48
DareERP57L1  : -------------MR.PCFIICAVLTVW..EG......G.SD.DRSAGMHD---TL... :  43
SasaERp57L2a : -------MASFLS.IPPFLLSV.IFSGAAV.RG.....G.AD.DYLAAEHE---T...K :  49
SasaERp57L2b : -------MASFLS.IPAFTLSVVIFCGAVV.RG.....G.AD.DYLAAEHE---T...K :  49
DareERp57L2  : --------.DEMI.RGLLCILVCSLSSSAREH....K...AD.DYLAPEHE---TL..K :  48
LeocERp57L   : -----MARLSV.L.CPAALL..AALG.VAV.GG.....G.AD.DGAVAQHE---TV... :  51

                    C57 forms disulfide bond with C95 in tapasin
               *         40        *         60        *         80
HosaERp57    : FFAPWCGHCKRLAPEYEAAATRLKGIVPLAKVDCTANTNTCNKYGVSGYPTLKIFRDGE : 110
SasaERp57a   : ..........K......V.........G.......VHN.V.Q................. :  99
SasaERp57b   : ...............F.V.........A.......VQN.V.Q.............K... :  99
DareERp57    : .....................................SKV.G................. : 100
LeocERp57    : .................S...................S.V...F............G.. : 115
SasaERp57L1  : .........QQ......T...K...T.S.......V.SE..GRF..N.........N.. : 107
DareERp57L1  : .........Q...........K...TLA.......V.SE..ERF..N.........N.. : 102
SasaERp57L2a : .Y........K...DF.T.......T...........PD..GRF..T.........N.. : 108
SasaERp57L2b : .Y........K...DF.T..S....T..........SPD..GRF..T.........N.. : 108
DareERp57L2  : .Y........K....F.S..S....T.T..........EI.KH...N.........N.Q : 107
LeocERp57L   : .........QK......E.......A..........HSE..SRF..N.........N.Q : 110

                *         100       *         120       *         140
HosaERp57    : EAGAYDGPRTADGIVSHLKKQAGPASVPLRTEEEFKKFISDKDASIVGFFDDSFSEAHS : 169
SasaERp57a   : D..P.......................E.K..AD.T.YVG.R...V....A.DG.P.KA : 158
SasaERp57b   : D.......................S.IE.K..AD.T.YVG.R...V....A.GG.P.QA : 158
DareERp57    : DS.G.......................E.KN.AD.E.Y.G.R...V....A.GG.A.QG : 159
LeocERp57    : .S.........................EIK.V...E...G.R...VI...AEGG.TTQA : 174
SasaERp57L1  : DFA......S......YM......S....HNGRDLDA.VNNF...V....SGVD.SQMA : 166
DareERp57L1  : .S..............YM......S..A.LK.ADLDG.VDNYE..V....SGED.AQLA : 161
SasaERp57L2a : D.SS.....S.....HFM......N..T..R.ADLEA.VNHF...V....SGPD.GQLA : 167
SasaERp57L2b : DSSS.....S.....HYM......N..T..S.ADVEA.VNHF...V....SGPDTAQLA : 167
DareERp57L2  : .SSS.....S.....DYM......D..L.HS.LDLE...NHF...V..L.SGTD.SQLA : 166
LeocERp57L   : .SS.............YM......S..E.....DLDS.V.HF.G.V....LGPE.ADLA : 169

                 *         160       *         180       *         200
HosaERp57    : EFLKAASNLRDNYRFAHTNVESLVNEYDDNGEGIILFRPSHLTNKFEDKTVAYTEQKMT : 228
SasaERp57a   : ....S..A..ESF......S.E.LQKHSVE..........R.N....EGS.KFS.DTF. : 217
SasaERp57b   : ....S..A..ESF......SGE.LQKNGVE.........AR.S....ESVIKFS.D.F. : 217
DareERp57    : .......A..ES.......N.D.LKKHGID........SPQ.S.....SS.LF..D.F. : 218
LeocERp57    : ....S..A..ES.......S.E.LQKHGID....V....PR.S....ESS.K.S.D.Y. : 233
SasaERp57L1  : .....S.AM..SH.....TDL..GLKHGVESDTVV....PR.NS....SL.KSD.-AVS : 224
DareERp57L1  : .....S.A...S.....STDVGAGLK.GVD..CVL....PR.SS....NV.K...-HLS : 219
SasaERp57L2a : .......VM.EHF.....IDMT.GLKHGVDT.RVL....PR.SS...ESVLHF..-TI. : 225
SasaERp57L2b : ......GAM..HF.....INMT.GLKHGVDT.SVL....PR.SG...ESVLRF..-TI. : 225
DareERp57L2  : ....G..LM.ESF.....TDLQ.GQK.GVTH.S.L...APR.SS...ESV.PH.G-SLS : 224
LeocERp57L   : ...R...SM..S......SA...RQRHGVQ..AVL...APQ.AS...ESV.RHRG-AVR : 227
```

```
                  *         220       *         240       *         260
HosaERp57     : SGKIKKFIQENIFGICPHMTEDNKDLIQGKDLLIAYYDVDYEKNAKGSNYWRNRVMMVA : 287
SasaERp57a    : NA...Q...D....M.....D....QMK.....V..........P...........K.. : 276
SasaERp57b    : NAM......D....M.....D....QMKD....V..........P...........K.. : 276
DareERp57     : .A.......D......A........QLK.....V..........P...........K.. : 277
LeocERp57     : .........D..L............QLK.....V..FE......P..T........K.. : 292
SasaERp57L1   : TASLRQ..RD.V..L...L.AE.RENMR.R...V.......LR.I..T........K.. : 283
DareERp57L1   : VSSLH..VKD..L.L.....ME.R.TVRES...T.FFN...LR.P..T......I.K.. : 278
SasaERp57L2a  : THTLRR..RD....M...L.NE.R.KLK.Q...T....L..LQ.P...........K.G : 284
SasaERp57L2b  : THTLRR..RD....M...L.NE.R.KLK.Q...T....L..LR.P...........K.G : 284
DareERp57L2   : VTGLRR..RD....L.....K...EVLRKR...T....L..LH.P..........LK.. : 283
LeocERp57L    : PDALRR..R.....M...L.HE.REQLMRQ...T.F.EL..LR.P.............. : 286
                    *        280        *         300       *         320
HosaERp57     : KKFLDAGHKLNFAVASRKTFSHELSD-FGLE-STAGEIPVVAIRTAKGEKFVMQEEFSR : 344
SasaERp57a    : .G...Q.N........KNS..QDIAE-M..D-ASS..L...G......D.Y..T..... : 333
SasaERp57b    : .S...Q.KT.......KNS...DI.E-M..D-ASS..L...G......D.Y..A..... : 333
DareERp57     : .G...Q.K..S....NKNR...DV.E-L..D-GSS..L.L.G......D.Y..K..... : 334
LeocERp57     : .S...Q.K..S....NKNS....I.E-...D-.SS..L...G......D.Y....... : 349
SasaERp57L1   : TQ.QSR.--.SY...N.AE.QE..EEE...GP.DG..L.LIT..NRE.H.YS.....T. : 340
DareERp57L1   : TQ.Q.R.--.T....D.QE.QD..EEE..VSS.EG.DV.L.T...RA.Q.YS.....T. : 335
SasaERp57L2a  : SQ.ASQ.--.S....N.RD.VD..EEE...GA.DG.DL.F.T...RQ.F.YT.R...T. : 341
SasaERp57L2b  : YQ.ASQ.--.S....N.RD.VD..EEE...GA.DG.DL.F.T...RQ.F.YT.R...T. : 341
DareERp57L2   : T..SSQ.--ML.S..N.ND.ME..EEE...SA.DGN.L.F.T...RT.D.YS.R...T. : 340
LeocERp57L    : SR.AGR.--.S....D.RD.PD..EE-....GAEG-.L..A.V..VL.Q.Y..R...T. : 341
                      *         340       *         360       *
HosaERp57     : DGKALERFLQDYFDGNLKRYLKSEPIPESNDGPVKVVVAENFDEIVNNENKDVLIEFYA : 403
SasaERp57a    : ...............K............N......T.......A...E.D......... : 392
SasaERp57b    : ...............K.........S..N......T.......A.....E......... : 392
DareERp57     : .........................V..N.......L......S...DDS......... : 393
LeocERp57     : ...............K.........V..........L.....E....DDS......... : 408
SasaERp57L1   : ...S.....E...A.K...QV...AAS.N..........D..E.....PS....V.... : 399
DareERp57L1   : ...S..K..E...AKR....V...............L..DT..A...DPE....V.... : 394
SasaERp57L2a  : ...S.....V...A.R....I.......K.K.........S.E....DPE......... : 400
SasaERp57L2b  : ...S.....E...A.R....I.......K.K.........S.E....DPE......... : 400
DareERp57L2   : ...S..S..E...A.R....V....V.AI.N.V......DT.E....DPE......... : 399
LeocERp57L    : .........E...A.R.........V..............T......DAS......... : 400
                 380       *         400       *         420       *
HosaERp57     : PWCGHCKNLEPKYKELGEKLSKDPNIVIAKMDATANDVPSPYEVRGFPTIYFSPANKKL : 462
SasaERp57a    : .......S....W........S..................Q.........F.A..GQ.M : 451
SasaERp57b    : .......S....W........S..................Q.........F.A..GQ.M : 451
DareERp57     : .......S.............E......................S..........GR.Q : 452
LeocERp57     : .......S.............D.................................G..Q : 467
SasaERp57L1   : .......S.....T....Q..A.TH..............PT.D.Q.....F.V..GQ.D : 458
DareERp57L1   : .....................GN................PN.D.Q.......V.SGQ.D : 453
SasaERp57L2a  : .......S........A.Q.YS.................QGFD.Q.......AQ.S..D : 459
SasaERp57L2b  : .......S........A.Q.YS.................QGFD.Q.......AR.D..D : 459
DareERp57L2   : .......K.....TA...M.YS............V....AG.D.Q.......AA.GR.S : 458
LeocERp57L    : ...........R.T...QQ..G.................EG.DIQ.......A.VGQ.A : 459

                                                     K/RXEL
                 440       *         460       *         480
HosaERp57     : NPKKYEGGRELSDFISYLQREATNPPVIQEEKPKKKKKAQEDL---------------- : 505
SasaERp57a    : S.........V.......KK.....L.A...ETS....-KNE.---------------- : 493
SasaERp57b    : S......A..V.......K......L.A...ETS..NI-.IE.---------------- : 493
DareERp57     : ..........V.......K.....TV.V..D.K.S...-KSE.---------------- : 494
LeocERp57     : S.........V.......KK..S..L.....EK.S...-KSE.SCLLERMKEQCLVDVN : 525
SasaERp57L1   : Q.R.......VN..LN..KE...H.L.LGTA--------R...---------------- : 493
DareERp57L1   : Q.RR......VN...T..KK.....LILDDS--------RDE.---------------- : 488
SasaERp57L2a  : Q..R...AH.VK...K..K...SHV..VSGV--------R...---------------- : 494
SasaERp57L2b  : Q..R...A..VK...K..K...SHI..VSGV--------R...---------------- : 494
DareERp57L2   : E..R...A..VK..VNF.K....K.LILNGV--------K.E.---------------- : 493
LeocERp57L    : A.RR..HAFHFHCISRW.KTRQVC.L--------------------------------- : 485
```

References:

1. Le SQ & Gascuel O (2008) An improved general amino acid replacement matrix. *Molecular biology and evolution* 25(7):1307-1320.
2. Kumar S, Stecher G, & Tamura K (2016) MEGA7: Molecular Evolutionary Genetics Analysis Version 7.0 for Bigger Datasets. *Molecular biology and evolution* 33(7):1870-1874.
3. Solovyev VV, Kosarev P, Seledsov I, & Vorobyev D (2006) Automatic annotation of eucaryotic genes, pseudogenes and promoters. *Genome Biology* 7(Supl.1):10.11-10.12.
4. Dong G, Wearsch PA, Peaper DR, Cresswell P, & Reinisch KM (2009) Insights into MHC class I peptide loading from the structure of the tapasin-ERp57 thiol oxidoreductase heterodimer. *Immunity* 30(1):21-32.

Additional file 12. Text S4. Alignment of deduced TAPBP, TAPBPR and TAPBPL amino acid sequences

Alignment of deduced Tapasin (TAPBP), Tapasin-related (TAPBPR) and Tapasin-like (TAPBPL) amino acid sequences from selected species. Numbering above and below the alignment relates to the human TAPBP and TAPBPR sequences. Sequence references are as follows: Atlantic salmon: TAPBPa_#C NP_001117077.1, TAPBPR NP_001133983.1, TAPBPL1a XP_014069540.1, TAPBPL1b XP_014017660.1, TAPBPL2 XP_014062182.1; Northern pike TAPBP XP_010899738.2; Zebrafish TAPBP GDQH01003123.1, TAPBPR XP_001919985.2, TAPBPL AAI71514.1; Medaka TAPBPR XP_011483883.1, TAPBPL XP_004075780.1; Spotted gar TAPBP GFIM01016833, TAPBPR XP_015193320.1, TAPBPL GFIM01040944.1; Frog (*Xenopus laevis*) TAPBPL XP_018100952.1; Turtle (*Chrysemys picta bellii*) TAPBPL XP_005298961.1; Chicken (*Gallus gallus*) TAPBP1 NP_001029988.2, TAPBPR NP_001026543.1, TAPBPL merged transcripts BU342879.1, BU369515.1 and BX257449.3; Kiwi (*Apteryx australis mantelli*) TAPBPL XP_013817376.1; Opossum (*Monodelphis domestica*) TAPBPL XP_007485846.1; Human (*Homo sapiens*) TAPBPR NP_060479.3, TAPBP NP_003181.3. The six conserved cysteines are shown above the alignment (C1-C6). Highlighted residues are as follows: TAPBP and TAPBPR TC2, TC3 and TN3-TN6 are regions that interfere with MHCI binding (1-3), sites interaction with ERp57 (1), N-linked glycosylation site interacting with CALR (4), transmembrane site associating with TAP (5), ER retention motif (6) and TAPBPR cysteine C94 interacting with UGT1 (7).

```
                                                                   TN4
                                                               C1
                                              1        *         20              *
Human TAPBPR     : ------MGTQEGWCLLLCLALSGAAETKPHPAEGQWRAVDVVLDCFLAKDGAHRG------ALASSEDRAR :  59
Chicken TAPBPR   : ------------------MLAVLAGTVEGLTPVPELRRVDVVLGCSYVWEGGLSR-AFG-------GSEHP :  45
Salmon TAPBPR    : -------------------MLEILLFGYLITCVSGQGGADVVLSCSLVEEGSGMG-GM---GGGALFSRTP :  48
Zebrafish TAPBPR : MQIVSDIQQNYSISCLVSSCSVMLTLLVSLSLVYGLDGADVVLSCSLIEEGGGMG-GMGGMMGGAMFKRTP :  70
Gar TAPBPR       : ----------------MRLAFGVLVLASHLMSSVSGSVADVVLACSLVEEGGGMG-GRLA-GAGALFTRDE :  53
Medaka TAPBPR    : -------------------MIEIIFFGLFITCVNADGAADVVLSCALVEEGSGLS-GM---EGGALFSRTP :  48
Salmon TAPBPL1a  : --------------MSLNVNILLCLFLCGEVPGIQSFEQVPWLPCQLVDESVTFN-DEG--HAETEYQHRD :  54
Salmon TAPBPL1b  : --------------MSLNVNIVICLFLCAEVPGIQSLEQVPWLPCMLVDERVKFN-DEG--HAETQYQHRN :  54
Zebrafish TAPBPL : ----MIVLRTRIHNMFWTIGGFILALVCLGVNGSQSLRDVQWLPCQFVDEKVQIN-EEG--HTETKYIHRE :  64
Medaka TAPBPL    : -----------------MGVFQKVVIYFYVIGVVRSVQQIPWLSCQFTDEHVSNN-SEG--HIETQLIPRE :  51
Salmon TAPBPL2   : ---------------MKTFTVLLCLIP---YAGVLGFLQVQWLRCRLKDEYVWTN-DEG--HIETNNSYRD :  50
Gar TAPBPL       : -------------MITTAVLLCVLICSGLRCVRSESLAEAGLLACELVEERVSSG-LSG--APETQFVNRK :  55
Frog TAPBPL      : ----------MDSRLYLIILNLLIVCNDIQSFEDLTSRKSRKLSCMYVDMRNKTL------LGYNDIMKQK :  55
Chicken TAPBPL   : ---------MAQRTLELPPCGHGSPQDGGAQCSNHKDPKQH-CFCIPRLRRILQLPCMFEMKKNIPLSSKT :  61
Kiwi TAPBPL      : ------MNYRTYLVAGCFLCIGIAKNLNGIATAGSSFRKSRQLACVFETSKIIPL------TKELEIIRLN :  59
Turtle TAPBPL    : ------MNFRIYTAVYCFLCTGILKHHNGVATAGSTFRQSRRLSCLFETTETIPL------LSEFTYIRQN :  59
Opossum TAPBPL   : ------MLGRGMLALRLLSPGLLWILGWHMVESGMTLGKAGQLPCLLEVALDPSE------TLNGERKWQH :  59
Salmon TAPBPa_27 : --------------MANISTIYKLSFLAFTYFIHVYGTSCPVLECWFVQEKPGRG-G----GFPAAMIQEK :  52
Pike TAPBP       : --------------MAXISTIYKLSIIAFIYFTNVYGTSCPVLECWFVQERPGRG-G----GFPAAMSQEK :  52
Zebrafish TAPBP  : --------------MSDISTVFKISVIAFTLFHGFYGSACPVLECWFVQEKPGHG-G----GLSTPMSQEK :  52
Gar TAPBP        : --------------MSDTATIYRLTLLALWGLAAVCGSRCPVLECWYVQEKAGQG-G----GFPAAMTQEK :  52
Chicken TAPBP    : -----------MAAGLRLLLAGLCWSQFRVEDAASPPPPPAPVRCALLEGVGRRG------GLPGGGNARP :  54
Human TAPBP      : ------------------MKSLSLLLAVALGLATAVSAGPAVIECWFVEDASGKG-----------LAKRP :  42
                                                        1         *                    20
                                                                   TN4
```

```
                                                                           C94        C2
                     40        *         60         *         80        *            100
Human TAPBPR      : ASLVLKQVPVLDDGSLEDFTDFQGGTLAQDD-PPIIFEASVDLVQIPQAEALLHADC--SGKEVTCEISRY : 127
Chicken TAPBPR    : ATLVLRGLSVTDDGTLGDVTDYEIPQADHSSSPPIIFEASEQLVSIPYAEALLHVDC--SGEEVSCELSPY : 114
Salmon TAPBPR     : AMLVLRDLAVTPDLSPDTLTPFNPPAVPDPD--NIILEAKVESPEIPEADLLLHADC--NEQEVTCEISRY : 115
Zebrafish TAPBPR  : ATLVFRDFVENEGLSPELVTPYNPPETPNPE--DLIFELMLPSIQIPNADLLLHADC--NEQEVVCEISRY : 137
Gar TAPBPR        : ATLVLRDLPVTADESLDTVTPFAPP-APDPE--NLIFEATVTSLEIPEADSLLHADC--NEQEVTCEISRY : 119
Medaka TAPBPR     : ATLVLRDVAVGPEEPLEMLSPFVPPSVPDPE--LFLFEASVTSPDIPNTDRLLHADC--NELEVLCEISRY : 115
Salmon TAPBPL1a   : AGLQFGHPGDS---------------ALNPN--TITFLVTG-----SKVDMRKYIEG-VVEHQLQCEIRRY : 102
Salmon TAPBPL1b   : AGLQFGHPGDS---------------ALNPN--AITFLVTG-----SKVDMRKYIEGAVVEHQLQCEIRRY : 103
Zebrafish TAPBPL  : SVLQFGKDGDS---------------ALHP---AITFLVTG-----SKVDMRRYLEG--SEDTLQCEIRRY : 110
Medaka TAPBPL     : AMVQFGLKGDA---------------PVNPH--AITFLITV-----SKLDLQRSIEG-VEVEELECEVRRY :  99
Salmon TAPBPL2    : AVLQFENSGNS---------------ALLSE--SITFLVAA-----SKVDMRKFVEG--PVDQLQCDIHRY :  97
Gar TAPBPL        : AALLLARPGREAD------TAQRLQEAQRAG--VVTFLATG-----SAVDVTRHVPG--GTEQLQCEIRRY : 111
Frog TAPBPL       : AFLVLSSRKQL------------SKEYFDSE--GITFILKD-----SPVNTMKFLKE--HTLDLVCEVNPY : 105
Chicken TAPBPL    : ETVLLNIRLLLPVAGAGGAQRLQTESLPAGN--LPSFIVQE-----PSVNILQHTDE--DINMLDCRISPY : 123
Kiwi TAPBPL       : VRLLLSGTESEEA------QRSHSENHPQDN--IPSFFVKE-----SSLDILQHANE--DINTLDCRISAY : 115
Turtle TAPBPL     : AWLLLSGAERP-----------HQENPPAAD--TLTFIVQES----SSLDILQHVDE--DTDKLECKINRY : 111
Opossum TAPBPL    : IQLVLSGSES-------------QSLSHQGN--SATFLLQD-----RAGVLLPHMDE--DVDKLECKVSRY : 108
Salmon TAPBPa_#C  : SLLYINTDPESEE------TKSQQGPSADIN-HDRVYYVTDPA-AILCSSSLHPPEG--SVHKPQCEINPF : 113
Pike TAPBP        : SLLYINTDPEWKR------PESQQRPSTDIN-XDRLYYITDPA-ATFCSSSLHPPEG--SVQKPQCEINPF : 113
Zebrafish TAPBP   : SLMFIRTEAYSEE------TKAELHPPADIS-SSRVYYVTDPA-GTFCSAALNPPKG--SVNKPKCEINPF : 113
Gar TAPBP         : SLLYVRTEPDP--------VGLQQNPPSDID-PKRIYYVTDAG-ASLCSPSLRAAEG--AVEKPQCEVSPF : 111
Chicken TAPBP     : ALLRFGGDAET-------------PPEPGPE-PEVTFNVSD-----PWGTLTPLGVP--PRTPPSCELNPT : 104
Human TAPBP       : GALLLRQGPGE------------PPPRPDLD-PELYLSVHDPA-GALQAAFRRYPRG---APAPHCEMSRF :  96
                           *                     40         *          60           *
                                                                                      TN3
```

```
                       *                      120       *         140             *
Human TAPBPR      : FLQMTETTV-------------KTAAWFMANMQVSGGGPSISLVMKTPRVTKNEA------LWHPTLNLPL : 179
Chicken TAPBPR    : SFQQ-------------EGNGLCSASWFLATIRL-SSGISIVLLLRGPSCSSQKE--GHDVTLHPKLRIPM : 169
Salmon TAPBPR     : FPRN-------------AKEGSTEPAYFIGSLQIEGGGLSLTLILQTLPLDQS----DRPALMQSKLELPL : 169
Zebrafish TAPBPR  : VPR--------------GADMDSLPAHFIGSIQLEGGGISLTLVLQTLHSETTQS--DAQPLMQSKLNLPL : 192
Gar TAPBPR        : FPRGPE-----------GTEPSPEDAFFIGSLQLEGGGVSLTLVLRTQPIPAEQEEDGGRPLRQSKLDLPL : 179
Medaka TAPBPR     : SFK--------------GLQDSSDQTFFMVSITVEELDFSISLILETLPVEKHQS-----TLMHNKLGLPL : 167
Salmon TAPBPL1a   : STESPQMRW-----PGLGAQEH--DIWFTCTIRHTDGVFIITSFLRHTPATPTPG------QAHYLNWAAI : 160
Salmon TAPBPL1b   : STEGIQVRW-----PGLGAQDH--DIWFTCTLRHTDGLFVITSFLRHTPATPTPG------QADYRNWAAI : 161
Zebrafish TAPBPL  : STGGIVMRW-----PVAGAQEY--DVWFTCTLRHTQGLFVITTFLRHTPATPAQG------QVDFLQWMSV : 168
Medaka TAPBPL     : STEGIHVRW-----PALGAEGY--NRWYTCILRHRKDKFSVTAFLRHPSDQPPPG------QQDYRSWPPI : 157
Salmon TAPBPL2    : SKGMSRVRW-----PSLGGTGH--DIWFTCTLRHTAGLFNITSFLRVTPATTMSA-----HQPDFLSWLTI : 156
Gar TAPBPL        : STGGIQVPW-----KGTAGPD---SAWFTCTLRHSANEFIVTAFLLYPSPAPQQ---------------PI : 159
Frog TAPBPL       : FTDNTQIIW-----PGIHVIENMEEAWFVSRFKHPNDKFQFTLFFTHISQKSLEE-------QDGSQLQAF : 164
Chicken TAPBPL    : FTANTQILW-----PGRKVKAHGLDTWFTCTIEHAAGKYTATAFLVQGHEDRE---------HQTPGQLHQ : 180
Kiwi  TAPBPL      : FTANTQIIW-----PGREIRASSLDSWFICTIKHTAEKYMTTAFLVQTHKENE---------NHSQGQLSP : 172
Turtle TAPBPL     : FTDNTQILW-----PGLQTQLHKLDSWFTGTIKHLADKFTVTVFLVQSSASKEAE-------NRVPEDVSQ : 170
Opossum TAPBPL    : FTANTQILW-----PGLPPRQAQLPTWHLVTISHTDAQFKASTFCFQSAESAQGT---------------- : 158
Salmon TAPBPa_#C  : MPQPSTVQWVVPLTDSAHSPIYLQADWYSAALQGLDGQLGLSSVMRAPTATKE------------------ : 166
Pike TAPBP        : MPQPSSVQWTVSLTDSAHSPIYLQADWYSASLQGLNGQLSLSSVMRAPTATKE------------------ : 166
Zebrafish TAPBP   : MPHASMVRWASALTDSAQSPVYLQADWFSVAAQGLDEQLTLSNIMRAPSASKE------------------ : 166
Gar TAPBP         : LPQPALVRWAAPLTAAGSSPAHLQADWLSAALRGLQGQLVLSSLLRAPSASDQ------------------ : 164
Chicken TAPBP     : NPQTGSDPWSRPLHPDARSPPTAGGQWWVAAVG--TPQYGVTALLQG----------------------GM : 151
Human TAPBP       : VPLPASAKWASGLTPAQNCPRALDGAWLMVSIS--SPVLSLSSLLRPQPEPQQEP---------------- : 149
                       80        *         100         *         120
                    ERp57 interaction C95 disulfide link to ERp57
```

```
                               IgV domain (QVM)
                                                   C3
                    160       *        180       *        200                  *
Human TAPBPR     : SPQGTVRTAVEFQVMTQTQSLSFLLGSSASLDCGFSMAPGL-----------DLISVEWRLQHKG-RGQLV : 238
Chicken TAPBPR   : SKEGTLLTTVEFQSSSNNTSLRTRLGSSITLDCHFALAPSF-----------LLSSLEWRRQHRG-SGRSL : 228
Salmon TAPBPR    : SQSGTLLTEVVFLVFSRSESHSAPLGGVALLDCGFRQQAPPP---------GWELGLEWRLQHRG-SGRKV : 230
Zebrafish TAPBPR : SQSATLLTEVVFVVFSRVPSIVALIGGDVMLDCGFRQKESVP---------GQDVALEWRLQHRG-NGRKI : 253
Gar TAPBPR       : SPSGTILNEVVFVVFTRLQSLTAPLGGSALIDCGYKEATPS-----------QEVALEWRLQHKG-HGRRI : 238
Medaka TAPBPR    : SQSGTLLTKAIFVVFSNTKSLSAPLRGNVVLDCGFKQQEVPL---------AHQVKLYYHKIHVD-HPKKV : 228
Salmon TAPBPL1a  : ADRAALTTSTVMLVLTRSPSVRVGLVKQQSLHCQFDVDHKA-----------ADLTVEWRFQRRG-ERTTL : 219
Salmon TAPBPL1b  : ADREMLTTSTVMLVFTRTPSVWVGLMKQSSLHCQFDVDHKA-----------ADLTVEWRLQRRS-ERTTL : 220
Zebrafish TAPBPL : SDRDLLTTTAAMVVLTRTPSVEVGFLKEPELHCQFAVDHKL-----------PNATVEWRLQRHG-ERTKL : 227
Medaka TAPBPL    : ADREVLTTTVAMLLKTETPSVKVRLRSQPKLHCQFSIDHRG-----------PNVTVEWHWQSRG-ERRML : 216
Salmon TAPBPL2   : GVKEQISASAVMVLMTRSPSVRVGLQEERTLHCQFAVDHKV-----------PHLTVEWHLQRHGARRTKL : 216
Gar TAPBPL       : GDADTLNTSVAMVVLTRTPTVQAGLLQDARLDCGFAVDHAQ-----------PDVTVEWRFRHRG-EQRKL : 218
Frog  TAPBPL     : PNAEIRYVLPVFMLSTQTPHVQTRLQEDALLNCDFSIDHHA------------DVNIVWKLLKKGGQEMKL : 223
Chicken TAPBPL   : GIAEQTRVSAVLAARTRLPRVRTALGKDVVLDCAFAADPRA------------AVAVRWALRKKG-RHERY : 238
Kiwi  TAPBPL     : GIAEQSHVSAVFLVHTRPSVVRSALSKDVLFDCAFSIDHQA------------DVTIQWVLHRKGGHKKLM : 231
Turtle TAPBPL    : GNAERFYLSGVFLVRTGPSLIQSGLNKDVLLSCAFSVDHHT------------DVTIKWVLQQKGGHKKLI : 229
Opossum TAPBPL   : ---GPAIFSGVFSLYTKASRVQVELYGTVLLACSFVMDHAP-----------EALDVRWALRQKGGKQREI : 215
Salmon TAPBPa_#C : -------PTVVLSVSSRTPLVRSRLGEPVVLDCGFWMEATSPL-------SGSGFAVEWRYQFRG-DGRLV : 222
Pike TAPBP       : -------PAVLLSVSSRTPVVRSRLGERVVLDCGFWAEASSPL-------SGSGFTVEWRYQFRG-DGRLV : 222
Zebrafish TAPBP  : -------PEVILSVSSKTPVVRCRLGEPVLLDCGFWIDPSSPL-------HGSGFSIEWRYQFRG-EGRLV : 222
Gar TAPBP        : -------ASVVLSVFSRRLVVRTRLGAPALLDCGFWARPGSPL-------WGAGFAVEWRYQFRG-EGRLV : 220
Chicken TAPBP    : GTEGTITAAVALAVLTHTPALRARVGSPIHLHCAFAAPPSP-------------FVLEWRHQNRG-AGRVL : 208
Human TAPBP      : --VLITMATVVLTVLTHTPAPRVRLGQDALLDLSFAYMPPTSEAASSLAPGPPPFGLEWRRQHIG-KGHLL : 217
                     *         140       *         160       *         180       *
                                                                         TN6    TN7


                                                                          C4
                     220                *         240            *         260         *
Human TAPBPR     : YSWT-AGQGQA--------VRKGATLEPAQLG------MARDASLTLPGLTIQDEGTYICQITTSLYRAQQ : 294
Chicken TAPBPR   : FRYR-VGNAGLT-------AQPKVHVDVKQLL------GNGDASLTLQEATVNDEGTYICLVSTAQHQVQH : 285
Salmon TAPBPR    : LEIR-AGQTETEEGPAVHVEREGSSVDAALLV------GQGNASLTLARLKVSDEGTYICTVSTGLYQAQQ : 294
Zebrafish TAPBPR : LDMK-AREMETEIGSDVFVDRENTSTEADLLV------RDGNASLTLRTLQVSDEGTYICTVGSGDFQTQQ : 317
Gar TAPBPR       : LQLR-AGREGEE--PTVHPEREGASVEPGLVL------EEGNVSLTLTNVKVADEGTYICTVGSGVYQAQQ : 300
Medaka TAPBPR    : YNTN-GHHLTLGFVSSVNVERKDSSMNAGLVV------REGNVSLTLPALKVSDEGTYICTVSIGHFHAQQ : 292
Salmon TAPBPL1a  : FSHS-SRSGQTE----------GSGVPLNAI-------GRGDASLTLPLTKQSSEGTYVCHVSVPPLFGSH : 272
Salmon TAPBPL1b  : FSHS-SRSGQRE----------GGGVELKGI-------GRGDASLTLPLTKQSSEGTYVCLVSVPPLFGSH : 273
Zebrafish TAPBPL : FSYS-SRTGKSE----------GKGVTVKTI-------AGGNASFKLEPTRKHSEGTYVCSVMVPPLYGSH : 280
Medaka TAPBPL    : FRYN-SRSGEIH----------GSGVGKKAL-------AGGDASYTLPFTKMSSEGSYICSLTVNPLFANM : 269
Salmon TAPBPL2   : FSYS-SSSGQTE----------GSGVAVKGI-------AGGDASLTVPITKVSSEGTYVCSVRVPPLNGSV : 269
Gar TAPBPL       : FSYS-RRSGRAET------EVPGATAAARDV-------SRGDASLRLSAVKVKSEGTYVCSVYVPPLYGSH : 275
Frog  TAPBPL     : LTYK-GSEKSVVY------HVKGIAMYVDEV-------PKGSASLVVKNVDLEKEGLYTCSVSVNSLFGDQ : 280
Chicken TAPBPL   : IAT----------------SGGRAEMFPQE--------PRGNASLLIRRVELSDEGTYICTVEAAALVLEQ : 285
Kiwi  TAPBPL     : FTYN-GSSKQVEH------MADRAEMFLEEI-------PKGNASLLLRNVEMRDEGTYSCSVSVSSLTGEQ : 288
Turtle TAPBPL    : FAYN-GFTRQVEH------EDKRAEMFLAEL-------PKGNASLLLRTVGVRDAGTYSCSVLVSSLLWAQ : 286
Opossum TAPBPL   : LKYD-GKQRQVTH------LHEQVEAFPSEI-------PGGDASIRLNNVAVNDQGDYFCSVSTAGLYWEL : 272
Salmon TAPBPa_#C : LAYD-GKTDRFAE-----TKEKRAGLDFTALH------ETGNASLILQEAQVRHTGTNICTVYLPYLLAQV : 281
Pike TAPBP       : LAYD-GKNDRLAE-----TQEKGAELDFTALH------ERGNASLILQEAQVRHSGTYICTVYLPYILXQV : 281
Zebrafish TAPBP  : LAYD-GKNDRFAE-----TSESGAEMDITGLY------QTGNASLILEESQVRHSGTYICTVYLPHLLAQV : 281
Gar TAPBP        : LAYD-GRGDRLEE------AEPGAELDAAEAH------RTGNVSLRLGGAEVRHAGTYICTVYLPYLLAQV : 278
Chicken TAPBP    : LAYD-SSTARAPR------ATPGAELLLGTRD----GDGVTAVTLRLAQPSPGDEGTYICSVFLPHGHTQT : 268
Human TAPBP      : LAATPGLNGQMPA------AQEGAVAFAAWDDDEPWGPWTGNGTFWLPTVQPFQEGTYLATIHLPYLQGQV : 282
                     200       *               220       *         240       *         260
                    ERp57 interacting sites                                   TN5        TN6
                                                          N-linked glycosylation site
```

```
                           IgC domain (PKV)
                                                  C5
                          280       *             300        *           320
Human TAPBPR      : IIQLNIQASPKVRLSLANEALL---------PTLICDIAGYYPL-DVVVTWTREEL------GGS--PAQV : 347
Chicken TAPBPR    : NIQLLVSEPPRVRVFPTE--AS-LKRDET--ITLTCNIAGYYPL-DISVSWTQKTP-------EDEVEISP : 343
Salmon TAPBPR     : VIQLHVTQPPRVSLSEEK--LV-FRDELP--QKLSCHCKNYYPL-DVQMEWISVSS------TDSEPSVLS : 353
Zebrafish TAPBPR  : IVQLHITQPPRITLSEEK--LS-FPDRTP--QRLSCHCNRYYPL-DVQVEWFSQAP------SSEESISLS : 376
Gar TAPBPR        : VIQLHITQTPSVTLTPNQ--LV-FQDDTP--QRVICHCDHYYPL-DVQVEWFSLSP------SASEPVPIS : 359
Medaka TAPBPR     : VIQLHIIQPPHISLSEEK--LM-LK--TP--QTLKCFSRNYYPL-DAQMEWLFVSP------TATEPELFK : 349
Salmon TAPBPL1a   : DITLQIMESPRVSLNVDS-TIS-LVDRGE--QKVVCEAEGYYPL-DVEMEWFREPS------GGGLLPEKL : 332
Salmon TAPBPL1b   : EIALHIMEPPRVSLNVDS-VIS-LVDTER--QKMVCEAVGYYPL-DVEMEWFREPS-GG---GGGLLPEKL : 335
Zebrafish TAPBPL  : DIPLSLSEQPRVSLNVGS-TLS-MVIGQD--QKLICEAESYYPL-DVDIEWYREPH------GGSPTPLFL : 340
Medaka TAPBPL     : ELNLHVEEPPRVSLNVGP-TLV-LEEGKE--QKVVCTAENYYPL-DVEILWHEQNPAASGQRVGAPLPQEL : 335
Salmon TAPBPL2    : DIALHIIEPPNVSLTTKE--------EQI--PRVVCEANGFYPL-DVDIDLFKETS-------SGQRPEKL : 322
Gar TAPBPL        : DVQLQIMESPRVSLSVAT-ALA-LQEEEE--QKVVCEITGYYPL-DVQVDWLHEQG------GGGRLPQVI : 335
Frog  TAPBPL      : VIHLEVVEPPIVNLNVKS--LL-LTEGQE--QKLVCEASKYYPL-DVNIEWLREGR------DQTLLPTVL : 339
Chicken TAPBPL    : AIQLQITEKPTVTVNVNS--LS-LVEGEQ--QKLVCDVRNYYPA-DIHVQWLREPQ------SAGQLPDTV : 344
Kiwi  TAPBPL      : AIQLQIEEKPTVIVNVNS--LS-LVEGEQ--YKLICDIRNYYPL-DAQAQWLRELK------GSRKVPDVV : 347
Turtle TAPBPL     : NIQLEIVEKPTVTLNADV--LS-LVEGDE--HKLVCDISHFYPH-DADAQWLQEPM------EQGMLPHVV : 345
Opossum TAPBPL    : SIEVAVVEAPKVRFSPQTPILT-LEEGEE--EKLVCDVRHYFPL-EAHVFWLRERL------EGRMMPEGV : 333
Salmon TAPBPa_#C  : AVELEIVEPPSLSIFPSP--LPLSVPGQV--VKVQCEASGFFPL-SLDFHWELT--------GPDGKVRPL : 339
Pike TAPBP        : AVELEIVEPPSLSIFPXP--LPLSVPGQV--VTVQCEASGFSPL-SLELHWELM--------ETDGNVRPL : 339
Zebrafish TAPBP   : AVDLEIVEPPSLSILPSP--LPLLVPGQV--LSVQCEASGFAPH-TLDLGWEFT--------GADGKSLSL : 339
Gar TAPBP         : AMELEVVEPPALLLSPSP--LW-ALPGHS--APVQCEASGFFPL-ALELQWEFLPA------GGAGEALAL : 337
Chicken TAPBP     : VLQLHVFEPPKVTLSPKN--LV-VAPGMS--AELRCHVSGFYPL-DVTVTWQRRAG-SSG--TSQSPRDTV : 330
Human TAPBP       : TLELAVYKPPKVSLMPAT--LARAAPGEAP-PELLCLVSHFYPSGGLEVEWELRGG------PGGRSQKAE : 344
                           *          280         *          300       *            320


                         TC2-TC3                                C6
                      *         340         *          360         *        380
Human TAPBPR      : SGASFSSLRQSVAGTYSISSSLTAEPG----SAG-ATYTCQVTHISLE-EPLG-ASTQVVPPERRT----- : 406
Chicken  TAPBPR   : SNTRFSSHRQSQDGTYSINSYLSVNLATA--QAP-ATYTCHVSHVALE-APIS-ISTHLKAPEHTE----- : 404
Salmon TAPBPR     : DQVSLSSHRQHSDRTMSISSHLTLHPSTF--PPG-TTVTCRVTHPALD-TPLS-LSLTVETPEPD------ : 413
Zebrafish TAPBPR  : KDSSLSSHRQHSDGTFSVSSHLTLLPDKH--PPG-TVVTCRVSHPSLE-TPTN-ATLTVEEPEPDT----- : 437
Gar TAPBPR        : NGVYFSSHRQHSDGTYSLSAYISVSPSED--LTG-ATFSCIVSHHSLS-EPIT-ASITVSAPEES------ : 419
Medaka TAPBPR     : DQGSLTSHRQHSDGTFSLSSHLVVPPAV---PPG-TKIICRVSHVGLD-APLD-VSVLVETPKID------ : 408
Salmon TAPBPL1a   : DTVLYSSHRHHQDGTYSLSAFFLLHASLH--DSG-SKYFCRVSHSSQR-MPIR-KSFTLNVTEYDSWN--A : 396
Salmon TAPBPL1b   : DTVLYSSHRHHQDGTYSLSAFFLLHASLH--DSG-SKYFCRVSHSSLR-MPIR-KSFTLIVTEVDSWN--- : 398
Zebrafish TAPBPL  : KNVLYSSHRIHQDGTYSLSAFFYLQPGLE--DSG-YKYTCRVSHKSLL-TPVR-KSFYLVVTEPDS----- : 401
Medaka TAPBPL     : KNILFSSHKHNQDQTYSLSAFFYLKASLT--LSG-KQFSCSVSHPSLR-MPIK-KSFILQVEEPSSLM--- : 398
Salmon TAPBPL2    : GNVLHSSHIQHHDGTHSVAAFVRLRPSPQ--DSGCETYSCRVSHVSLQGLYIH-KSII----GPGC----- : 381
Gar TAPBPL        : KNILFTSHRHHADGTYSISAFFILRPSLA--DHD-ARYTCRVSHVSLR-TPIR-KSFTLTVTERLRIGSIL : 401
Frog  TAPBPL      : KNVLYSSHKHNNDGTYSLSSFFIFTASQL--DNG-AVLTCRVEHMSLK-HPIR-KSVKVRVEESPSY---- : 401
Chicken TAPBPL    : PNVLTSSHLRSSNGTYSFSRFFLLTATLR--DNG-HTYTCRVEHSSLQ-APIR-RSITVAVREATS----- : 405
Kiwi  TAPBPL      : KNVLSSNHRQSSNGTYSFSRYFLLTASLE--DNE-HTYTCRVDHQSLQ-APIR-RSVIVQVRGTS------ : 407
Turtle TAPBPL     : THVVSSSHRHNSDGTYSFSSYFLLKASLR--DDG-RRYTCRVEHQSLK-HPIK-KSLTVKVTESTS----- : 406
Opossum TAPBPL    : KNVLFSSHRKNGDETFSFSSYYLLKASLQ--DDG-FRYTCLVEHEGLR-FPIR-KSIIIRVTEKSG----- : 394
Salmon TAPBPa#C   : GQGSVTGHRQGPDNTYSQTSRLELDSAKLDLGRG-GEVTCVAVHPGGT----RRASVTLNVIGINGPSIED : 405
Pike TAPBP        : GQGSVSGHRQGPDGTYSQTTRLQLDSAKLDLGRG-GEVTCVAVHPGGT----RRARVTLNVIGVSAPSIED : 405
Zebrafish TAPBP   : GQGSVTGHRRASDGTFSQSSRLELDSTKLRLARG-GEISCVAKHDGGT----RRASAALNVIGVAAPSIED : 405
Gar TAPBP         : EGATVSGHRQGRAGTFTQTSRLELDLGRLRLEHG-GQLVCVAKHEGGT----RRPGVTLNIAGVGGPTVED : 403
Chicken TAPBP     : MDSWTSGHRQAADGTYSRTAAARLIPARPQ-HHG-DVYSCVVTHTALA-KPMR-VSVRLLLAGTEGPHLED : 397
Human TAPBP       : GQRWLSALRHHSDGSVSLSGHLQPPPVTTE-QHG-ARYACRIHHPSLP-ASGRSAEVTLEVAGLSGPSLED : 412
                        *         340         *          360          *        380         *
```

```
                       *            400       *         420       *         440
Human TAPBPR     : ALGVIFA--SSLFLLALMFLGLQRRQAPTGLGLLQAERWETTSCADTQSSHLHEDRTARVSQPS- : 468
Chicken TAPBPR   : LEGLVGGAIATAIFVSVLFIVLRRKRAAEPKPEQLLTASE------------------------- : 444
Salmon TAPBPR    : ---SYWMVLGFLVITVLFFYQVMK----------------------------------------- : 434
Zebrafish TAPBPR : ---AFWTISLMLLISVVFLYQAFK----------------------------------------- : 458
Gar TAPBPR       : QLWSMVGGVLSFVLFLFGLFMLLR----------------------------------------- : 443
Medaka TAPBPR    : ----SYWWFLGFLIVTILFFYSFKK---------------------------------------- : 429
Salmon TAPBPL1a  : ALWFFSGFGFILVMVATLFVML-PRLSSARKANQRKPY--------------------------- : 433
Salmon TAPBPL1b  : FLWLFFGCGFILVMVATLCVML-PRLSSARKANKRKPY--------------------------- : 435
Zebrafish TAPBPL : TMWYIAVFGFIIAMLVILCYML-PQVLAGRR--SKKMF--------------------------- : 436
Medaka TAPBPL    : FNLAVGLVLISLSGVLIILLLY-LNSARRPSKIVRNHTESQW----------------------- : 439
Salmon TAPBPL2   : WTWIHLTLPGLVCLIFVIIFIAVTRQRFF------------------------------------ : 410
Gar TAPBPL       : FWIFVAFIVMMLLLLLVILLP--YLHSARREAKKKKPY--------------------------- : 437
Frog  TAPBPL     : QEFFLVI--LIIFLSVVLLTLILH----------------------------------------- : 423
Chicken TAPBPL   : TTWLLLL--LLLGLTGCLVASL-HHLHQVRSTTKPKPY--------------------------- : 440
Kiwi  TAPBPL     : IIWVLL---LLLGLSVCLAVVL-HYFYKVKSTAKPKPY--------------------------- : 441
Turtle TAPBPL    : ATWFLVI--LLVLLAGCLVVTL-CYLYRGMGPNKPKPY--------------------------- : 441
Opossum TAPBPL   : SSWGLLI--ILIILMVVIFVLL-RYLHQVKSMNKTKPY--------------------------- : 429
Salmon TAPBPa_27 : SMAMVAVALGLYGLIKIVSWTF-SSGSDDTNSQEKKVK--------------------------- : 442
Pike TAPBP       : SMAMVAVALCLYGLIKVISWTFI---SSGSEDAEKKAK--------------------------- : 440
Zebrafish TAPBP  : SMAMVAVALLLYGMIKFLSWTFSSSDSGDSELNDKKEK--------------------------- : 443
Gar TAPBP        : SMAMVAVALLLYGLIKIVSWTLKTTDSSVPEEPEKKQE--------------------------- : 441
Chicken TAPBP    : ITGLFLVAFVLCGLIRWLY-----PKAARPKEETKKSQ--------------------------- : 430
Human TAPBP      : SVGLFLSAFLLLGLFKALGWAA--VYLSTCKDSKKKAE--------------------------- : 448
                          400       *            420
                                  TAP association    ER retention motif
```

References:

1. Dong G, Wearsch PA, Peaper DR, Cresswell P, & Reinisch KM (2009) Insights into MHC class I peptide loading from the structure of the tapasin-ERp57 thiol oxidoreductase heterodimer. *Immunity* 30(1):21-32.
2. Hermann C, Trowsdale J, & Boyle LH (2015) TAPBPR: a new player in the MHC class I presentation pathway. *Tissue antigens* 85(3):155-166.
3. Morozov GI, *et al.* (2016) Interaction of TAPBPR, a tapasin homolog, with MHC-I molecules promotes peptide editing. *Proceedings of the National Academy of Sciences of the United States of America* 113(8):E1006-1015.
4. Rizvi SM, Del Cid N, Lybarger L, & Raghavan M (2011) Distinct functions for the glycans of tapasin and heavy chains in the assembly of MHC class I molecules. *Journal of immunology* 186(4):2309-2320.
5. Sadasivan B, Lehner PJ, Ortmann B, Spies T, & Cresswell P (1996) Roles for calreticulin and a novel glycoprotein, tapasin, in the interaction of MHC class I molecules with TAP. *Immunity* 5(2):103-114.
6. Paulsson KM, Jevon M, Wang JW, Li S, & Wang P (2006) The double lysine motif of tapasin is a retrieval signal for retention of unstable MHC class I molecules in the endoplasmic reticulum. *Journal of immunology* 176(12):7482-7488.
7. Neerincx A, *et al.* (2017) TAPBPR bridges UDP-glucose:glycoprotein glucosyltransferase 1 onto MHC class I to provide quality control in the antigen presentation pathway. *eLife* 6(e23049).

Additional file 13: Figure S9. PSME1 and PSME2 data

Table of Contents		Page
S9a	Phylogeny of deduced PSME1, PSME2 and PSME3 amino acid sequences	1
S9b	Alignment of deduced PSME1, PSME2 and PSME3 amino acid sequences	2

S9a. Phylogeny of deduced PSME1, PSME2 and PSME3 amino acid sequences

The evolutionary history was inferred using the Neighbor-Joining method (1). The percentage of replicate trees in which the associated taxa clustered together in the bootstrap test (100 replicates) are shown next to the branches (2). The tree is drawn to scale, with branch lengths in the same units as those of the evolutionary distances used to infer the phylogenetic tree. The evolutionary distances were computed using the Poisson correction method (3) and are in the units of the number of amino acid substitutions per site. All ambiguous positions were removed for each sequence pair. Sequence accession numbers are shown in parenthesis and Atlantic salmon (Salmon) sequences are shown with red font.

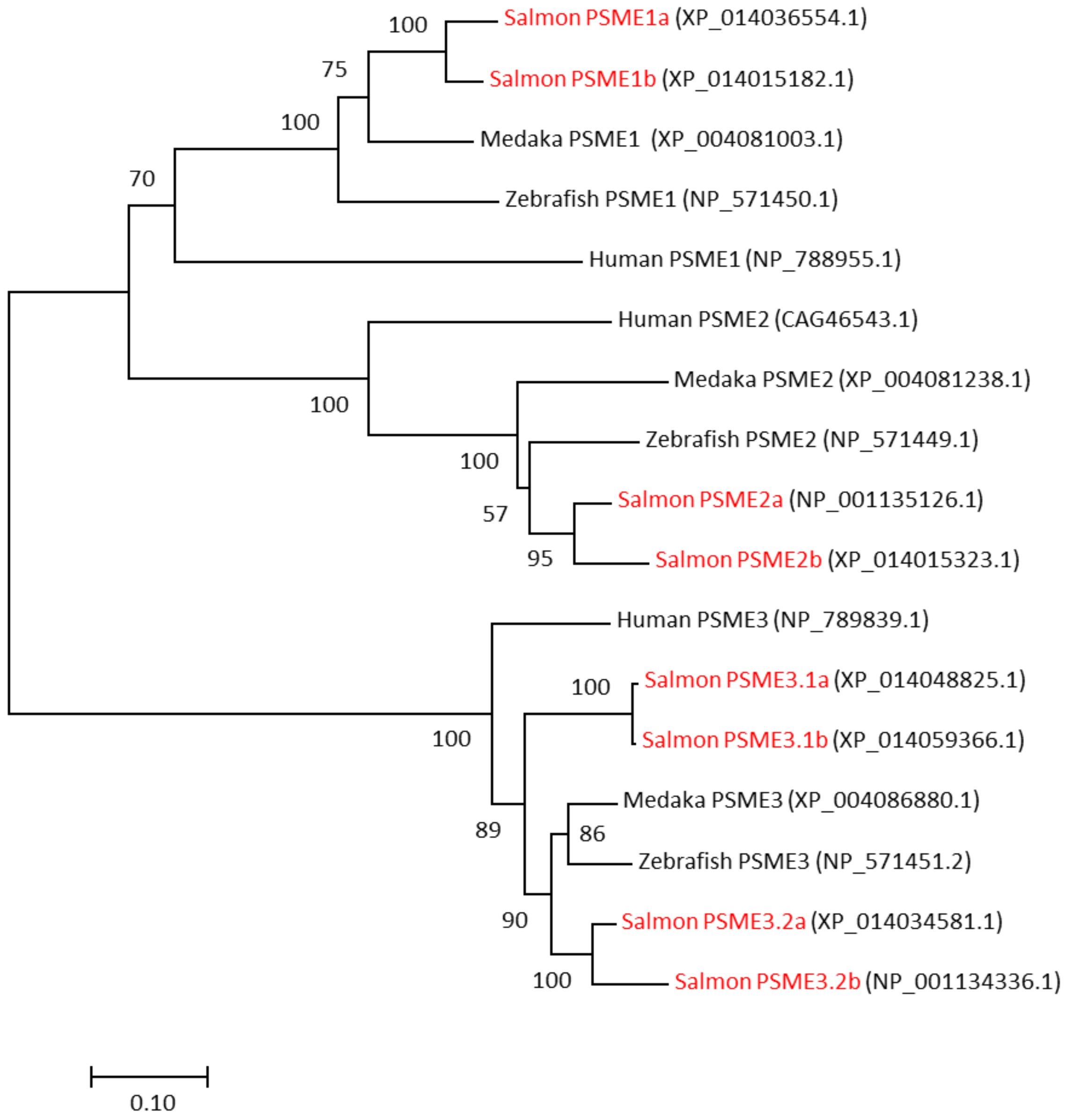

Figure S9b. Alignment of deduced PSME1, PSME2 and PSME3 amino acid sequences

Alignment of deduced PSME1, PSME2 and PSME3 amino acid sequences from Atlantic salmon (Sasa), medaka (Orla), zebrafish (Dare) and human (Hosa). Sequence references are shown in Figure S9a. Amino acid residues are colour coded according to physiochemical properties.

```
                       *        20         *        40         *        60
HosaPSME1     : MAM---LRVQPEAQAKVDVFREDLCTKTENLLGSYFPKKISELDAFLKEPALNEANLSNLKAPLDIPVP :  66
SasaPSME1a    : .TS---IDIR..SKKM..D.CTR.TKEA.T.VT.F..Q..A.MEML..K-SFSTDG.AA..S.....M. :  65
SasaPSME1b    : .TS---IDIR..SKKQ..D.CTQ.TKEA.T.VT.F..Q..A.M.ML..T-S.STEG.AA.......I. :  65
OrlaPSME1     : .TS---.DIRH.SKKQ..E.CQT.SKEA.E..SKF..E..EQ.QML..T-SF.CDD.AS.......I. :  65
DarePSME1     : .TS---.DMS.ASKKQ..G.SQKITKEA.Q.ISKI..E..A.M.NV.QG-SCSLKD..VI......I. :  65
HosaPSME2     : ..KPCGV.LSG.VRKQ.E...QN.FQEA.EF.YRFL.Q..IY.NQL.Q.DS..V.D.TS.R......I. :  69
SasaPSME2a    : .SRSSV.KIKSANAV..EN..QS.YQQA.D.FSN.I.L..TQ..NL...ED.SITD..T.H......I. :  69
SasaPSME2b    : .SKSSVMKIKSVNAV..EH.HHS.YQQADD.FSN.I.L...Q..NL...DD..IPD..T.Q......I. :  69
OrlaPSME2     : .SKAAT.KIKSASAV..EN..QS.YHEAG..FSNLI.Q..IQ...L.RDD.FILTD--S.Q......I. :  67
DarePSME2     : .SKANV.KLNSDNAVRIENY.QS.YKQA.D.FSN.I.L...H..NL..GDEFSITD..S.H......I. :  69
HosaPSME3     : ..S--L.K.DQ.VKL...S...RITSEA.D.VANF....LL...S.....I..IHD.TQIHSDMNL... :  67
SasaPSME3.1a  : .SSK-GIK.DNDLKT...A...RITGEA...VADF....LL...S.....I..V.D.KEIHSEINVK.. :  68
SasaPSME3.1b  : .SSK-GIK.DNDLKT...A...RITGEA...VADF....LL...S.....I..V.D.KEIHSEIN.K.. :  68
SasaPSME3.2a  : .NS--L.K.DN.LKT...A...RITGEA.E.VA.F..N.LL...H...D.G..ICE.KEIHSEINLT.. :  67
SasaPSME3.2b  : .TV--VVF..------..A..QRITGEA...VA.F..N.LL...H...D.I..ICE.KEIHSEINLT.. :  61
OrlaPSME3     : .SS--L.K.DS.IKT...A...RITAEA.D.VANF....LL...S...D.SI.IVE.KDIHSEINLT.. :  67
DarePSME3     : .SS--L.K.DN.IKT...A...RITSEA.D.VANF....LL...Q...D.TI.ITE.QEIHSEINLT.. :  67

                *        80         *       100         *       120         *        1
HosaPSME1     : DPVKEKEKEE---RKKQQEKEDKDEKK----KGEDEDKGPPCGPVNCNEKIVVLLQRLKPEIKDVIEQL : 128
SasaPSME1a    : ..A..EA.-----...KE...A.EG..EKDSDK....A..A...IP...RVES..KEI..Q.QILK.K. : 129
SasaPSME1b    : ..A..EA.-----Q..KE...A.EG..DKDSEK....A......IP...RVES..KEI..Q.QLLK.K. : 129
OrlaPSME1     : ..A..E..-----...KE..DA.EG..DKDSDK....A......IYS..RVES...EV....QTLK.K. : 129
DarePSME1     : .....EL.-----...KE...A.EG..----DK....A......IA...TVEK.IKQI....QTLK.C. : 125
HosaPSME2     : ..P-P.DD------EMETD.-------------QEKKEV.K..FLPG...VLS..ALV...VWTLK.KC : 118
SasaPSME2a    : ..PTPEDE------EMETD.N.---------DD.KKK.A.K..FIKG.....K..D.V....LALR.TI : 123
SasaPSME2b    : ..PTAEDE------EMETD.N.---------DKKKKK.A.S..LIKG.....K..D.V....LSLR.TI : 123
OrlaPSME2     : E.PSPE.E------DMETD.DE---------D.-KKK.A.K..FIKG....MT..E.V....VALR.TI : 120
DarePSME2     : ..PAPEDE------EMETD.NE---------DD.KKK.A.K..FIKG..R..K..DIV....MGLK.TC : 123
HosaPSME3     : ..ILLTNSHDGLDGPTYKKRRLDEC--EEAFQ-GTKVFVM.N.MLKS.QQL.DIIEKV....RLL..KC : 133
SasaPSME3.1a  : ..IILNNSHDGVDVQNSRKRKME.GLEDDNCQDGPKVFAM.G.MIKS.GQL.D.IE.V....RTL..KC : 137
SasaPSME3.1b  : ..IILNNSHDGVDVQNSRKRKME.GLDDDNCQDGPKVFAM.G.MIKS.GQL.D.IE.V....RTL..KC : 137
SasaPSME3.2a  : ..IILSNLHDGLEAQNAKKRKME.GSGEDKVA-GTKVFVM.G.MMKS.A.L.D.IEKV....RTL..KC : 135
SasaPSME3.2b  : ..ILLSNLHGGLEAQNAKKRKME.GTGEDKVA-GTKVLIM.S.MMKS.A.L.G.IEKV....RTL..KC : 129
OrlaPSME3     : ..ILIPNLHDGLEAQNAKKRKLE.GSGEDKMT-GTKVFVM.G.MMKS.GNL.D.IEKV....RTL..KC : 135
DarePSME3     : ..ILLTDIHDGIEGQNAKKRKFE.GAGDDKVG-GTKVFVM.G.MMKS.G.L.E.IEKV....RTLM.KC : 135

                 40         *       160         *       180         *       200
HosaPSME1     : NL--------------VTTWLQLQIPRIEDGNNFGVAVQEKVFELMTSLHTKLEGFHTQISKYFSERGDA : 184
SasaPSME1a    : .T--------------.SM.V.....K.................L.NTR..I...Q......Y...... : 185
SasaPSME1b    : .T--------------.SM.V.....K.................L.NTR..I.E.Q......Y...... : 185
OrlaPSME1     : .T--------------.SM.V.......................L..TR..I.AIQ......Y...... : 185
DarePSME1     : .T--------------.SM.I...V.....................NTR..I...Q......Y...... : 181
HosaPSME2     : I.--------------.I..I.HL..K.....D....I....L.RVNAVK..V.A.Q.T.......... : 174
SasaPSME2a    : IT--------------.SC.I.HL..K.....D....I...IL.RIVAVK..VD....N.N........ : 179
SasaPSME2b    : IT--------------.SC.I.HL..K.....D....I...IL.RIAAVK..VD....N.N........ : 179
OrlaPSME2     : IT--------------.SC.I.HL..K.....D....I...IL.RIAAVK..VD..Q.N.N........ : 176
DarePSME2     : IT--------------.SC.IAHL..K.....D....I...IL.RI.AVK..V...Q.N.N........ : 179
HosaPSME3     : .TPSGKGPHICFDLQ.KM.V..L...........SI..ETVAELRTVESEAASYLD...R.YIT.AKL : 202
SasaPSME3.1a  : .T--------------.KM.V..L...........SI..ETVAELRTVEGEAASYLD...R.YIT.AKL : 193
SasaPSME3.1b  : .T--------------.KM.V..L...........SI..ETVAELRTVEGEAASYLD...R.YIT.AKL : 193
SasaPSME3.2a  : .T--------------.KM.V..L...........SI..ETVAELRTVEGEAASYLD...R.YIT.AKL : 191
SasaPSME3.2b  : .T--------------.KM.V..L...........SI..ETVAELRTVEGEAASYLD...G.YIT.AKL : 185
OrlaPSME3     : .T--------------.KM.V..L...........SI..ETVAELRTVEGEAASYLD...R.YIT.AKL : 191
DarePSME3     : .T--------------.KM.V..L...........SI..ETVAELRTVEGEAAS.LD...R.YIT.AKL : 191
```

```
                   *       220         *       240         *       260         *
HosaPSME1     : VTKAAKQPHVGDYRQLVHELDEAEYRDIRLMVMEIRNAYVRRQGQGRGGQRQLSQATHSLTLQARG- : 250
SasaPSME1a    : .D..S.E..............QYQ.CEL.IV.L....T.AVLLDIINKNYDKIKKPRGDC--K.LIY : 250
SasaPSME1b    : .A..S................QYQ.CEL.IV.L....T.ALLFDIINKNYDKIKKPRGDC--K.LIY : 250
OrlaPSME1     : .A..S................QYQ.CEL..HILD...T.AVLFDIINKNYDKIKRPRGDG--K.LIY : 250
DarePSME1     : .A..S.......F........QHQ.CEL.II.L....T.AMLYDVITKNFDKIKKPRGD.SSK.LIY : 248
HosaPSME2     : .A..S.ET..M...A....R...A.VEL.A..LDL.AF.AELYHIISSNLEKIVNPKGEE--KPSMY : 239
SasaPSME2a    : .S..S.LT..M...S....K...V.S...VILLD..GF.AELYDIISKNLEKVTNPKGEE--KPSMY : 244
SasaPSME2b    : .A..S.ST..M...S....K...V.S...VILLD..GF..ELYDIISKNLEKVTNPKGEE--KPSMY : 244
OrlaPSME2     : .A..S.ET..M...S....K.R.LFS.L.VILLD..GF.AELYDIINKNLEKVTNPKGEE--KSSMY : 241
DarePSME2     : .A..S.DT..M...S....K...A.SE..VI.LD..GF.AELYDVISKNLEKVTNPKGEE--KPSMY : 244
HosaPSME3     : .S.I..Y...E...RT.T.I..K..ISL..IIS.L..Q..TLHDMILKNIEKIKRPRS.N--AETLY : 267
SasaPSME3.1a  : .S.IT.Y...E..QRT.T.I..K..ISLKII.S.L..Q..TLHDMILKNIEKIKRPRS.N--NEALY : 258
SasaPSME3.1b  : .S.IT.Y...E..QRT.T.I..K..ISLKII.S.L..Q..TLHDMILKNIEKIKRPRS.N--NEALY : 258
SasaPSME3.2a  : .S.I..Y...E...RT.T.I..K..ISLKII.S.L..Q..TLHDMILKNIDKIKKPRS.N--AEALY : 256
SasaPSME3.2b  : .S.I..Y...E...RT.T.IG.KK.ISLKVI.S.L..Q..TLHDMILKNIDKIKKPRS.N--AEALY : 250
OrlaPSME3     : .S.I..Y...E...RT.T.I..K..ISLKII.S.L..Q..TLHDMILKNIEKIKRPRS.N--SDALY : 256
DarePSME3     : .S.I..Y...E...RT.T.I..K..ISLKII.S.L..Q..TLHDMILKNIEKIKRPRS.N--TDALY : 256
```

References:

1. Saitou N & Nei M (1987) The neighbor-joining method: a new method for reconstructing phylogenetic trees. *Molecular biology and evolution* 4(4):406-425.
2. Felsenstein J (1985) Confidence Limits on Phylogenies: An Approach Using the Bootstrap. *Evolution; international journal of organic evolution* 39(4):783-791.
3. Zuckerkandl E & Pauling L (1965) Molecules as documents of evolutionary history. *Journal of theoretical biology* 8(2):357-366.

Additional file 14. Figure S10. ERAP data

Table of Contents		Page
S10a	Phylogeny of deduced ERAP1 and ERAP2 amino acid sequences	1
S10b	Alignment of deduced ERAP1 and ERAP2 amino acid sequences	2

Figure S10a. Phylogeny of deduced ERAP1 and ERAP2 amino acid sequences.

The evolutionary history was inferred using the Neighbor-Joining method (1). The percentage of replicate trees in which the associated taxa clustered together in the bootstrap test (100 replicates) are shown next to the branches (2). The tree is drawn to scale, with branch lengths in the same units as those of the evolutionary distances used to infer the phylogenetic tree. The evolutionary distances were computed using the Poisson correction method (3) and are in the units of the number of amino acid substitutions per site. All ambiguous positions were removed for each sequence pair. Sequence accession numbers are shown in parenthesis and Atlantic salmon sequences are shown with red font.

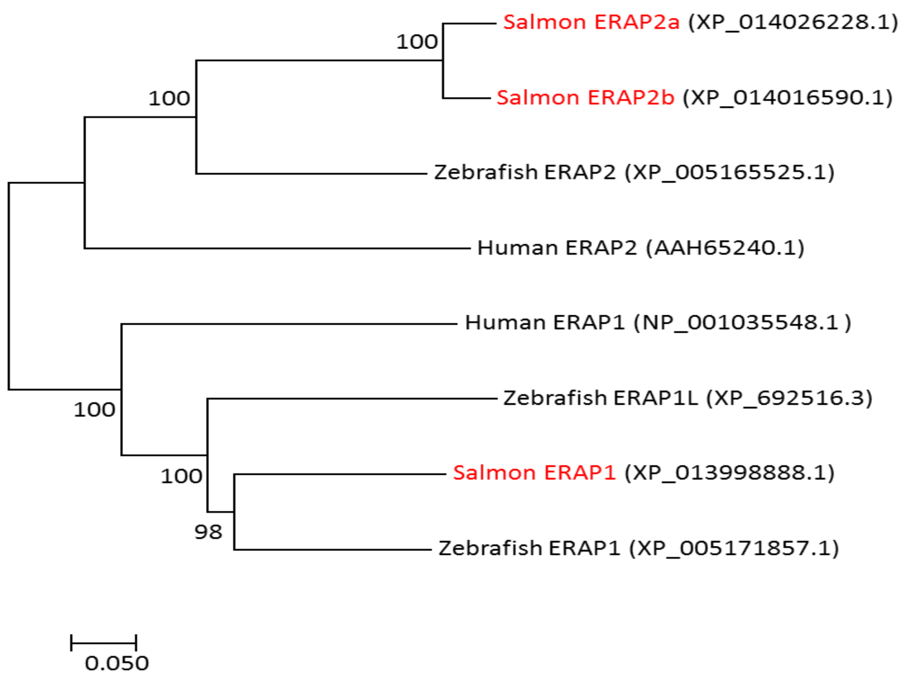

Figure S10b. Alignment of deduced ERAP1 and ERAP2 amino acid sequences

Alignment of deduced ERAP1 and ERAP2 amino acid sequence from Atlantic , medaka, zebrafish and human. Sequence references can be found in Figure S10a. Amino acid residues are colour coded according to their physiochemical properties and numbering above the alignment is consecutive.

```
                        *        20         *        40         *        60         *        80
SasaERAP2a   : ----------------MVRFLVLALLSLAGV-TQTSASPTQASEPPNPT--EEQPPLGTGSLSFPWSHLRLPGYIVPLHYHL :  63
SasaERAP2b   : --------------MFW....F.........-....S............--......N.............E........ :  65
OrlaERAP2    : --------------MASIKL.E.L.C.FFL.GCHS.Q.SQ.V.SA.H.K--G.PS...EDN.....R....R..I...... :  66
DareERAP2    : ----------------.MHL.F.I..F.ESL------CYV.S..SNS..--GDN--.A.DG.P....KV...N....V.... :  56
HosaERAP2    : MFHSSAMVNSHRKPMFNIHRGFYC.TAILPQICIC.QFSVPS.YHFTED--PGAF.VA.NGER...QE....SVVI....D. :  80
SasaERAP1    : ----------------.RTLT.TI.VL.HVSFAPSL.AQLPGDHDT.DKSSSLL..IA.NGQP...I.M...ETVS.I..D. :  66
OrlaERAP1.1  : --------------------MFPL..L.FALLPSSHGAQIPSPDQ.KE------I.VA.NGQL...DRM...KT.S....D. :  56
DareERAP1    : ------------------MATMTR.PV.LNLIVLPIHLWAAVTPE.TS------F.ISSSGEP...NKM...DT.S....N. :  58
DareERAP1L   : ---------------------M.F.AKYFHFTLVALFCALF.GNHAMT------VNKNKTV.P...DKM...EIVK.Q..D. :  55
HosaERAP1    : ------------MVFLPLKWSLATMSF.LSSLLALLTVS.PSWCQSTEAS-----.KRSDGTP...NKI...E.VI.V..D. :  65

                     *       100         *       120         *       140         *       160
SasaERAP2a   : LLHPNLTMLSYSGTVRIELQVQNNTNWVVLHSKGLRITTATMLDQNLAHLS--DRVLPVLHNPTHEQTAIFSPRVLSG-GQK : 142
SasaERAP2b   : .......I...I...........................V.........--.Q...........V......A.T.-... : 144
OrlaERAP2    : V.Q....T.RFT.S.Q.QID....S........D.Q.SK..I..H.F...T--.Q........S...IG........S-... : 145
DareERAP2    : .I.....T..FT.S.K..ID.K...........N.K.Y...V..EHE....--EKT.S..EY.L...I.....KI.TS-.E. : 135
HosaERAP2    : FV.....S.DFVASEK..VL.S.A.QFII....D.E..N..LQSEEDSRYMKPGKE.K..SY.A...I.LLV.EK.TP-HL. : 161
SasaERAP1    : .V.....S.DFT.E.Q.Q...FED.STII....D.Q.AK.EL.APEGPGSL--PVP.Q..EY.AFH.L.LM.DVL.VR-.GM : 145
OrlaERAP1.1  : S......T.DFT.V...D.D.HED.SV....A.KMY.SS.LL.APAG------T...Q..EY.RFH.L.LM.DV..TK-.R. : 131
DareERAP1    : .I.....S.DFT.S.Q.QIE.LQD.KTII....N.Q.SS.RL..A.I.Q----QQP.K..EY.YFQ.I.LV.DKA.LKR.HV : 136
DareERAP1L   : .I.....S.TFT.E.Q.QIE.KQD.RAII....N.QVSK.LL.GSRQH.H---HQD.QISEFEAN..I.L..EGFTFE-KGS : 133
HosaERAP1    : .I.A...T.TFW..TKV.ITASQP.STII...HH.Q.SR..LRKGAGER..--EEP.Q..EH.RQ..I.LLA.EP.LV-.LP : 144
                   *       180         *       200         *       220         *       240

SasaERAP2a   : YFLFLEFGAELGEGFYGFYRSTYRTSAGETRNLASTHFEPTSARMAFPCFDEPSFKANYSISIRRSLAHTALSNMPVEQTVV : 224
SasaERAP2b   : .........D................T....T.....................I..........P............E. : 226
OrlaERAP2    : ...YI.....FA.......K...K..K....T........................F..Q....PQYIS.....IVK..E : 227
DareERAP2    : ...Y.....P.SD..H...K.....KS....V..............L......V.....TVR...GPS.I......L....E : 217
HosaERAP2    : .YVAMD.Q.K..D..E...K.....LG....I..V.D....Q...........L....F..K...ESR.I......KVK.IE : 243
SasaERAP1    : .KVR...S.N.SDS.H...K.S....TK..V.FM...Q..A....A........A....FT.Q...ESR.I......KVK..E : 227
OrlaERAP1.1  : .EVQ...A.N.SDSYH...K.S....T..V.VM...Q..A.F..G........A....FT.R.I.EPR.I.I....KVR..E : 213
DareERAP1    : .SVE.H.A.N.S.S.H...K......K.DV.VV...Q..A....A........A....F.VQ...EAK.I......KLR.LE : 218
DareERAP1L   : HVVH...Y.N.SDS.H...KGQ.T.NS..V.M....Q....H..A........A....FT.RV..ESR.ISI....KLR..E : 215
HosaERAP1    : .TVVIHYAGN.S.T.H...K.....KE..L.I....Q....A...........A...SF..K...EPR.L.I....LVKS.T : 226
                 *       260         *       280         *       300         *       320

SasaERAP2a   : LDDGLMEDRFAVSVRMSSYLVAFIVCDFRSVSATTASGVKVSVYAAPEKWQQTHYALKAAVKLLEFYEKYFNIKYPLPKQDL : 306
SasaERAP2b   : .............................................................................S....... : 308
OrlaERAP2    : .HG..L..H..P..K..T.....VI...K..T......I...I.......E......EV...M.D...E....P....... : 309
DareERAP2    : ISN..F..H.EA..K.....L...I...K...GL..T.INI.I..V....H..Q...E..LR.....Q....L.....L.. : 299
HosaERAP2    : .EG..L..H.ETT.K..T....Y.....H.L.GF.S......I..S.D.RN......Q.SL...D.....D.Y...S.L.. : 325
SasaERAP1    : .PG.VL..H.DT.....T.......S..Q...K..SH...I....V.D.IN..DF..N...R..D..DD..D.P....... : 309
OrlaERAP1.1  : .PGD.L..H.DTT....T....Y..S..Q...R..QH...I.I..V...IN..AF..D......D..DD..D.P....... : 295
DareERAP1    : .KN..F..Q.D...K..T....Y..S..L.I.K.SQH..QI....V...ID.AEF..D......D..DD..D.P....... : 300
DareERAP1L   : .A..IL..Q.DTM.K..T......I...H.I.KKSQH..EI...TV...IS.AE...DT..TM.D..DE..D.P.....H.. : 297
HosaERAP1    : VAE..I..H.D.T.K..T......IS..E...KI.K.........V.D.IN.AD...D...T......D..S.P....... : 308
                *       340         *       360         *       380         *       400         *

SasaERAP2a   : VAIPDFQAGAMENWGLITFRETSLLYDPATSSASDRVWVTMVIAHELAHQWFGNLVTMEWWNDIWLNEGFARYMEYISVNAT : 388
SasaERAP2b   : .......S....................T.......L...K.............................T.......DT. : 390
OrlaERAP2    : I......S........T.Y.........L...V..KL......G..............K...................E.. : 391
DareERAP2    : I.....ES........T.Y.........DI.....KL......G..............D................SV..E.V : 381
HosaERAP2    : I.....AP..........Y......F..K......KL...R......................K....K...L.A.... : 407
SasaERAP1    : A......S........T.Y..AG..F..NK.....KLGI..................Q....L.......KF..FV...I. : 391
OrlaERAP1.1  : A......S........T.Y...G..F..DR.....KLGI.K.......................L.......KF..FV.LDI. : 377
DareERAP1    : A......S........T.Y..SA..F..HK.....KLGI..I...............Q....L.......KF..FV...I. : 382
DareERAP1L   : A......S........S.Y..SG..F..EK..S..KLGI.K................Q....L.......KF...V...I. : 379
HosaERAP1    : A......S........T.Y..SA..F.AEK....SKLGI..TV....................L.......KF..FV..SV. : 390
                       420         *       440         *       460         *       480         *
```

```
SasaERAP2a  : YPKLRVEDYLVDTCFAAIGRDSLNSSRPISSAAESPTQIAEMFDTVSYDKGACVLHMLRHYLTDQVFQSGIVRYLRRYSYSN : 470
SasaERAP2b  : ..........L....V...............V.......K........N......................M.......... : 472
OrlaERAP2   : ..N.K..E..LH.......H...........P..N....K....................F...E...........K..FG. : 473
DareERAP2   : ..E.K...HFL....G...............L.......K........E....I......F...EG.....I.....F..C. : 463
HosaERAP2   : ..E.QFD..FLNV..EV.TK..........KP..T....Q....E...N....I.N..KDF.GEEK..K..IQ..KKF..R. : 489
SasaERAP1   : N.E.Q.N.FFLGK..E.MEV...S..H.V..QVDT....Q....D........I.N...DF..PEA.KI...........Q. : 473
OrlaERAP1.1 : ..E.Q.D.FFLGK..E.MEV...S..H.V.TPV.N....Q....D........I.N...DF..PEA.EI..I...K.F..Q. : 459
DareERAP1   : N.E.Q....FLGK..E.MEV...S..H.V.TPV.N.A..Q....D........I.N...EF..PKI.KL...K..KH...Q. : 464
DareERAP1L  : H.E.Q.N..FLEK..T.LSV...S..H.V.TPV.N.AE.S....D...R....I.N...DF..PE..KY..IN..KKH..Q. : 461
HosaERAP1   : H.E.K.G..FFGK..D.MEV.A....H.V.TPV.N.A..R....D........I.N...E..SADA.K....Q..QKH..K. : 472
                 500         *         520         *         540         *         560         *
SasaERAP2a  : AHNQDLWDSLANTCSEEEFTSG-----EHCYSSRQAAKNAYLYAGEHLDLTTMMNTWMLQTGVPLVTVTRQGSRLLLKQERF : 547
SasaERAP2b  : .R............P.......-----G...NS........................T..........A......V...... : 549
OrlaERAP2   : .Q.................I..-----K..NRD.SN......F........AI....T..K.I.......K.PL...R.H.. : 550
DareERAP2   : .R.E......IK.....D.AA.-----.Y....A..T....RF....V..KK.....T..K.I.....K...RK.HIG.... : 540
HosaERAP2   : .K.D....S..S.S.L.SD...-----GV.H.DPKMTS.MLAFL..NAEVKE..T..T..K.I..LV.KQD.CS.R.Q.... : 566
SasaERAP1   : TV.SH..E..T.I.QSDDLDE.RLKDEGF.SQEKAQSGAPKW.S.DQ..VRAI.D..T..E.F.....EVR.RQVR.S...Y : 555
OrlaERAP1.1 : TV.SH..E..TDI..SDDLDE.RMKHK.F.SKRKDESGASKW.S.DE..VRAI.D..T..E.F.....EVK.REVR.S.... : 541
DareERAP1   : TV.SN..E..T.V.DSDGLDE.RLKGD.S.RH.ASNTGASKWHSEDE..VKA..E..T..E.F..I.IAVK.REVR.S...Y : 546
DareERAP1L  : TV.SH..E..T.I.TSDGLD..RLKLDGF.SKHTAETPASKWFKEDSV.VGAI.D..T..E.F..I..EVK.QEVT...... : 543
HosaERAP1   : TK.E.....M.SI.PTDGVKG----MDGF.SR.QHSSSSSHWHQEG-V.VK......T..K.F..I.I.VR.RNVHM...HY : 549
                580         *         600         *         620         *         640         *

SasaERAP2a  : LRTAHPSDPAWPSLQQGFLWHIPLTYRTDTSTSIHRHLMTTLTDSVDVGEEVGWVKVNVDMAGYYLVHYDGSGWDNLIQLLK : 629
SasaERAP2b  : ...T.............Y............................E............................D...... : 631
OrlaERAP2   : .K.VL...Q.S..................A.SI.......STS..IHI...AS...I.S..T...V...EDD...VMAK.MR : 632
DareERAP2   : .KIVL.D..S.H...D.Y........K.SH.DHEVK.ILDKKS.VLLLD.H.D...L.T..N...I....EE..NA.TE... : 622
HosaERAP2   : .QGVFQE..E.RA..ERY........S.SS.NV....ILKSK..TL.LP.KTS...F...SN...I...E.H...Q..TQ.N : 648
SasaERAP1   : .K.DD.-------.QTH...QV....I.SS.STV..F.LK.R..VLYLP.....I.F....S...M...E.E..RS.TS..L : 631
OrlaERAP1.1 : ...DN.-------..TE...Q.....M.SA.R.V..F.LK.KS.VLYLP...D...F..N.S...M...G.G..NSI.S..H : 617
DareERAP1   : .KSDDL-------.QTSS..QV....I.SD..TV..F.LK.K..VLYLP...D.I.F....S...I...E.....D..T... : 622
DareERAP1L  : .KG.ES-------.NSSS..QV....I.SG.NAVQ.F.LK.ER.VFYLP.K.E.I.F...LR...I...ESG...C..KQ.R : 619
HosaERAP1   : MKGSDG------APDT.Y...V...FI.SK.DMV..F.LK.K..VLILP...E.I.F..G.N...I...EDD...S.TG... : 625

              660         *         680         *         700         *         720         *         7
SasaERAP2a  : DNHTALSFMDRTHLIHNAFQLTTAGRLSLDKALDLIGYLRSESHTVPLLEGLGYLEAFYRMVERRDIPDVTQNLRTYILWYF : 711
SasaERAP2b  : N................................................Q..A...........M.........S....... : 713
OrlaERAP2   : E......YK............VS..H...N..M.....QL.K.................NLI.KI.ELEL.KD.GR...YF. : 714
DareERAP2   : V.......K..AS........V........R.....A..K..T.N....Q.....QS..KLI.K.K.A...H..K....Q.. : 704
HosaERAP2   : Q...L.RPK..VG...DV...VG....T......MTY..QH.TSSPA.....S...S..H.MD..N.S.ISE..KR.L.Q.. : 730
SasaERAP1   : T..R...SN..AS..N.....VSV.KVG..T....SL..SK.TDIM.VTQ...E.VPL.KLM.K..MEGLENQMKG..VEL. : 713
OrlaERAP1.1 : H......GN..AS....V...VSVEKVR..T..E.SL..SR.TEIMAVWQ.F.E.VPL.KLM.K..M.ELENQMKVCGVSQ- : 698
DareERAP1   : H......SN..AS..N.....VSV.K.P.......TL..SK.TEIM.VTQ.FNE.VPL.KLM.K..MVELENQMKG...QL. : 704
DareERAP1L  : M...VF.SN..AS...DI...VSIEKVP.....N.SL..SK..EIM.VTQ.FSE.VPL.KLM.K..MQELENQ.KSHLVKL. : 701
HosaERAP1   : GT...V.SN..AS..N.....VSI.K..IE.....SL..KH.TEIM.VFQ..NE.IPM.KLM.K..MNE.ETQFKAFLIRLL : 707

             40         *         760         *         780         *         800         *         820
SasaERAP2a  : RDVIDRQTWSDKGSVSERRLRSELLSLACHLGDLPCLKQAQRSFTHWLDSNSTLNLPADVAETVYSVGAQEDTGWASLLQTY : 793
SasaERAP2b  : .G...................................E................S.....T...F.......S......HI. : 795
OrlaERAP2   : .A...Q.....S...........V.......DYP...ER.NQH.QE..Q..G.....T............H.H..N...D.. : 796
DareERAP2   : K....K.S...D.M..D....EDV.....D.DYP...EK.E.L.VS.VK..G.IS..T..S....M....D.S..VY..RQ. : 786
HosaERAP2   : KP.....S.......WD.M...A..K...D.NHA..IQK.AEL.SQ.ME.SGK..I.T..LKI.......TTA..NY..EQ. : 812
SasaERAP1   : .GL........D....Q.V...Y..LFGSVRNHP..VAT.THL.NK.RA.DGNMS..S..SLA.FAI..RDPE..DF.FEK. : 795
OrlaERAP1.1 : .EHKQNSVFNCRFDLGSSTIS--H.VAGVTNQYS-----TREMLHEIRSFFDS.TEE.GSQMRCIQQSYETIEDNIRWMDRN : 773
DareERAP1   : QKL.EQ.L.T.E......M...Y..LFG.VR.HP..VSN.TQL.NL.R..DGNMS..N..TMA.F....RTED..DF.FEK. : 786
DareERAP1L  : QPL....S...N......M..NY..LF..VRRYPS.VST.TQL.HK.KE.DGKMW..T..SLV..TE..RT.D..DF..EK. : 783
HosaERAP1   : ..L..K...T.E......M...Q..L...VHNYQ..VQR.EGY.RK.KE..GN.S..V..TLA.FA....STE..DF.YSK. : 789

                         *         840         *         860         *         880         *         900
SasaERAP2a  : THSLSETHKRKILSALASSRDTNKLTRLLELGVEGEVIRTQDLDSLIVMVARNPRGHHLAWSYVQKYWSTLVDKFQLGSFSI : 875
SasaERAP2b  : .L.......H...............H......L................................................. : 877
OrlaERAP2   : KI....AQ.N...Q..T.....G..L...Q..L..K...S...S...L......K..Y...NF.K.N.DE..E.L.....C. : 878
DareERAP2   : GV.MC..E.S.F....T..K.SE..S...Q..M..T..K..N.P...Y......V..F...DF.K.H.NE..E..P....G. : 868
HosaERAP2   : EL.M.SAEQN...Y..ST.KHQE..LK.I...M..K..K..N.AA.LHAI..R.K.QQ...DF.REN.TH.LK..D...YD. : 894
SasaERAP1   : R..QHTSV.SR.K...SI.PLQH..K..M.QSLA...MK....PHVVIS.S...K.YK...DFLRHN.H...K..D...H.. : 877
OrlaERAP1.1 : LPL.QAWLN-----KHG.RNHHQE.--------------------------------------------------------- : 793
DareERAP1   : KE.MYVSM.SR.KQ..MT.PLDH..KWMM.QSL...L.K....PYVVTS.S...KAYKH..DFL.AN.DS.IK..D...H.. : 868
DareERAP1L  : KR.V.PSE.WM.KA..SY.PLAH..QW...RSS...IMK....P.MLIS.SK..K.FK...DFLKSN.GK..K..D...SA. : 865
HosaERAP1   : QF...S.E.SQ.EF..CRTQNKE..QW..DESFK.DK.K..EFPQILTLIG...V.YP...QFLR.N.NK..Q..E...S.. : 871
```

```
                   *        920         *        940         *        960         *        980
SasaERAP2a  : RNIIIGTTAQFSSTEELTEVRVFFESIH-EQASQLRVTEVAMDNVQKNILWLQRNLGTLRSWLDQQID-------------- : 942
SasaERAP2b  : ........G..F................-.........Q.....I.....M..........N.H..-------------- : 944
OrlaERAP2   : .H.L....G....PDD....QL.....K-.......A.QI.L..MK...R.I....E...K..NE..K-------------- : 945
DareERAP2   : .E..V..VT........R..ES..K..Q-..V....IIQ..TE..E...V..K...E.M.T..QRRLN-------------- : 935
HosaERAP2   : .M..S....H...KDK.Q..KL....LE-A.G.H.DIFQTVLETIT...K..EK..P...T..MVNT--------------- : 960
SasaERAP1   : SGMVT.V.N.Y.TR.M.D...G..D.LSE.TG.G..CIQQTYESIEE..R.MDQH.PQ.KA...R.AQGARTETQGHEDL-- : 957
OrlaERAP1.1 : ---------------------------------------------------------------------------------- :   -
DareERAP1   : AHMVV.V.N.Y.TR.M.A...N..SLLQP.TGAE..CIQQ.VE.IEE..R.MDK..PL.KA..HRYY.QQKNTE-------- : 942
DareERAP1L  : SRVVV.V.D.Y.TK.M.D..EL..G.LAQD.G.G..SIQQ.LEKI.Q....MD..VPL.KA...HHTS-------------- : 933
HosaERAP1   : AHMVM...N...TRTR.E..KG..S.LK-.NG....CVQQTIETIEE..G.MDK.FDKI.V..QSEKLERM----------- : 941
```

References:

1. Saitou N & Nei M (1987) The neighbor-joining method: a new method for reconstructing phylogenetic trees. *Molecular biology and evolution* 4(4):406-425.
2. Felsenstein J (1985) Confidence Limits on Phylogenies: An Approach Using the Bootstrap. *Evolution; international journal of organic evolution* 39(4):783-791.
3. Zuckerkandl E & Pauling L (1965) Molecules as documents of evolutionary history. *Journal of theoretical biology* 8(2):357-366.
